# Viral vector delivered immunogen focuses HIV-1 antibody specificity and increases durability of the circulating antibody recall response

**DOI:** 10.1371/journal.ppat.1011359

**Published:** 2023-05-31

**Authors:** LaTonya D. Williams, Xiaoying Shen, Sheetal S. Sawant, Siriwat Akapirat, Lindsay C. Dahora, Matthew Zirui Tay, Sherry Stanfield-Oakley, Saintedym Wills, Derrick Goodman, DeAnna Tenney, Rachel L. Spreng, Lu Zhang, Nicole L. Yates, David C. Montefiori, Michael A. Eller, David Easterhoff, Thomas J. Hope, Supachai Rerks-Ngarm, Punnee Pittisuttithum, Sorachai Nitayaphan, Jean-Louis Excler, Jerome H. Kim, Nelson L. Michael, Merlin L. Robb, Robert J. O’Connell, Nicos Karasavvas, Sandhya Vasan, Guido Ferrari, Georgia D. Tomaras, RV305 study team

**Affiliations:** 1 Center for Human Systems Immunology, Duke University School of Medicine, Durham, North Carolina, United States of America; 2 Department of Surgery, Duke University School of Medicine, Durham, North Carolina, United States of America; 3 Duke Human Vaccine Institute, Duke University School of Medicine, Durham, North Carolina, United States of America; 4 Department of Retrovirology, US Army Medical Directorate, Armed Forces Research Institute of Medical Sciences, Bangkok, Thailand; 5 Department of Immunology, Duke University School of Medicine, Durham, North Carolina, United States of America; 6 Department of Molecular Genetics Microbiology, Duke University School of Medicine, Durham, North Carolina, United States of America; 7 US Military HIV Research Program, Walter Reed Army Institute of Research, Silver Spring, Maryland, United States of America; 8 Henry M. Jackson Foundation for the Advancement of Military Medicine, Bethesda, Maryland, United States of America; 9 Department of Medicine, Duke University School of Medicine, Durham, North Carolina, United States of America; 10 Department of Cell and Developmental Biology, Feinberg School of Medicine, Northwestern University, Chicago, Illinois, United States of America; 11 Thai Ministry of Public Health, Nonthaburi, Thailand; 12 Royal Thai Army Component, Armed Forces Research Institute of Medical Sciences, Bangkok, Thailand; Vaccine Research Center, UNITED STATES

## Abstract

The modestly efficacious HIV-1 vaccine regimen (RV144) conferred 31% vaccine efficacy at 3 years following the four-shot immunization series, coupled with rapid waning of putative immune correlates of decreased infection risk. New strategies to increase magnitude and durability of protective immunity are critically needed. The RV305 HIV-1 clinical trial evaluated the immunological impact of a follow-up boost of HIV-1-uninfected RV144 recipients after 6–8 years with RV144 immunogens (ALVAC-HIV alone, AIDSVAX B/E gp120 alone, or ALVAC-HIV + AIDSVAX B/E gp120). Previous reports demonstrated that this regimen elicited higher binding, antibody Fc function, and cellular responses than the primary RV144 regimen. However, the impact of the canarypox viral vector in driving antibody specificity, breadth, durability and function is unknown. We performed a follow-up analysis of humoral responses elicited in RV305 to determine the impact of the different booster immunogens on HIV-1 epitope specificity, antibody subclass, isotype, and Fc effector functions. Importantly, we observed that the ALVAC vaccine component directly contributed to improved breadth, function, and durability of vaccine-elicited antibody responses. Extended boosts in RV305 increased circulating antibody concentration and coverage of heterologous HIV-1 strains by V1V2-specific antibodies above estimated protective levels observed in RV144. Antibody Fc effector functions, specifically antibody-dependent cellular cytotoxicity and phagocytosis, were boosted to higher levels than was achieved in RV144. V1V2 Env IgG3, a correlate of lower HIV-1 risk, was not increased; plasma Env IgA (specifically IgA1), a correlate of increased HIV-1 risk, was elevated. The quality of the circulating polyclonal antibody response changed with each booster immunization. Remarkably, the ALVAC-HIV booster immunogen induced antibody responses post-second boost, indicating that the viral vector immunogen can be utilized to selectively enhance immune correlates of decreased HIV-1 risk. These results reveal a complex dynamic of HIV-1 immunity post-vaccination that may require careful balancing to achieve protective immunity in the vaccinated population.

**Trial registration**: RV305 clinical trial (ClinicalTrials.gov number, NCT01435135). ClinicalTrials.gov Identifier: NCT00223080.

## Author summary

One strategy for improving vaccine-elicited antibody durability is booster immunization. However, the impact of boosting on the quality of the polyclonal antibody response is not fully understood and may alter vaccine efficacy. We discovered that canarypox vector boost (ALVAC-HIV vectored vaccine component only) drove the recall of HIV-1-specific inverse correlate of risk (V2 IgG) and promoted more durable immunity. We found that delayed boosting increased the concentration of putative protective V1V2-specific IgG circulating in blood, enabling 80% of vaccinees to achieve this level in RV305 compared to 63% in RV144. In contrast to Env IgG1 and IgA1, Env IgG3 was not boosted. However, Env IgG1 correlated with the magnitude of the ADCP response, demonstrating that antibody function can be elevated independent of IgG3 and IgA1. By including a delayed boost as part of a HIV-1 prime-boost vaccine strategy, vaccine-elicited antibodies can reach the putative protective threshold in a larger proportion of the population. However, a second immunogen boost within a 6-month interval shifts the antibody isotype/subclass profile, resulting in decreased antibody functions. A canarypox prime regimen can drive antibody specificity and function. Our study informs selection of immunogen types and immunization intervals for HIV-1 vaccine developers to elicit protective immunity.

## Introduction

Elicitation of durable, broadly protective immune responses is a key goal for human immunodeficiency virus type-1 (HIV-1) vaccine development. Results from the modestly protective phase III RV144 HIV-1 vaccine efficacy trial in Thailand (ClinicalTrials.gov NCT00223080) catalyzed efforts to define immune correlates of risk, which lent insights into the mechanistic underpinnings of protection [1,2]. The canarypox ALVAC-HIV (vCP1521) prime (four doses) and alum-adjuvanted AIDSVAX B/E gp120 protein boost regimen (two doses), administered to more than 16,000 Thai heterosexual adults at low risk of HIV-1 infection, afforded 60.5% vaccine efficacy at 12 months (post-hoc analysis) [3]. Analysis of HIV-1-infected and uninfected vaccine recipients revealed that high levels of binding plasma immunoglobulin A (IgA) (monomeric) antibodies to certain regions of HIV-1 Envelope (Env) [constant region 1 (C1), subtype A gp140)] correlated directly with the rate of infection (decreased vaccine efficacy) [2]. Statistical modeling showed interaction of low IgA levels with four immune response variables (antibody-dependent cellular cytotoxicity (ADCC) [4], IgG avidity, tier 1 neutralization, Env-specific CD4 T cells) correlated with reduced risk of infection [2]. Further studies identified linear variable region 2 (V2) [2,5] and variable region 3 (V3)-directed antibodies [6] (in the presence of low IgA and neutralizing antibodies) [5], variable regions 1 and 2 (V1V2) IgG3 [7], V1V2-specific antibody-dependent complement activation [8], and Env CD4 T cell polyfunctionality [9] as correlates of decreased HIV-1 risk. Antibody Fc effector functions [10] including ADCC [11,12], virion capture [13], and antibody-dependent cellular phagocytosis (ADCP) [14–17], and Fc receptor genotype [18] are also likely to have contributed to protective immunity against HIV-1 in RV144. Sieve analysis of HIV-1 breakthrough viruses in vaccine and placebo recipients who became infected identified sites of immune pressure against viruses matching the vaccine at lysine position 169 and isoleucine position 181 in V2 (K169 and I181) and I307 in V3 that were associated with increased vaccine efficacy (48%, 78%, and 52% for strains matching K169, mismatching I181, and matching I307, respectively), highlighting that V2 and V3 contain sequence-specific protective epitopes [6,19,20]. Vaccine efficacy waned to 31.2% at 42 months of follow-up [1]. Coincident with decreased efficacy was the waning of antibodies associated with lower HIV-1 acquisition risk: plasma IgG antibodies recognizing V1V2 of the HIV-1 Env glycoprotein [2,21], which declined to below detectable levels 6–12 months post last RV144 immunization [7,22,23]. Thus, prolonging durability of responses associated with decreased HIV-1 acquisition risk in RV144 may guide vaccine development towards improved efficacy.

In a previous study comparing two ALVAC vector-prime/protein-boost vaccine regimens, utilizing different strains for both the vector and protein immunogens (HVTN 097 and HVTN 100 trials conducted in South Africa), we found that the viral sequence influenced antibody specificity, with higher elicitation of V2-specific antibodies observed in HVTN 097 (used the same regimen as the RV144 trial) compared to HVTN 100 (used an adapted clade C regimen) [24]. These results indicate a role for ALVAC-HIV vCP1521 vector prime in targeting V2 specificities such as the linear V2 hotspot (V2.hs) that correlated with decreased HIV-1 risk.

The RV305 HIV-1 clinical trial (ClinicalTrials.gov NCT01435135) was designed to evaluate the impact of late boosting of HIV-1-uninfected RV144 recipients after 6–8 years with the vaccine components used in the RV144 trial. RV305 participants were randomly assigned to one of three arms and boosted with ALVAC-HIV plus AIDSVAX B/E (group 1), AIDSVAX B/E alone (group 2), or ALVAC-HIV alone (group 3) or placebo at weeks 0 and 24. Late boosting of RV144 vaccinees with ALVAC-HIV/AIDSVAX B/E or AIDSVAX B/E alone resulted in increased plasma IgG and IgA titers against gp120 and scaffolded gp70 V1V2 vaccine-matched antigens, tier 1 neutralizing antibody titers, and improved CD4 T cell functionality [25]. However, titers post second boost declined to lower levels than post first boost in these groups. Evaluation of binding antibody responses in cervicovaginal mucus, seminal plasma, and rectal fluids showed correlation with plasma levels [26]. Monoclonal antibodies (mAbs) isolated from RV305 vaccine recipients demonstrated increased variable heavy chain complementarity determining region 3 (HCDR3) lengths and mutation frequencies compared to those isolated from the RV144 trial, structural and genetic features that could favor affinity maturation toward conserved epitopes and antibody penetration into partially occluded conserved regions of HIV-1 Env [27,28]. The isolated mAbs mapped to the CD4 binding site, V2, V3, C1C2, and gp120 regions of HIV-1 Env, comprising memory B cell clonal lineages that were present after the initial RV144 immunization regimen and persisted after boosting [28–30]. V2-specific mAbs mediated ADCP, increased breadth and potency of ADCC against RV144 breakthrough viruses, and blocked Env α_4_β_7_ integrin binding, which has been postulated as a potentially protective mechanism to inhibit viral transmission [28,31–33]. Furthermore, boosting of RV144 recipients in RV305 with AIDSVAX B/E enhanced Env C1C2-specific mAb ADCC breadth and potency [29].

How subsequent vaccine immunogen boosting impacts the subclass composition of IgG and IgA antibodies and Fc-dependent antibody effector functions is area of active investigation for all types of vaccines. In this study, we hypothesized that the specificity, breadth, and function of antibody responses associated with protective immunity could be improved with an HIV-1 immunogen boost years after the initial vaccination. We pre-specified the primary hypotheses that the magnitudes of each antibody type (IgG, IgG1, IgG3, and IgG4) elicited to vaccine-matched antigens (92TH023 gp120 gD 293F mon, A244 D11 gp120_avi, AE.A244 V1V2 tags, MN gp120 gDneg/293F) would be (i) higher after the first RV305 boost compared to RV144 peak immunogenicity, (ii) higher after the second RV305 boost compared to after the first boost, and (iii) higher for the ALVAC-HIV + protein (gp120) group compared to the protein only group after the first and second boosts. Furthermore, that (iv) Env breadth for IgG and IgG3 antibodies would be higher after the first boost compared to RV144 and that (v) V1V2 breadth or concentrations for IgG and IgG3 after boosting would exceed those measured in RV144. Exploratory analyses included breadth of binding to linear V2 epitopes, Fc-mediated antibody functions including ADCP activity, ADCC activity, virion capture, and association of humoral and cellular immunity. We also explored whether ALVAC-HIV only boosting focused HIV-1 antibody specificity to V2, a correlate of decreased HIV-1 risk in RV144.

## Results

### ALVAC-HIV boost directs antibody specificity towards V2 and V3, and CD4i binding antibodies

**Fig 1:**
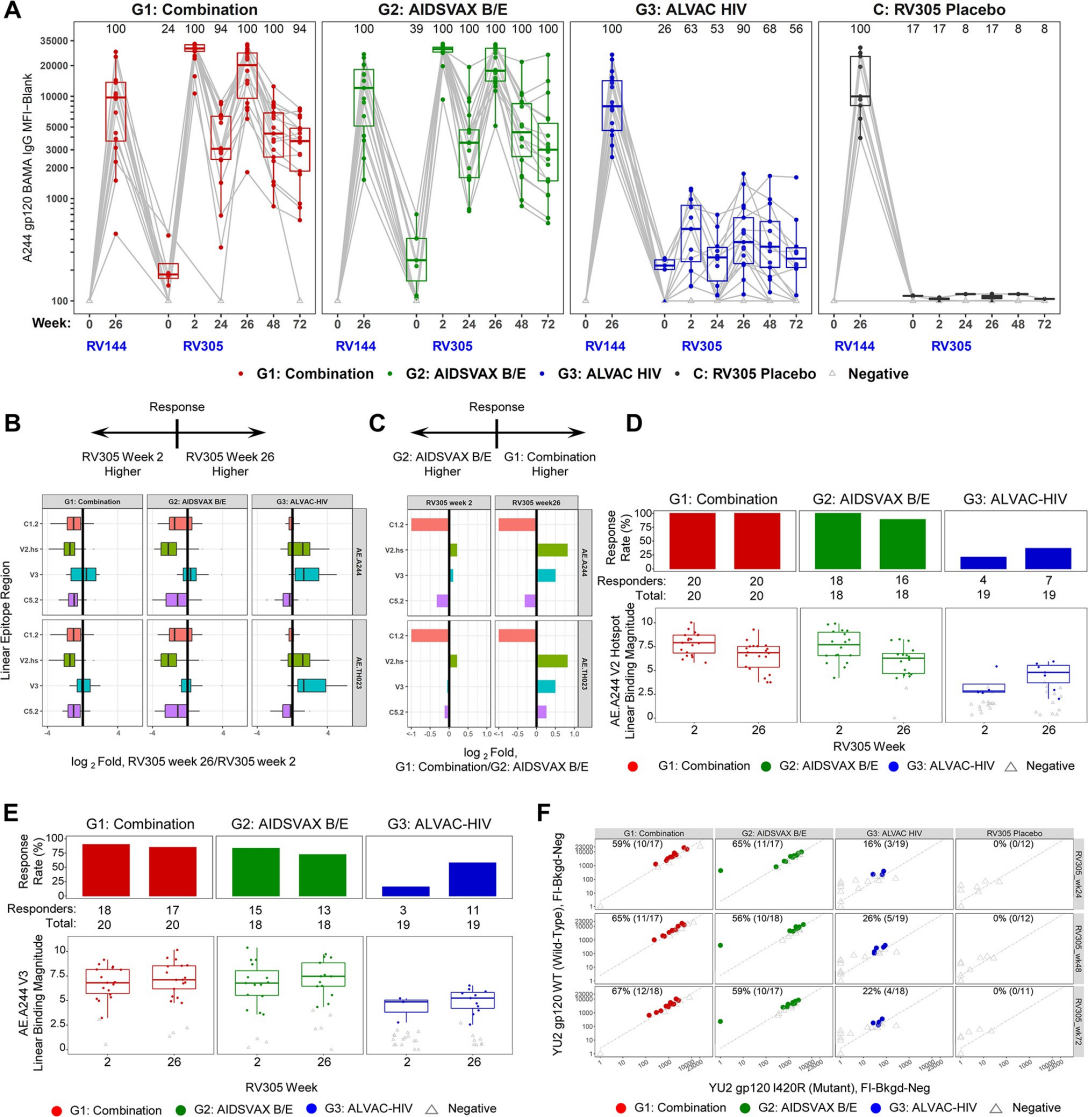
ALVAC-HIV elicits V2, V3, and CD4 inducible (CD4i) binding antibody responses. (**A**) Magnitude and kinetics of plasma IgG to A244 D11 gp120 (vaccine strain boost immunogen) measured by BAMA in 70 RV305 participants at RV144 weeks 0 (pre-vaccination) and 26 (2 weeks post final RV144 vaccination) and RV305 weeks 0 (RV305 baseline; time point of first RV305 boost), 2 (two weeks post RV305 first boost), 24 (time point of second RV305 boost), 26 (two weeks post second RV305 boost), 48 (6 months post second boost), and 72 (1 year post second boost). IgG BAMA response magnitude is expressed as mean fluorescence intensity (MFI) after blank bead subtraction (MFI-Blank). Red, Combination group (ALVAC-HIV + AIDSVAX B/E) (n = 20 vaccinees); green, AIDSVAX B/E only group (n = 18 vaccinees); blue, ALVAC-HIV only group (n = 19 vaccinees); black, RV305 placebo group (RV144 vaccinees administered RV305 placebo) (n = 13 participants). Boxplots depict the median (midline) and 25^th^ and 75^th^ percentiles, with the colored symbols indicating the response for a single participant measured at a 1:50 dilution. Open gray triangles indicate negative responders. Gray lines connect the response from a single participant between time points. Response rates at each time point are shown at the top of each plot.(**B**) Log_2_ fold difference in post second boost (RV305 week 26) / post first boost (RV305 week 2) plasma IgG binding to linear epitopes in C1.2, V2 hotspot (V2.hs), V3, and C5.2 within AE.A244 and AE.TH023 gp120 assessed by peptide microarray mapping assay. Horizontal bar pointing to the left of the x = 0 line (solid black vertical line) indicates a higher response magnitude measured at RV305 week 2 compared to RV305 week 26; horizontal bar pointing the right indicates a higher response magnitude measured at RV305 week 26 versus RV305 week 2. (**C**) Log_2_ fold difference in Combination (ALVAC-HIV + AIDSVAX B/E) group / AIDSVAX B/E only group plasma IgG binding to linear epitopes in C1.2, V2.hs, V3, and C5.2 within AE.A244 gp120 and AE.92TH023 gp120 assessed by peptide microarray mapping assay. Horizontal bar pointing to the left of the x = 0 line at indicates a higher response magnitude measured in the AIDSVAX B/E only group; horizontal bar pointing to the right indicates a higher response magnitude measured in the Combination group. (**D**) Response rate and magnitude of post first boost (RV305 week 2) and post second (RV305 week 26) boost plasma IgG binding to linear AE.A244 V2.hs. The number of positive responders is shown over the total number of individuals analyzed at each time point, represented as a bar graph displaying percent responders. Box plots depict the median (midline) and 25^th^ and 75^th^ percentiles, with the colored symbols indicating the epitope mapping response magnitude for a single participant. (**E**) Response rate and magnitude of plasma IgG binding to AE.A244 V3 linear peptide. (**F**) Prevalence of CD4-induced (CD4i) IgG antibodies among RV305 participants. Differential binding plots displaying BAMA MFI-Blank values for IgG binding to YU2 gp120 WT (y-axis) and YU2 gp120 I420R mutant (x-axis) proteins at RV144 week 26 and RV305 weeks 0, 2, and 26. The diagonal dashed gray line indicates a wild-type to mutant binding ratio of 2.5 (cut-off for positivity). The CD4-induced (CD4i) monoclonal antibody 17b was used as a positive control for YU2 gp120 WT/I420R differential binding. Colored symbols represent positive responders with differential binding ratios of ≥ 2.5, indicating the presence of CD4i specificities. Response rate (percent responders over the total number of participants analyzed) is shown at the top of each plot.

Although Rerks-Ngarm et al. [25] reported no boosting of responses observed in ALVAC-HIV only recipients, further examination of the specificities of RV305 vaccine-elicited antibodies in plasma, targeting conformational and linear epitopes, could provide a better understanding of the contribution of ALVAC-HIV and the protein boost to the vaccine-induced binding antibody response. Thus, we used a binding antibody multiplex assay (BAMA), sensitive in the nanogram per milliliter range for detection of antigen-specific antibodies in plasma [7], to assess total IgG responses in a subset of 70 RV305 participants (including 57 vaccine and 13 placebo recipients distributed across the three treatment arms). Env-specific binding antibody responses were evaluated in longitudinal plasma samples collected at study entry (RV305 week 0; time point of first boost), 2 weeks post first boost (RV305 week 2), at second boost (RV305 week 24), 2 weeks, 6 months, and 12 months post second boost (RV305 weeks 26, 48, and 72, respectively) and compared with matching RV144 samples obtained at RV144 baseline (week 0) and 2 weeks post last RV144 immunization (RV144 week 26). Analysis of plasma obtained at RV305 week 0 revealed low magnitude total IgG antibodies to the protein boost antigen, A244 gp120 (**Fig 1A**), indicating persistence of low level of antibody responses over the 6-8-year rest interval. This result is in agreement with an earlier study demonstrating weak binding and neutralizing antibody responses measured at RV305 baseline [25]. As expected, median response magnitudes in the ALVAC-HIV/AIDSVAX B/E and AIDSVAX B/E only groups rose to levels higher than RV144 peak (RV144 week 26) after the first boost (RV305 week 2), declining post second boost (RV305 week 26) (**Fig 1A**). Notably, as quantified by BAMA, the ALVAC-HIV only boost elicited low magnitude total IgG, with 63.2% and 89.5% response rates against A244 gp120 elicited post first and second boosts, respectively (RV305 weeks 2 and 26, respectively), above the level of binding observed in placebo recipients (response rates of 16.7% at RV305 weeks 2 and 26) (**Fig 1A and S1 Table**).

The binding antibody repertoire in RV144 consisted of four dominant linear epitope specificities (C1, V2, V3, and C5 regions in gp120), among which V2-directed antibodies of multiple subtypes, including a core hotspot region within V2 (amino acids 161–179) which correlated with decreased risk of HIV-1 acquisition [5,34]. V3 CRF01_AE peptide-specific IgG was identified as an additional correlate of reduced infection risk in RV144 in subjects with low levels of plasma IgA and neutralizing antibodies [5]. To evaluate IgG responses to linear epitopes induced by RV305, we performed peptide microarray mapping assays for the subset of 70 participants against a library containing overlapping peptides covering seven full length HIV-1 Env gp160 consensus sequences (clades A, B, C, D, group M, CRF01_AE, and CRF02_AG) and six multi-clade virus strain gp120 sequences. Plasma IgG collected from ALVAC-HIV/AIDSVAX B/E and AIDSVAX B/E only recipients post first and second boosts (RV305 weeks 2 and 26, respectively) bound to linear epitopes in C1, V2, V3, and C5 regions of gp120, with V3 and C5.2 comprising the dominant specificities to Envelope and V2 co-dominant against AE.A244 and AE.TH023 (**S1A and S1B Fig and S2 Table**). Median binding intensities across C1, V2, and C5 decreased post second boost whereas reactivity against V3 either increased, decreased, or remained relatively unmodified post second boost, depending on the antigen (**Figs 1B, S1A, S1C, and S2**). ALVAC-HIV/AIDSVAX B/E and AIDSVAX B/E only groups showed overall comparable binding to vaccine strain linear epitopes (AE.A244, B.MN, and AE.TH023), with the AIDSVAX B/E only group demonstrating higher binding magnitudes and response rates to C1.2 post first and second boosts (**Figs 1C, S1 and S2**). The ALVAC-HIV only group showed lower levels of linear binding antibody responses compared to ALVAC-HIV/AIDSVAX B/E and AIDSVAX B/E only groups (**S1A Fig**). However, binding responses to V2 hotspot and V3 linear epitopes increased in both magnitude and frequency from post first boost to post second boost (**Figs 1B, 1D–1E and S2**). In particular, ALVAC-HIV only response rates to V2 hotspot against AE.A244 and AE.TH023 increased from 21% post first boost to 37% post second boost, with V3 response rates increasing from 16–21% post first boost to 53–58% post second boost (**S3 Table and S2 Fig**). B.MN-specific V2 hotspot and V3 responses were not detected in the ALVAC-HIV only group, consistent with absence of this B.MN sequence in the RV144 ALVAC-HIV (vCP1521) canarypox vector prime (**S2 Fig**).

We previously reported that RV144 elicited IgG antibodies to conformational epitopes against the CD4 binding site (CD4bs) and CD4-inducible (CD4i) epitopes [2]. The identification of monoclonal antibodies against the CD4bs in RV305 vaccinees [27] suggests the presence of circulating antibodies targeting this region that can be boosted to levels higher than those attained by the primary RV144 vaccine regimen. However, the prevalence of circulating antibodies that recognize epitopes exposed on trimeric Env after CD4 engagement (CD4i epitopes) in RV305 vaccine recipients has not been previously studied. To determine whether late boosting improved antibody recognition of CD4bs and CD4i epitopes, we measured IgG to a panel of recombinant CD4bs and CD4i wild-type and mutant differential binding proteins in longitudinal plasma samples by BAMA. No RSC3-reactive CD4bs-directed antibodies were detected during RV305 (**S3 Fig**). CD4i antibodies developed at comparable frequencies in ALVAC-HIV/AIDSVAX and AIDSVAX B/E only recipients–ranging from 0–6% at RV144 peak (**S4 Fig**), expanding to a frequency of 59–65% by 24 weeks post first RV305 boost, and persisted at rates of 56–67% by 6 months post second boost (RV305 week 48) and 59–67% at 1 year post second boost (RV305 week 72) (**Fig 1F**). Although we did not have data on the magnitude of the CD4i response at two weeks post the second boost (RV305 week 26), the persistence of these specificities out one year from that second boost in over half of the vaccinees is remarkable. Notably, the ALVAC-HIV only vaccinees also developed CD4i-specific antibodies that reached 26% at week 48 and were maintained at a 22% response rate at week 72 (**Fig 1F**).

### Delayed boosting with ALVAC-HIV/AIDSVAX B/E or AIDSVAX B/E increases V1V2-specific IgG concentration and breadth

**Fig 2:**
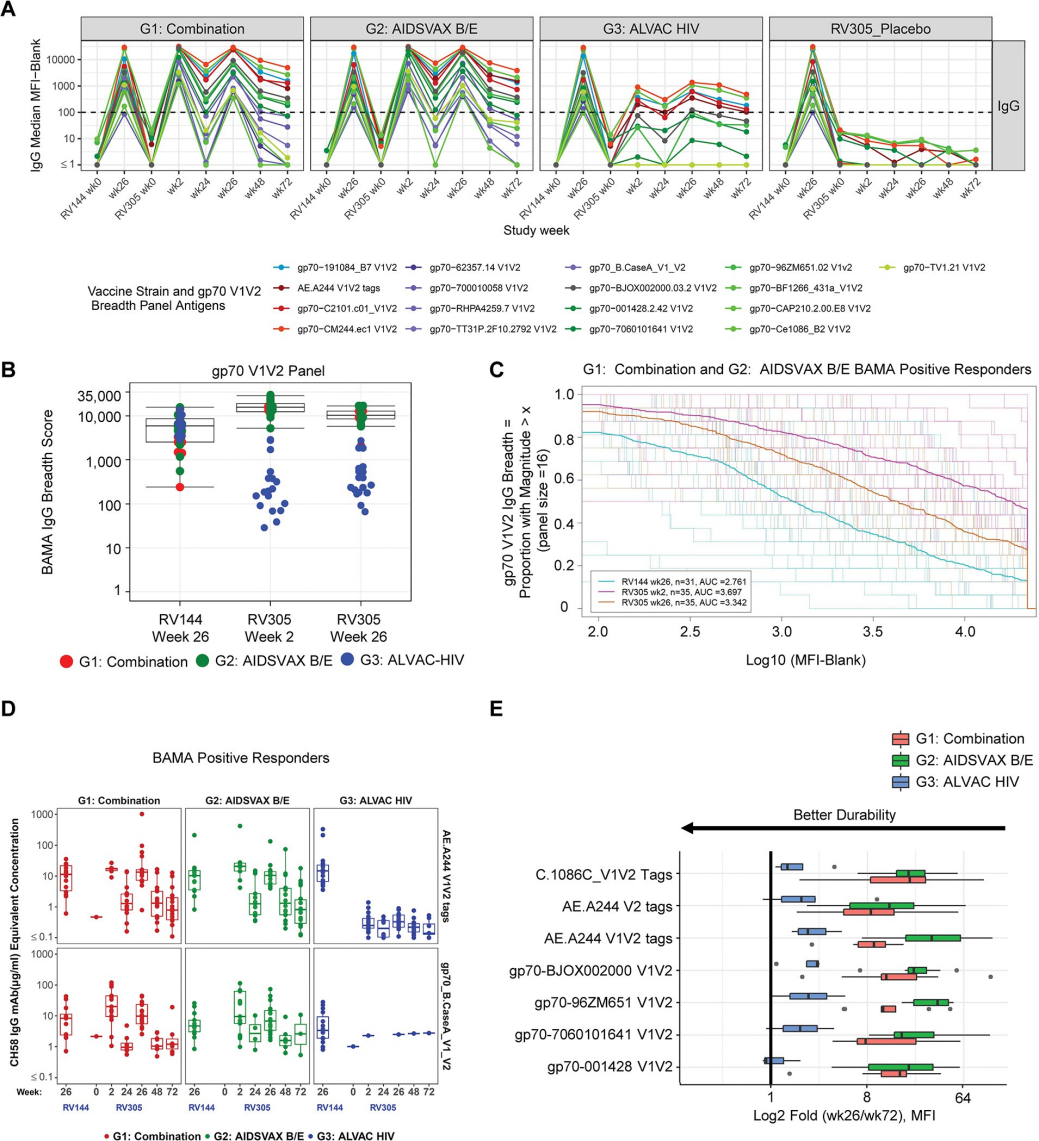
Delayed boosting with ALVAC-HIV/AIDSVAX B/E or AIDSVAX B/E increases V1V2-specific IgG concentration and breadth. (**A**) Longitudinal plasma IgG binding antibody responses to AE.A244 V1V2 tags (V1V2 from RV144 vaccine boost–A244 gp120) and a panel of 16 geographically and genetically diverse gp70 V1V2 scaffold proteins representing global HIV-1 diversity determined by BAMA. Group median MFI among positive responders is plotted for each strain, color-coded by HIV-1 subtype; blue, clade A; red, CRF01_AE; purple, clade B; gray, CRF01_BC, green, clade C. Dotted black horizontal line, showing MFI equal to 100 indicates the minimum threshold for positivity for individual samples. (**B**) BAMA IgG breadth scores for binding to the gp70 V1V2 breadth panel. Scores were calculated based on averaging the mean fluorescence intensities (MFIs) of the individual antigens in the panel. Each symbol represents the breadth score for a single vaccine recipient. Red, Combination group (ALVAC-HIV + AIDSVAX B/E); green, AIDSVAX B/E only group; blue, ALVAC-HIV only group. Boxplots depict the median (midline) and 25^th^ and 75^th^ percentiles for the Combination (ALVAC-HIV/AIDSVAX B/E) and AIDSVAX B/E only groups. Differences in breadth scores between RV144 and RV305 post boost time points was assessed using the two-sided Wilcoxon Signed Rank test, combining data for Combination and AIDSVAX B/E only groups (Table 1). (**C**) Magnitude-breadth (MB) plot showing the number of antigens in the V1V2 panel (n = 16) with positive binding (breadth) (y-axis) at a given response magnitude (log10 binding antibody MFI) (x-axis) among positive responders in the Combination (ALVAC-HIV + AIDSVAX B/E) and AIDSVAX B/E only groups. Dashed lines display MB curves for each individual plasma sample measured at RV144 week 26 (turquoise), RV305 week 2 (pink), and RV305 week 26 (orange). Solid bold lines show the median MB among positive responders at each immunization time point. AUC values summarize the MB at a given time point across the entire range of MFI values. (**D**) Plasma IgG concentrations to V1V2 antigens associated with RV144 vaccine efficacy extrapolated by 4-parameter logistic (4-PL) regression of V2-specific monoclonal antibody CH58 standard curve titrations run in each BAMA. Concentrations are plotted in μg/mL for positive responders in each group across the studied immunogenicity time points, with each dot representing the concentration for a single plasma sample. The midline of the box plot denotes the median concentration, and the ends of the box plot denote the 25^th^ and 75^th^ percentiles among positive responses. **(E)** Durability of V1V2 IgG responses. Fold decline in binding V1V2 IgG MFI between 2 weeks after the last RV305 boost (week 26) to 12 months post last boost (week 72). Results are presented as log_2_ fold change, with the midline of the box plots indicating median and ends of the box plots indicating the 25^th^ and 75^th^ percentiles. The whiskers denote the minimum and maximum data points no more than 1.5 times the interquartile range (IQR). Black dots represent data points that lie outside of the median ± 1.5 times the IQR. Criteria for the fold (wk26/wk72) calculation: 1) response is positive at week 26, 2) MFI < 23000 at week 26, 3) MFI > 100 at week 72. Antigens with greater than or equal to 6 data points meeting this criteria for both the Combination (ALVAC-HIV/AIDSVAX B/E) and AIDSVAX B/E only groups are plotted for each vaccine boost regimen. Proximity of the bar to the y-axis indicates better durability.

To delineate the diversity of the V1V2 response, we used BAMA to measure total IgG against a global panel of HIV-1 V1V2 antigens representing multiple clades and circulating recombinant forms (CRF) (2, 7). ALVAC-HIV/AIDSVAX B/E and AIDSVAX B/E only RV305 boosts induced comparable cross-clade V1V2 IgG, two weeks post first boost (RV305 week 2), that was similar or higher in magnitude than the peak RV144 response (week 26) (depending on the antigen), with titers that declined post second boost (**Fig 2A**). V1V2-specific IgG antibodies were present in up to 94% and 100% ALVAC-HIV/AIDSVAX B/E and AIDSVAX B/E only vaccinees, respectively, 1 year post second boost (**S1 Table**). The ALVAC-HIV only group demonstrated low level IgG responses against certain clade A (gp70-191084_B7 V1V2), CRF01_AE (AE.A244 V1V2 tags, AE.A244 V2 tags, gp70-C2101.c01_V1V2, and gp70-CM244.ec1 V1V2), and clade C (C.1086C V1V2 tags, gp70-Ce1086_B2 V1V2) V1V2 strains, with response rates that ranged from 47–100% at two weeks post first boost (week 2), 78–100% at two weeks post second boost (week 26), and 22–83% at 1 year post second boost (week 72) against these antigens (**Fig 2A and S1 Table**). As expected, median response magnitudes for the placebo group (i.e. RV144 vaccine only group) did not increase and response rates did not exceed 16.7% at any RV305 sampling time point (**Fig 2A and S1 Table**).

The durability of IgG V1V2 binding antibody responses during RV305 was analyzed as the fold decline of responses from week 26 to week 72 (between the last boost and final sampling time point) among positive responders for antigens with at least six data points per group. The AIDSVAX B/E only group showed a trend towards greater fold decline compared to the ALVAC-HIV/AIDSVAX B/E group (**Fig 2E**), indicating that ALVAC-HIV may drive the longevity of V1V2 IgG responses. Among the vaccine groups, the ALVAC-HIV only group showed the best durability of V1V2 IgG, albeit low, supporting the hypothesis that inclusion of ALVAC-HIV in the late boost may modulate the longevity of responses (**Fig 2E**).

We next probed whether IgG V1V2 binding antibody breadth improved following the first and second RV305 boosts. Breadth scores were calculated as the mean of the BAMA mean fluorescence intensities (MFI) of antigens across a standard HIV-1 envelope panel consisting of 16 V1V2 scaffold proteins representing global HIV-1 diversity [23]. V1V2 IgG binding breadth significantly increased approximately 2.6-fold after the first RV305 boost (week 2) with ALVAC-HIV/ AIDSVAX B/E or AIDSVAX B/E alone compared to RV144 peak (week 26) (**Table 1**, FDR *P* < 0.0001) (**Fig 2B**). Following the second boost (RV305 week 26), IgG breadth declined approximately 1.5-fold in these groups but was significantly higher than breadth measured at RV144 peak (**Table 1**, *P* <0.0001). Breadth of IgG binding to V1V2 in the ALVAC-HIV only group was not enhanced with boosting (**Fig 2B**).

**Table 1:** Primary and exploratory statistical analysis: comparison of binding antibody multiplex assay (BAMA) IgG breadth scores at RV144 week 26 and RV305 week 2 (primary analysis) and RV144 week 26 and RV305 week 26 (exploratory analysis).

			Primary Analysis Tier		Exploratory Analysis Tier
			Comparison: Log breadth score at RV144_wk26—RV305_wk2		Comparison: Log breadth score at RV144_wk26—RV305_wk26
**Isotype**	**Variable**	**Breadth Panel**	**Raw.*P*** ^**a**^	**FDR.*P*** ^**b**^	**Raw.*P*** ^**a**^
IgG	Magnitude	V1V2	**<0.0001**	**<0.0001**	**<0.0001**
IgG	Magnitude	gp120	**<0.0001**	**<0.0001**	**<0.0001**
IgG	Magnitude	gp140	**<0.0001**	**<0.0001**	**<0.0001**

Data from participants in group 1 (received ALVAC-HIV + AIDSVAX B/E boost) and group 2 (received AIDSVAX B/E only boost) was combined to compare between two time points.

Breadth scores were calculated as the mean of the log transformed MFIs of the individual antigens in a given panel.

^a^*P* values determined by Wilcoxon Signed Rank Test, with *P* values less than 0.05 shown in bold font.

^b^FDR.*P* is the *P* value post correction for multiple comparisons using the Benjamini and Hochberg false discovery rate method.

To further evaluate the proportion of globally diverse strains targeted by IgG V1V2-specific antibodies, we plotted magnitude-breadth (MB) curves for AIDSVAX B/E/ALVAC-HIV and AIDSVAX B/E only positive responders (combined for vaccinees in both groups) as a composite measure of the fraction of the 16 antigen V1V2 panel (breadth) bound at a given magnitude. Area under the MB curve values increased 1.3-fold from RV144 week 26 (AUC = 2.761) to RV305 week 2 (AUC = 3.697) and declined 1.1-fold by RV305 week 26 (AUC = 3.342), indicating overall greater MB at RV305 post boost time points compared to RV144 (**Fig 2C**). Moreover, a higher fraction of vaccinees demonstrated broad binding at higher magnitudes after booster immunization, with approximately 50% and 30% V1V2 breadth achieved post first and second RV305 boosts, respectively, at the highest magnitude of the binding response (at 4.34 log10 MFI) compared to approximately 10% breadth seen at RV144 peak at this same magnitude (**Fig 2C**).

RV144 vaccine recipients with high V1V2 scaffold IgG antibody titers were more likely to be protected than those with lower titers, indicating an association between peak concentration and vaccine efficacy [21,35]. We previously reported that V1V2 IgG concentration of >2.98 CH58 μg/mL was associated with decreased HIV-1 risk [35]. To determine the relevance of plasma V1V2 IgG concentrations boosted by RV305 vaccination to potential protective levels of V1V2 response, we calculated microgram per milliliter (μg/mL) concentrations based on V2 monoclonal antibody CH58 standard curves run in each assay. Plasma V1V2 IgG concentrations to AE.A244 V1V2 tags, gp70 B.case A V1V2, and gp70 B.caseA2 V1 V2 169K (antigens correlated with RV144 vaccine efficacy) were boosted in the ALVAC-HIV/AIDSVAX B/E (1.5-fold, 2.4-fold, and 2.4-fold, respectively) and AIDSVAX B/E only (2.0-fold, 2.0-fold, and 5.5-fold, respectively) groups after the first RV305 boost compared to RV144 but declined approximately 1.3–3.4-fold against these antigens after the second boost albeit still higher than RV144 (**Figs 2D and S5 and S4 Table**). Median concentrations in μg/mL for positive responders in both groups were [formatted as (Combination group median concentration, AIDSVAX B/E only group median concentration)]: RV144 week 26: AE.A244 V1V2 tags (11.5, 10.3), gp70_B.CaseA_V1_V2 (8.4, 4.7), gp70_B.CaseA2 V1/V2/169K (0.6, 0.25), RV305 week 2: AE.A244 V1V2 tags (17.3, 20.8), gp70_B.CaseA_V1_V2 (20.0, 9.5), gp70_B.CaseA2 V1/V2/169K (1.4, 1.4), RV305 week 26: AE.A244 V1V2 tags (13.4, 10.5), gp70_B.CaseA_V1_V2 (9.8, 6.8), gp70_B.CaseA2 V1/V2/169K (0.75, 0.40) (**S4 Table**). IgG V1V2 concentrations were not boosted compared to RV144 by the ALVAC-HIV only regimen, consistent with the expectation for protein (RV144) versus ALVAC (RV305) immunizations (**Figs 2D and S5 and S4 Table**). After protein boosting, a higher percentage of vaccinees had IgG gp70_B.CaseA_V1_V2 concentrations above the putative protective threshold associated with vaccine efficacy (2.98 μg/mL) compared to RV144 (63% positive responders at RV144 week 26 vs. 80% and 79% positive responders at RV305 weeks 2 and 26, respectively) [35] (**S5 Table**).

To quantify the breadth of binding to linear V2 hotspot, MB curves were plotted based on the percentages of V2 sequence variants each subject responded to at certain magnitude levels (**S6 Fig**). MB of the V2 hotspot binding response dropped for the AIDSVAX B/E only group and slightly for the ALVAC-HIV/AIDSVAX B/E group from post first boost to post second boost whereas the V2 hotspot response MB was maintained in the ALVAC-HIV only group and even showed a trend of slight increase from post first boost to post second boost.

### Delayed boosting with ALVAC-HIV/AIDSVAX B/E or AIDSVAX B/E increases breadth of Env-specific IgG response

**Fig 3:**
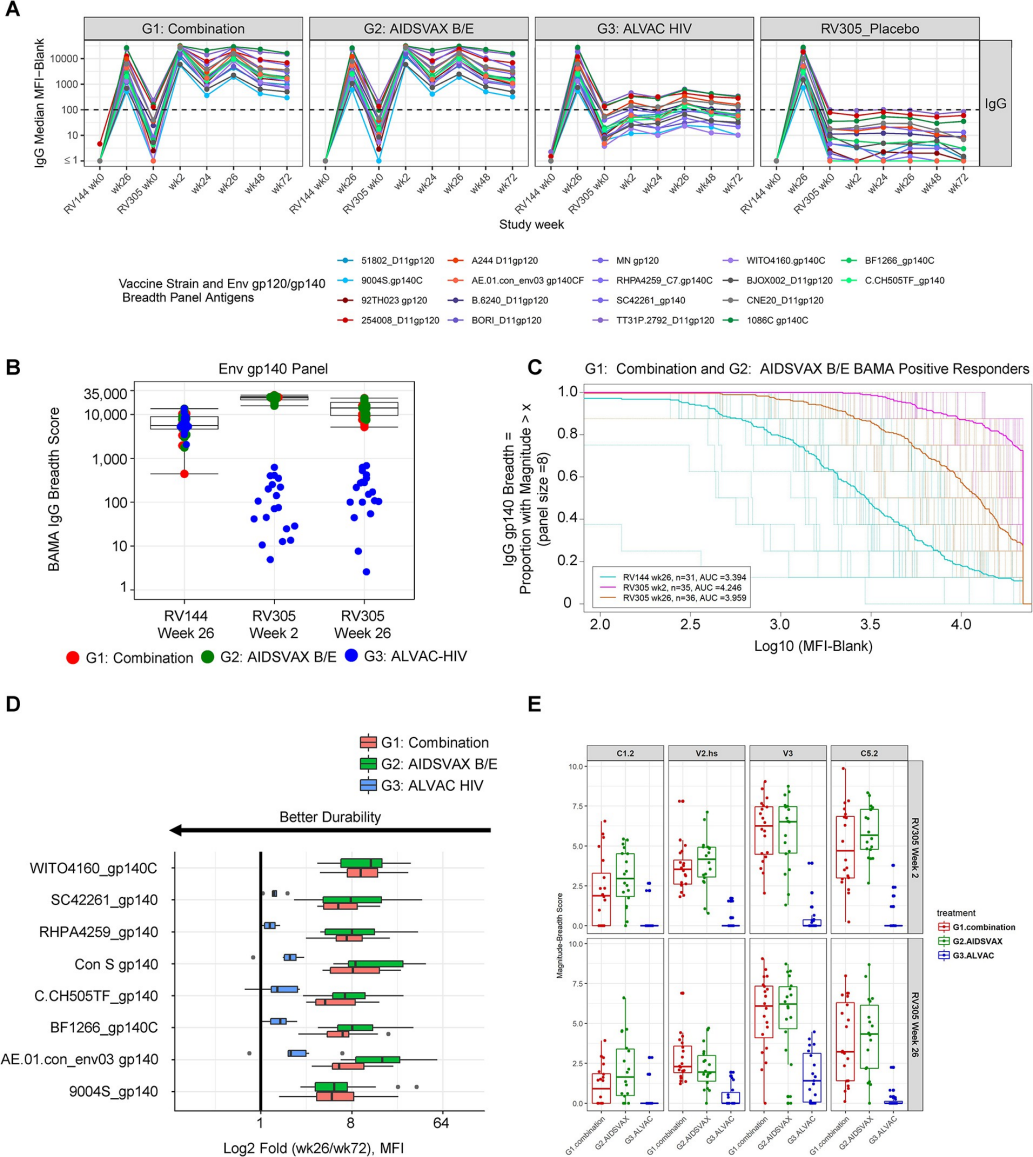
Delayed boosting with ALVAC-HIV/AIDSVAX B/E or AIDSVAX B/E increases breadth of Env-specific IgG response. (**A**) Kinetics of plasma IgG responses in RV305 vaccine recipients to vaccine strain (92TH023 gp120, A244 gp120, and MN gp120) and gp120 (n = 8) and gp140 (n = 8) Env breadth panel antigens representing genetic and geographic HIV-1 diversity. Group median BAMA MFI binding values among positive responders, color coded by HIV-1 subtype, are shown for two RV144 and 6 RV305 sampling time points; blue, clade A; red, CRF01_AE; purple, clade B; gray, CRF01_BC, green, clade C. (**B**) BAMA IgG breadth scores to the gp140 Env breadth panel at RV144 and RV305 post boost time points. Box and whisker plots show the median and interquartile ranges of scores across the Combination and AIDSVAX B/E only groups. Comparison of median breadth scores (aggregated for the Combination and AIDSVAX B/E only groups) across post RV144 boost (week 26) and RV305 boost time points (weeks 2 and 26) were performed using the two-sided Wilcoxon Signed Rank Test (Table 1). (**C**) Magnitude-breadth plot of IgG binding antibody responses to the gp140 breadth panel among Combination and AIDSVAX B/E only positive responders at 2 weeks post final RV144 vaccination (week 26) and 2 weeks post first and second RV305 boosts (weeks 2 and 26). Breadth is defined as the proportion of antigens in the 8 antigen gp140 breadth panel (y-axis) with log10 (MFI-Blank) greater than the threshold on the x-axis. Dashed lines display MB curves for each individual plasma sample measured at RV144 week 26 (turquoise), RV305 week 2 (pink), and RV305 week 26 (orange). Solid bold lines show the median MB among positive responders at each immunization time point. AUC values summarize the MB at a given time point across the entire range of MFI values. (**D**) Durability of Env gp140 IgG responses. Fold decline in IgG antibody binding magnitude to gp140 antigens from two weeks post last RV305 boost (week 26) to 12 months post last boost (week 72). Results are presented as log2 fold change, with the midline of the box plots indicating median and ends of the box plots indicating the 25th and 75th percentiles. The whiskers denote the minimum and maximum data points no more than 1.5 times the interquartile range (IQR). Data points that lie outside of the median ± 1.5 times the IQR are shown as black dots. Criteria for the fold (wk26/wk72) calculation: 1) response is positive at week 26, 2) MFI < 23000 at week 26, 3) MFI > 100 at week 72. Antigens with greater than or equal to 6 data points meeting this criteria for both the Combination (ALVAC-HIV/AIDSVAX B/E) and AIDSVAX B/E only groups are plotted for each vaccine boost regimen. Proximity of the bar to the y-axis indicates better durability. (**E**) Magnitude-breadth scores across C1.2, V2 hotspot (V2.hs), V3, and C5.2 linear epitopes in gp120 calculated as weighted means, using a hierchical clustering tree method (R package “mdw”) for binding to all strains with a positivity rate of >20% for any time point for each epitope. Box plots depict the median (midline) and 25th and 75th percentiles, with each symbol, color coded by group, indicating the epitope mapping breadth score for a single participant.

RV144 elicited antibodies capable of binding Env sequences of multiple HIV-1 subtypes [2,13,23], but the boostability of envelope binding breadth after delayed ALVAC-HIV and/or AIDSVAX B/E booster vaccination is not well understood. We used BAMA to analyze the breadth of IgG binding across the cohort of 70 vaccine recipients, employing well-characterized antigen panels consisting of 8 gp120 and 8 gp140 proteins from diverse clades and geographical regions (**Fig 3A**) [23]. Median gp120 and gp140 breadth scores were similar for the ALVAC-HIV/AIDSVAX B/E and AIDSVAX B/E only groups, with both being approximately 2.6–2.7-fold (against gp120) (**S7B Fig and S6 Table**) and 4.3–4.5-fold (against gp140) (**Fig 3B and S6 Table**) higher after the first boost compared to RV144 (**Table 1**, FDR *P* < 0.0001). Despite the slight reduction in Env breadth post second RV305 boost, breadth scores were approximately 1.6–1.8-fold and 2.4–2.6-fold greater than RV144 peak against gp120 and gp140 antigens, respectively (**Table 1**, *P* < 0.0001 for gp120 and gp140 breadth scores) (**Figs S7B and S3B**). Similar to what was observed for V1V2 IgG breadth, gp120 and gp140 breadth scores in the ALVAC-HIV only group were not elevated to RV144 peak level with boosting (**Figs S7B and 3B and S6 Table**). AUC for gp120 and gp140 MB curves increased from RV144 by approximately 1.2–1.3 fold post first RV305 boost and 1.1–1.2-fold post second boost among ALVAC-HIV/AIDSVAX B/E and AIDSVAX B/E only recipients (**Figs 3C and S7C**). The level of breadth achieved by high magnitude responders during RV305 exceeded that measured at RV144 peak (**Figs 3C and S7C**).

Analysis of the durability of IgG responses to envelope gp120 and gp140 sequences from 2 weeks post second boost (RV305 week 26) to 1 year post second boost (RV305 week 72) revealed a trend towards lower fold decline of responses to A244 gp120 (**S8 Fig**) and the consensus Envelope protein AE.01.con_Env03 gp140 (**Fig 3D**) in the ALVAC-HIV/AIDSVAX B/E group compared to the AIDSVAX B/E only group. Similar to the V1V2 IgG durability analysis, the ALVAC-HIV only group showed lower fold decline compared to both the other two groups for Env-binding IgG response (**Figs 3D and S8**).

IgG to C1.2, V3, and C5.2 linear epitopes showed a broad coverage of cross-clade Env sequences in the peptide array library, in contrast to V2, which was highly focused on AE.A244 and AE.TH023 of CRF01_AE, followed by C.1086 of clade C (**S1A and S1B Fig)**. Binding to AE.A244, AE.TH023 and C.1086 sequences accounted for 71% and 54% of linear binding to V2 hotspot for ALVAC-HIV/AIDSVAX B/E and AIDSVAX B/E only group, respectively, post first boost, and 78% and 76% of the response for these two groups, respectively, post second boost (**S1B Fig**).

To elucidate the breadth of antibodies elicited against linear epitope binding specificities, MB scores were calculated as the weighted mean of binding magnitudes across the different strains for each epitope. Following the first boost, ALVAC-HIV/AIDSVAX B/E and AIDSVAX B/E only recipients showed comparable C1.2, V2.hs, V3, and C5.2 MB scores that modestly decreased for C1.2, V2.hs, and C5.2 epitopes post second boost (**Fig 3E**). MB scores were lower for the ALVAC-HIV only group relative to the other groups, but breadth of binding elicited against V3 increased from post first boost to post second boost (**Fig 3E**).

### Delayed boost increases IgG1 gp120 and gp140 breadth

To determine the impact of delayed boosting on modifying IgG subclass levels, we analyzed antigen-specific binding antibody responses in longitudinal plasma samples by enzyme linked immunosorbent assay (ELISA) and BAMA. We used ELISA to screen the entire cohort of RV305 participants (n = 162) for IgG1-IgG4 against three proteins: the vaccine-matched subtype AE gp120 envelope protein A244gD and V1V2 scaffold protein antigens, gp70 V1V2 92TH023 (CRF01_AE) and gp70 V1V2 Case A2 (clade B), correlated with reduced HIV-1 risk in RV144 [2,21]. To evaluate the breadth of IgG subclass responses, we performed BAMA to measure IgG1-IgG4 to vaccine strain and Env and V1V2 breadth panel antigens in plasma from the subset of 70 participants. Primary analysis included comparison of BAMA total IgG, IgG1, IgG3, and IgG4 response magnitudes at RV305 peak versus RV144 peak and two weeks post final RV305 vaccination to address the prespecified hypothesis that boosting after a long interval with ALVAC + AIDSVAX B/E, compared to AIDSVAX B/E alone, confers enhanced binding antibody responses against four primary variables: 92TH023 gp120, A244 gp120, MN gp120 (vaccine strain envelopes), and AE.A244 V1V2 tags, an independent correlate of decreased HIV-1 risk in the RV144 vaccine efficacy trial [7,21].

**Fig 4:**
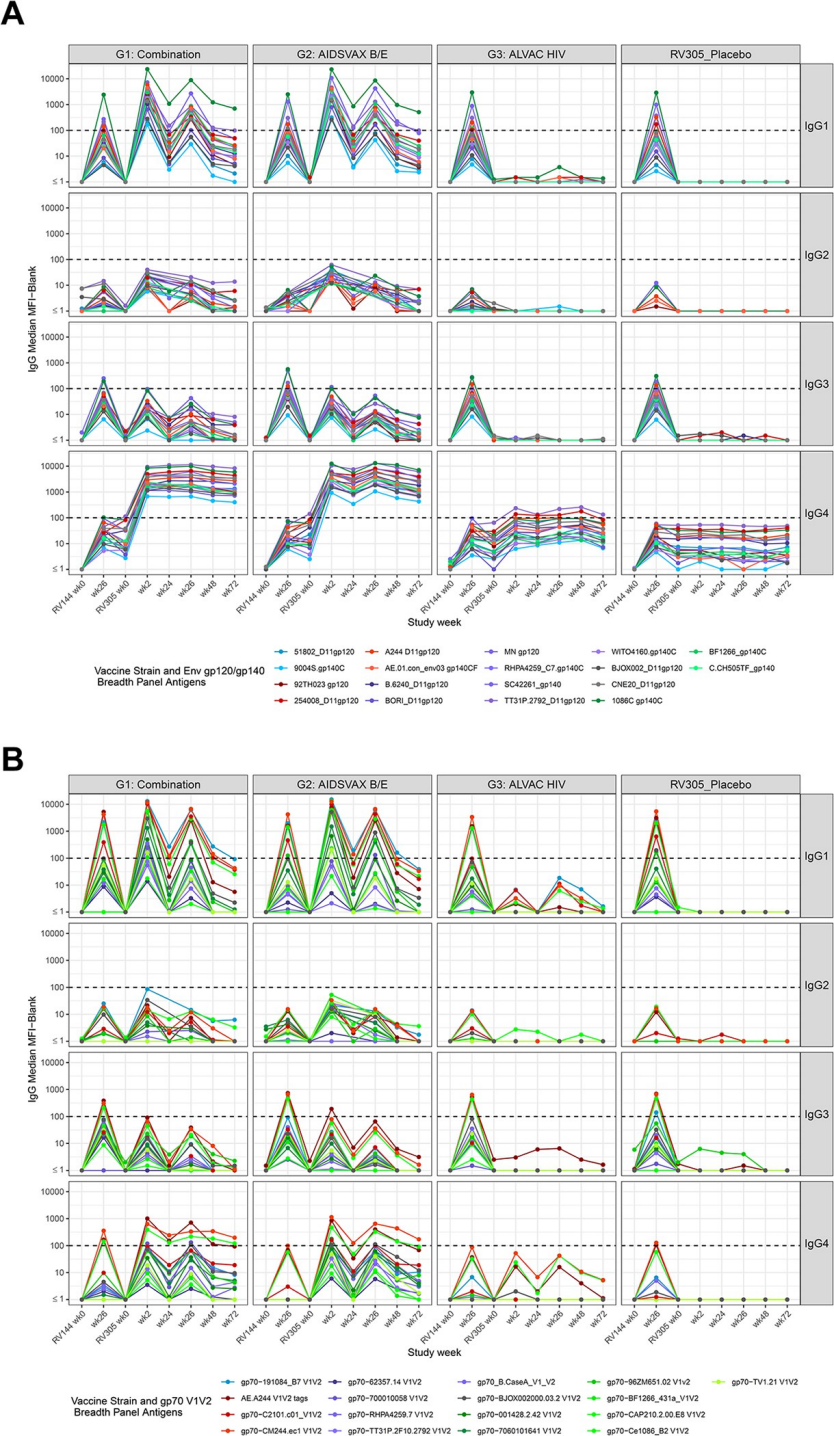
Binding antibody multiplex assay plasma IgG subclass (IgG1-IgG4) responses. Plasma from a subset of 70 RV305 recipients was tested for IgG1-IgG4 subclass binding to (**A**) vaccine strain immunogens (92TH023, A244 gp120, MN gp120), gp120 (n = 8) and gp140 (n = 8) Env breadth panel and (**B**) V1V2 (n = 16) breadth panel antigens and to AE.A244 V1V2 tags. Group median BAMA MFI is shown at each time point. Dotted black horizontal line, showing MFI equal to 100 indicates the minimum threshold for positivity for individual samples.

We first probed the kinetics of the vaccine-elicited IgG1 subclass response. Boosting with ALVAC-HIV/AIDSVAX B/E and AIDSVAX B/E alone elicited higher ELISA IgG1 binding antibody titers compared to RV144 peak (week 26); IgG1 peaked two weeks post first boost, with titers declining two weeks post second boost but persisting through week 72 at levels higher than RV144 week 48 levels (against gp120 A244 gD and gp70 V1V2 92TH023) (**S9A Fig**). BAMA revealed a similar pattern of development of IgG1 responses after late boosting (**Fig 4**). Primary analysis showed significantly higher HIV-1-specific total IgG and IgG1 at RV305 peak (week 2) compared to RV144 peak (week 26) to each vaccine strain envelope (92TH023 gp120, A244 gp120, MN gp120) and AE.A244 V1V2 tags (FDR *P* < 0.0001) (**Table 2 and S7A and S7D Fig**). HIV-1-specific total IgG and IgG1 magnitudes were significantly lower post second RV305 boost (week 26) compared to RV305 peak (week 2) (**Table 2**, FDR *P* < 0.0001) but significantly higher than RV144 peak for all antigens (**Table 2**, P < 0.0001) except for AE.A244 V1V2 tags (**Table 2,** *P* = 0.38 for IgG, *P* = 0.6 for IgG1; **S7A and S7D Fig**). Binding magnitudes did not differ significantly between ALVAC-HIV/AIDSVAX B/E and AIDSVAX B/E only groups at each time point tested (FDR *P* > 0.05) (**Tables 3, S1, and S7**). Plasma IgG1 antibodies from RV305 vaccinees were capable of binding multiple strains of HIV-1 Env and V1V2, resulting in enhancement of IgG1 breadth with ALVAC-HIV/AIDSVAX B/E and AIDSVAX B/E only delayed booster vaccination (**Figs 4 and S7E–S7G**).

**Table 2:** Primary and exploratory statistical analysis: comparison of binding antibody multiplex assay (BAMA) total IgG and IgG1 response magnitudes at RV144 week 26 versus RV305 week 2, RV305 week 2 versus RV305 week 26, and RV144 week 26 versus RV305 week 26.

			Primary Analysis Tier				Exploratory Analysis Tier
			Comparison: RV144_wk26—RV305_wk2		Comparison: RV305_wk2—RV305_wk26		Comparison: RV144_wk26—RV305_wk26
**Isotype**	**Variable**	**Antigen**	**Raw.*P***	**FDR.*P***	**Raw.*P***	**FDR.P**	**Raw.*P***
IgG	Magnitude^a^	92TH023 gp120 gDneg 293F mon	**<0.0001**	**<0.0001**	**<0.0001**	**<0.0001**	**<0.0001**
IgG	Magnitude^a^	A244 D11gp120_avi	**<0.0001**	**<0.0001**	**<0.0001**	**<0.0001**	**<0.0001**
IgG	Magnitude^a^	AE.A244 V1V2 tags	**<0.0001**	**<0.0001**	**<0.0001**	**<0.0001**	**0.38**
IgG	Magnitude^a^	MN gp120 gDneg/293F	**<0.0001**	**<0.0001**	**<0.0001**	**<0.0001**	**<0.0001**
IgG1	Magnitude^a^	92TH023 gp120 gDneg 293F mon	**<0.0001**	**<0.0001**	**<0.0001**	**<0.0001**	**<0.0001**
IgG1	Magnitude^a^	A244 D11gp120_avi	**<0.0001**	**<0.0001**	**<0.0001**	**<0.0001**	**<0.0001**
IgG1	Magnitude^a^	AE.A244 V1V2 tags	**<0.0001**	**<0.0001**	**<0.0001**	**<0.0001**	**0.6**
IgG1	Magnitude^a^	MN gp120 gDneg/293F	**<0.0001**	**<0.0001**	**<0.0001**	**<0.0001**	**<0.0001**

Data from participants in group 1 (received ALVAC-HIV + AIDSVAX B/E boost) and group 2 (received AIDSVAX B/E only boost) was combined to compare between two time points.

^a^Log transformed MFI values; response magnitudes compared using the Wilcoxon Signed Rank test.

*P* values less than <0.05 are shown in bold font.

FDR.*P* is the *P* value post correction for multiple comparisons using the Benjamini and Hochberg false discovery rate method.

**Table 3:** Primary statistical analysis: comparison of binding antibody multiplex assay (BAMA) total IgG and IgG1 response magnitudes between the Combination (ALVAC-HIV + AIDSVAX B/E) group (group 1) and AIDSVAX B/E only group (group 2) at RV305 week 2 and RV305 week 26.

			Comparison: Combination Group (Group 1) vs. AIDSVAX B/E Only Group (Group 2)			
			RV305 Week 2		RV305 Week 26	
Isotype	Variable	Antigen	Raw.*P*	FDR.*P*	Raw.*P*	FDR.*P*
IgG	Magnitude^a^	92TH023 gp120 gDneg 293F mon	0.46	0.68	0.65	0.83
IgG	Magnitude^a^	A244 D11gp120_avi	0.66	0.83	0.52	0.75
IgG	Magnitude^a^	AE.A244 V1V2 tags	0.16	0.29	1	1
IgG	Magnitude^a^	MN gp120 gDneg/293F	0.13	0.26	0.92	0.98
IgG1	Magnitude^a^	92TH023 gp120 gDneg 293F mon	0.97	1	0.76	0.92
IgG1	Magnitude^a^	A244 D11gp120_avi	0.53	0.76	0.57	0.77
IgG1	Magnitude^a^	AE.A244 V1V2 tags	0.99	1	0.90	0.98
IgG1	Magnitude^a^	MN gp120 gDneg/293F	0.62	0.82	0.34	0.53

^a^Log transformed MFI values; response magnitudes compared using the Wilcoxon Signed Rank test.

FDR.*P* is the *P* value post correction for multiple comparisons using the Benjamini and Hochberg false discovery rate method.

### Delayed boost lowers IgG3

**Fig 5:**
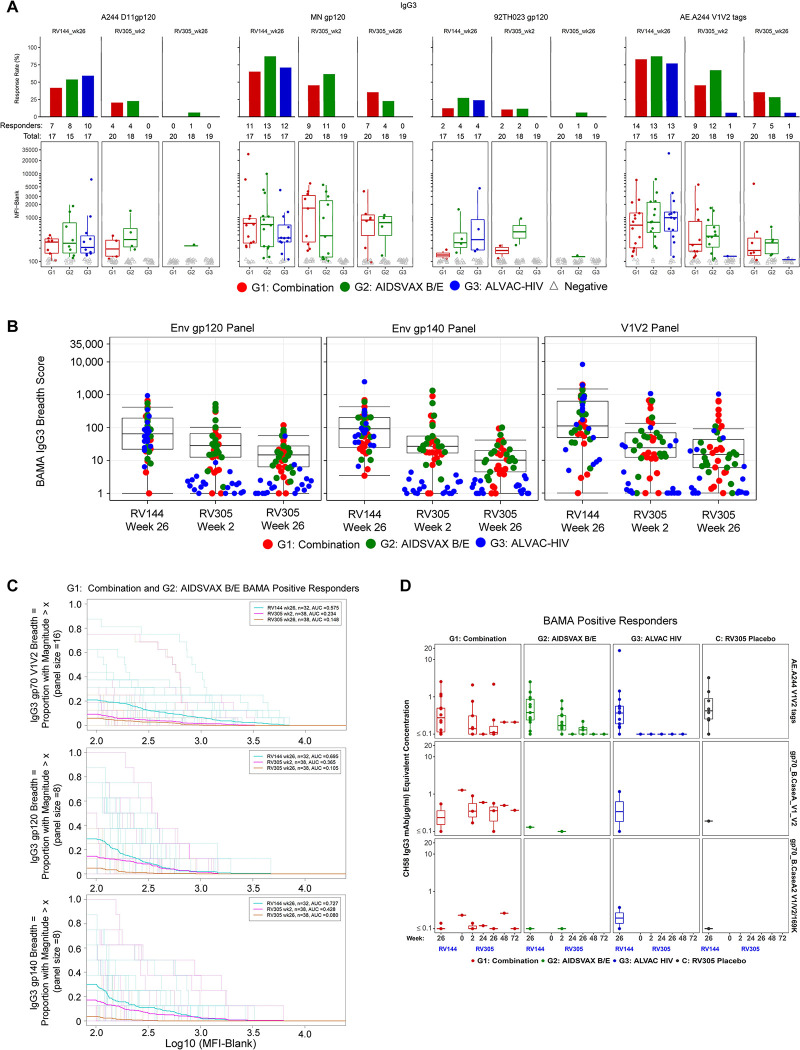
Delayed boost lowers IgG3. **(A)** Response rate (top panel) and response magnitude (bottom panel) for HIV-1 envelope-specific IgG3 in plasma at two weeks post final RV144 vaccination (week 26) and two weeks post first and second RV305 boosts (weeks 2 and 26, respectively) against four vaccine-matched antigens (A244 D11 gp120, MN gp120, 92TH023 gp120, and AE.A244 V1V2 tags) identified as primary immune variables. The number of positive responders is shown over the total number of individuals analyzed at each time point, represented as a bar graph displaying percent responders. Box plots depict the median (midline) and 25^th^ and 75^th^ percentiles, with the colored symbols indicating the BAMA IgG3 response magnitude for a single participant expressed as MFI. Open gray triangles depict negative responders. (**B**) IgG3 breadth scores, defined as the mean of IgG3 responses to Env gp120, gp140, and gp70 V1V2 breadth panel antigens (panel size = 8, 8, and 16 antigens, respectively), are shown for RV144 week 26 and RV305 weeks 2 and 26. Each symbol represents the breadth score for a single participant. Red, Combination (ALVAC-HIV + AIDSVAX B/E) group; green, AIDSVAX B/E only group; blue, ALVAC-HIV only group. Box plots show the distribution of breadth scores among Combination and AIDSVAX B/E only boost recipients, displaying the median (midline), 25^th^ and 75^th^ percentiles. (**C**) Magnitude breadth (MB) plots of IgG3 binding antibody responses to the gp70 V1V2 breadth panel (top plot), gp120 breadth panel (middle plot), and gp140 breadth panel (bottom plot) measured at RV144 week 26 (turquoise), RV305 week 2 (pink), and RV305 week 26 (orange) for positive responders that received ALVAC-HIV + AIDSVAX B/E or AIDSVAX B/E only boosts. The y-axes depicts breadth (number of antigens in a given panel with positive response, depicted as a percentage) at a given response magnitude (log 10 binding antibody MFI) across the x-axes. Sample-specific and group averaged MB curves are represented by dashed and solid lines, respectively. AUC values summarize the MB profile at a given time point across the entire range of binding values across the x-axis. (**D**) Concentration of IgG3 antibodies to three V1V2 antigens associated with decreased HIV-1 risk in RV144 plotted for BAMA positive responders by vaccine group across longitudinal RV144 and RV305 time points. V2-specific microgram per milliter (μg/ml) equivalent concentrations for each plasma sample were quantified by 4 parameter logistic (4PL) regression based on standard curve titration of the IgG3 V2-specific monoclonal antibody CH58 curves run in each BAMA. Each symbol represents the concentration for a single plasma sample. The midline of the box plot denotes the median concentration, and the ends of the box plot denote the 25^th^ and 75^th^ percentiles among positive responses.

Since Env- and V1V2-specific IgG3 was associated with lower infection risk in the RV144 trial [7] and was shown to decline soon after vaccination [7,14], we examined whether ALVAC and/or AIDSVAX B/E late boosting improved the level and breadth of IgG3 binding antibodies against HIV-1 gp120, gp140, and V1V2 envelope protein sequences. As determined by ELISA, RV305 protein boosts elicited IgG3 to gp120 A244 gD and gp70 V1V2 92TH023 that was lower than RV144 (week 26) after the first RV305 boost (lower for gp120 A244 gD) and the second boost, declining rapidly to baseline levels 6 months post second boost (**S9C Fig**). BAMA analysis against an array of proteins representing global HIV-1 diversity [23] demonstrated IgG3 kinetics similar to those measured by ELISA (**Figs 4 and 5**). Late boosting with ALVAC-HIV alone did not induce detectable ELISA or BAMA IgG3 responses above baseline in the majority of vaccinees, with a small proportion of ALVAC-HIV only recipients (≤10.5%) exhibiting BAMA IgG3 at post boost time points against gp70-Ce1086_B2 V1V2 (clade C), gp70-CM244.ec1 V1V2 (clade AE), and AE.A244 V1V2 tags (clade AE) (**Figs 4 and 5 and S8 Table**). Pairwise comparison of BAMA response magnitudes in ALVAC-HIV/AIDSVAX B/E and AIDSVAX B/E only groups at RV144 peak (week 26) versus RV305 boosts (week 2 and week 26) showed significantly lower IgG3 magnitudes to 92TH023 gp120, A244 gp120, MN gp120, and AE.A244 V1V2 tags after each RV305 boost (FDR *P* < 0.007) (**Fig 5 and Table 4**). Response magnitudes between ALVAC-HIV/AIDSVAX B/E and AIDSVAX B/E arms were not significantly different from each other against the four variables included in the primary analysis (FDR *P* > 0.05) (**Table 5 and Fig 4**).

**Table 4:** Primary statistical analysis: comparison of binding antibody multiplex assay (BAMA) IgG3 response magnitudes at RV144 week 26 versus RV305 week 2 and RV305 week 2 versus RV305 week 26.

			Comparison: RV144_wk26—RV305_wk2		Comparison: RV305_wk2—RV305_wk26	
Isotype	Variable	Antigen	Raw.*P*	FDR.*P*	Raw.*P*	FDR.*P*
IgG3	Magnitude^a^	92TH023 gp120 gDneg 293F mon	**0.0033**	**0.007**	**<0.0001**	**<0.0001**
IgG3	Magnitude^a^	A244 D11gp120_avi	**<0.0001**	**<0.0001**	**<0.0001**	**<0.0001**
IgG3	Magnitude^a^	AE.A244 V1V2 tags	**<0.0001**	**<0.0001**	**<0.0001**	**<0.0001**
IgG3	Magnitude^a^	MN gp120 gDneg/293F	**<0.0001**	**0.0001**	**<0.0001**	**<0.0001**

Data from participants in group 1 (received ALVAC-HIV + AIDSVAX B/E boost) and group 2 (received AIDSVAX B/E only boost) was combined to compare between two time points.

^a^Log transformed MFI values; response magnitudes compared using the Wilcoxon Signed Rank test.

*P* values less than <0.05 are shown in bold font.

FDR.*P* is the *P* value post correction for multiple comparisons using the Benjamini and Hochberg false discovery rate method.

**Table 5:** Primary statistical analysis: comparison of binding antibody multiplex assay (BAMA) IgG3 response magnitudes between the Combination (ALVAC-HIV + AIDSVAX B/E) group (group 1) and AIDSVAX B/E only group (group 2) at RV305 week 2 and RV305 week 26.

			Comparison: Combination Group (Group 1) vs. AIDSVAX B/E Only Group (Group 2)			
			RV305 Week 2		RV305 Week 26	
Isotype	Variable	Antigen	Raw.*P*	FDR.*P*	Raw.*P*	FDR.*P*
IgG3	Magnitude^a^	92TH023 gp120 gDneg 293F mon	0.66	0.83	0.18	0.31
IgG3	Magnitude^a^	A244 D11gp120_avi	0.33	0.52	0.25	0.42
IgG3	Magnitude^a^	AE.A244 V1V2 tags	0.14	0.26	0.29	0.48
IgG3	Magnitude^a^	MN gp120 gDneg/293F	0.76	0.92	0.83	0.95

^a^Log transformed MFI values; response magnitudes compared using the Wilcoxon Signed Rank test.

FDR.*P* is the *P* value post correction for multiple comparisons using the Benjamini and Hochberg false discovery rate method.

BAMA IgG3 response rates among ALVAC-HIV/AIDSVAX B/E and AIDSVAX B/E only recipients against vaccine-matched and breadth panel antigens ranged from 0% to 55% at RV305 peak, falling to 0–32% by two weeks post second RV305 vaccination and 0–8% by 1 year post second RV305 vaccination (**S8 Table**). IgG3 gp120, gp140, and V1V2 breadth scores in these groups significantly decreased with each RV305 boost (FDR *P* < 0.002 post first boost and *P* < 0.0004 post second boost, **Table 6**) and were significantly lower than breadth observed at RV144 peak immunogenicity (*P* < 0.0001, **Tables 6 and S9 and Fig 5B**). A comparison of AUC MB curves across the sampling time points showed progressively diminished IgG3 MB in ALVAC-HIV/AIDSVAX B/E and AIDSVAX B/E only groups after two RV305 boosts (**Fig 5C**). Remarkably, RV305 vaccinees with the highest V1V2 IgG3 binding profiles (BAMA MFIs top 2 ranked for each antigen) were mostly boosted with ALVAC-HIV/AIDSVAX B/E (**S10 Fig**), suggesting that ALVAC-HIV impacts the development of the vaccine-induced V1V2 IgG3 response.

**Table 6:** Primary and exploratory statistical analysis: comparison of binding antibody multiplex assay (BAMA) IgG3 breadth scores at RV144 week 26 versus RV305 week 2 (primary analysis), RV305 week 2 versus RV305 week 26, and RV144 week 26 versus RV305 week 26 (exploratory analysis).

			Primary Analysis Tier		Exploratory Analysis Tier	
			Comparison: Log breadth score at RV144_wk26—RV305_wk2		Comparison: Log breadth score at RV305_wk2—RV305_wk26	Comparison: Log breadth score at RV144_wk26—RV305_wk26
Isotype	Variable	Breadth Panel	Raw.*P*^a^	FDR.*P*^b^	Raw.*P*^a^	Raw.*P*^a^
IgG3	Magnitude	V1V2	**<0.0001**	**<0.0001**	**0.0004**	**<0.0001**
IgG3	Magnitude	gp120	**<0.0001**	**<0.0001**	**<0.0001**	0
IgG3	Magnitude	gp140	**0.0008**	**0.002**	**<0.0001**	0

Data from participants in group 1 (received ALVAC-HIV + AIDSVAX B/E boost) and group 2 (received AIDSVAX B/E only boost) was combined to compare between two time points.

Breadth scores were calculated as the mean of the log transformed MFIs of the individual antigens in a given panel.

^a^*P* values determined by Wilcoxon Signed Rank Test, with P values less than 0.05 shown in bold font.

^b^FDR.*P* is the *P* value post correction for multiple comparisons using the Benjamini and Hochberg false discovery rate method.

V1V2 IgG3 levels were too low, in most cases, to accurately quantify CH58 μg/mL equivalent concentrations. In cases where V1V2 IgG3 levels were quantifiable, the concentrations declined to undetectable levels by week 72 (**Fig 5D and S9 Table**). The fraction of RV305 recipients demonstrating positive Env-specific IgG3 binding at 2 weeks post second boost and 1 year post second boost was too low for calculation of median fold decline (requires at least 6 positive data points per group). However, for antigens with at least three positive responders in the ALVAC-HIV/AIDSVAX B/E and AIDSVAX B/E only groups at week 26 (n = 5 antigens), vaccine recipients boosted with ALVAC-HIV/AIDSVAX B/E exhibited lower IgG3 percent decline from week 26 to week 72 compared to AIDSVAX B/E only recipients against 4 of the 5 antigens, suggesting enhanced durability of IgG3 against 1086C gp140, gp70-CM244 V1V2, AE.A244 V1V2 tags, and gp70-Ce1086 V1V2 elicited by the ALVAC-HIV-containing late boost regimen (**S11 Fig**).

### IgG4 and IgA1 are boosted by RV305 vaccination

**Fig 6:**
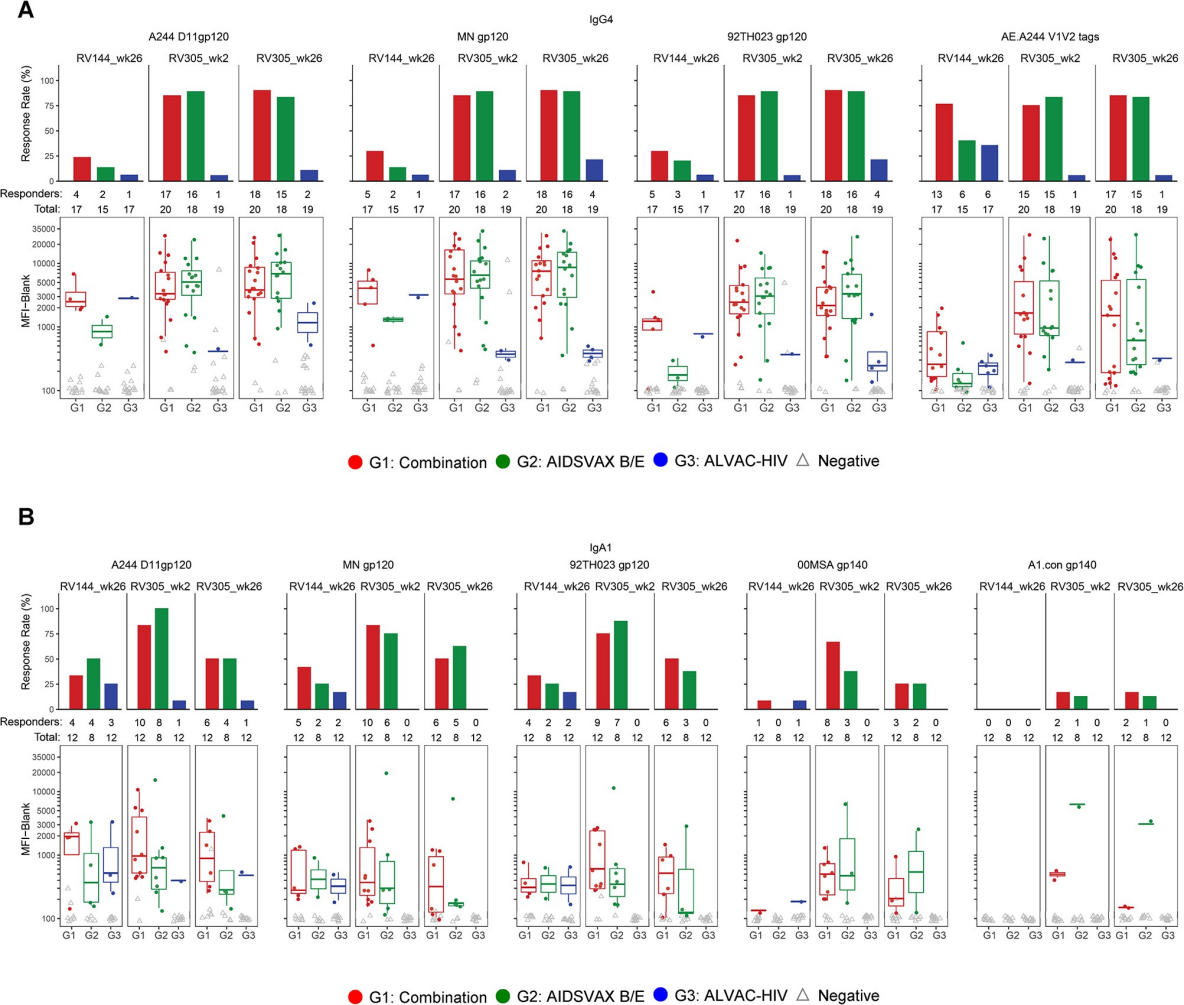
Delayed boost increases IgG4 and IgA1. (**A**) HIV-1-specific plasma IgG4 levels to vaccine-matched gp120 Envelope (A244 D11 gp120, MN gp120, 92TH023 gp120) and V1V2 (AE.A244 V1V2 tags) primary antigens determined by BAMA. Response rates (top panel) and binding magnitudes (bottom panel) are plotted for each group at two weeks post final RV144 vaccination (week 26) and two weeks post first and second RV305 boosts (weeks 2 and 26, respectively). Box plots (bottom panel) denote the median (midline) and interquartile ranges among positive responses. Solid dots depict positive responders, and open gray triangles depict non responders. (**B**) BAMA analysis of plasma IgA1 subclass levels in 32 randomly selected RV305 vaccinees (n = 12, 8, and 12 in groups 1, 2, and 3, respectively) against a panel of 5 Envelope proteins encompassing vaccine immunogens (A244 D11 gp120, MN gp120, 92TH023 gp120), 00MSA gp140, and clade A consensus (A.con.env03 gp140) proteins. Response rates (top panel) and binding magnitudes (bottom panel) are plotted for each group at two weeks post final RV144 vaccination (week 26) and two weeks post first and second RV305 boosts (weeks 2 and 26, respectively). Box plots (bottom panel) denote the median (midline) and interquartile ranges among positive responses. Solid dots depict positive responders, and open gray triangles depict non responders.

Among the IgG subclasses in humans, IgG2 and IgG4 exhibit reduced affinity for Fc receptors to mediate effector functions [36]. IgG2 and IgG4 subclass levels inversely correlated with functional activity in RV144 [14], thus we explored the impact of late boosting on circulating IgG2 and IgG4 levels in RV305 recipients as a surrogate for a less functional antibody response. IgG2 was not frequently elicited during RV305, with response magnitudes after boosting elevated slightly above baseline (**Figs S9B, S12A, and 4 and S11 Table**). ELISA IgG4 to gp120 A244 gD and gp70 scaffold antigens could not be detected in RV144, but late boosting with ALVAC-HIV/AIDSVAX B/E and AIDSVAX B/E alone elicited higher IgG4 binding antibody titers at two weeks post first and second boosts compared to RV144, with higher levels maintained up to 12 months post second boost (**S9D Fig**). Primary analysis demonstrated that IgG4 titers against vaccine-matched antigens did not statistically differ between the first and second RV305 boosts (FDR *P* > 0.05) (**Table 7 and Figs 6A and S9D**), with similar magnitudes observed between ALVAC-HIV/AIDSVAX B/E and AIDSVAX B/E only groups (FDR *P* >0.05) (**Tables 8 and S12**).

**Table 7:** Primary statistical analysis: comparison of binding antibody multiplex assay (BAMA) IgG4 response magnitudes at RV144 week 26 versus RV305 week 2 and RV305 week 2 versus RV305 week 26 by isotype.

			Comparison: RV144_wk26—RV305_wk2		Comparison: RV305_wk2—RV305_wk26	
Isotype	Variable	Antigen	Raw.*P*	FDR.*P*	Raw.*P*	FDR.*P*
IgG4	Magnitude	92TH023 gp120 gDneg 293F mon	**<0.0001**	**<0.0001**	0.4	0.61
IgG4	Magnitude	A244 D11gp120_avi	**<0.0001**	**<0.0001**	**0.04**	0.08
IgG4	Magnitude	AE.A244 V1V2 tags	**0.004**	**0.007**	0.32	0.52
IgG4	Magnitude	MN gp120 gDneg/293F	**<0.0001**	**<0.0001**	0.16	0.29

Data from participants in group 1 (received ALVAC-HIV + AIDSVAX B/E boost) and group 2 (received AIDSVAX B/E only boost) were combined to compare between two time points.

^a^Log transformed MFI values; response magnitudes compared using the Wilcoxon Signed Rank test.

*P* values less than <0.05 are shown in bold font.

FDR.*P* is the *P* value post correction for multiple comparisons using the Benjamini and Hochberg false discovery rate method.

**Table 8:** Primary statistical analysis: comparison of binding antibody multiplex assay (BAMA) IgG4 response magnitudes between the Combination (ALVAC-HIV + AIDSVAX B/E) group (group 1) and AIDSVAX B/E only group (group 2) at RV305 week 2 and RV305 week 26.

			Comparison: Combination Group (Group 1) vs. AIDSVAX B/E Only Group (Group 2)			
			RV305 Week 2		RV305 Week 26	
Isotype	Variable	Antigen	Raw.*P*	FDR.*P*	Raw.*P*	FDR.*P*
IgG4	Magnitude	92TH023 gp120 gDneg 293F mon	0.57	0.77	0.83	0.95
IgG4	Magnitude	A244 D11gp120_avi	0.54	0.77	0.8	0.95
IgG4	Magnitude	AE.A244 V1V2 tags	0.99	1	0.92	0.98
IgG4	Magnitude	MN gp120 gDneg/293F	0.87	0.98	0.9	0.98

*P* values less than <0.05 are shown in bold font.

FDR.*P* is the *P* value post correction for multiple comparisons using the Benjamini and Hochberg false discovery rate method

In the RV144 vaccine trial, plasma IgA to A.00MSA gp140 and A1 Con gp140 correlated with increased risk of HIV-1 infection (decreased vaccine efficacy) [2,4]. We previously showed that delayed boosting of RV144 recipients with ALVAC-HIV/AIDSVAX B/E or AIDSVAX B/E alone induced significantly higher total IgA binding antibody responses compared to RV144 [25]. To delineate the subclass composition of vaccine-elicited plasma IgA, we measured IgA1 and IgA2 responses to Envelope proteins by BAMA in serial plasma samples from 32 randomly selected RV305 participants. In the ALVAC-HIV/AIDSVAX B/E and AIDSXAX B/E only groups, IgA1 to vaccine strain antigens (A244 gp120, 92TH023 gp120, and MN gp120) and antigens associated with increased HIV-1 risk (IgA to 00MSA 4076 and A1 Con gp140) in RV144 increased two weeks post first boost and was not further boosted following the second boost (**Fig 6B and S13 Table**). Comparison of IgA responses between RV144 and RV305 peak time points (week 26 and week 2, respectively) revealed significantly higher magnitude of IgA1 responses at RV305 peak compared to RV144 peak for all antigens (**S14 Table**). IgA1 response rates decreased post second boost for all vaccine groups (**S13 Table**). Similar response rates and magnitudes were observed between ALVAC-HIV/AIDSVAX B/E and AIDSXAX B/E only groups at RV144 and RV305 peak immunogenicity visits (**S13 Table and Fig 6B**). Plasma IgA2 responses could not be detected at any of the RV144 or RV305 time points studied (**S12B Fig and S15 Table**).

### Antibody-dependent phagocytosis and virion capture are boosted by RV305 vaccination

**Fig 7:**
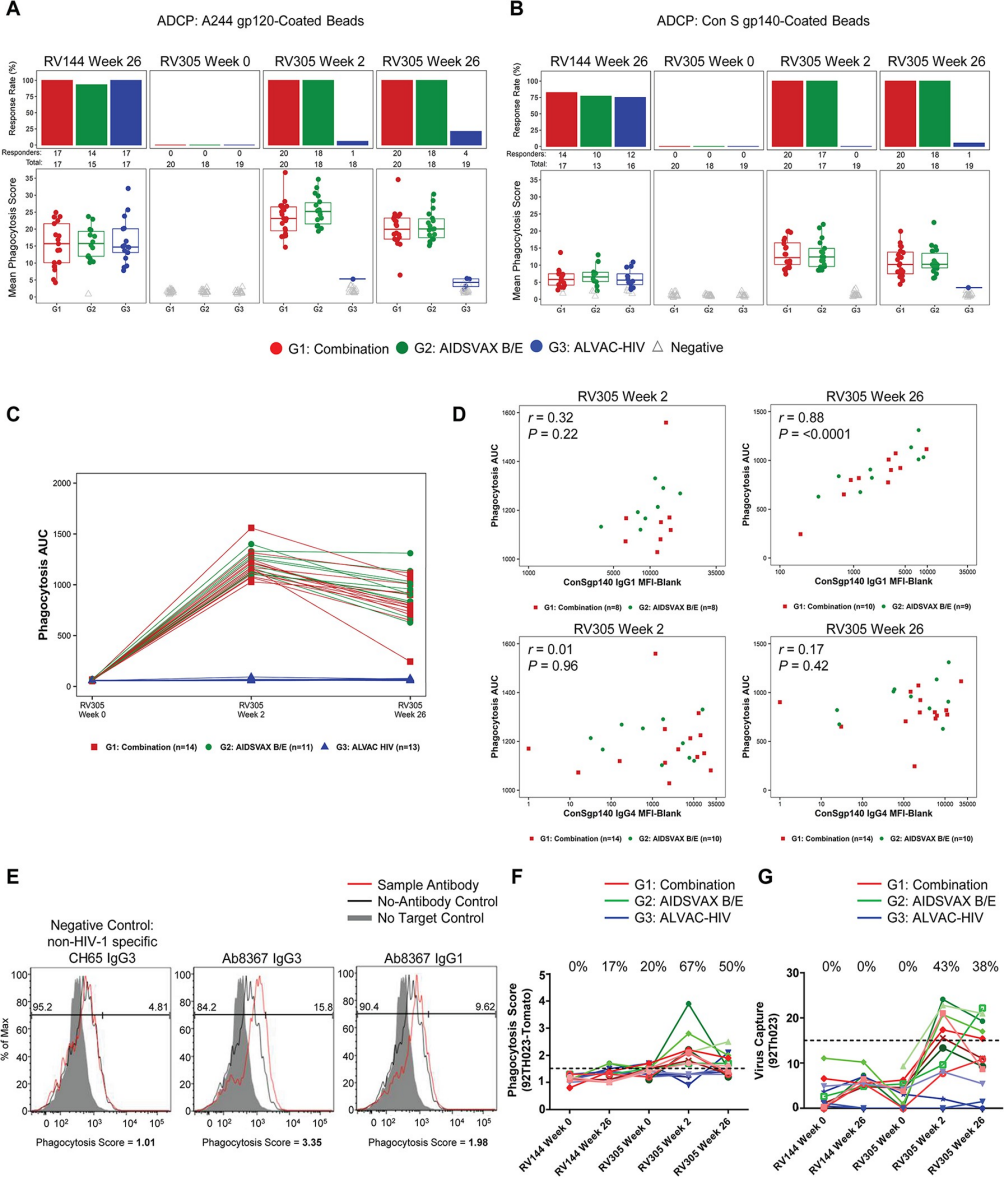
Antibody-dependent cellular phagocytosis (ADCP) is boosted by RV305 vaccination and is associated with IgG1 binding response. Plasma antibody-mediated uptake of (**A**) Con S gp140 or (**B**) A244 gp120-conjugated fluorescent beads in THP-1 cells was quantified by flow cytometry for vaccinees in the down-selected set of 70 RV305 participants. Top panels in **A** and **B** show the number of responders over the total number of participants analyzed whereas bottom panels denote the mean phagocytosis score per group at RV144 peak immunogenicity (week 26), RV305 baseline (week 0) and post boost time points (weeks 2 and 26). The box plots show the distribution of positive responses, with the mid-line denoting the median and the ends of the boxplot denoting the 25^th^ and 75^th^ percentiles. Data are representative of two independent experiments. (**C**) Phagocytosis AUC was calculated for a subset of 38 vaccinees (n = 14, 11, and 13 in groups 1, 2, and 3, respectively) whose plasma was tested for Con S gp140 ADCP. AUC for ADCP scores from a 5 point, 5-fold dose response curve from 50 μg/ml to 0.08 μg/ml is shown. (**D**) Spearman correlations between Con S gp140 phagocytosis AUC and IgG1 or IgG4 Con S gp140 BAMA MFI (aggregated for Combination and AIDSVAX B/E only groups) at RV305 week 2 and week 26; two-sided *P* values are shown for Spearman’s rho correlation coefficients. IgG2 and IgG3 binding levels were too low to perform correlation analysis. (**E**) Representative histograms of HIV-1_CM235_Tomato virion internalization by primary monocytes in the presence of Ab8367, a gp120-specific native IgG3 monoclonal antibody (mAb) isolated from a RV305 vaccinee by antigen-specific single memory B cell sorting. Ab8367 IgG1 and IgG3 recombinant mAbs mediated phagocytosis, showing greater potency when expressed as IgG3 (middle panel) compared to IgG1 (right panel). Red lines represent antibody-mediated internalization of virions; black lines represent internalization of virions in the absence of antibody. The shaded gray region represents the negative control in the absence of virus. The influenza hemagglutinin (HA)-specific broadly neutralizing antibody CH65 IgG3 was used as a negative control. Data are representative of two independent experiments. (**F**) Purified plasma IgG from 15 RV305 vaccine recipients (n = 5 in each group) was tested for antibody-mediated HIV-1_92TH023_-Tomato virion internalization in primary monocytes, analyzed across RV144 and RV305 baseline and immunogenicity time points by flow cytometry. Each solid line, color coded by vaccination group, represents one vaccinee. The horizontal dashed black line denotes the threshold for positivity. Response rates at each time point are shown at the top of the graph. (**G**) HIV-1_92TH023_ infectious virion capture was measured in 15 RV305 vaccinees (n = 5 in each group) using purified IgG from longitudinal plasma samples. The horizontal dashed black line represents the threshold for positivity. Each solid line represents one vaccinee. The percentage of vaccinees with vaccine-elicited antibodies capable of infectious virion capture is shown at the top of the graph for each time point.

To determine the impact of delayed and repetitive boosting on ADCP function, we examined plasma phagocytosis activity in 70 participants. ADCP of HIV-1 Env Con S gp140 and A244 gp120-conjugated fluorescent beads was examined in a human monocytic cell line (THP-1 cells) using a flow cytometry based method [37]. ALVAC-HIV/AIDSVAX B/E and AIDSVAX B/E only recipients displayed 1.3–1.6-fold higher ADCP activity after the first boost, compared to the peak RV144 response, that waned after the second boost (**Fig 7A and 7B**). Notably, the ALVAC-HIV only regimen elicited A244 gp120 ADCP in 6% and 21% of recipients after the first and second boosts, respectively (**Fig 7A**), coinciding with the development of other antibody responses in the ALVAC-HIV group following the second boost.

To develop a high resolution phagocytosis dataset, we generated Con S gp140 phagocytosis dose-response curves for 32 participants using various concentrations of IgG purified from plasma collected at RV305 baseline (week 0), after the first boost (week 2), and after the second boost (week 26) (**Fig 7C**). To identify the antibody epitopes/subclasses that mediate phagocytosis function, we used statistical models that predict virion and bead phagocytosis function from epitope and subclass-specific binding data. Con S gp140 bead phagocytosis AUC values for ALVAC-HIV/AIDSVAX B/E and AIDSVAX B/E only groups were aggregated and correlated with Con S gp140-specific IgG1, IgG2, IgG3, and IgG4 binding measured at each post boost time point. While Env-specific IgG ADCP did not significantly correlate with IgG1 at RV305 week 2 (Spearman *rho* = 0.32, *P* = 0.22), IgG1 levels at RV305 week 26 were highly associated with ADCP (Spearman *rho* = 0.88, *P* < 0.0001) (**Fig 7D**). In contrast, IgG4 showed no correlation with phagocytosis at either post boost time point (**Fig 7D**). Binding levels of antigen-specific IgG2 and IgG3 were too low to perform correlation analysis. Thus, IgG1 but not IgG4 is associated with phagocytosis function in RV305.

To determine the capacity of vaccine-elicited Env IgG1 and IgG3 to mediate HIV-1 virion phagocytosis, a native IgG3 gp120-specific monoclonal antibody (Ab8367) isolated from memory B cells of an RV305 vaccine recipient using antigen-specific single cell sorting was expressed as recombinant IgG1 and IgG3 and compared for phagocytosis activity in primary monocytes. Ab8367 IgG3 mediated greater internalization of HIV-1_CM235_-tdTomato virions than Ab8367 IgG1 (phagocytosis scores of 3.4 versus 2.0 for IgG3 and IgG1, respectively), in line with previous observations that IgG3 is more potent than IgG1 for ADCP [33,37–39] (**Fig 7E**). Although IgG3 concentrations are lower than IgG1, IgG3 antibodies elicited in RV305 are functional for phagocytosis and could contribute in some individuals to the ADCP response.

We next purified IgG from a subset of 15 vaccinees (5 participants from each arm) and measured virion internalization activity against HIV-1_92TH023_ in primary monocytes. In RV144, antibody-dependent virion phagocytosis was elicited at low to undetectable levels (17%) (**Fig 7F**). After the first RV305 boost, IgG from ALVAC-HIV/AIDSVAX B/E and AIDSVAX B/E only participants showed detectable phagocytosis, with potency declining after the second boost (**Fig 7F**).

We previously demonstrated that RV144 vaccination elicited antibodies that could bind infectious virions including the vaccine strains HIV-1_CM244_ and HIV-1_MN_ [13]. We found that in RV144, virion capture was not detectable against HIV-1_92TH023_ (**Fig 7G**). However, boosting in RV305 conferred detectable virion capture responses in 43% of vaccinees after the first immunization but did not increase after the second immunization. Responses were higher in ALVAC-HIV/AIDSVAX B/E and AIDSVAX B/E only groups compared to ALVAC-HIV only plasma IgG, which did not capture HIV-1_92TH023_ infectious virions.

### Antibody-dependent cellular cytotoxicity is boosted by RV305 vaccination

**Fig 8:**
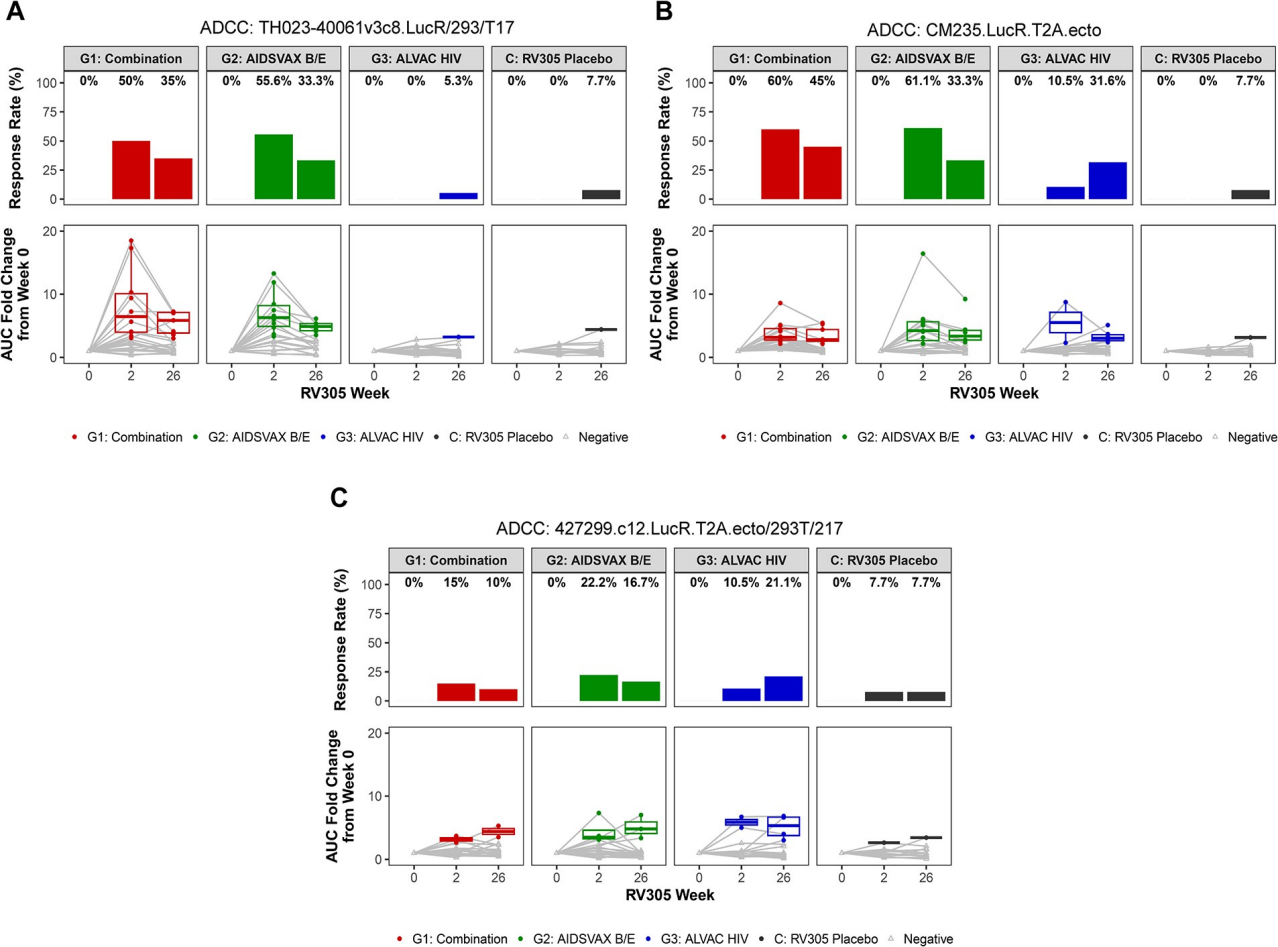
Antibody-dependent cellular cytotoxicity (ADCC) of HIV-1-infected target cells is boosted by RV305 vaccination. Plasma samples from 70 participants, drawn at RV305 study enrollment (week 0) and 2 weeks after the first and second RV305 boosts (weeks 2 and 26, respectively), were serially diluted and tested for ADCC-mediating antibody responses against (**A**) HIV-1 AE.TH023 (RV144 vaccine strain prime), (**B**) AE.CM235 (RV144 vaccine strain boost), and (**C**) AE.427299 (infected RV144 placebo strain)-infected CEM.NKR_CCR5_ target cells using the luciferase ADCC assay. Results are reported as AUC fold change from week 0 (bottom panel), and response rates are shown in the top panel. Box plots depict the distribution of AUC fold change values, with the midline denoting the median and ends of the box plot representing the interquartile range. Each dot represents one sample, with the gray lines connecting samples from the same donor. Solid dots indicate positive responders, and open gray triangles indicate non-responders.

To determine whether additional boosting improved the quality of ADCC responses, we measured the ability of plasma from 70 RV305 participants to mediate killing of target cells (CEM.NKR_CCR5_) infected with HIV-1 AE.TH023 (tier 1), AE.427299 (RV144 placebo breakthrough transmitted founder), and AE.CM235 (tier 2) infectious molecular clone (IMC) reporter viruses using a Luciferase-based ADCC assay [12,28,29,40,41]. Plasma obtained at RV305 baseline (week 0) and two weeks post first and second boosts (weeks 2 and 26, respectively) was tested at six five-fold serial dilutions (starting at 1:50), and AUC measurements were calculated to define ADCC activity. Magnitude of ADCC activity was evaluated both as AUC and as the AUC fold change over baseline values, which were calculated as the AUC of post boost sample divided by the AUC of matched week 0 samples. Positive calls were made for vaccine recipients based on AUC fold change over baseline values. Positivity cutoffs were calculated per antigen as the mean plus two standard deviations of AUC fold change over baseline values for all participants in the RV305 placebo group, with weeks 2 and 26 combined for this cutoff calculation. ADCC responses could be detected within each study arm, developing against the three strains in ALVAC-HIV/AIDSVAX B/E and AIDSVAX B/E only recipients and two of three strains in ALVAC-HIV only recipients (**Fig 8**). Frequency of responders was highest against AE.CM235, followed by AE.92TH023 and AE.427299 post first and second boosts (**Fig 8**). In the ALVAC-HIV/AIDSVAX B/E and AIDSVAX B/E only groups, ADCC activity peaked at week 2 and declined post second boost (AUC fold change over baseline between RV305 weeks 2 and 26: *P* < 0.0001 for AE.CM235, *P* = 0.0002 AE.TH023, *P* = 0.38 for AE.427299). ALVAC-HIV/AIDSVAX B/E and AIDSVAX B/E only groups displayed higher post boost AUC values compared to the ALVAC-HIV only group against AE.CM235 and AE.TH023 (**S13 Fig**). Consistent with the kinetics of ADCP responses, ADCC response rates against AE.CM235 and AE.427299 increased in the ALVAC-HIV only group from post first boost (10.5% against both viruses) to post second boost (31.6% and 21.1%, respectively) (**Fig 8B and 8C**) exceeding rates observed in placebo recipients (<7.7%).

### Distinct immunogenicity profiles elicited by ALVAC-HIV only and AIDSVAX B/E-containing booster regimens

**Fig 9:**
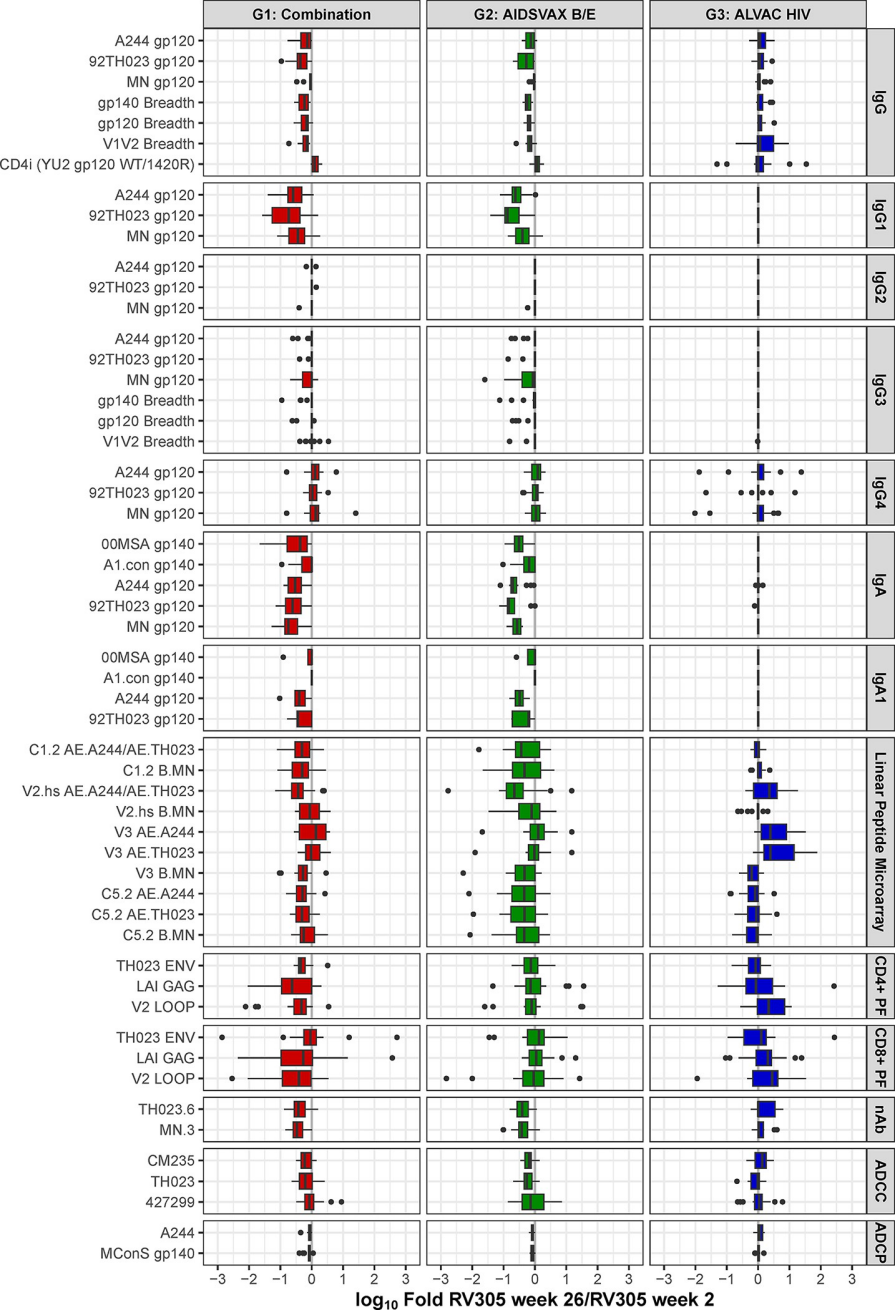
Fold change in humoral and cellular response magnitudes from RV305 week 2 to week 26. The fold change in magnitude of humoral and cellular immune measurements (n = 54) from 2 weeks post first boost (week 2) to 2 weeks post second boost (week 26) was calculated for each participant (n = 70) and then log 10 transformed. Measurements were grouped by generalized features (BAMA IgG, IgG1, IgG2, IgG3, IgG4, IgA, IgA1, linear peptide microarray binding, CD4 and CD8 T cell polyfunctionality, neutralizing antibody (nAb), ADCC, or ADCP effector functions) indicated by the gray bars on the right. The boxplots span the 25^th^ percentile to the 75^th^ percentile, with a black bar at the median. Whiskers extend from the greater of the minimum data point and median—1.5 times the interquartile range (IQR) to the lesser of the maximum data point and median + 1.5 times the IQR, where IQR = 75^th^-25^th^ percentile. Data points that lie outside of the median ± 1.5 times the IQR are shown as black dots. Horizontal bar pointing to the left of the x = 0 line indicates a higher response magnitude measured at RV305 week 2 compared to RV305 week 26; horizontal bar pointing to the right of the x = 0 line indicates a higher response magnitude measured at RV305 week 26 compared to RV305 week 2.

To further probe whether ALVAC-HIV/AIDSVAX B/E, AIDSVAX B/E only, or ALVAC-HIV only regimens boosted different antibody specificities and functions with similar dynamics, we plotted log 10 fold change between peak from the two immunizations for response magnitudes for 54 antigen-specific humoral and cellular features analyzed based on the cohort of 70 participants: Env IgG, IgG1, IgG2, IgG3, IgG4, and IgA1 binding, Env gp120, gp140, and V1V2 IgG and IgG3 breadth, CD4i IgG, binding to C1.2, V2 hotspot V3, and C5.2 linear epitopes of vaccine-matched strains, neutralization, ADCP, ADCC, and previously reported IgA magnitudes and Gag- and Env-specific CD4 and CD8 T cell polyfunctionality scores [25] (**Fig 9**). Similar boosting effects (similar week 26/ week 2 median fold change values) were observed for ALVAC-HIV/AIDSVAX B/E and AIDSVAX B/E only groups for these 54 antibody features, with an overall contraction of responses from post first boost to post second boost (negative median log 10 fold change values). The ALVAC-HIV only group, on the other hand, did not show similar contraction of response from post first to post second boost for IgG, IgA, and IgG and IgA subclasses responses as did the other two groups, and further, showed continued boosting (positive median log 10 fold change values) of the binding response to V2.hs and V3 linear epitopes, V2-specific CD4 and CD8 T cell polyfunctionality, and tier 1 nAb (TH023.6). The range of log 10 fold change values across the different variables among RV305 vaccine recipients indicated modest heterogeneity of responses within groups.

**Fig 10:**
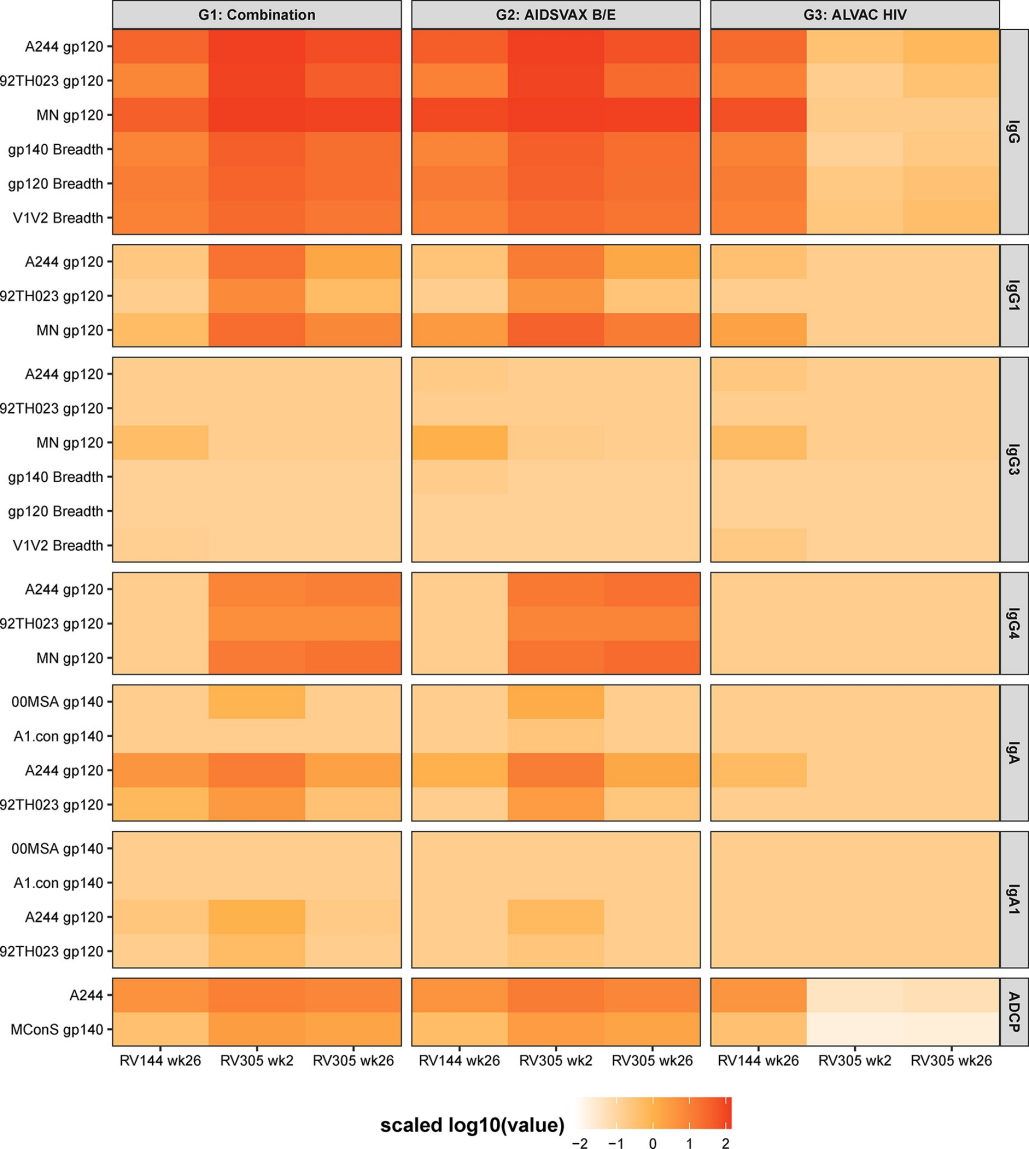
Addition of a boost improved anti-viral immunity associated with correlates of HIV-1 risk. Heatmap of 28 immune response variables longitudinally analyzed at RV144 peak immunogenicity (week 26) and two weeks post first and second RV305 boosts (weeks 2 and 26, respectively). All data was log10-transformed and then each variable [measurement type (BAMA MFI, BAMA breadth score, and ADCP score)] was scaled across participant, visit, antigen, and in the case of BAMA isotype/subclass for vaccinees in the down-selected set of 70 RV305 participants. The median of the scaled values are plotted by visit and treatment group, ordered by assay type [BAMA (IgG, IgG1, IgG3, IgG4, IgA, IgA1), and antigen-conjugated bead antibody-dependent phagocytosis (ADCP)]; scaling precludes comparison of variables with each other, but variables can be compared across groups and visits. Columns designate immunogenicity time points whereas rows represent immune measurements. Color intensity is directly proportional to response magnitude, with the darker colors indicating higher magnitudes and the lighter colors indicating lower magnitudes.

We then log 10 transformed and scaled each variable, plotting the median scaled values by visit and treatment group as a heatmap to enable quantitative comparison of variables across RV144 week 26, RV305 week 2, and RV305 week 26 immunogenicity time points for immune response (rows) (**Fig 10**) on a same relative scale across assays. Linear peptide microarray, T cell, nAb, virion capture, virion internalization, and ADCC measurements at RV144 week 26 were not included in the heatmap, as RV144 samples for these assays were not analyzed at the same time as the corresponding RV305 samples and therefore could not be compared with RV305 measurements. The overall architecture of immune responses to vaccine regimens containing AIDSVAX B/E was characterized by similarly elevated IgG (binding/breadth), IgG1, IgG4, and ADCP after two boosts compared to RV144.

## Discussion

Developing strategies to increase the magnitude and durability of protective immunity is critically needed in order to improve upon the modest efficacy of the one partially efficacious HIV vaccine regimen to date. The hypothesis tested in this study was that protective immunity can be boosted to higher magnitudes with improved durability following additional booster immunizations. We evaluated the specificity and quality of antibody responses in the follow-up immunization study of HIV-1-uninfected RV144 recipients after 6–8 years and found that antibody correlates of HIV-1 infection risk were substantially modulated by the delayed booster.

We demonstrated that the first booster immunization of the combination (ALVAC/protein) and protein-only boosts elicited (i) higher HIV-1-specific IgG V1V2 breadth and concentrations and (ii) higher ADCP and ADCC than the peak immunogenicity time point in the RV144 vaccine efficacy trial. Specifically, the median V1V2 IgG concentration was boosted to 9.5–20 μg/mL, which is greater than the median peak concentration measured in RV144 associated with a decrease in HIV-1 infection (at least 2.98 μg/mL gp70.B (CaseA2)-V1V2) specific IgG [35]) enabling 80% of vaccinees to achieve this level in RV305 compared to 63% in RV144. The level of V1V2-specific antibodies binding to multiple HIV-1 subtypes was identified as an inverse correlate of HIV-1 risk in RV144 [21]. We show that IgG breadth scores as well as overall AUC magnitude-breadth to V1V2 were boosted higher than RV144 peak levels after revaccination in RV305. Results from multiple preclinical studies are consistent with a role for V2-specific antibodies in protection and control of simian immunodeficiency virus (SIV) or simian human immunodeficiency virus (SHIV) infection [42–48]. Our findings underscore the important concept that late boosting improves cross-clade recognition of strains not included in the vaccine. Thus, increased breadth and concentrations of V1V2 antibodies may increase vaccine efficacy and potentially protect against genetically diverse strains.

Easterhoff *et al*. [27] reported that the delayed boost expanded the CD4bs reactive memory B cells. Interestingly, mAbs generated from memory B cells with long third heavy chain complementarity determining regions (HCDR3) neutralized a difficult-to-neutralize primary isolate suggesting that the boost provided improved antibody function. Our study did not evaluate CD4bs blocking antibodies, but we did evaluate antibody responses to the CD4 induced epitope (CD4i). This specificity was substantially elevated in the majority of the vaccinees after the boost and there was evidence for contributions from both the protein and ALVAC immunogens. CD4i-specific antibody specificities are thought to contribute to the ability to mediate antibody effector recognition and killing of infected cells [49]. A vaccine regimen that can induce CD4i-specific antibodies, in addition to a higher magnitude and breadth of V2-specific antibodies, is hypothesized to have improved antibody effector recognition of multiple virus targets. V2-specific memory B cell responses were evaluated in eight vaccine recipients and there were persistent V2 Env memory B cell clonal lineages with maturation of V2-specific epitope recognition and antibody effector functions [28]. The combination of the circulating antibody data in this report and the characterization of monoclonal antibodies from memory B cells demonstrate that long-lived V1V2 specific memory B cells were boosted by the delayed immunization strategy. An important follow-up trial, the RV306 clinical trial (Clinical Trials.gov Identifier: NCT01931358), assessed the impact of 12, 15, and 18 month boost intervals following the RV144 primary vaccination series [50] and reported that V1V2 IgG was optimally boosted with a longer delay between the prime and boost. These results suggest a way forward for inducing durable protective immunity.

We previously reported that vaccine-elicited Env V1V2 IgG3 correlated with decreased HIV-1 risk in RV144 [7], and in the HVTN 505 efficacy trial (DNA prime/rAd5 boost), Env IgG3 binding breadth also correlated with reduced risk of infection [51]. HIV-1-specific antibodies of the IgG3 subclass have been shown to mediate superior neutralization, ADCP [33,37], and antibody-dependent trogocytosis (ADCT)] [38,39]. This increased functionality is mainly related to higher affinities for Fc gamma receptor 2A (FcγR2A) due to the increased hinge length relative to other antibody subclasses such as IgG1 [38,39]. We currently hypothesize that the induction and maintenance of functional IgG3 is a critical goal for HIV-1 vaccine development. We found that late boosting after 6–8 years re-elicited Env- and V1V2-specific IgG3 in RV305 vaccine recipients that was of lower magnitude and breadth than that measured at RV144 peak, declining to undetectable levels after an additional boost administered after a 6 month interval, consistent with recent reports by Fishinger et al. [17] for this same vaccine regimen. Previous studies have also documented that repeated protein boosting dampened the IgG3 response [7,23,52,53]. Developing immunogen and adjuvant designs towards promoting durability of IgG3 and/or IgG1 anti-viral effector functions is critically needed, while preserving physiological Ig class-switching. Further work to profile B cells of vaccine recipients with differential IgG3 durability can define markers associated with B cell activation, survival, and proliferation that may provide insights into understanding the factors that influence the decay of IgG3 and how this potentially protective response can be programmed for persistence.

In contrast to the lack of boosting in the IgG3 response rate and magnitude post boost, Env IgG4 and IgA1 did increase. These effects could be linked to repeated antigen exposure known to skew responses towards the IgG4 subclass [7,14,36,52,53]. Our results on IgG4 are consistent with a previous report [17]. We previously reported that the delayed protein boost in the RV305 trial elevated IgA levels [25], and here we show that IgA1 levels are increased with additional boosting. Since both HIV-specific IgG4 and particular specificities of IgA were associated with blocking some, but not all, effector function [4,14,16], one hypothesis is that an HIV-1 vaccine may need to precisely tune the concentrations and specificities of antibody responses to optimize efficacy. Results from the RV306 clinical trial indicated that boosting after a 12 month interval did not increase plasma IgA levels to antigens identified as correlates of risk, suggesting that booster vaccination at this time point may be an option [50].

In our study, we found that RV305 elicited the antibody Fc effector functions of ADCP and ADCC. The ability of delayed boosts to elicit Fc-mediated antibody functions is notable because ADCP and ADCC were associated with delayed SIV [54] or SHIV [55–57] acquisition or viremia control after infection [58,59] in nonhuman primate studies. Similarly, ADCC correlated with decreased risk of infection in subjects with low anti-Env IgA response in RV144 [2,4], and breastmilk ADCC activity was associated with reduced risk of transmission in women with high viral loads [60]. ADCP and FcR binding correlated with decreased HIV-1 risk in HVTN 505 [51]. Notably, a B cell signature that correlated with ADCP was associated with correlates of protection in preclinical studies with different vaccine regimens [61], supporting the notion that ADCP is an important effector function to elicit for vaccine efficacy.

Our study revealed additional unique aspects of the immune responses elicited in RV305 that increased our previous understanding of the impact of delayed boosts on vaccine induced antibody responses [17]. In fact, the correlation between IgG1 binding magnitude and ADCP activity shown here suggests that even though IgG3 on a per antibody basis has improved function compared to IgG1 [37,38], ADCP activity observed in RV305 was predominantly mediated by the higher concentrations of vaccine-elicited IgG1. Moreover, our findings that ADCP and ADCC increased after the second ALVAC-HIV only boost, although of low magnitude, further supports the contribution of ALVAC-HIV to Fc-mediated functional antibody responses.

Several past and ongoing HIV-1 clinical trials, utilizing various immunogens, have evaluated late boost strategies to enhance pre-existing vaccine-induced responses and revealed different outcomes. Modified vaccinia virus ankara-B boosts (Clinical Trials.gov Identifier: NCT01923610) revealed enhanced cellular and humoral immunogenicity after the late boost [62]. However, the HIVIS06 trial (Clinical Trials.gov Identifier: NCT01461447) indicated that the third HIV-MVA boost did not improve the magnitude of responses observed after the second HIV-MVA immunization [63,64]. Spearman *et al*. [65] reported on antibody responses, which did not include anti-V2 responses, after a heterologous boost given up to 17 years after the prime immunization. In addition to the detection of baseline antibody responses, antibody titers were rapidly boosted indicating the presence of long-lived memory B cells that could be recalled quickly. Lastly, the Uhambo/HVTN 702 phase 2b/3 trial, a RV144 follow-up study that evaluated the efficacy of an adapted clade C ALVAC-HIV/gp120 vaccine regimen in South Africa, delivered with a different adjuvant (MF59) than the one used in RV144 (alum), included additional late boosts [66]. Despite the lack of efficacy [67], an immune correlates analysis revealed a correlation of V1V2 IgG with CD4 T cells and decreased HIV-1 risk in Uhambo/HVTN 702 [68]. This same vaccine regimen [69] had higher V1V2 breadth and ADCP responses after booster immunization, consistent with the results in this study for RV305. Notably, there was an initial slower decay of the V1V2 antibody and ADCP response after the booster immunization, suggesting the boost also improves the persistence of the antibody response. Data from these trials suggest that late boosting may increase immune responses, although this may not occur optimally with all regimens.

Remarkably, in this study, we demonstrated that inclusion of ALVAC in the boost immunization, either in combination with protein or alone, better maintained V2 response magnitude and breadth compared to the gp120 protein only group. Median fold decline of V2-specific IgG from two weeks post second boost to the 1 year durability time point was highest for the AIDSVAX B/E only group, followed the Combination group, and then the ALVAC-HIV only group. Percent decline of V2 IgG3 magnitude post second boost trended higher for the AIDSVAX B/E only group relative to the ALVAC-HIV/AIDSVAX B/E group. These results suggest a role for ALVAC-HIV vector in contributing to the durability of V2-specific IgG and IgG3, consistent with the findings of Felber *et al*. [70] that demonstrated improved magnitude and antibody durability with co-administration of vector and protein immunization in non-human primates [71]. The ALVAC vector immunization may impact adaptive immune responses and the protective efficacy of vaccination through its innate immune stimulatory properties. ALVAC can infect DCs [72] and induce antiviral responses [73,74] through production of antiviral genes and inflammatory cytokines. ALVAC primes and activates the inflammasome pathway and was shown to stimulate the cGAS/IFI16-STING-type I IFN pathway to prime AIM2 (an innate sensor for ALVAC) [75]. A transcriptomic analysis of RV144 vaccinees reported that type 1 interferons activating the IRF7 antiviral transcriptomic program was associated with decreased HIV-1 risk [76] and that the IFN γ signalling pathway significantly correlated with the frequency of CD4+ T cells and the V1V2 IgG. The preferential boosting and maintenance of certain epitope specificities alone or in combination with the gp120 protein boost may be due to sequence similarities in the prime and boost sequences [24].

A limitation of the current study is the lack of concurrent testing of RV144 peak immunogenicity samples with RV305 samples for analysis of linear epitope specificity and ADCC responses. However, analysis of RV305 post boost time points revealed restored responses to antigenic regions associated with decreased HIV-1 risk in RV144 (V2 hotspot, V3) [5] that were boosted to higher levels than RV305 baseline. Whether the magnitude and frequency of linear responses after the prolonged rest period and subsequent boosts were higher than RV144 cannot be discerned from this analysis. The presence of circulating ADCC antibodies against HIV-1-infected cells, infected with difficult to neutralize viruses, during RV305 that were not detected during RV144 may be due to differences in maturation of antibody responses as reported by Easterhoff et al. [27–29]. In fact, the isolation of a larger number of monoclonal antibodies capable of mediating ADCC in RV305 compared to RV144 [27–29], suggests that the boost increased the frequency of ADCC responses resulting in improved functional quality in eliminating HIV-1 infected cells.

Results from preclinical [77,78] and clinical efficacy studies [2,9,51,79,80] support key roles for combined cellular and humoral immunity for vaccine-elicited protection. We examined the relationship of antibody and cellular responses elicited in RV305 and found evidence of coordinated humoral and cellular responses. Both antibody responses (low magnitude V2 hotspot- and V3-directed linear antibodies, tier 1 nAb (TH023.6), and ADCP) and cellular responses (V2-specific CD4 and CD8 T cell polyfunctionality) were boosted by the second ALVAC booster immunization in contrast to the contraction observed when protein was included in the boosting regimen, consistent with a coordinated cellular and humoral response. Moreover, the high frequency of T follicular helper cells, associated with high affinity antibody production and affinity maturation, in the RV144 regimen [81] demonstrate the interconnected role of cellular and humoral immunity with this vaccine regimen.

The late booster immunizations in RV305 changed the quality and magnitude of immunity present after each immunization. Although the protein boost elevates more vaccinees above the putative protective antibody threshold, the overall proportions of antibody isotypes and antiviral functions are skewed. The optimal match of sequences in the prime and boost along with the use of the viral vector can drive antibody specificity and function, indicating that future vaccine immunogens can utilize prime-boost regimens to focus the immune response. These findings inform current vaccine immunogen designs aimed at improving breadth and population coverage of future HIV-1 vaccines.

## Materials and methods

### Ethics statement

The RV305 clinical trial (ClinicalTrials.gov number, NCT01435135) [25] was conducted in accordance with the Declaration of Helsinki and with approval granted by the Institutional Review Boards of Walter Reed Army Institute of Research, the Thai Ministry of Public Health, the Royal Thai Army Medical Department, Faculty of Tropical Medicine, Mahidol University, Chulalongkorn University Faculty of Medicine, and Siriraj Hospital. Written informed consent was obtained from clinical trial participants. Plasma samples were provided by the U.S. Military HIV Research Program (MHRP), and the institutional review board of Duke University Health System approved human specimen handling.

### Clinical trial participants

The RV305 clinical trial (ClinicalTrials.gov number, NCT01435135) [25] is a randomized, double-blind placebo-controlled trial designed to evaluate late boost strategies in 162 HIV-1-uninfected RV144 vaccine recipients who were at low risk for HIV-1 infection and completed the full RV144 immunization series (ClinicalTrials.gov number, NC00223080) [1]. RV305 study participants received one of three boost regimens or placebo (45:9 vaccine to placebo ratio) six to eight years after the conclusion of RV144, consisting of two doses of either: (1) ALVAC-HIV (vCP1521) (Sanofi Pasteur) [a recombinant canarypox virus vector engineered to express HIV-1 subtype B *gag* and *protease* (LAI strain) and subtype E gp120 (92TH023 strain) linked to the transmembrane anchoring portion of gp41 (LAI strain) without the gp41 ectodomain] co-administered with AIDSVAX B/E (Global Solutions for Infectious Diseases) [a recombinant bivalent combination of HIV-1 gp120 subtype B (MN strain) and subtype E (A244 strain) proteins adjuvanted with alum] (group 1), (2) AIDSVAX B/E alone (group 2), or (3) ALVAC-HIV (vCP1521) alone (group 3) delivered intramuscularly at weeks 0 and 24.

All assays were performed blinded to treatment status. All 162 clinical trial participants were studied for the presence of IgG subclass binding antibody responses using enzyme-linked immunosorbent assays (ELISA). The first 70 subjects enrolled in this study were profiled for vaccine-induced humoral responses in binding antibody multiplex assays (BAMA), linear peptide microarray assays, ADCP assays, and ADCC assays as outlined below. Infectious virion capture assays were performed on samples from 15 subjects. Selection of study subjects was done by block randomization. The study arms were relatively well distributed, with the cohort of 70 participants consisting of 20 vaccine and 3 placebo recipients from Group 1 [ALVAC-HIV/AIDSVAX B/E (combination) regimen], 18 vaccine and 6 placebo recipients from Group 2 (AIDSVAX only regimen), and 19 vaccine and 4 placebo recipients from Group 3 (ALVAC-HIV only regimen).

### Enzyme linked immunosorbent assays

Plasma was assessed for HIV-1 Env binding IgG1-IgG4 by enzyme linked immunosorbent assay (ELISA). Briefly, U-bottom 2HB plates (Thermo Fisher Scientific) were coated with A244 gD gp120, gp70 V1V2 92TH023 and gp70 V1V2 case A2 scaffold proteins at 3 μg/mL in Dulbecco’s phosphate buffered saline (D-PBS) (Sigma-Aldrich) at 4°C overnight. Wells were washed three times with wash buffer (PBS, 0.1% Tween 20 and 0.1% Thimerosal, pH 7.4) using Microplate Washer ELx405 (BioTek) and blocked with 220 μL/well of blocking buffer (D-PBS, 5% skim milk) for two hours at room temperature. Plasma samples were initially diluted at 1:100 (A244 gD gp120) or 1:25 (gp70 scaffold proteins) in blocking buffer and two-fold serial dilutions were performed. Each well received 100 μL of diluted specimen and plates were incubated for 2 hours at room temperature. All wells were washed 5 times with wash buffer and 100 μL/well of mouse anti-human IgG1, IgG2, IgG3, or IgG4 (1 μg/mL) (Bethyl Laboratories) was added and incubated for 1 hour at room temperature. Plates were washed with wash buffer and horseradish peroxidase (HRP)-conjugated anti-mouse IgG (Bethyl laboratories) was added and incubated for 1 hour at room temperature. Wells were washed and ABTS ELISA substrate (KPL) was added for 1 hour and then plates were read at 405 nm absorbance on a Spectramax 340 PC ELISA reader (Molecular Devices). Geometric mean titers (GMT) were calculated from final dilutions giving a positive result (405 nm absorbance > cut-off value; 2X background level, wells without capturing antigen) from individual samples.

### Binding antibody multiplex assays (BAMA)

Plasma binding antibody reactivity to HIV-1 Envelope gp120, gp140, and V1V2 breadth panel antigens, representing geographic and global HIV-1 diversity [23], was determined by a custom BAMA as previously described [2,7,23,82]. Recombinant envelope proteins were provided by Drs. Hua-Xin Liao, James W. Peacock, Barton F. Haynes (Duke Human Vaccine Institute Laboratory of Protein Expression, Duke Protein Production Facility) and Abraham Pinter (New Jersey Medical School, Rutgers University) and produced as previously described [10,23,83]. Briefly, plasma samples were diluted at 1:50 (for IgG) or 1:40 (for IgG1-IgG4 and IgA1-IgA2 subclasses) and incubated with carboxylated fluorescent bead sets (Bio-Rad) covalently coupled to Env proteins. HIV-1-specific antibody subclasses were detected with biotinylated mouse anti-human IgG (Southern Biotech), IgG1 (BD Pharmingen), IgG2 (Southern Biotech), IgG3 (Calbiochem), IgG4 (BD Pharmingen), IgA1 (Southern Biotech), or IgA2 (Southern Biotech), followed by washing and incubation with streptavidin phycoerythrin (BD Pharmingen). Beads were acquired on a Bio-Plex 200 instrument (Bio-Rad, Inc.), with antibody binding expressed as mean fluorescence intensity (MFI). Positive controls included polyclonal human HIV immune globulin (HIVIG, NIH AIDS Reagent Program) and purified IgG1-IgG4 human myeloma protein (Sigma) titrations and single-point monoclonal antibody (mAb) controls VRC01 IgG1 (provided by John Mascola, NIH Vaccine Research Center), 2G12 IgG, 17b IgG, and 7B2 IgA. CH58 IgG1 and IgG3 mAbs were used as standard controls for V1V2 antigen binding to calculate μg/mL mAb equivalent concentrations using four parameter logistic (4PL) curve analysis (Bio-Plex Manager Software). HIV-1 seronegative human serum (NHS, Sigma), blank (uncoupled) beads, MulVgp70_His6 (empty gp70 scaffold) coupled beads, and a blank well on each assay plate were included as negative controls. Samples were batch-tested in duplicate, with plasma from different time points from the same subject examined side-by-side in the same assay run. CD4bs responses were measured by examining differential binding to a resurfaced stabilized core (RSC3) and a RSC3 protein containing an isoleucine (I) deletion at amino acid position 371 and proline (P) to asparagine (N) mutation at position 363 (RSC3Δ371/P363N), which abrogates binding of antibodies antigenically related to VRC01 [84]. CD4i-directed activity was detected by measuring differential binding to YU2 gp120 wild-type (YU2 gp120 WT) and a mutant of YU2 gp120 WT containing an I to arginine (R) substitution at amino acid 420 (YU2 gp120 I420R) shown to reduce binding of 17b-like CD4i antibodies. A differential binding ratio (wild-type to mutant) of greater than or equal to 2.5 defined positive plasma reactivity for CD4bs and CD4i. All assays were performed under Good Clinical Laboratory Practice (GCLP) conditions with Levey Jennings antigen tracking for positive control binding. Sample data that passed assay quality control criteria were used for statistical analyses and plots. Final response magnitude was background [plate matched blank well control and well matched blank bead] subtracted MFI, and was reported as MFI-Blank. Samples were considered positive if MFI-Blank values were greater than the 95^th^ percentile MFI-Blank of all baseline samples [and a minimum of 100], and three-fold over the MFI [before and after blank bead subtraction] of the matched baseline sample. HIV-1 Env gp120, gp140, and V1V2 breadth scores were calculated as the mean of the MFI-Blank of the individual antigens in a given panel, for samples that had data for all antigens in the panel. For calculations of area under the magnitude breadth curves (AUC-MB) MFI-Blank values for negative responses were set to 1, and values greater than 22000 were set to 22000, to help analyze data from positive responses, and those within the linear range of the assay.

### Linear epitope mapping

Mapping of plasma IgG to linear epitopes was performed by peptide microarray as previously described [2,82], with modifications. Array slides (JPT Peptide Technologies GmbH) were printed with peptide libraries of 15mers overlapping by 12, covering 7 consensus full length HIV-1 Env gp160 (clade A, B, C, D, group M, CRF1, and CRF2) and 6 HIV-1 virus strain gp120 (AE.A244, AE.TH023, B.MN, C.1086, C.TV1, C.ZM651) sequences. Slides were blocked in PBS containing 1% powdered milk, 5% normal goat serum, and 0.5% Tween and incubated with plasma diluted at 1:50, followed by washing and incubation with goat anti-human IgG AlexaFluor 647 (Jackson Immunoresearch) diluted 1:500 in blocking buffer. Arrays were analyzed on an Axon GenePix 4300 scanner (Molecular Devices) using a PMT setting of 580, 100% laser power. Scanned images were analyzed using Genepix Pro 7 Software (Molecular Devices) and microarray data processed using R package pepStat [5]. Binding intensity against each peptide was defined as the median of the fluorescence signal for three replicates. Values for RV305 post-vaccination samples were baseline subtracted from matched RV144 pre-vaccination sample values and expressed as log2 intensity. Positivity criteria for peptide binding was defined as greater than the 95^th^ percentile of all baseline samples and three-fold over participant baseline. Magnitude breadth (MB) scores for binding to linear epitopes were computed in R using the mdw package using the maximum diversity weight method.

### Antibody purification and depletion

Depletion and purification of IgG antibodies from plasma samples was performed using a Protein G HP MultiTrap column (GE Healthcare, Inc.) as previously described [85].

### Antibody-dependent cellular phagocytosis (ADCP) assays

ADCP assays were performed as previously described, with modifications [37]. Uptake of antibody opsonized, Env antigen-coated fluorescent beads or fluorescently labeled HIV-1 by THP-1 cells (ATCC; #TIB-201) or human primary monocytes, respectively, was measured by flow cytometry. Briefly, A244 gp120- or Con S gp140 Env-conjugated NeutrAvidin fluorescent 1-μm beads (Thermo Fisher) or infectious HIV-1_92TH023_-Tomato [86,87] (provided by Dr. Thomas J. Hope, Northwestern University) were incubated with diluted plasma for 2 hours to form immune complexes. THP-1 cells or primary monocytes pre-treated with anti-human CD4 were added, followed by spinoculation for 1 hour and incubation at 37°C for 1 hour to allow for phagocytosis to occur. Cells were fixed with 2% paraformaldehyde and analyzed on a flow cytometer. Phagocytosis scores were calculated as the mean fluorescence intensity (MFI) multiplied by the percentage of bead or virus positive THP-1 cells or monocytes for the test plasma or mAb divided by the no antibody control. Criteria for a positive response was that the ADCP score for the post-vaccination sample had to be greater than the 95^th^ percentile baseline antigen-specific cutoff and three times over the participant-specific baseline. If no participant-matched baseline sample was available to test, a cutoff of three times over the median of all baselines was used. CD4 binding-site-specific mAb CH31 was included as a positive control, and influenza receptor binding site mAb CH65 was used as a negative control. Assays were performed in duplicate or triplicate for each sample.

For a subset of thirty-eight participants, Con S gp140 bead area under the curve (AUC) phagocytosis scores were calculated using a 5 point, 5-fold dose response curve from 50 μg/mL to 0.08 μg/mL. AUC values from vaccination groups 1 and 2 were aggregated and analyzed for correlation with Con S gp140 IgG subclass BAMA MFI.

### Monoclonal antibody isolation, expression, and virion phagocytosis

Native IgG3 monoclonal antibodies were isolated by antigen-specific single memory B cell sorting of peripheral blood mononuclear cells from RV305 vaccinees using A244 gp120 fluorescently labeled probes as previously described [27]. Immunoglobulin variable heavy and light chain genes were amplified by reverse transcriptase-polymerase chain reaction, de novo synthesized, and cloned into IgG3 wild-type and IgG1_4A expression vectors as previously described [37,88]. Epitope-matched subclass-specific monoclonal antibodies were tested for ADCP potency against HIV-1_CM235_-tdTomato fluorescently labeled virions in human monocytes [30,89,90].

### Antibody-dependent infectious virion capture assays

Infectious virion capture assays were performed as previously described [13]. Briefly, purified IgG was mixed with HIV-1_TH023_ virions to form antibody-virion immune complexes, which were passed through a protein G column. Flow through virus was added to TZM-bl cells and capture was calculated as the percentage of flow through virus infection of TZM-bl cells relative to a no antibody control. Positive controls included HIVIG, V1V2-directed quaternary mAb PG9 IgG1, CD4 binding site-directed mAb CH31 IgG1, and gp41-directed mAb 7B2 IgG1. Respiratory Syncytial Virus (RSV)-specific mAb Palivizumab IgG1 was included as a negative control in each assay. Assays were performed in duplicate for each study participant. The positivity cutoff for each virus was defined as the mean plus two standard deviations of the fold change from baseline (RV305 week 0) to week 2 and week 26 (combined) for the placebo group.

### Antibody-dependent cellular cytotoxicity assays

The ability of plasma samples to mediate killing of CEM.NKR_CCR5_ CD4^+^ target cells (NIH AIDS Reagent Program) infected with HIV-1 AE.TH023.6, AE.427299, AE.CM235 infectious molecular clone (IMC) viruses (Gen. Bank Accession Numbers AF009393.1, JN944655, AF259954.1, respectively) containing a *Renilla* Luciferase (Luc) reporter gene [91] was measured using a Luc-based ADCC assay as previously described [12]. Plasma was tested after 5-fold serial dilutions starting at 1:50. Peripheral blood mononuclear cells (PBMC) from a normal healthy seronegative donor with a Fc gamma receptor IIIa F/V phenotype, collected by leukapheresis, [92] were used as a source of NK cells and incubated with target cells at an effector to target ratio of 30:1. Killing was measured as reduction in Luciferase activity compared to control wells that contained effector and target cells without plasma. ADCC responses were considered positive if the percent killing activity was greater than 15% and antibody titer exceeded 1:200. The negative control was mAb palivizumab (Synagis), which mediates ADCC [93] but is specific for respiratory syncytial virus. Positive controls were a cocktail of HIV-1 mAbs (HIV-1 mAb mix) demonstrated to mediate ADCC [A32, 2G12 [94], CH44, and 7B2] [41].

### RV305 T cell data set

The RV305 T cell data set, published in a prior manuscript [25], was used to analyze fold change of cellular responses across the vaccination groups. HIV-1 Gag-specific (subtype B LAI strain), Env-specific (CRF01_AE TH023 strain), and V2-specific CD4^+^ and CD8^+^ T cell function was assessed using intracellular cytokine staining as previously described [25]. A set of six markers/cytokines were measured: CD154, CD107, interleukin-2 (IL-2), interleukin-4 (IL-4), interferon gamma (IFN-γ), and tumor necrosis factor alpha (TNF-α). Functionality and polyfunctionality scores for each subject were determined using COMPASS (http://github.com/RGLab/COMPASS) [9].

### RV305 neutralization data set

The RV305 neutralization data set, originating from a prior publication [25], examined neutralizing antibody titers in plasma against CRF01_AE 92TH023 and B.MN Env pseudoviruses in TZM-bl cells as previously described. Measures of neutralization were utilized to compare immune profiles of the different vaccine groups.

### Statistical analysis

A pre-specified statistical analysis plan outlined the primary analysis hypotheses and objectives for this study. For comparison of response magnitude, between two time points, the Wilcoxon signed rank Test was used, and for comparisons between groups, the Wilcoxon rank sum test was used. The Benjamini and Hochberg (1995) false discovery rate method was used to correct for multiple comparisons for the 70 tests of the pre-determined comparisons included in the primary analysis. All tests were two-sided, and differences were considered statistically significant for FDR-corrected p < 0.05. Primary analyses were performed in SAS (Copyright (c) 2002–2012 by SAS Institute Inc., Cary, NC, USA. SAS (r) Proprietary Software 9.4 (TS1M3)) or R statistical software (R Core Team (2018). URL https://www.R-project.org/). R package ggplot2 was used for generating plots.

## Supporting information

S1 Fig Linear epitope mapping of RV305 plasma. (**A**) Heatmap of median RV305 week 2 (left panel) and week 26 (right panel) plasma binding to peptides of different gp120 strains (median for 70 RV305 participants). Each row represents a single strain included in the epitope mapping peptide library. Group median binding magnitude to each strain is shown. Color intensity is proportional to binding intensity, with the darker colors indicating higher binding and the lighter colors indicating lower binding. (**B**) Spider plots demonstrating breadth of C1.2, V2 hotspot (V2.hs), V3, and C5.2 targeting against consensus and virus strain peptides for each vaccine group at RV305 week 2 (top panel) and week 26 (bottom panel). (**C**) Log_2_ fold difference in linear peptide binding to vaccine strain sequence (B.MN) at RV305 week 26 compared to RV305 week 2. Horizontal bar pointing to the left of the x = 0 line (solid black vertical line) indicates a higher response magnitude measured at RV305 week 2 compared to RV305 week 26; horizontal bar pointing the right indicates a higher response magnitude measured at RV305 week 26 versus RV305 week 2. (**D**) Log_2_ fold difference in B.MN linear peptide binding of group 1 versus group 2 plasma at RV305 weeks 2 and 26. Horizontal bar pointing to the left of the x = 0 line indicates a higher response magnitude measured in the AIDSVAX B/E only group; horizontal bar pointing to the right indicates a higher response magnitude measured in the Combination group.
(TIF)

S2 Fig IgG binding magnitudes and response rates to vaccine-matched linear epitopes at RV305 week 2 and week 26. A response was considered positive if the intensity of binding to each peptide was greater than the 95^th^ percentile of all baseline sample binding to the peptide and the binding signal (log 2 fold difference over baseline) was greater than 1.58, which represents a 3-fold difference post- and pre-immunization. Filled circles represent positive responders, and open triangles represent non-responders.
(TIF)

S3 Fig Lack of RSC3-reactive CD4 binding site IgG antibodies elicited in RV144 and RV305. Plasma IgG antibodies to the CD4 binding site (CD4bs) region of gp120 were assessed by BAMA using resurfaced stabilized gp120 core (RSC3) and CD4 binding site knockout mutant (RSC3 delta371/P363N) proteins that detect CD4bs antibodies of the VRC01 class. Differential binding plots display BAMA FI-Bkgd-Blank values for IgG binding to RSC3 (y-axis) and RSC3 delta371/P363N mutant (x-axis) proteins at RV144 week 26 and RV305 weeks 0, 2, 24, 26, 48, and 72. The diagonal dashed gray line indicates a wild-type to mutant binding ratio of 2.5 (cut-off for positivity). The CD4bs broadly neutralizing antibody VRC01 was used as a positive control for RSC3/RSC3 delta371/P363N differential binding. The glycan-specific monoclonal antibody 2G12 was used as a negative control, which showed equivalent binding to wild-type versus mutant. Open gray triangles represent non-responders with differential binding ratios of ≤ 2.5, indicating the lack of RSC3-reactive CD4bs specificities. Response rate (percent responders over the total number of participants analyzed) is shown at the top of each plot.
(TIFF)

S4 Fig Longitudinal YU2 gp120 WT-reactive CD4i IgG binding antibodies elicited in RV144 and RV305 (related to Fig 1). Prevalence of CD4-induced (CD4i) IgG antibodies among RV305 vaccine and placebo recipients. Differential binding plots displaying BAMA FI-Bkgd-Blank values for IgG binding to YU2 gp120 WT (y-axis) and YU2 gp120 I420R mutant (x-axis) proteins at RV144 week 26 and RV305 weeks 0 and 2. The diagonal dashed gray line indicates a wild-type to mutant binding ratio of 2.5 (cut-off for positivity). The CD4-induced (CD4i) monoclonal antibody 17b was used as a positive control for YU2 gp120 WT/I420R differential binding. Colored symbols represent positive responders with differential binding ratios of ≥ 2.5, indicating the presence of CD4i specificities. Response rate (percent responders over the total number of participants analyzed) is shown at the top of each plot.
(TIFF)

S5 Fig Plasma IgG concentrations to V1V2 antigens associated with RV144 vaccine efficacy. V1V2 concentrations were extrapolated by 4-parameter logistic (4-PL) regression of V2-specific monoclonal antibody CH58 standard curve titrations run in each BAMA. Concentrations are plotted in μg/mL for (A) placebo recipients (i.e. RV144 vaccine group only) and (B) for each RV305 vaccine group across the studied immunogenicity time points, with each dot representing the concentration for a single plasma sample. The midline of the box plot denotes the median concentration, and the ends of the box plot denote the 25^th^ and 75^th^ percentiles among positive responses.
(TIFF)

S6 Fig Magnitude-breadth of linear V2 hotspot binding response. MB plots characterizing IgG breadth (response rate) (y-axis) and magnitude (log2 fold difference post second boost / post first boost binding intensity) (x-axis) against a cross-clade panel of 11 V2 hotspot peptides measured by linear peptide microarray. Solid curves are the median MB at RV305 week 2 (red) and week 26 (green). AUC values summarize the MB at a given time point across the entire range of binding values on the x-axis.
(TIFF)

S7 Fig Delayed boosting with ALVAC-HIV/AIDSVAX B/E or AIDSVAX B/E increases IgG and IgG1 breadth. (**A**) HIV-1-specific plasma IgG levels to vaccine-matched gp120 Envelope (A244 D11 gp120, MN gp120, 92TH023 gp120) and V1V2 (AE.A244 V1V2 tags) primary antigens determined by BAMA. Response rates (top panel) and binding magnitudes (bottom panel) are plotted for each group at two weeks post final RV144 vaccination (week 26) and two weeks post first and second RV305 boosts (weeks 2 and 26, respectively). Box plots (bottom panel) denote the median (midline) and interquartile ranges among positive responses. Solid dots depict positive responders, and open gray triangles depict non responders. (**B**) IgG breadth scores against the global gp120 breadth panel, calculated for each participant as the mean of the MFIs across the 8 antigens in the panel. Box and whisker plots show the median and interquartile ranges of scores across the Combination and AIDSVAX B/E only groups. Differences in median breadth scores (aggregated for the Combination and AIDSVAX B/E only groups) across post RV144 boost (week 26) and RV305 boost time points (weeks 2 and 26) were assessed using the two-sided Wilcoxon Signed Rank Test (Table 1). (**C**) Magnitude-breadth plot of IgG binding antibody responses to the gp120 breadth panel among Combination and AIDSVAX B/E only positive responders at 2 weeks post final RV144 vaccination (week 26) and 2 weeks post first and second RV305 boosts (weeks 2 and 26). Breadth is defined as the proportion of antigens in the 8 antigen gp120 breadth panel (y-axis) with log10 (MFI-blank) greater than the threshold on the x-axis. Dashed lines display MB curves for each individual plasma sample measured at RV144 week 26 (turquoise), RV305 week 2 (pink), and RV305 week 26 (orange). Solid bold lines show the median MB among positive responders at each immunization time point. AUC values summarize the MB at a given time point across the entire range of MFI values. (**D**) Vaccine-elicited IgG1 binding antibody response rates and binding magnitudes to the four antigens in the primary analysis measured by BAMA. (**E**) gp120 and (**F**) gp140 (**G**) gp70 V1V2 IgG1 MB plots, comprising data combined for Combination (ALVAC-HIV + AIDVAX B/E) and AIDSVAX B/E positive responses.
(TIF)

S8 Fig Durability of Env gp120 IgG responses. Fold decline in IgG antibody binding magnitude to gp120 antigens from two weeks post last RV305 boost (week 26) to 12 months post last boost (week 72). Results are presented as log_2_ fold change, with the midline of the box plots indicating median and ends of the box plots indicating the 25^th^ and 75^th^ percentiles. The whiskers denote the minimum and maximum data points no more than 1.5 times the interquartile range (IQR). Data points that lie outside of the median ± 1.5 times the IQR are shown as black dots. Criteria for the fold (wk26/wk72) calculation: 1) response is positive at week 26, 2) MFI < 23000 at week 26, 3) MFI > 100 at week 72. Antigens with greater than or equal to 6 data points meeting this criteria for both the Combination (ALVAC-HIV/AIDSVAX B/E) and AIDSVAX B/E only groups are plotted for each vaccine boost regimen. Proximity of the bar to the y-axis indicates better durability.
(TIFF)

S9 Fig ELISA plasma IgG subclass (IgG1-IgG4) binding antibody responses against HIV gp120 A244gD, gp70V1V2 92TH023 scaffold, and gp70 CaseA2 scaffold proteins. ELISA IgG1-IgG4 (A-D, respectively) reciprocal titers to A244 gD, gp70V1V2 92TH023 scaffold, and gp70V1V2 Case A2 scaffold are shown for RV305 recipients (n = 162) randomized to receive two additional boosts of either ALVAC-HIV + AIDSVAX B/E (group 1, n = 45 vaccinees), AIDSVAX B/E (group 2, n = 45 vaccinees), or ALVAC-HIV (group 3, n = 45 vaccinees), 6–8 years post last RV144 vaccination (RV305 regimen). Arrows indicate time of immunizations. Error bars depict 95% confidence intervals.
(TIF)

S10 Fig Highest V1V2 IgG3 binding magnitude observed among ALVAC-HIV/AIDSVAX B/E only recipients. Dot plots illustrating IgG3 positive responders at RV305 weeks 26 and 72 (2 weeks post second boost and one year post second boost, respectively). Vertical lines separate Env (gp140 +gp120), V1V2, and other (V3 and Gag) antigens.
(TIFF)

S11 Fig Durability of IgG3 responses. Percent decline of IgG3 response magnitude (MFI) to V1V2, gp120, and gp140 antigens from RV305 week 26 to week 72 (2 weeks post second boost to 12 months post second boost). Box plots depict the median, 25^th^ and 75^th^ percentiles, and the whiskers denote minimum and maximum data points no more than 1.5 times the interquartile range (IQR). Data points that lie outside of the median ± 1.5 times the IQR are shown as black dots. The criteria for the percent decline calculation are that the response is positive at week 26 and the MFI is less than 23,000 at week 26. Antigens with at least three data points meeting this criteria for the Combination (ALVAC-HIV/AIDSVAX B/E) and AIDSVAX B/E only groups are plotted. Proximity of the bar to the y-axis indicates better durability.
(TIFF)

S12 Fig Low frequency IgG2 and lack of IgA2 induced in RV305. (**A**) IgG2 and (**B**) IgA2 responses to vaccine strain immunogens in the RV144 prime (92TH023 gp120) and boost (A244 gp120, MN gp120) and CRF01_AE A244 V1V2 are plotted for RV305 recipients in each group at RV144 peak (week 26) and two weeks after the first and second RV305 boosts (weeks 2 and 26). The number of positive responders for each subclass is shown over the total number of individuals analyzed at each time point, represented as a bar graph displaying percent responders. Box plots depict the median (midline) and 25^th^ and 75^th^ percentiles, with the colored symbols indicating the response magnitude for each positive responder. Open gray triangles indicate non-responders.
(TIF)

S13 Fig Antibody-dependent cellular cytotoxicity (ADCC) activity reported as AUC. A luciferase-based ADCC assay was used to profile the ability of plasma antibodies from 70 RV305 participants to mediate killing of cells infected with AE.CM235, AE.TH023, and AE.427299 HIV-1 infectious molecular clones. RV305 baseline and post first and second boost (RV305 weeks 2 and 26, respectively) plasma was diluted 5-fold, and results are reported as AUC for each time point, plotted for each vaccine boost regimen. Box plots show the distribution of AUC values; the midline denotes the median and the ends of the box plot denote the 25^th^ and 75^th^ percentiles for positive responses. Solid dots depict positive responders, and open gray triangles depict non responders. Gray lines connect samples from the same donor.
(TIFF)

S1 Table BAMA IgG response rates and group median binding magnitudes (MFI) against gp120, gp140, V1V2, V3, CD4 inducible, CD4 binding site, and Gag HIV-1 regions. (PDF)

S2 Table Linear peptide epitope definition. (PDF)

S3 Table Plasma IgG binding to linear V2 hotspot at RV305 weeks 2 and 26. (PDF)

S4 Table Median IgG CH58 μg/mL equivalent V1V2 concentrations among positive responders. (PDF)

S5 Table Percent of positive responders in all three RV305 vaccine groups for IgG gp70_B.CaseA_V1_V2 CH58 μg/mL equivalent concentrations above 2.98 μg/mL concentration associated with decreased HIV-1 risk. (PDF)

S6 Table Median IgG breadth scores per vaccination group. (PDF)

S7 Table BAMA plasma binding IgG1 response rates and group median binding magnitudes (MFI) to gp120, gp140, V1V2, V3, CD4 inducible, CD4 binding site, and Gag HIV-1 regions. (PDF)

S8 Table BAMA plasma binding IgG3 response rates and group median binding magnitudes (MFI) to gp120, gp140, V1V2, V3, CD4 inducible, CD4 binding site, and Gag HIV-1 regions. (PDF)

S9 Table Median IgG3 breadth scores by group and study week. (PDF)

S10 Table Median IgG3 CH58 μg/mL equivalent V1V2 concentrations. (PDF)

S11 Table BAMA plasma binding IgG2 response rates and group median binding magnitudes (MFI) to gp120, gp140, V1V2, V3, CD4 inducible, CD4 binding site, and Gag HIV-1 regions. (PDF)

S12 Table BAMA plasma binding IgG4 response rates and group median binding magnitudes (MFI) to gp120, gp140, V1V2, V3, CD4 inducible, CD4 binding site, and Gag HIV-1 regions. (PDF)

S13 Table IgA1 percent responders and group median binding magnitudes (MFI) by antigen, study week, and group. (PDF)

S14 Table IgA1 binding magnitude comparison at RV144 week 26 and RV305 week 2. (PDF)

S15 Table IgA2 percent responders by antigen, study week, and group. (PDF)
