# Supplementary figures and images for: Viral vector delivered immunogen focuses HIV-1 antibody specificity and increases durability of the circulating antibody recall response
Source: PLoS Pathog. 2023 May 31;19(5):e1011359. doi: 10.1371/journal.ppat.1011359 (PMC10284421; doi:10.1371/journal.ppat.1011359)

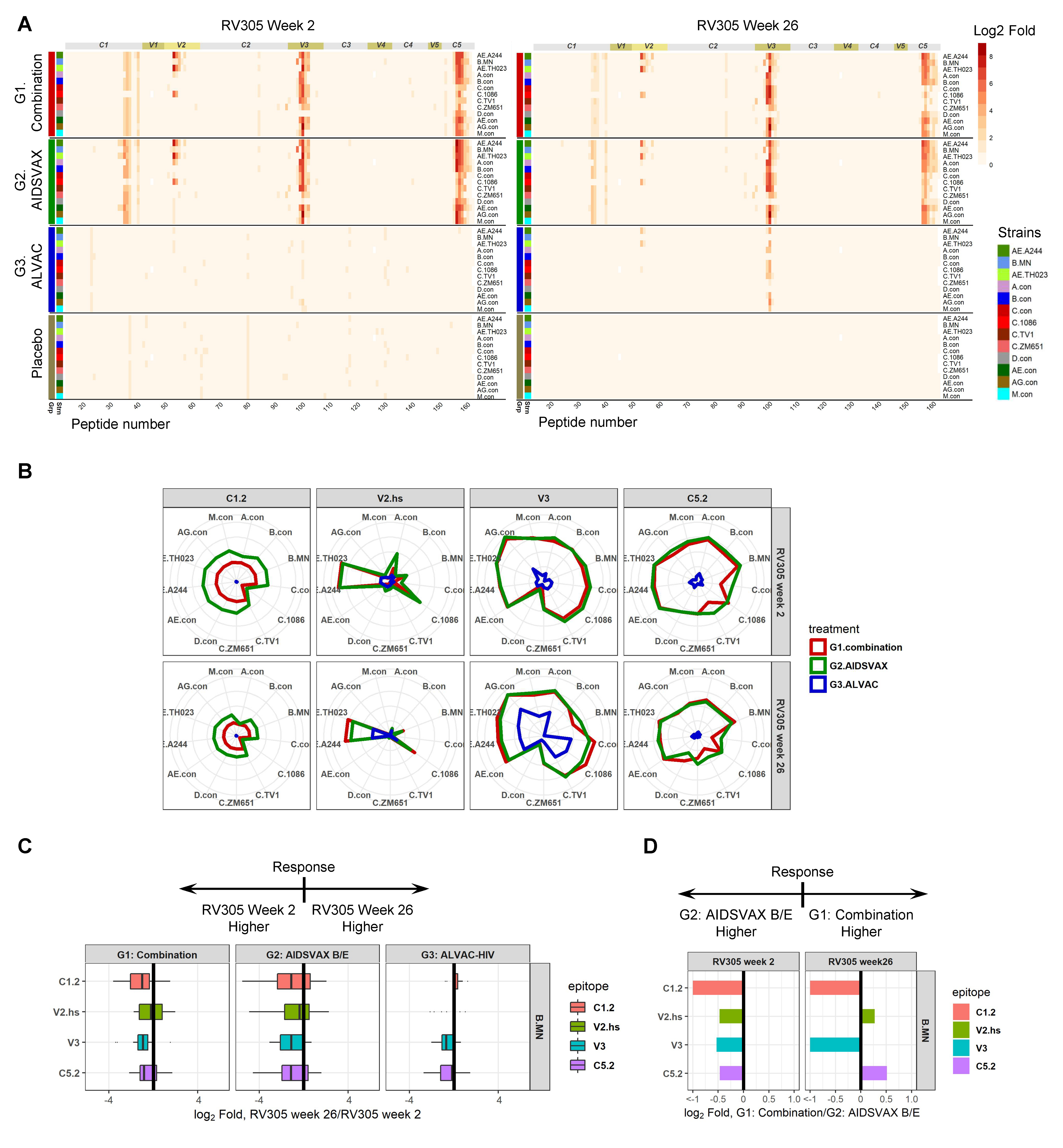

Supplement: S1 Fig — (A) Heatmap of median RV305 week 2 (left panel) and week 26 (right panel) plasma binding to peptides of different gp120 strains (median for 70 RV305 participants). Each row represents a single strain included in the epitope mapping peptide library. Group median binding magnitude to each strain is shown. Color intensity is proportional to binding intensity, with the darker colors indicating higher binding and the lighter colors indicating lower binding. (B) Spider plots demonstrating breadth of C1.2, V2 hotspot (V2.hs), V3, and C5.2 targeting against consensus and virus strain peptides for each vaccine group at RV305 week 2 (top panel) and week 26 (bottom panel). (C) Log2 fold difference in linear peptide binding to vaccine strain sequence (B.MN) at RV305 week 26 compared to RV305 week 2. Horizontal bar pointing to the left of the x = 0 line (solid black vertical line) indicates a higher response magnitude measured at RV305 week 2 compared to RV305 week 26; horizontal bar pointing the right indicates a higher response magnitude measured at RV305 week 26 versus RV305 week 2. (D) Log2 fold difference in B.MN linear peptide binding of group 1 versus group 2 plasma at RV305 weeks 2 and 26. Horizontal bar pointing to the left of the x = 0 line indicates a higher response magnitude measured in the AIDSVAX B/E only group; horizontal bar pointing to the right indicates a higher response magnitude measured in the Combination group. (TIF) [file ppat.1011359.s001.tif]

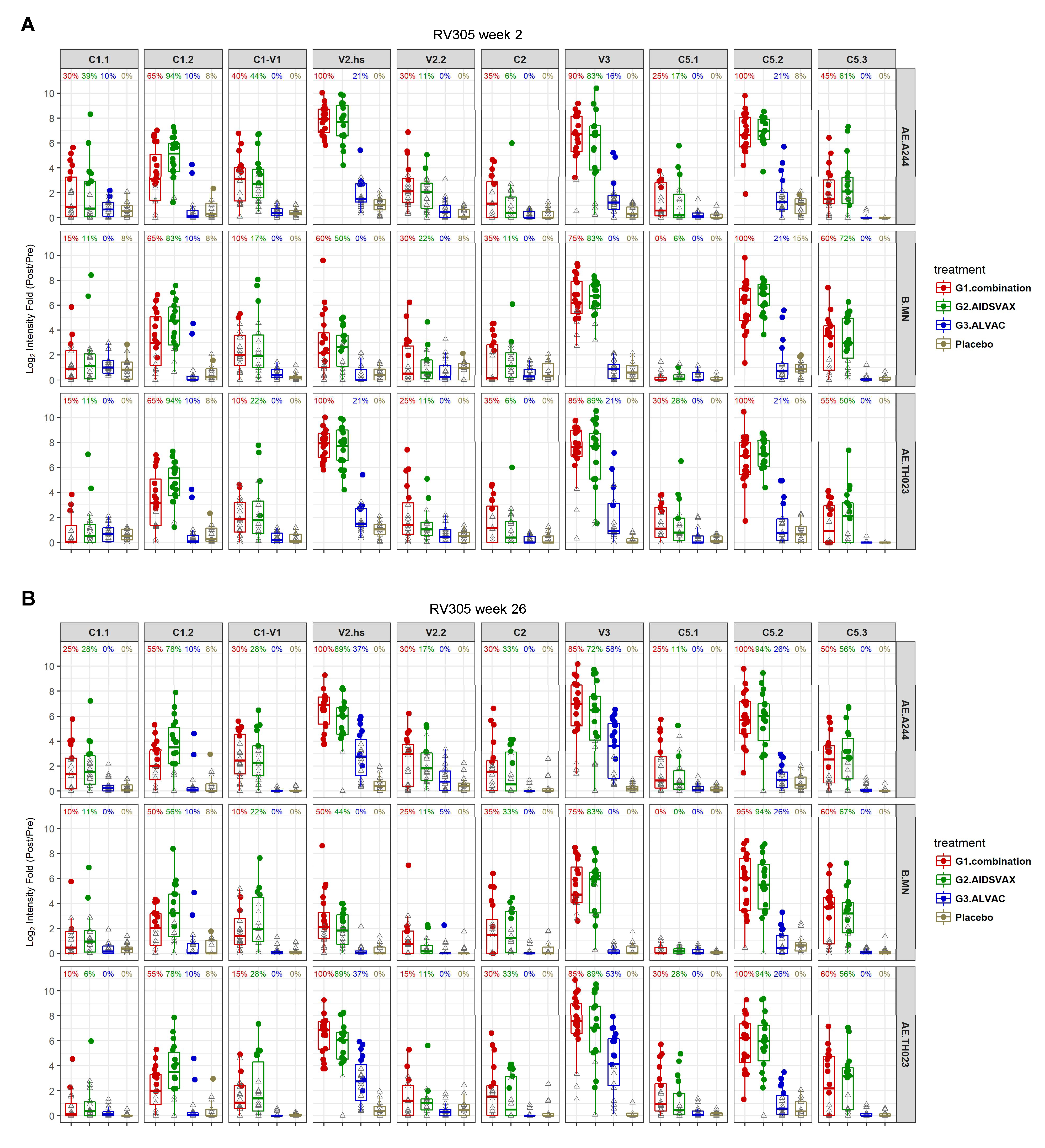

Supplement: S2 Fig — A response was considered positive if the intensity of binding to each peptide was greater than the 95th percentile of all baseline sample binding to the peptide and the binding signal (log 2 fold difference over baseline) was greater than 1.58, which represents a 3-fold difference post- and pre-immunization. Filled circles represent positive responders, and open triangles represent non-responders. (TIF) [file ppat.1011359.s002.tif]

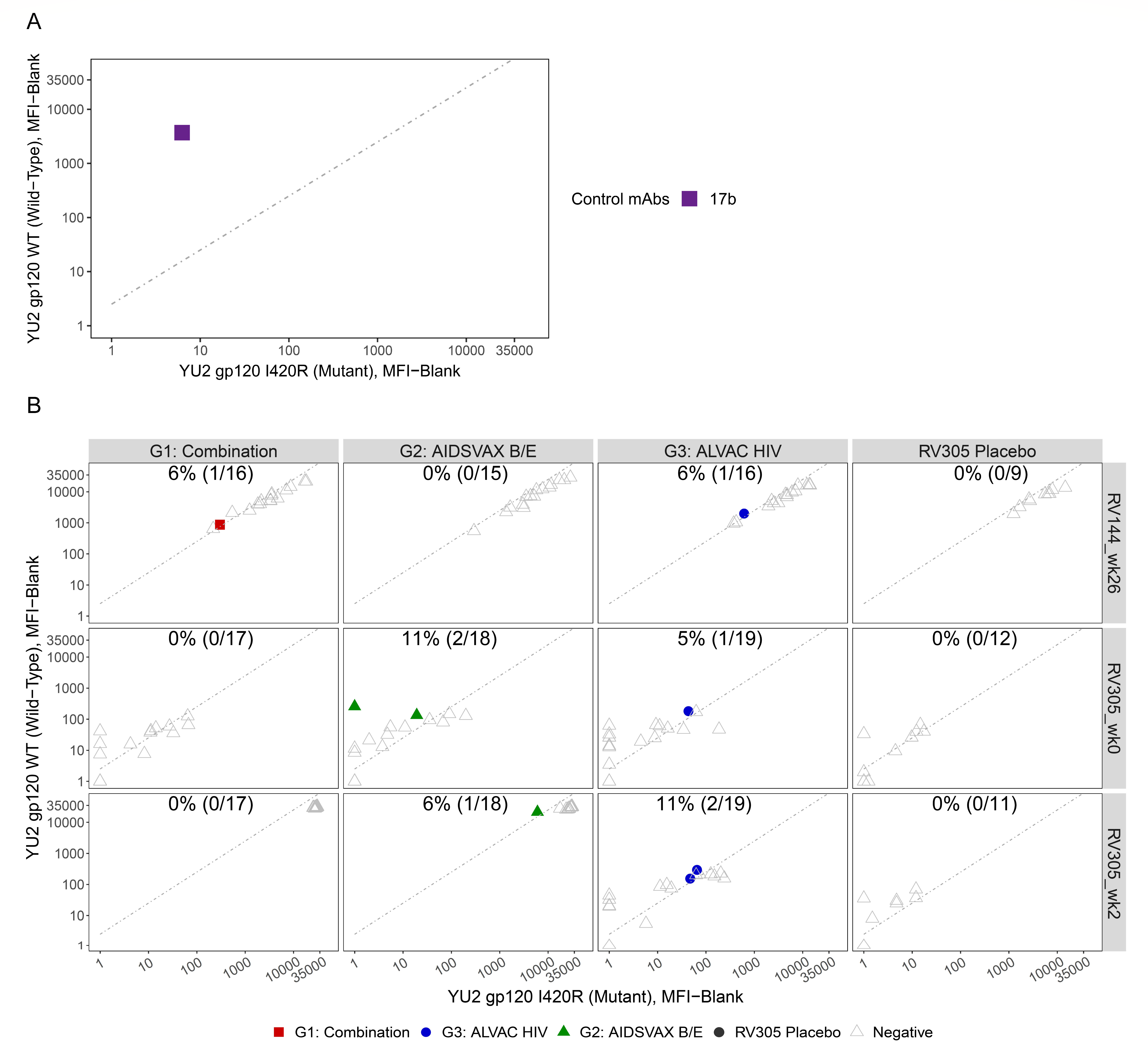

Supplement: S4 Fig — Prevalence of CD4-induced (CD4i) IgG antibodies among RV305 vaccine and placebo recipients. Differential binding plots displaying BAMA FI-Bkgd-Blank values for IgG binding to YU2 gp120 WT (y-axis) and YU2 gp120 I420R mutant (x-axis) proteins at RV144 week 26 and RV305 weeks 0 and 2. The diagonal dashed gray line indicates a wild-type to mutant binding ratio of 2.5 (cut-off for positivity). The CD4-induced (CD4i) monoclonal antibody 17b was used as a positive control for YU2 gp120 WT/I420R differential binding. Colored symbols represent positive responders with differential binding ratios of ≥ 2.5, indicating the presence of CD4i specificities. Response rate (percent responders over the total number of participants analyzed) is shown at the top of each plot. (TIFF) [file ppat.1011359.s004.tiff]

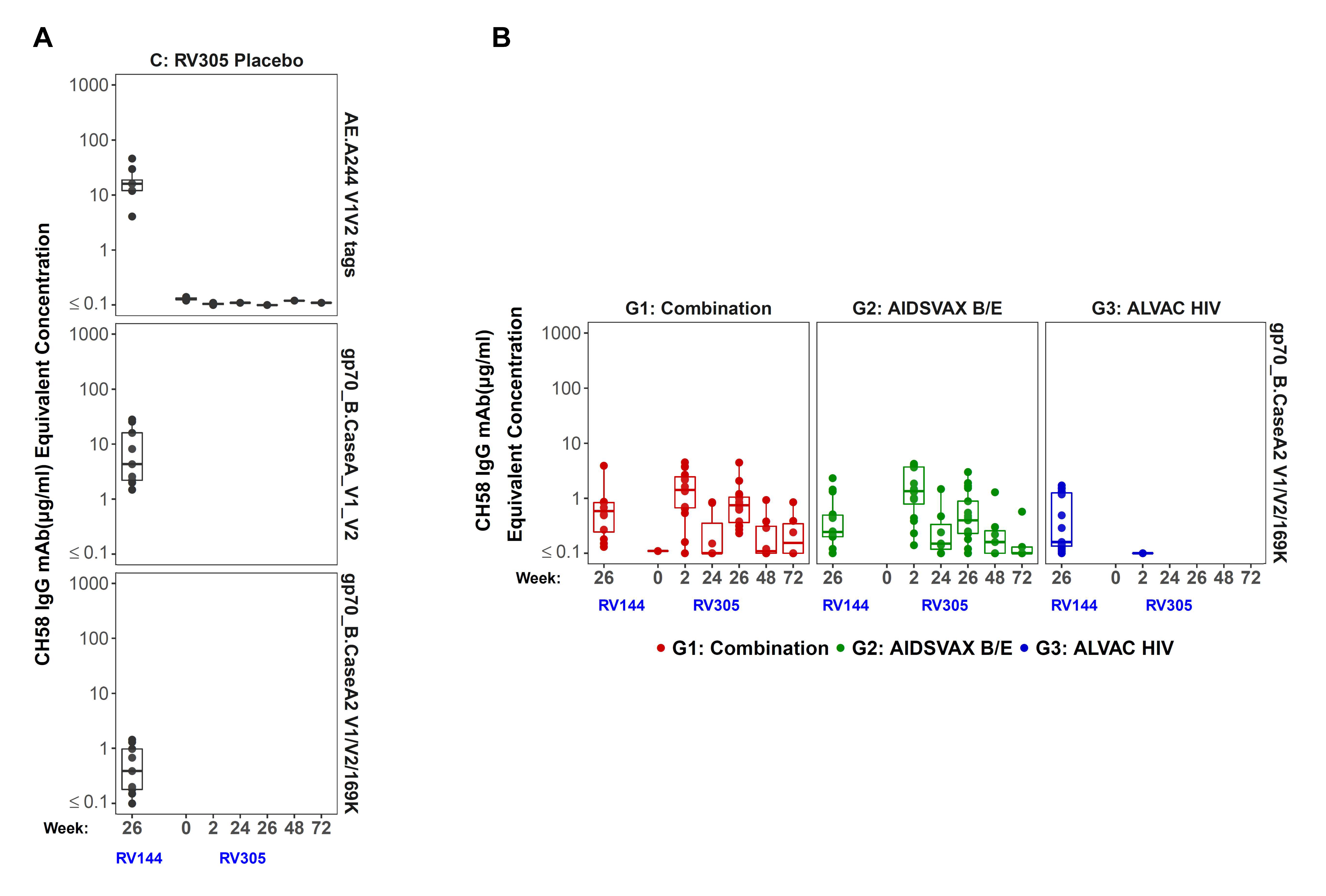

Supplement: S5 Fig — V1V2 concentrations were extrapolated by 4-parameter logistic (4-PL) regression of V2-specific monoclonal antibody CH58 standard curve titrations run in each BAMA. Concentrations are plotted in μg/mL for (A) placebo recipients (i.e. RV144 vaccine group only) and (B) for each RV305 vaccine group across the studied immunogenicity time points, with each dot representing the concentration for a single plasma sample. The midline of the box plot denotes the median concentration, and the ends of the box plot denote the 25th and 75th percentiles among positive responses. (TIFF) [file ppat.1011359.s005.tiff]

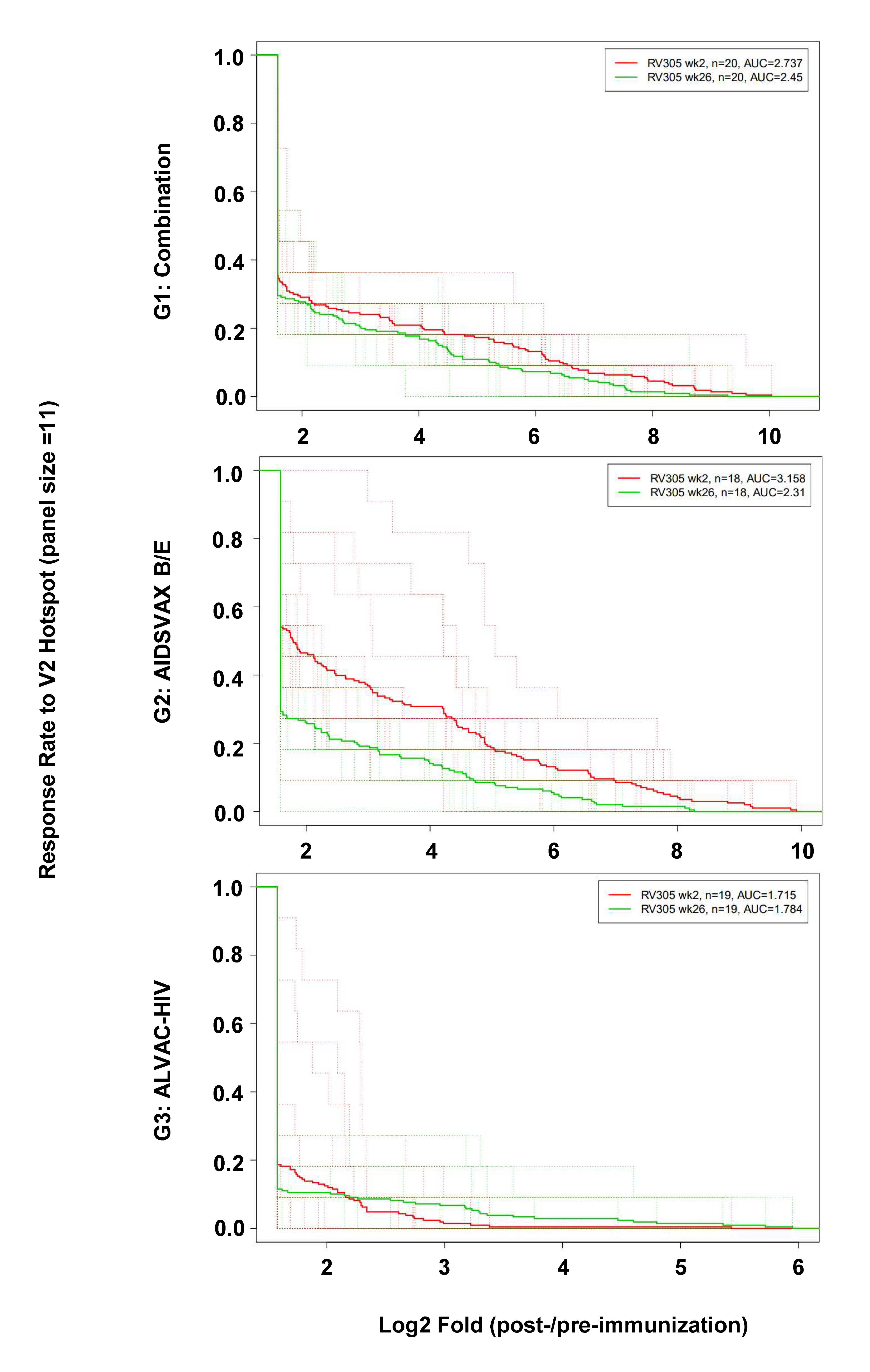

Supplement: S6 Fig — MB plots characterizing IgG breadth (response rate) (y-axis) and magnitude (log2 fold difference post second boost / post first boost binding intensity) (x-axis) against a cross-clade panel of 11 V2 hotspot peptides measured by linear peptide microarray. Solid curves are the median MB at RV305 week 2 (red) and week 26 (green). AUC values summarize the MB at a given time point across the entire range of binding values on the x-axis. (TIFF) [file ppat.1011359.s006.tiff]

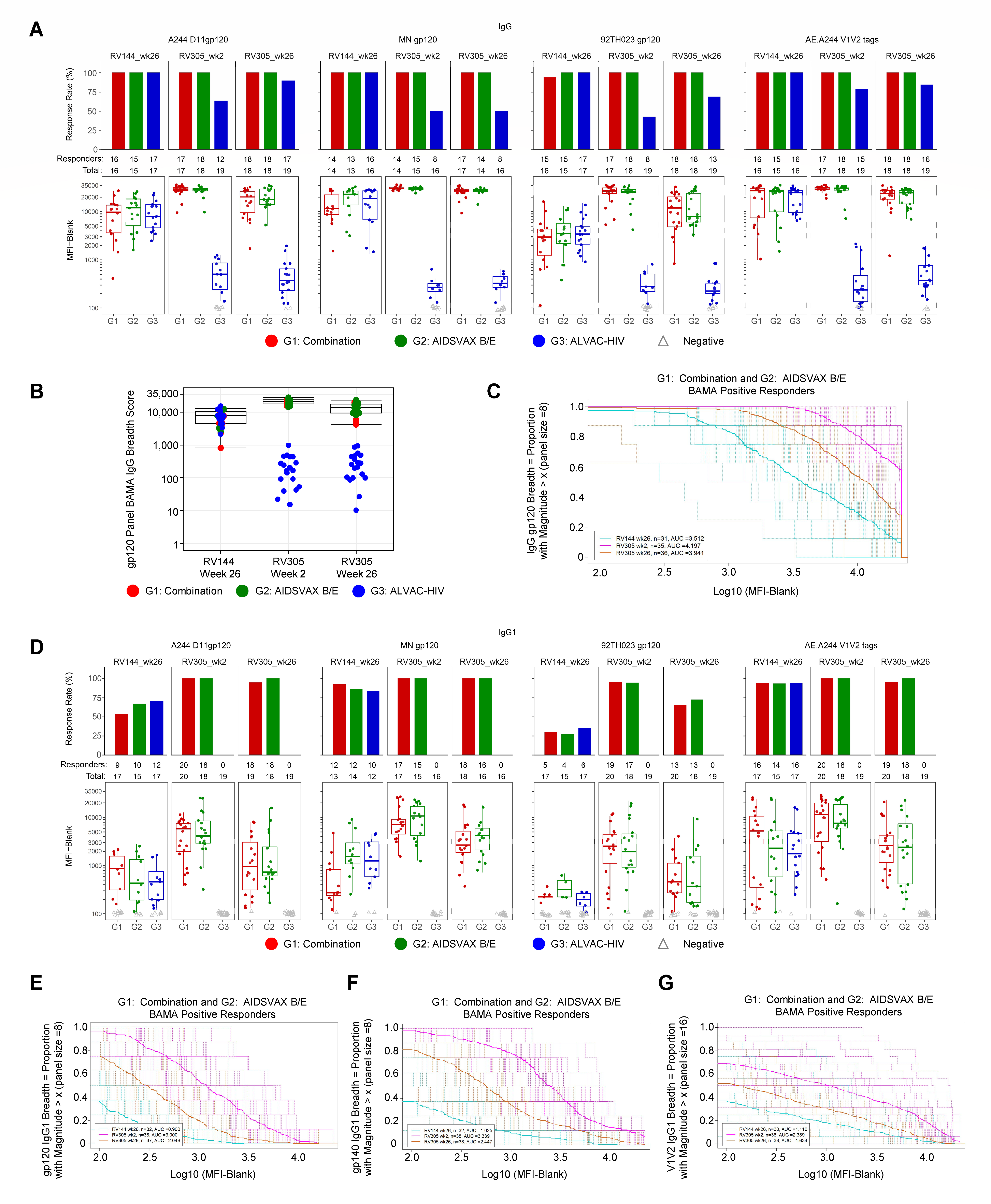

Supplement: S7 Fig — (A) HIV-1-specific plasma IgG levels to vaccine-matched gp120 Envelope (A244 D11 gp120, MN gp120, 92TH023 gp120) and V1V2 (AE.A244 V1V2 tags) primary antigens determined by BAMA. Response rates (top panel) and binding magnitudes (bottom panel) are plotted for each group at two weeks post final RV144 vaccination (week 26) and two weeks post first and second RV305 boosts (weeks 2 and 26, respectively). Box plots (bottom panel) denote the median (midline) and interquartile ranges among positive responses. Solid dots depict positive responders, and open gray triangles depict non responders. (B) IgG breadth scores against the global gp120 breadth panel, calculated for each participant as the mean of the MFIs across the 8 antigens in the panel. Box and whisker plots show the median and interquartile ranges of scores across the Combination and AIDSVAX B/E only groups. Differences in median breadth scores (aggregated for the Combination and AIDSVAX B/E only groups) across post RV144 boost (week 26) and RV305 boost time points (weeks 2 and 26) were assessed using the two-sided Wilcoxon Signed Rank Test (Table 1). (C) Magnitude-breadth plot of IgG binding antibody responses to the gp120 breadth panel among Combination and AIDSVAX B/E only positive responders at 2 weeks post final RV144 vaccination (week 26) and 2 weeks post first and second RV305 boosts (weeks 2 and 26). Breadth is defined as the proportion of antigens in the 8 antigen gp120 breadth panel (y-axis) with log10 (MFI-blank) greater than the threshold on the x-axis. Dashed lines display MB curves for each individual plasma sample measured at RV144 week 26 (turquoise), RV305 week 2 (pink), and RV305 week 26 (orange). Solid bold lines show the median MB among positive responders at each immunization time point. AUC values summarize the MB at a given time point across the entire range of MFI values. (D) Vaccine-elicited IgG1 binding antibody response rates and binding magnitudes to the four antigens in the primary an [file ppat.1011359.s007.tif]

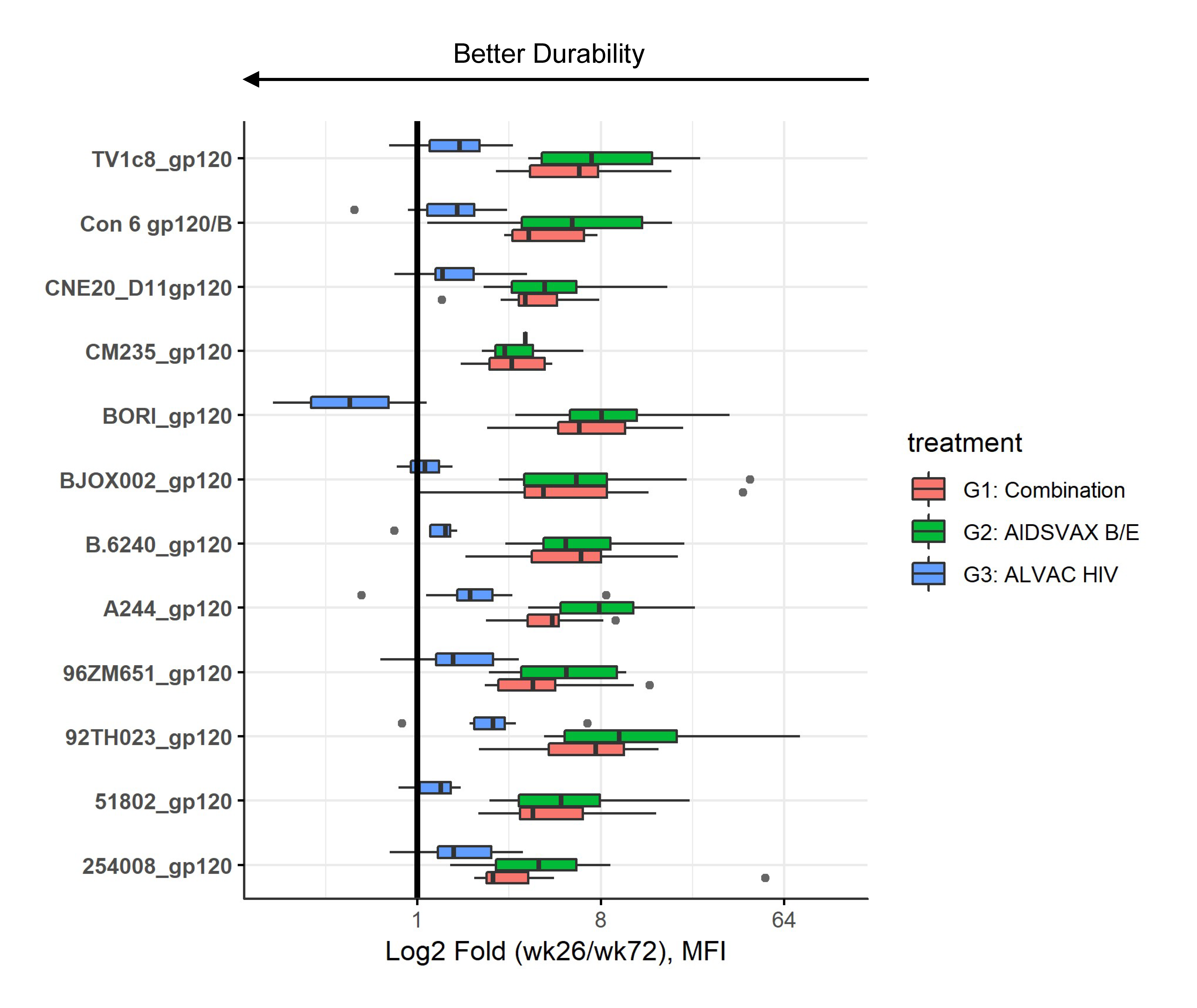

Supplement: S8 Fig — Fold decline in IgG antibody binding magnitude to gp120 antigens from two weeks post last RV305 boost (week 26) to 12 months post last boost (week 72). Results are presented as log2 fold change, with the midline of the box plots indicating median and ends of the box plots indicating the 25th and 75th percentiles. The whiskers denote the minimum and maximum data points no more than 1.5 times the interquartile range (IQR). Data points that lie outside of the median ± 1.5 times the IQR are shown as black dots. Criteria for the fold (wk26/wk72) calculation: 1) response is positive at week 26, 2) MFI < 23000 at week 26, 3) MFI > 100 at week 72. Antigens with greater than or equal to 6 data points meeting this criteria for both the Combination (ALVAC-HIV/AIDSVAX B/E) and AIDSVAX B/E only groups are plotted for each vaccine boost regimen. Proximity of the bar to the y-axis indicates better durability. (TIFF) [file ppat.1011359.s008.tiff]

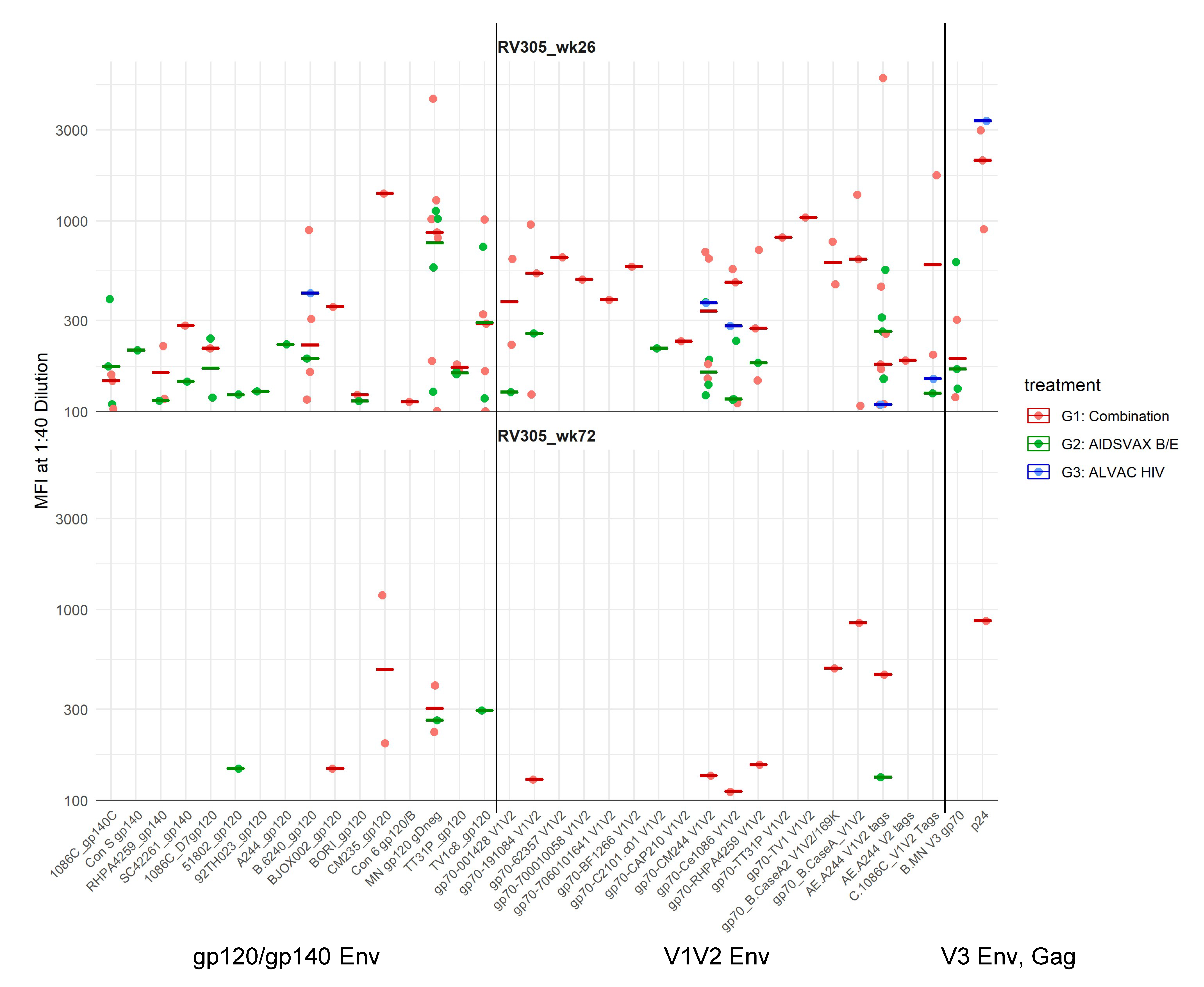

Supplement: S10 Fig — Dot plots illustrating IgG3 positive responders at RV305 weeks 26 and 72 (2 weeks post second boost and one year post second boost, respectively). Vertical lines separate Env (gp140 +gp120), V1V2, and other (V3 and Gag) antigens. (TIFF) [file ppat.1011359.s010.tiff]

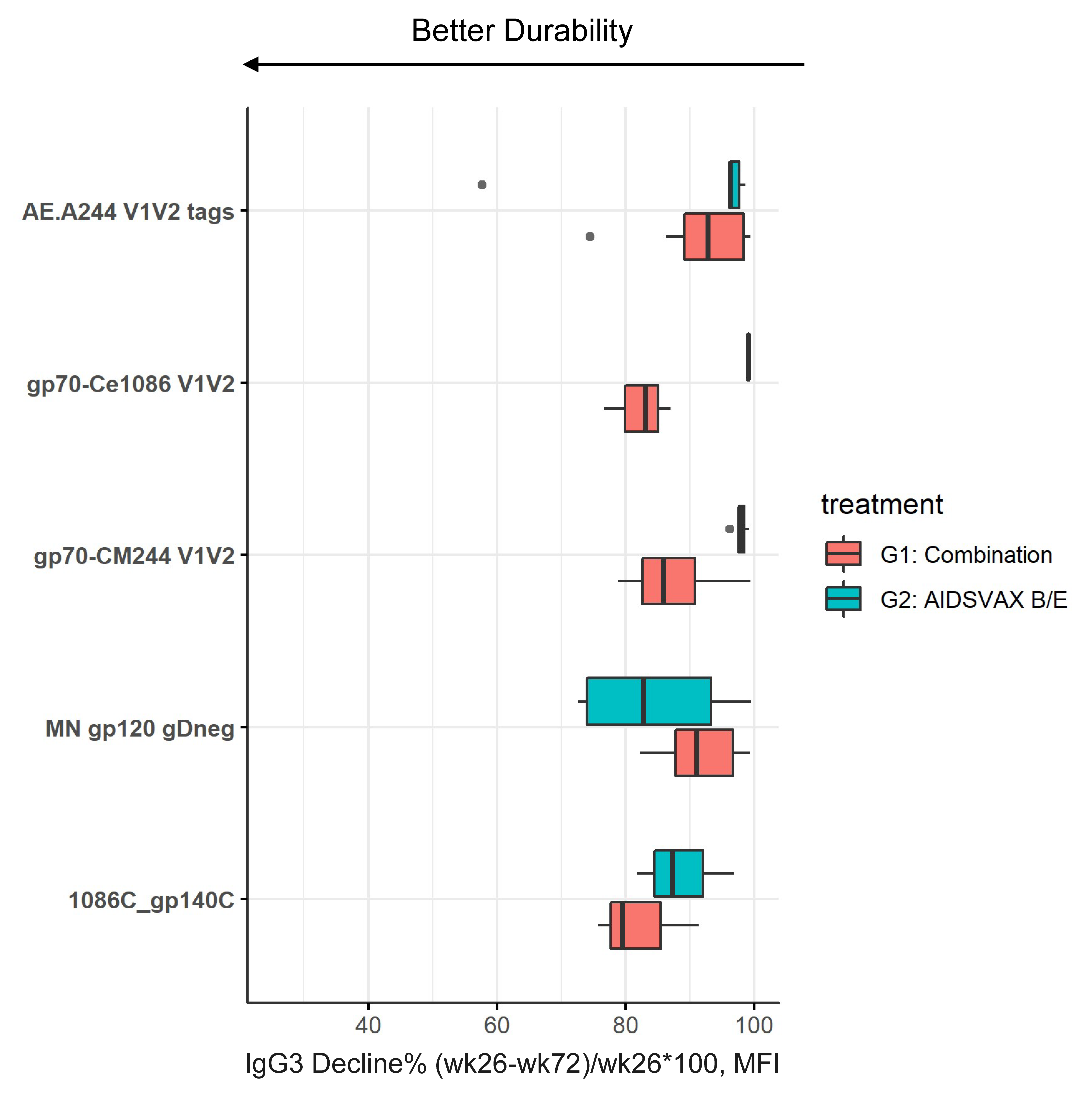

Supplement: S11 Fig — Percent decline of IgG3 response magnitude (MFI) to V1V2, gp120, and gp140 antigens from RV305 week 26 to week 72 (2 weeks post second boost to 12 months post second boost). Box plots depict the median, 25th and 75th percentiles, and the whiskers denote minimum and maximum data points no more than 1.5 times the interquartile range (IQR). Data points that lie outside of the median ± 1.5 times the IQR are shown as black dots. The criteria for the percent decline calculation are that the response is positive at week 26 and the MFI is less than 23,000 at week 26. Antigens with at least three data points meeting this criteria for the Combination (ALVAC-HIV/AIDSVAX B/E) and AIDSVAX B/E only groups are plotted. Proximity of the bar to the y-axis indicates better durability. (TIFF) [file ppat.1011359.s011.tiff]

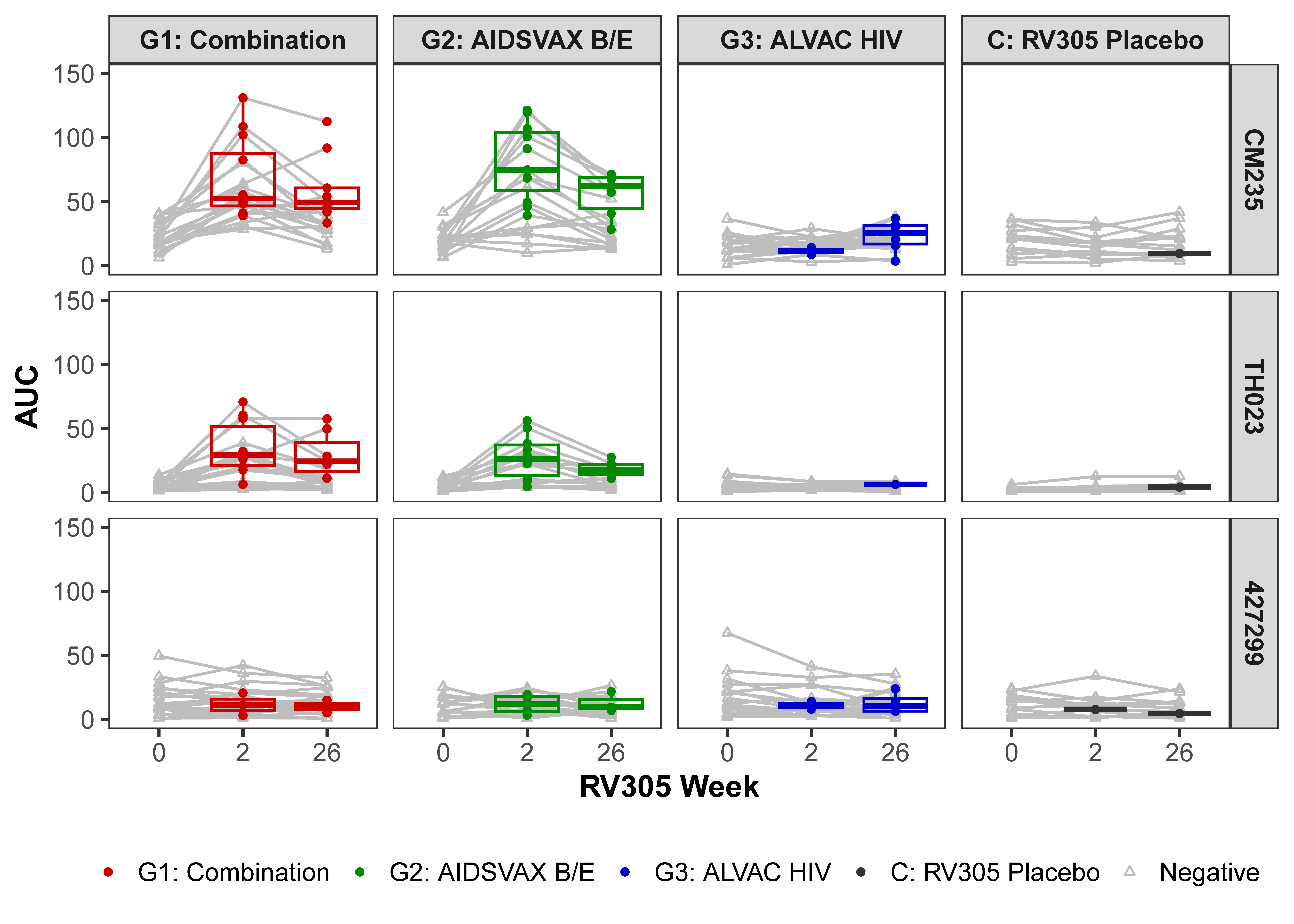

Supplement: S13 Fig — A luciferase-based ADCC assay was used to profile the ability of plasma antibodies from 70 RV305 participants to mediate killing of cells infected with AE.CM235, AE.TH023, and AE.427299 HIV-1 infectious molecular clones. RV305 baseline and post first and second boost (RV305 weeks 2 and 26, respectively) plasma was diluted 5-fold, and results are reported as AUC for each time point, plotted for each vaccine boost regimen. Box plots show the distribution of AUC values; the midline denotes the median and the ends of the box plot denote the 25th and 75th percentiles for positive responses. Solid dots depict positive responders, and open gray triangles depict non responders. Gray lines connect samples from the same donor. (TIFF) [file ppat.1011359.s013.tiff]
