# Supplementary material for: Viral vector delivered immunogen focuses HIV-1 antibody specificity and increases durability of the circulating antibody recall response
Source: PLoS Pathog. 2023 May 31;19(5):e1011359. doi: 10.1371/journal.ppat.1011359 (PMC10284421; doi:10.1371/journal.ppat.1011359)
Supplement: S1 Table — (PDF) [file ppat.1011359.s014.pdf]

S1 Table. BAMA plasma IgG response rates and group median binding magnitudes (MFI) against gp120, gp140, V1V2, V3, CD4 inducible, CD4 binding site, and Gag HIV-1 regions.

					Group 1: Combination		Group 2: AIDSVAX B/E		Group 3: ALVAC-HIV		RV305_Placebo Group	
**Isotype**	**Clade**	**Env Region**	**Antigen**	**Study Week**	**Response Rate** ***(Responders/ Total)***	**Median MFI** ***Responders***	**Response Rate** ***(Responders/ Total)***	**Median MFI** ***Responders***	**Response Rate** ***(Responders/ Total)***	**Median MFI** ***Responders***	**Response Rate** ***(Responders/ Total)***	**Median MFI** ***Responders***
IgG	A	gp120	51802_D11gp120.avi/293F	RV144_wk26	87.5 (14/16)	1241	100 (15/15)	1378	100 (17/17)	1603	100 (9/9)	1507
IgG	A	gp120	51802_D11gp120.avi/293F	RV305_wk0	0.0 (0/17)		0.0 (0/18)		10.5 (2/19)	110	0.0 (0/12)	
IgG	A	gp120	51802_D11gp120.avi/293F	RV305_wk2	100 (17/17)	11094	100 (18/18)	12067	36.8 (7/19)	144	0.0 (0/12)	
IgG	A	gp120	51802_D11gp120.avi/293F	RV305_wk24	88.9 (16/18)	1136	100 (18/18)	1264	21.1 (4/19)	130	0.0 (0/12)	
IgG	A	gp120	51802_D11gp120.avi/293F	RV305_wk26	100 (18/18)	4348	100 (18/18)	5270	42.1 (8/19)	183	0.0 (0/12)	
IgG	A	gp120	51802_D11gp120.avi/293F	RV305_wk48	100 (17/17)	1177	100 (18/18)	1366	31.6 (6/19)	156	0.0 (0/12)	
IgG	A	gp120	51802_D11gp120.avi/293F	RV305_wk72	94.4 (17/18)	967	100 (18/18)	971	16.7 (3/18)	154	0.0 (0/12)	
IgG	B	gp120	B.6240_D11gp120/293F	RV144_wk26	100 (16/16)	2390	100 (15/15)	4821	100 (17/17)	2874	100 (9/9)	4314
IgG	B	gp120	B.6240_D11gp120/293F	RV305_wk0	11.8 (2/17)	132	27.8 (5/18)	146	10.5 (2/19)	206	8.3 (1/12)	164
IgG	B	gp120	B.6240_D11gp120/293F	RV305_wk2	100 (17/17)	21216	100 (18/18)	23199	47.4 (9/19)	196	8.3 (1/12)	160
IgG	B	gp120	B.6240_D11gp120/293F	RV305_wk24	94.4 (17/18)	1904	100 (18/18)	1698	42.1 (8/19)	214	8.3 (1/12)	188
IgG	B	gp120	B.6240_D11gp120/293F	RV305_wk26	100 (18/18)	10374	100 (18/18)	12415	63.2 (12/19)	252	8.3 (1/12)	168
IgG	B	gp120	B.6240_D11gp120/293F	RV305_wk48	100 (17/17)	2567	100 (18/18)	2223	52.6 (10/19)	175	8.3 (1/12)	162
IgG	B	gp120	B.6240_D11gp120/293F	RV305_wk72	94.4 (17/18)	1907	100 (18/18)	1672	38.9 (7/18)	189	8.3 (1/12)	150
IgG	B	gp120	BORI_D11gp120.avi/293F	RV144_wk26	87.5 (14/16)	1347	100 (15/15)	2019	100 (17/17)	1373	100 (9/9)	1510
IgG	B	gp120	BORI_D11gp120.avi/293F	RV305_wk0	0.0 (0/17)		5.6 (1/18)	135	0.0 (0/19)		0.0 (0/12)	
IgG	B	gp120	BORI_D11gp120.avi/293F	RV305_wk2	100 (17/17)	14565	100 (18/18)	15278	5.3 (1/19)	804	0.0 (0/12)	
IgG	B	gp120	BORI_D11gp120.avi/293F	RV305_wk24	88.9 (16/18)	908	100 (18/18)	1036	5.3 (1/19)	113	0.0 (0/12)	
IgG	B	gp120	BORI_D11gp120.avi/293F	RV305_wk26	100 (18/18)	4966	100 (18/18)	5601	21.1 (4/19)	126	0.0 (0/12)	
IgG	B	gp120	BORI_D11gp120.avi/293F	RV305_wk48	100 (17/17)	1037	94.4 (17/18)	1233	15.8 (3/19)	154	0.0 (0/12)	
IgG	B	gp120	BORI_D11gp120.avi/293F	RV305_wk72	94.4 (17/18)	773	94.4 (17/18)	876	16.7 (3/18)	126	0.0 (0/12)	
IgG	B	gp120	MN gp120 gDneg/293F	RV144_wk26	100 (14/14)	11570	100 (13/13)	23045	100 (16/16)	18892	100 (9/9)	19927
IgG	B	gp120	MN gp120 gDneg/293F	RV305_wk0	18.8 (3/16)	138	33.3 (5/15)	307	14.3 (2/14)	342	8.3 (1/12)	121
IgG	B	gp120	MN gp120 gDneg/293F	RV305_wk2	100 (14/14)	30096	100 (15/15)	29522	50.0 (8/16)	271	8.3 (1/12)	115
IgG	B	gp120	MN gp120 gDneg/293F	RV305_wk24	94.4 (17/18)	7103	100 (18/18)	7282	47.4 (9/19)	177	8.3 (1/12)	117
IgG	B	gp120	MN gp120 gDneg/293F	RV305_wk26	100 (17/17)	27400	100 (14/14)	28020	50.0 (8/16)	328	16.7 (2/12)	112
IgG	B	gp120	MN gp120 gDneg/293F	RV305_wk48	100 (16/16)	8561	100 (16/16)	10597	43.8 (7/16)	205	8.3 (1/12)	116
IgG	B	gp120	MN gp120 gDneg/293F	RV305_wk72	94.1 (16/17)	5788	100 (15/15)	4536	50.0 (8/16)	144	0.0 (0/12)	

					Group 1: Combination		Group 2: AIDSVAX B/E		Group 3: ALVAC-HIV		RV305_Placebo Group	
Isotype	Clade	Env Region	Antigen	Study Week	Response Rate (Responders/ Total)	Median MFI Responders	Response Rate (Responders/ Total)	Median MFI Responders	Response Rate (Responders/ Total)	Median MFI Responders	Response Rate (Responders/ Total)	Median MFI Responders
IgG	CRF01_AE	gp120	92TH023 gp120 gDneg 293F mon	RV144_wk26	93.8 (15/16)	2985	100 (15/15)	3522	100 (17/17)	3437	100 (9/9)	3848
IgG	CRF01_AE	gp120	92TH023 gp120 gDneg 293F mon	RV305_wk0	0.0 (0/17)		11.1 (2/18)	211	0.0 (0/19)		0.0 (0/12)	
IgG	CRF01_AE	gp120	92TH023 gp120 gDneg 293F mon	RV305_wk2	100 (17/17)	26957	100 (18/18)	26091	42.1 (8/19)	283	0.0 (0/12)	
IgG	CRF01_AE	gp120	92TH023 gp120 gDneg 293F mon	RV305_wk24	88.9 (16/18)	1215	100 (18/18)	1394	26.3 (5/19)	146	0.0 (0/12)	
IgG	CRF01_AE	gp120	92TH023 gp120 gDneg 293F mon	RV305_wk26	100 (18/18)	12094	100 (18/18)	7928	68.4 (13/19)	225	0.0 (0/12)	
IgG	CRF01_AE	gp120	92TH023 gp120 gDneg 293F mon	RV305_wk48	100 (17/17)	1693	100 (18/18)	1826	36.8 (7/19)	278	0.0 (0/12)	
IgG	CRF01_AE	gp120	92TH023 gp120 gDneg 293F mon	RV305_wk72	94.4 (17/18)	1481	100 (18/18)	1011	38.9 (7/18)	119	0.0 (0/12)	
IgG	CRF01_AE	gp120	A244 D11gp120_avi	RV144_wk26	100 (16/16)	9754	100 (15/15)	12060	100 (17/17)	7989	100 (9/9)	9974
IgG	CRF01_AE	gp120	A244 D11gp120_avi	RV305_wk0	23.5 (4/17)	181	38.9 (7/18)	250	26.3 (5/19)	222	16.7 (2/12)	112
IgG	CRF01_AE	gp120	A244 D11gp120_avi	RV305_wk2	100 (17/17)	29423	100 (18/18)	28980	63.2 (12/19)	505	16.7 (2/12)	105
IgG	CRF01_AE	gp120	A244 D11gp120_avi	RV305_wk24	94.4 (17/18)	3064	100 (18/18)	3514	52.6 (10/19)	267	8.3 (1/12)	117
IgG	CRF01_AE	gp120	A244 D11gp120_avi	RV305_wk26	100 (18/18)	20359	100 (18/18)	17801	89.5 (17/19)	376	16.7 (2/12)	109
IgG	CRF01_AE	gp120	A244 D11gp120_avi	RV305_wk48	100 (17/17)	4322	100 (18/18)	4490	68.4 (13/19)	339	8.3 (1/12)	117
IgG	CRF01_AE	gp120	A244 D11gp120_avi	RV305_wk72	94.4 (17/18)	3648	100 (18/18)	3009	55.6 (10/18)	261	8.3 (1/12)	105
IgG	CRF01_AE	gp120	CM235 gp120	RV144_wk26	0.0 (0/16)		13.3 (2/15)	12907	5.9 (1/17)	13565	22.2 (2/9)	11579
IgG	CRF01_AE	gp120	CM235 gp120	RV305_wk0	0.0 (0/17)		0.0 (0/18)		0.0 (0/19)		0.0 (0/12)	
IgG	CRF01_AE	gp120	CM235 gp120	RV305_wk2	82.4 (14/17)	26696	100 (18/18)	24789	0.0 (0/19)		0.0 (0/12)	
IgG	CRF01_AE	gp120	CM235 gp120	RV305_wk24	11.1 (2/18)	12265	5.6 (1/18)	11370	0.0 (0/19)		0.0 (0/12)	
IgG	CRF01_AE	gp120	CM235 gp120	RV305_wk26	55.6 (10/18)	18384	66.7 (12/18)	15450	5.3 (1/19)	9863	0.0 (0/12)	
IgG	CRF01_AE	gp120	CM235 gp120	RV305_wk48	11.8 (2/17)	20153	5.6 (1/18)	11824	0.0 (0/19)		0.0 (0/12)	
IgG	CRF01_AE	gp120	CM235 gp120	RV305_wk72	5.6 (1/18)	27984	5.6 (1/18)	13201	0.0 (0/18)		0.0 (0/12)	
IgG	CRF07_BC	gp120	BJOX002_D11gp120.avi/293F	RV144_wk26	87.5 (14/16)	846	100 (15/15)	1063	100 (17/17)	739	100 (9/9)	1487
IgG	CRF07_BC	gp120	BJOX002_D11gp120.avi/293F	RV305_wk0	0.0 (0/17)		0.0 (0/18)		0.0 (0/19)		0.0 (0/12)	
IgG	CRF07_BC	gp120	BJOX002_D11gp120.avi/293F	RV305_wk2	100 (17/17)	5964	100 (18/18)	5910	10.5 (2/19)	179	0.0 (0/12)	
IgG	CRF07_BC	gp120	BJOX002_D11gp120.avi/293F	RV305_wk24	88.9 (16/18)	656	94.4 (17/18)	824	10.5 (2/19)	147	0.0 (0/12)	
IgG	CRF07_BC	gp120	BJOX002_D11gp120.avi/293F	RV305_wk26	94.4 (17/18)	2424	100 (18/18)	2482	26.3 (5/19)	113	0.0 (0/12)	
IgG	CRF07_BC	gp120	BJOX002_D11gp120.avi/293F	RV305_wk48	100 (17/17)	640	94.4 (17/18)	800	21.1 (4/19)	138	0.0 (0/12)	
IgG	CRF07_BC	gp120	BJOX002_D11gp120.avi/293F	RV305_wk72	94.4 (17/18)	507	88.9 (16/18)	581	11.1 (2/18)	141	0.0 (0/12)	
IgG	CRF07_BC	gp120	CNE20_D11gp120.avi/293F	RV144_wk26	100 (16/16)	6167	100 (15/15)	7900	100 (17/17)	6176	100 (9/9)	10133
IgG	CRF07_BC	gp120	CNE20_D11gp120.avi/293F	RV305_wk0	29.4 (5/17)	158	44.4 (8/18)	154	21.1 (4/19)	209	16.7 (2/12)	140
IgG	CRF07_BC	gp120	CNE20_D11gp120.avi/293F	RV305_wk2	100 (17/17)	27134	100 (18/18)	27110	57.9 (11/19)	272	16.7 (2/12)	126
IgG	CRF07_BC	gp120	CNE20_D11gp120.avi/293F	RV305_wk24	94.4 (17/18)	4119	100 (18/18)	4284	57.9 (11/19)	247	16.7 (2/12)	129
IgG	CRF07_BC	gp120	CNE20_D11gp120.avi/293F	RV305_wk26	100 (18/18)	15972	100 (18/18)	16250	68.4 (13/19)	352	16.7 (2/12)	137
IgG	CRF07_BC	gp120	CNE20_D11gp120.avi/293F	RV305_wk48	100 (17/17)	3892	100 (18/18)	4774	63.2 (12/19)	297	16.7 (2/12)	125
IgG	CRF07_BC	gp120	CNE20_D11gp120.avi/293F	RV305_wk72	94.4 (17/18)	3811	100 (18/18)	3253	55.6 (10/18)	214	16.7 (2/12)	117

S1 Table continued

					Group 1: Combination		Group 2: AIDSVAX B/E		Group 3: ALVAC-HIV		RV305_Placebo Group	
Isotype	Clade	Env Region	Antigen	Study Week	Response Rate *(Responders/ Total)*	Median MFI *Responders*	Response Rate *(Responders/ Total)*	Median MFI *Responders*	Response Rate *(Responders/ Total)*	Median MFI *Responders*	Response Rate *(Responders/ Total)*	Median MFI *Responders*
IgG	Consensus	gp120	Con 6 gp120/B	RV144_wk26	93.8 (15/16)	4001	100 (15/15)	6913	100 (17/17)	3559	100 (9/9)	5424
IgG	Consensus	gp120	Con 6 gp120/B	RV305_wk0	0.0 (0/17)		16.7 (3/18)	155	10.5 (2/19)	161	0.0 (0/12)	
IgG	Consensus	gp120	Con 6 gp120/B	RV305_wk2	100 (17/17)	27634	100 (18/18)	26608	47.4 (9/19)	200	0.0 (0/12)	
IgG	Consensus	gp120	Con 6 gp120/B	RV305_wk24	88.9 (16/18)	3211	100 (18/18)	2967	31.6 (6/19)	200	0.0 (0/12)	
IgG	Consensus	gp120	Con 6 gp120/B	RV305_wk26	100 (18/18)	13836	100 (18/18)	16660	57.9 (11/19)	248	0.0 (0/12)	
IgG	Consensus	gp120	Con 6 gp120/B	RV305_wk48	100 (17/17)	2816	100 (18/18)	3659	50.0 (9/18)	234	0.0 (0/12)	
IgG	Consensus	gp120	Con 6 gp120/B	RV305_wk72	94.4 (17/18)	2835	100 (18/18)	2350	38.9 (7/18)	157	0.0 (0/12)	
IgG	A	gp140	9004S.gp140C.avi	RV144_wk26	87.5 (14/16)	568	100 (15/15)	607	94.1 (16/17)	589	100 (9/9)	722
IgG	A	gp140	9004S.gp140C.avi	RV305_wk0	0.0 (0/17)		0.0 (0/18)		0.0 (0/19)		0.0 (0/12)	
IgG	A	gp140	9004S.gp140C.avi	RV305_wk2	100 (17/17)	5544	100 (18/18)	5665	5.3 (1/19)	108	0.0 (0/12)	
IgG	A	gp140	9004S.gp140C.avi	RV305_wk24	88.9 (16/18)	388	94.4 (17/18)	425	0.0 (0/19)		0.0 (0/12)	
IgG	A	gp140	9004S.gp140C.avi	RV305_wk26	94.4 (17/18)	2017	100 (18/18)	1862	5.3 (1/19)	104	0.0 (0/12)	
IgG	A	gp140	9004S.gp140C.avi	RV305_wk48	94.1 (16/17)	428	94.4 (17/18)	509	0.0 (0/19)		0.0 (0/12)	
IgG	A	gp140	9004S.gp140C.avi	RV305_wk72	77.8 (14/18)	337	88.9 (16/18)	336	0.0 (0/18)		0.0 (0/12)	
IgG	B	gp140	RHPA4259_C7.gp140C.avi	RV144_wk26	100 (16/16)	3026	100 (15/15)	3166	100 (17/17)	2732	100 (9/9)	3383
IgG	B	gp140	RHPA4259_C7.gp140C.avi	RV305_wk0	0.0 (0/17)		5.6 (1/18)	140	5.3 (1/19)	109	0.0 (0/12)	
IgG	B	gp140	RHPA4259_C7.gp140C.avi	RV305_wk2	100 (17/17)	25240	100 (18/18)	26364	10.5 (2/19)	146	0.0 (0/12)	
IgG	B	gp140	RHPA4259_C7.gp140C.avi	RV305_wk24	94.4 (17/18)	1570	100 (18/18)	1664	15.8 (3/19)	138	0.0 (0/12)	
IgG	B	gp140	RHPA4259_C7.gp140C.avi	RV305_wk26	100 (18/18)	11196	100 (18/18)	12142	21.1 (4/19)	147	0.0 (0/12)	
IgG	B	gp140	RHPA4259_C7.gp140C.avi	RV305_wk48	100 (17/17)	2098	100 (18/18)	2410	15.8 (3/19)	193	0.0 (0/12)	
IgG	B	gp140	RHPA4259_C7.gp140C.avi	RV305_wk72	94.4 (17/18)	1386	94.4 (17/18)	1459	11.1 (2/18)	157	0.0 (0/12)	
IgG	B	gp140	SC42261_gp140.avi/293F	RV144_wk26	100 (16/16)	4467	100 (15/15)	4930	100 (17/17)	4176	100 (9/9)	5313
IgG	B	gp140	SC42261_gp140.avi/293F	RV305_wk0	0.0 (0/17)		5.6 (1/18)	178	10.5 (2/19)	156	0.0 (0/12)	
IgG	B	gp140	SC42261_gp140.avi/293F	RV305_wk2	100 (17/17)	29102	100 (18/18)	29607	36.8 (7/19)	129	0.0 (0/12)	
IgG	B	gp140	SC42261_gp140.avi/293F	RV305_wk24	94.4 (17/18)	3951	100 (18/18)	3644	31.6 (6/19)	167	0.0 (0/12)	
IgG	B	gp140	SC42261_gp140.avi/293F	RV305_wk26	100 (18/18)	17453	100 (18/18)	19077	47.4 (9/19)	154	0.0 (0/12)	
IgG	B	gp140	SC42261_gp140.avi/293F	RV305_wk48	100 (17/17)	4347	100 (18/18)	3835	36.8 (7/19)	185	0.0 (0/12)	
IgG	B	gp140	SC42261_gp140.avi/293F	RV305_wk72	94.4 (17/18)	2967	100 (18/18)	2537	33.3 (6/18)	124	0.0 (0/12)	
IgG	B	gp140	WITO4160.gp140C.avi	RV144_wk26	87.5 (14/16)	1539	100 (15/15)	1634	100 (17/17)	1340	100 (9/9)	1675
IgG	B	gp140	WITO4160.gp140C.avi	RV305_wk0	0.0 (0/17)		0.0 (0/18)		0.0 (0/19)		0.0 (0/12)	
IgG	B	gp140	WITO4160.gp140C.avi	RV305_wk2	100 (17/17)	22958	100 (18/18)	22458	0.0 (0/19)		0.0 (0/12)	
IgG	B	gp140	WITO4160.gp140C.avi	RV305_wk24	94.4 (17/18)	825	94.4 (17/18)	975	0.0 (0/19)		0.0 (0/12)	
IgG	B	gp140	WITO4160.gp140C.avi	RV305_wk26	100 (18/18)	8042	100 (18/18)	8037	5.3 (1/19)	127	0.0 (0/12)	
IgG	B	gp140	WITO4160.gp140C.avi	RV305_wk48	100 (17/17)	1242	94.4 (17/18)	1370	5.3 (1/19)	116	0.0 (0/12)	
IgG	B	gp140	WITO4160.gp140C.avi	RV305_wk72	94.4 (17/18)	767	94.4 (17/18)	826	0.0 (0/18)		0.0 (0/12)	

					Group 1: Combination		Group 2: AIDSVAX B/E		Group 3: ALVAC-HIV		RV305_Placebo Group	
Isotype	Clade	Env Region	Antigen	Study Week	Response Rate *(Responders/ Total)*	Median MFI *Responders*	Response Rate *(Responders/ Total)*	Median MFI *Responders*	Response Rate *(Responders/ Total)*	Median MFI *Responders*	Response Rate *(Responders/ Total)*	Median MFI *Responders*
IgG	C	gp140	1086C gp140C_avi	RV144_wk26	100 (16/16)	26768	100 (15/15)	26285	100 (17/17)	27586	100 (9/9)	27750
IgG	C	gp140	1086C gp140C_avi	RV305_wk0	58.8 (10/17)	235	50.0 (9/18)	296	63.2 (12/19)	220	25.0 (3/12)	372
IgG	C	gp140	1086C gp140C_avi	RV305_wk2	100 (17/17)	31958	100 (18/18)	31784	78.9 (15/19)	587	25.0 (3/12)	323
IgG	C	gp140	1086C gp140C_avi	RV305_wk24	94.4 (17/18)	21005	100 (18/18)	21089	73.7 (14/19)	549	25.0 (3/12)	352
IgG	C	gp140	1086C gp140C_avi	RV305_wk26	100 (18/18)	29551	100 (18/18)	30773	89.5 (17/19)	878	33.3 (4/12)	272
IgG	C	gp140	1086C gp140C_avi	RV305_wk48	100 (17/17)	23500	100 (18/18)	22833	89.5 (17/19)	465	33.3 (4/12)	245
IgG	C	gp140	1086C gp140C_avi	RV305_wk72	100 (18/18)	16273	100 (18/18)	16107	88.9 (16/18)	403	33.3 (4/12)	242
IgG	C	gp140	BF1266_gp140C.avi/293F	RV144_wk26	93.8 (15/16)	2913	100 (15/15)	2635	100 (17/17)	2888	100 (9/9)	3690
IgG	C	gp140	BF1266_gp140C.avi/293F	RV305_wk0	5.9 (1/17)	143	5.6 (1/18)	182	0.0 (0/19)		0.0 (0/12)	
IgG	C	gp140	BF1266_gp140C.avi/293F	RV305_wk2	100 (17/17)	26395	100 (18/18)	26516	36.8 (7/19)	253	0.0 (0/12)	
IgG	C	gp140	BF1266_gp140C.avi/293F	RV305_wk24	94.4 (17/18)	2121	100 (18/18)	2228	31.6 (6/19)	157	0.0 (0/12)	
IgG	C	gp140	BF1266_gp140C.avi/293F	RV305_wk26	100 (18/18)	10797	100 (18/18)	13110	63.2 (12/19)	234	0.0 (0/12)	
IgG	C	gp140	BF1266_gp140C.avi/293F	RV305_wk48	100 (17/17)	2687	100 (18/18)	2394	42.1 (8/19)	237	0.0 (0/12)	
IgG	C	gp140	BF1266_gp140C.avi/293F	RV305_wk72	94.4 (17/18)	1788	100 (18/18)	1476	33.3 (6/18)	173	0.0 (0/12)	
IgG	C	gp140	C.CH505TF_gp140/293F	RV144_wk26	93.8 (15/16)	2076	100 (15/15)	2422	100 (17/17)	2464	100 (9/9)	2949
IgG	C	gp140	C.CH505TF_gp140/293F	RV305_wk0	0.0 (0/17)		0.0 (0/18)		0.0 (0/19)		0.0 (0/12)	
IgG	C	gp140	C.CH505TF_gp140/293F	RV305_wk2	100 (17/17)	24712	100 (18/18)	25670	31.6 (6/19)	213	0.0 (0/12)	
IgG	C	gp140	C.CH505TF_gp140/293F	RV305_wk24	94.4 (17/18)	1483	100 (18/18)	1721	21.1 (4/19)	160	0.0 (0/12)	
IgG	C	gp140	C.CH505TF_gp140/293F	RV305_wk26	100 (18/18)	9330	100 (18/18)	9856	57.9 (11/19)	157	0.0 (0/12)	
IgG	C	gp140	C.CH505TF_gp140/293F	RV305_wk48	100 (17/17)	2020	100 (18/18)	2041	36.8 (7/19)	197	0.0 (0/12)	
IgG	C	gp140	C.CH505TF_gp140/293F	RV305_wk72	94.4 (17/18)	1671	100 (18/18)	1345	27.8 (5/18)	113	0.0 (0/12)	
IgG	Consensus	gp140	Con S gp140 CFI	RV144_wk26	100 (16/16)	6658	100 (15/15)	7256	100 (17/17)	7438	100 (9/9)	8346
IgG	Consensus	gp140	Con S gp140 CFI	RV305_wk0	0.0 (0/17)		22.2 (4/18)	139	10.5 (2/19)	123	0.0 (0/12)	
IgG	Consensus	gp140	Con S gp140 CFI	RV305_wk2	100 (17/17)	30967	100 (18/18)	29915	36.8 (7/19)	287	0.0 (0/12)	
IgG	Consensus	gp140	Con S gp140 CFI	RV305_wk24	94.4 (17/18)	4656	100 (18/18)	3211	31.6 (6/19)	215	0.0 (0/12)	
IgG	Consensus	gp140	Con S gp140 CFI	RV305_wk26	100 (18/18)	24378	100 (18/18)	24286	68.4 (13/19)	269	0.0 (0/12)	
IgG	Consensus	gp140	Con S gp140 CFI	RV305_wk48	100 (17/17)	5150	100 (18/18)	3698	47.4 (9/19)	253	0.0 (0/12)	
IgG	Consensus	gp140	Con S gp140 CFI	RV305_wk72	94.4 (17/18)	4372	100 (18/18)	2295	33.3 (6/18)	187	0.0 (0/12)	

S1 Table continued

					Group 1: Combination		Group 2: AIDSVAX B/E		Group 3: ALVAC-HIV		RV305_Placebo Group	
Isotype	Clade	Env Region	Antigen	Study Week	Response Rate *(Responders/ Total)*	Median MFI *Responders*	Response Rate *(Responders/ Total)*	Median MFI *Responders*	Response Rate *(Responders/ Total)*	Median MFI *Responders*	Response Rate *(Responders/ Total)*	Median MFI *Responders*
IgG	Consensus CRF01_AE	gp140	AE.01.con_env03 gp140CF_avi	RV144_wk26	93.8 (15/16)	4519	100 (15/15)	5337	100 (17/17)	4014	100 (9/9)	5019
IgG	Consensus CRF01_AE	gp140	AE.01.con_env03 gp140CF_avi	RV305_wk0	0.0 (0/17)		5.6 (1/18)	179	0.0 (0/19)		0.0 (0/12)	
IgG	Consensus CRF01_AE	gp140	AE.01.con_env03 gp140CF_avi	RV305_wk2	100 (17/17)	28704	100 (18/18)	28432	36.8 (7/19)	258	0.0 (0/12)	
IgG	Consensus CRF01_AE	gp140	AE.01.con_env03 gp140CF_avi	RV305_wk24	88.9 (16/18)	1793	100 (18/18)	1540	31.6 (6/19)	182	0.0 (0/12)	
IgG	Consensus CRF01_AE	gp140	AE.01.con_env03 gp140CF_avi	RV305_wk26	100 (18/18)	15658	100 (18/18)	15659	68.4 (13/19)	371	0.0 (0/12)	
IgG	Consensus CRF01_AE	gp140	AE.01.con_env03 gp140CF_avi	RV305_wk48	100 (17/17)	2431	100 (18/18)	2083	47.4 (9/19)	390	0.0 (0/12)	
IgG	Consensus CRF01_AE	gp140	AE.01.con_env03 gp140CF_avi	RV305_wk72	94.4 (17/18)	1722	100 (18/18)	1091	38.9 (7/18)	215	0.0 (0/12)	
IgG	A	V1V2	gp70-191084_B7 V1V2	RV144_wk26	93.8 (15/16)	15762	100 (15/15)	17071	100 (17/17)	13666	100 (9/9)	24718
IgG	A	V1V2	gp70-191084_B7 V1V2	RV305_wk0	5.9 (1/17)	903	0.0 (0/18)		10.5 (2/19)	177	0.0 (0/12)	
IgG	A	V1V2	gp70-191084_B7 V1V2	RV305_wk2	100 (17/17)	30612	100 (18/18)	30769	89.5 (17/19)	562	0.0 (0/12)	
IgG	A	V1V2	gp70-191084_B7 V1V2	RV305_wk24	94.4 (17/18)	2747	94.4 (17/18)	3849	52.6 (10/19)	338	0.0 (0/12)	
IgG	A	V1V2	gp70-191084_B7 V1V2	RV305_wk26	100 (18/18)	26786	100 (18/18)	27022	100 (19/19)	635	0.0 (0/12)	
IgG	A	V1V2	gp70-191084_B7 V1V2	RV305_wk48	100 (17/17)	3419	100 (18/18)	2712	89.5 (17/19)	344	0.0 (0/12)	
IgG	A	V1V2	gp70-191084_B7 V1V2	RV305_wk72	88.9 (16/18)	1990	100 (18/18)	1347	66.7 (12/18)	235	0.0 (0/12)	
IgG	B	V1V2	gp70-62357.14 V1V2	RV144_wk26	68.8 (11/16)	810	86.7 (13/15)	517	82.4 (14/17)	340	88.9 (8/9)	655
IgG	B	V1V2	gp70-62357.14 V1V2	RV305_wk0	0.0 (0/17)		0.0 (0/18)		0.0 (0/19)		0.0 (0/12)	
IgG	B	V1V2	gp70-62357.14 V1V2	RV305_wk2	82.4 (14/17)	1535	88.9 (16/18)	747	0.0 (0/19)		0.0 (0/12)	
IgG	B	V1V2	gp70-62357.14 V1V2	RV305_wk24	5.6 (1/18)	104	16.7 (3/18)	299	0.0 (0/19)		0.0 (0/12)	
IgG	B	V1V2	gp70-62357.14 V1V2	RV305_wk26	77.8 (14/18)	771	72.2 (13/18)	687	0.0 (0/19)		0.0 (0/12)	
IgG	B	V1V2	gp70-62357.14 V1V2	RV305_wk48	5.9 (1/17)	113	16.7 (3/18)	276	0.0 (0/19)		0.0 (0/12)	
IgG	B	V1V2	gp70-62357.14 V1V2	RV305_wk72	0.0 (0/18)		16.7 (3/18)	176	0.0 (0/18)		0.0 (0/12)	
IgG	B	V1V2	gp70-700010058 V1V2	RV144_wk26	37.5 (6/16)	545	60.0 (9/15)	292	58.8 (10/17)	703	44.4 (4/9)	571
IgG	B	V1V2	gp70-700010058 V1V2	RV305_wk0	0.0 (0/17)		0.0 (0/18)		5.3 (1/19)	180	0.0 (0/12)	
IgG	B	V1V2	gp70-700010058 V1V2	RV305_wk2	94.1 (16/17)	8278	100 (18/18)	7156	5.3 (1/19)	192	0.0 (0/12)	
IgG	B	V1V2	gp70-700010058 V1V2	RV305_wk24	22.2 (4/18)	589	27.8 (5/18)	236	5.3 (1/19)	240	0.0 (0/12)	
IgG	B	V1V2	gp70-700010058 V1V2	RV305_wk26	94.4 (17/18)	3402	100 (18/18)	7252	5.3 (1/19)	191	0.0 (0/12)	
IgG	B	V1V2	gp70-700010058 V1V2	RV305_wk48	35.3 (6/17)	586	61.1 (11/18)	198	10.5 (2/19)	177	0.0 (0/12)	
IgG	B	V1V2	gp70-700010058 V1V2	RV305_wk72	27.8 (5/18)	502	38.9 (7/18)	199	5.6 (1/18)	241	0.0 (0/12)	

S1 Table continued

					Group 1: Combination		Group 2: AIDSVAX B/E		Group 3: ALVAC-HIV		RV305_Placebo Group	
Isotype	Clade	Env Region	Antigen	Study Week	Response Rate *(Responders/ Total)*	Median MFI *Responders*	Response Rate *(Responders/ Total)*	Median MFI *Responders*	Response Rate *(Responders/ Total)*	Median MFI *Responders*	Response Rate *(Responders/ Total)*	Median MFI *Responders*
IgG	B	V1V2	gp70-RHPA4259.7 V1V2	RV144_wk26	75.0 (12/16)	1582	93.3 (14/15)	854	94.1 (16/17)	860	88.9 (8/9)	939
IgG	B	V1V2	gp70-RHPA4259.7 V1V2	RV305_wk0	0.0 (0/17)		0.0 (0/18)		0.0 (0/19)		0.0 (0/12)	
IgG	B	V1V2	gp70-RHPA4259.7 V1V2	RV305_wk2	82.4 (14/17)	4192	94.4 (17/18)	764	5.3 (1/19)	106	0.0 (0/12)	
IgG	B	V1V2	gp70-RHPA4259.7 V1V2	RV305_wk24	0.0 (0/18)		16.7 (3/18)	286	5.3 (1/19)	149	0.0 (0/12)	
IgG	B	V1V2	gp70-RHPA4259.7 V1V2	RV305_wk26	83.3 (15/18)	884	88.9 (16/18)	844	5.3 (1/19)	124	0.0 (0/12)	
IgG	B	V1V2	gp70-RHPA4259.7 V1V2	RV305_wk48	0.0 (0/17)		16.7 (3/18)	326	5.3 (1/19)	139	0.0 (0/12)	
IgG	B	V1V2	gp70-RHPA4259.7 V1V2	RV305_wk72	11.1 (2/18)	5309	11.1 (2/18)	339	5.6 (1/18)	139	0.0 (0/12)	
IgG	B	V1V2	gp70-TT31P.2F10.2792 V1V2	RV144_wk26	68.8 (11/16)	1086	93.3 (14/15)	569	88.2 (15/17)	360	100 (9/9)	480
IgG	B	V1V2	gp70-TT31P.2F10.2792 V1V2	RV305_wk0	0.0 (0/17)		0.0 (0/18)		0.0 (0/19)		0.0 (0/12)	
IgG	B	V1V2	gp70-TT31P.2F10.2792 V1V2	RV305_wk2	88.2 (15/17)	2801	88.9 (16/18)	1434	0.0 (0/19)		0.0 (0/12)	
IgG	B	V1V2	gp70-TT31P.2F10.2792 V1V2	RV305_wk24	16.7 (3/18)	146	16.7 (3/18)	354	5.3 (1/19)	113	0.0 (0/12)	
IgG	B	V1V2	gp70-TT31P.2F10.2792 V1V2	RV305_wk26	83.3 (15/18)	902	83.3 (15/18)	946	0.0 (0/19)		0.0 (0/12)	
IgG	B	V1V2	gp70-TT31P.2F10.2792 V1V2	RV305_wk48	11.8 (2/17)	288	27.8 (5/18)	218	5.3 (1/19)	102	0.0 (0/12)	
IgG	B	V1V2	gp70-TT31P.2F10.2792 V1V2	RV305_wk72	16.7 (3/18)	130	16.7 (3/18)	304	5.6 (1/18)	101	0.0 (0/12)	
IgG	B	V1V2	gp70_B.CaseA2 V1/V2/169K	RV144_wk26	75.0 (12/16)	2551	93.3 (14/15)	1000	88.2 (15/17)	1434	100 (9/9)	816
IgG	B	V1V2	gp70_B.CaseA2 V1/V2/169K	RV305_wk0	5.9 (1/17)	234	0.0 (0/18)		0.0 (0/19)		0.0 (0/12)	
IgG	B	V1V2	gp70_B.CaseA2 V1/V2/169K	RV305_wk2	100 (17/17)	14893	94.4 (17/18)	11759	5.3 (1/19)	114	0.0 (0/12)	
IgG	B	V1V2	gp70_B.CaseA2 V1/V2/169K	RV305_wk24	38.9 (7/18)	241	38.9 (7/18)	238	0.0 (0/19)		0.0 (0/12)	
IgG	B	V1V2	gp70_B.CaseA2 V1/V2/169K	RV305_wk26	88.9 (16/18)	3547	94.4 (17/18)	2436	0.0 (0/19)		0.0 (0/12)	
IgG	B	V1V2	gp70_B.CaseA2 V1/V2/169K	RV305_wk48	47.1 (8/17)	404	38.9 (7/18)	397	0.0 (0/19)		0.0 (0/12)	
IgG	B	V1V2	gp70_B.CaseA2 V1/V2/169K	RV305_wk72	33.3 (6/18)	651	27.8 (5/18)	925	0.0 (0/18)		0.0 (0/12)	
IgG	B	V1V2	gp70_B.CaseA_V1_V2	RV144_wk26	75.0 (12/16)	2060	86.7 (13/15)	957	88.2 (15/17)	713	100 (9/9)	856
IgG	B	V1V2	gp70_B.CaseA_V1_V2	RV305_wk0	5.9 (1/17)	483	0.0 (0/18)		5.3 (1/19)	228	0.0 (0/12)	
IgG	B	V1V2	gp70_B.CaseA_V1_V2	RV305_wk2	100 (17/17)	8695	94.4 (17/18)	3045	5.3 (1/19)	314	0.0 (0/12)	
IgG	B	V1V2	gp70_B.CaseA_V1_V2	RV305_wk24	44.4 (8/18)	149	22.2 (4/18)	530	0.0 (0/19)		0.0 (0/12)	
IgG	B	V1V2	gp70_B.CaseA_V1_V2	RV305_wk26	88.9 (16/18)	2495	94.4 (17/18)	2513	5.3 (1/19)	349	0.0 (0/12)	
IgG	B	V1V2	gp70_B.CaseA_V1_V2	RV305_wk48	52.9 (9/17)	219	33.3 (6/18)	216	5.3 (1/19)	375	0.0 (0/12)	
IgG	B	V1V2	gp70_B.CaseA_V1_V2	RV305_wk72	41.2 (7/17)	279	16.7 (3/18)	636	5.6 (1/18)	393	0.0 (0/12)	
IgG	C	V1V2	C.1086C_V1_V2 Tags	RV144_wk26	93.8 (15/16)	9909	100 (15/15)	7366	100 (17/17)	9967	100 (9/9)	8938
IgG	C	V1V2	C.1086C_V1_V2 Tags	RV305_wk0	5.9 (1/17)	271	0.0 (0/18)		5.3 (1/19)	120	0.0 (0/12)	
IgG	C	V1V2	C.1086C_V1_V2 Tags	RV305_wk2	100 (17/17)	27702	100 (18/18)	24278	47.4 (9/19)	241	0.0 (0/12)	
IgG	C	V1V2	C.1086C_V1_V2 Tags	RV305_wk24	83.3 (15/18)	932	83.3 (15/18)	884	15.8 (3/19)	190	0.0 (0/12)	
IgG	C	V1V2	C.1086C_V1_V2 Tags	RV305_wk26	100 (18/18)	12150	100 (18/18)	8502	77.8 (14/18)	231	0.0 (0/12)	
IgG	C	V1V2	C.1086C_V1_V2 Tags	RV305_wk48	88.2 (15/17)	1027	94.4 (17/18)	980	47.4 (9/19)	172	0.0 (0/12)	
IgG	C	V1V2	C.1086C_V1_V2 Tags	RV305_wk72	72.2 (13/18)	651	77.8 (14/18)	564	22.2 (4/18)	196	0.0 (0/12)	

S1 Table continued

					Group 1: Combination		Group 2: AIDSVAX B/E		Group 3: ALVAC-HIV		RV305_Placebo Group	
Isotype	Clade	Env Region	Antigen	Study Week	Response Rate *(Responders/ Total)*	Median MFI *Responders*	Response Rate *(Responders/ Total)*	Median MFI *Responders*	Response Rate *(Responders/ Total)*	Median MFI *Responders*	Response Rate *(Responders/ Total)*	Median MFI *Responders*
IgG	C	V1V2	gp70-001428.2.42 V1V2	RV144_wk26	81.3 (13/16)	1266	93.3 (14/15)	767	94.1 (16/17)	932	100 (9/9)	650
IgG	C	V1V2	gp70-001428.2.42 V1V2	RV305_wk0	0.0 (0/17)		0.0 (0/18)		5.3 (1/19)	156	0.0 (0/12)	
IgG	C	V1V2	gp70-001428.2.42 V1V2	RV305_wk2	100 (17/17)	12117	100 (18/18)	14937	15.8 (3/19)	161	0.0 (0/12)	
IgG	C	V1V2	gp70-001428.2.42 V1V2	RV305_wk24	44.4 (8/18)	292	50.0 (9/18)	242	10.5 (2/19)	220	0.0 (0/12)	
IgG	C	V1V2	gp70-001428.2.42 V1V2	RV305_wk26	100 (18/18)	3054	100 (18/18)	3637	21.1 (4/19)	394	0.0 (0/12)	
IgG	C	V1V2	gp70-001428.2.42 V1V2	RV305_wk48	58.8 (10/17)	224	61.1 (11/18)	264	21.1 (4/19)	300	0.0 (0/12)	
IgG	C	V1V2	gp70-001428.2.42 V1V2	RV305_wk72	44.4 (8/18)	187	33.3 (6/18)	294	16.7 (3/18)	242	0.0 (0/12)	
IgG	C	V1V2	gp70-7060101641 V1V2	RV144_wk26	87.5 (14/16)	1888	93.3 (14/15)	1466	94.1 (16/17)	856	100 (9/9)	1479
IgG	C	V1V2	gp70-7060101641 V1V2	RV305_wk0	0.0 (0/17)		0.0 (0/18)		0.0 (0/19)		0.0 (0/12)	
IgG	C	V1V2	gp70-7060101641 V1V2	RV305_wk2	100 (17/17)	23957	94.4 (17/18)	22614	26.3 (5/19)	266	0.0 (0/12)	
IgG	C	V1V2	gp70-7060101641 V1V2	RV305_wk24	66.7 (12/18)	425	66.7 (12/18)	432	10.5 (2/19)	142	0.0 (0/12)	
IgG	C	V1V2	gp70-7060101641 V1V2	RV305_wk26	100 (18/18)	6526	100 (18/18)	6470	42.1 (8/19)	311	0.0 (0/12)	
IgG	C	V1V2	gp70-7060101641 V1V2	RV305_wk48	76.5 (13/17)	542	77.8 (14/18)	594	26.3 (5/19)	205	0.0 (0/12)	
IgG	C	V1V2	gp70-7060101641 V1V2	RV305_wk72	55.6 (10/18)	440	66.7 (12/18)	312	5.6 (1/18)	1193	0.0 (0/12)	
IgG	C	V1V2	gp70-96ZM651.02 V1v2	RV144_wk26	56.3 (9/16)	5793	60.0 (9/15)	9426	47.1 (8/17)	14582	66.7 (6/9)	5154
IgG	C	V1V2	gp70-96ZM651.02 V1v2	RV305_wk0	0.0 (0/17)		0.0 (0/18)		0.0 (0/18)		0.0 (0/12)	
IgG	C	V1V2	gp70-96ZM651.02 V1v2	RV305_wk2	100 (17/17)	27885	100 (18/18)	29665	0.0 (0/19)		0.0 (0/12)	
IgG	C	V1V2	gp70-96ZM651.02 V1v2	RV305_wk24	5.9 (1/17)	15948	16.7 (3/18)	16562	0.0 (0/19)		0.0 (0/12)	
IgG	C	V1V2	gp70-96ZM651.02 V1v2	RV305_wk26	88.9 (16/18)	9104	88.2 (15/17)	17546	10.5 (2/19)	3677	0.0 (0/12)	
IgG	C	V1V2	gp70-96ZM651.02 V1v2	RV305_wk48	11.8 (2/17)	13075	16.7 (3/18)	15537	5.3 (1/19)	4176	0.0 (0/12)	
IgG	C	V1V2	gp70-96ZM651.02 V1v2	RV305_wk72	11.1 (2/18)	5306	11.1 (2/18)	21388	5.6 (1/18)	2332	0.0 (0/12)	
IgG	C	V1V2	gp70-BF1266_431a_V1V2	RV144_wk26	68.8 (11/16)	1111	93.3 (14/15)	657	88.2 (15/17)	428	88.9 (8/9)	781
IgG	C	V1V2	gp70-BF1266_431a_V1V2	RV305_wk0	0.0 (0/17)		0.0 (0/18)		5.3 (1/19)	1210	0.0 (0/12)	
IgG	C	V1V2	gp70-BF1266_431a_V1V2	RV305_wk2	88.2 (15/17)	4002	83.3 (15/18)	10519	10.5 (2/19)	613	0.0 (0/12)	
IgG	C	V1V2	gp70-BF1266_431a_V1V2	RV305_wk24	16.7 (3/18)	139	22.2 (4/18)	427	5.3 (1/19)	1013	0.0 (0/12)	
IgG	C	V1V2	gp70-BF1266_431a_V1V2	RV305_wk26	83.3 (15/18)	887	83.3 (15/18)	1349	10.5 (2/19)	592	0.0 (0/12)	
IgG	C	V1V2	gp70-BF1266_431a_V1V2	RV305_wk48	11.8 (2/17)	376	27.8 (5/18)	443	5.3 (1/19)	1094	0.0 (0/12)	
IgG	C	V1V2	gp70-BF1266_431a_V1V2	RV305_wk72	11.1 (2/18)	128	16.7 (3/18)	319	5.6 (1/18)	741	0.0 (0/12)	
IgG	C	V1V2	gp70-CAP210.2.00.E8 V1V2	RV144_wk26	62.5 (10/16)	464	66.7 (10/15)	256	47.1 (8/17)	643	66.7 (6/9)	313
IgG	C	V1V2	gp70-CAP210.2.00.E8 V1V2	RV305_wk0	0.0 (0/17)		0.0 (0/18)		5.3 (1/19)	193	0.0 (0/12)	
IgG	C	V1V2	gp70-CAP210.2.00.E8 V1V2	RV305_wk2	88.2 (15/17)	1282	94.4 (17/18)	2447	5.3 (1/19)	198	0.0 (0/12)	
IgG	C	V1V2	gp70-CAP210.2.00.E8 V1V2	RV305_wk24	5.6 (1/18)	138	16.7 (3/18)	189	5.3 (1/19)	248	0.0 (0/12)	
IgG	C	V1V2	gp70-CAP210.2.00.E8 V1V2	RV305_wk26	66.7 (12/18)	524	88.9 (16/18)	471	5.3 (1/19)	204	0.0 (0/12)	
IgG	C	V1V2	gp70-CAP210.2.00.E8 V1V2	RV305_wk48	11.8 (2/17)	105	16.7 (3/18)	196	5.3 (1/19)	252	0.0 (0/12)	
IgG	C	V1V2	gp70-CAP210.2.00.E8 V1V2	RV305_wk72	0.0 (0/18)		16.7 (3/18)	167	5.6 (1/18)	276	0.0 (0/12)	

S1 Table continued

					Group 1: Combination		Group 2: AIDSVAX B/E		Group 3: ALVAC-HIV		RV305_Placebo Group	
Isotype	Clade	Env Region	Antigen	Study Week	Response Rate *(Responders/ Total)*	Median MFI *Responders*	Response Rate *(Responders/ Total)*	Median MFI *Responders*	Response Rate *(Responders/ Total)*	Median MFI *Responders*	Response Rate *(Responders/ Total)*	Median MFI *Responders*
IgG	C	V1V2	gp70-Ce1086_B2 V1V2	RV144_wk26	100 (16/16)	25367	100 (15/15)	26916	100 (16/16)	25778	100 (9/9)	27375
IgG	C	V1V2	gp70-Ce1086_B2 V1V2	RV305_wk0	11.8 (2/17)	809	0.0 (0/18)		5.3 (1/19)	172	8.3 (1/12)	156
IgG	C	V1V2	gp70-Ce1086_B2 V1V2	RV305_wk2	100 (17/17)	31437	100 (18/18)	30199	88.9 (16/18)	715	8.3 (1/12)	152
IgG	C	V1V2	gp70-Ce1086_B2 V1V2	RV305_wk24	100 (18/18)	3711	100 (18/18)	4353	63.2 (12/19)	319	8.3 (1/12)	153
IgG	C	V1V2	gp70-Ce1086_B2 V1V2	RV305_wk26	100 (18/18)	27168	100 (18/18)	27105	94.4 (17/18)	1112	8.3 (1/12)	122
IgG	C	V1V2	gp70-Ce1086_B2 V1V2	RV305_wk48	100 (17/17)	5238	100 (18/18)	4194	84.2 (16/19)	766	0.0 (0/12)	
IgG	C	V1V2	gp70-Ce1086_B2 V1V2	RV305_wk72	88.9 (16/18)	3234	100 (18/18)	2116	83.3 (15/18)	430	0.0 (0/12)	
IgG	C	V1V2	gp70-TV1.21 V1V2	RV144_wk26	75.0 (12/16)	1904	93.3 (14/15)	964	88.2 (15/17)	789	100 (9/9)	804
IgG	C	V1V2	gp70-TV1.21 V1V2	RV305_wk0	0.0 (0/17)		0.0 (0/18)		5.3 (1/19)	222	0.0 (0/12)	
IgG	C	V1V2	gp70-TV1.21 V1V2	RV305_wk2	94.1 (16/17)	3497	94.4 (17/18)	5014	5.3 (1/19)	238	0.0 (0/12)	
IgG	C	V1V2	gp70-TV1.21 V1V2	RV305_wk24	27.8 (5/18)	154	16.7 (3/18)	604	5.3 (1/19)	249	0.0 (0/12)	
IgG	C	V1V2	gp70-TV1.21 V1V2	RV305_wk26	88.9 (16/18)	1075	94.4 (17/18)	1076	5.3 (1/19)	241	0.0 (0/12)	
IgG	C	V1V2	gp70-TV1.21 V1V2	RV305_wk48	23.5 (4/17)	229	38.9 (7/18)	177	5.3 (1/19)	291	0.0 (0/12)	
IgG	C	V1V2	gp70-TV1.21 V1V2	RV305_wk72	16.7 (3/18)	120	16.7 (3/18)	453	5.6 (1/18)	301	0.0 (0/12)	
IgG	CRF01_AE	V1V2	AE.A244 V1V2 tags	RV144_wk26	100 (16/16)	27031	100 (15/15)	26584	100 (16/16)	24876	100 (9/9)	26465
IgG	CRF01_AE	V1V2	AE.A244 V1V2 tags	RV305_wk0	5.9 (1/17)	255	0.0 (0/18)		0.0 (0/19)		16.7 (2/12)	172
IgG	CRF01_AE	V1V2	AE.A244 V1V2 tags	RV305_wk2	100 (17/17)	31299	100 (18/18)	30144	78.9 (15/19)	237	16.7 (2/12)	122
IgG	CRF01_AE	V1V2	AE.A244 V1V2 tags	RV305_wk24	88.9 (16/18)	2792	94.4 (17/18)	2323	52.6 (10/19)	229	8.3 (1/12)	124
IgG	CRF01_AE	V1V2	AE.A244 V1V2 tags	RV305_wk26	100 (18/18)	23865	100 (18/18)	24628	84.2 (16/19)	372	8.3 (1/12)	113
IgG	CRF01_AE	V1V2	AE.A244 V1V2 tags	RV305_wk48	100 (17/17)	1947	100 (18/18)	2600	78.9 (15/19)	218	8.3 (1/12)	134
IgG	CRF01_AE	V1V2	AE.A244 V1V2 tags	RV305_wk72	88.9 (16/18)	1140	100 (18/18)	1563	44.4 (8/18)	209	8.3 (1/12)	114
IgG	CRF01_AE	V1V2	gp70-C2101.c01_V1V2	RV144_wk26	93.8 (15/16)	6247	100 (15/15)	6365	82.4 (14/17)	4648	100 (9/9)	8421
IgG	CRF01_AE	V1V2	gp70-C2101.c01_V1V2	RV305_wk0	5.9 (1/17)	224	5.6 (1/18)	201	5.3 (1/19)	133	0.0 (0/12)	
IgG	CRF01_AE	V1V2	gp70-C2101.c01_V1V2	RV305_wk2	100 (17/17)	31258	100 (18/18)	29921	61.1 (11/18)	514	8.3 (1/12)	146
IgG	CRF01_AE	V1V2	gp70-C2101.c01_V1V2	RV305_wk24	94.4 (17/18)	1854	94.4 (17/18)	1347	36.8 (7/19)	197	8.3 (1/12)	164
IgG	CRF01_AE	V1V2	gp70-C2101.c01_V1V2	RV305_wk26	100 (18/18)	24748	100 (18/18)	27521	77.8 (14/18)	735	8.3 (1/12)	151
IgG	CRF01_AE	V1V2	gp70-C2101.c01_V1V2	RV305_wk48	100 (17/17)	1719	100 (18/18)	1725	68.4 (13/19)	386	0.0 (0/12)	
IgG	CRF01_AE	V1V2	gp70-C2101.c01_V1V2	RV305_wk72	83.3 (15/18)	1565	94.4 (17/18)	814	55.6 (10/18)	258	0.0 (0/12)	
IgG	CRF01_AE	V1V2	gp70-CM244.ec1 V1V2	RV144_wk26	100 (16/16)	29404	100 (15/15)	29574	100 (17/17)	28878	100 (9/9)	30831
IgG	CRF01_AE	V1V2	gp70-CM244.ec1 V1V2	RV305_wk0	5.9 (1/17)	2247	5.6 (1/18)	320	10.5 (2/19)	161	8.3 (1/12)	219
IgG	CRF01_AE	V1V2	gp70-CM244.ec1 V1V2	RV305_wk2	100 (17/17)	31934	100 (18/18)	31140	100 (18/18)	907	8.3 (1/12)	208
IgG	CRF01_AE	V1V2	gp70-CM244.ec1 V1V2	RV305_wk24	94.4 (17/18)	7019	100 (18/18)	7383	68.4 (13/19)	320	8.3 (1/12)	200
IgG	CRF01_AE	V1V2	gp70-CM244.ec1 V1V2	RV305_wk26	100 (18/18)	29177	100 (18/18)	29118	94.4 (17/18)	1530	8.3 (1/12)	167
IgG	CRF01_AE	V1V2	gp70-CM244.ec1 V1V2	RV305_wk48	100 (17/17)	9443	100 (18/18)	7624	89.5 (17/19)	1095	0.0 (0/12)	
IgG	CRF01_AE	V1V2	gp70-CM244.ec1 V1V2	RV305_wk72	94.4 (17/18)	6044	100 (18/18)	3847	83.3 (15/18)	586	0.0 (0/12)	

S1 Table continued

					Group 1: Combination		Group 2: AIDSVAX B/E		Group 3: ALVAC-HIV		RV305_Placebo Group	
Isotype	Clade	Env Region	Antigen	Study Week	Response Rate *(Responders/ Total)*	Median MFI *Responders*	Response Rate *(Responders/ Total)*	Median MFI *Responders*	Response Rate *(Responders/ Total)*	Median MFI *Responders*	Response Rate *(Responders/ Total)*	Median MFI *Responders*
IgG	CRF07_BC	V1V2	gp70-BJOX002000.03.2 V1V2	RV144_wk26	81.3 (13/16)	5025	93.3 (14/15)	3550	82.4 (14/17)	8541	88.9 (8/9)	4636
IgG	CRF07_BC	V1V2	gp70-BJOX002000.03.2 V1V2	RV305_wk0	5.9 (1/17)	1310	0.0 (0/18)		0.0 (0/19)		0.0 (0/12)	
IgG	CRF07_BC	V1V2	gp70-BJOX002000.03.2 V1V2	RV305_wk2	100 (17/17)	28047	100 (18/18)	29194	26.3 (5/19)	700	0.0 (0/12)	
IgG	CRF07_BC	V1V2	gp70-BJOX002000.03.2 V1V2	RV305_wk24	55.6 (10/18)	1423	66.7 (12/18)	1237	5.3 (1/19)	400	0.0 (0/12)	
IgG	CRF07_BC	V1V2	gp70-BJOX002000.03.2 V1V2	RV305_wk26	100 (18/18)	9156	100 (17/17)	18018	26.3 (5/19)	786	0.0 (0/12)	
IgG	CRF07_BC	V1V2	gp70-BJOX002000.03.2 V1V2	RV305_wk48	58.8 (10/17)	1276	83.3 (15/18)	848	15.8 (3/19)	715	0.0 (0/12)	
IgG	CRF07_BC	V1V2	gp70-BJOX002000.03.2 V1V2	RV305_wk72	50.0 (9/18)	1303	50.0 (9/18)	1081	11.1 (2/18)	960	0.0 (0/12)	
IgG	CRF01_AE	V2	AE.A244 V2 tags/293F	RV144_wk26	87.5 (14/16)	4573	100 (15/15)	5213	100 (17/17)	4660	100 (9/9)	6294
IgG	CRF01_AE	V2	AE.A244 V2 tags/293F	RV305_wk0	5.9 (1/17)	272	0.0 (0/18)		0.0 (0/19)		0.0 (0/12)	
IgG	CRF01_AE	V2	AE.A244 V2 tags/293F	RV305_wk2	100 (17/17)	25364	100 (18/18)	25638	57.9 (11/19)	282	0.0 (0/12)	
IgG	CRF01_AE	V2	AE.A244 V2 tags/293F	RV305_wk24	88.9 (16/18)	546	94.4 (17/18)	516	21.1 (4/19)	224	0.0 (0/12)	
IgG	CRF01_AE	V2	AE.A244 V2 tags/293F	RV305_wk26	100 (18/18)	7362	100 (18/18)	6267	78.9 (15/19)	285	0.0 (0/12)	
IgG	CRF01_AE	V2	AE.A244 V2 tags/293F	RV305_wk48	94.1 (16/17)	701	100 (18/18)	542	66.7 (12/18)	230	0.0 (0/12)	
IgG	CRF01_AE	V2	AE.A244 V2 tags/293F	RV305_wk72	70.6 (12/17)	719	83.3 (15/18)	480	38.9 (7/18)	296	0.0 (0/12)	
IgG	B	V3	B.MN V3 gp70	RV144_wk26	93.8 (15/16)	1775	93.3 (14/15)	2285	88.2 (15/17)	1727	88.9 (8/9)	539
IgG	B	V3	B.MN V3 gp70	RV305_wk0	5.9 (1/17)	108	5.6 (1/18)	138	0.0 (0/19)		0.0 (0/12)	
IgG	B	V3	B.MN V3 gp70	RV305_wk2	100 (17/17)	12631	100 (18/18)	12071	5.3 (1/19)	158	0.0 (0/12)	
IgG	B	V3	B.MN V3 gp70	RV305_wk24	72.2 (13/18)	1082	88.9 (16/18)	473	0.0 (0/19)		0.0 (0/12)	
IgG	B	V3	B.MN V3 gp70	RV305_wk26	100 (18/18)	4900	100 (18/18)	6555	5.3 (1/19)	165	0.0 (0/12)	
IgG	B	V3	B.MN V3 gp70	RV305_wk48	88.2 (15/17)	776	94.4 (17/18)	725	5.3 (1/19)	192	0.0 (0/12)	
IgG	B	V3	B.MN V3 gp70	RV305_wk72	66.7 (12/18)	952	88.9 (16/18)	503	5.6 (1/18)	187	0.0 (0/12)	
IgG	N/A	CD4i	HxB2 new 8b core 6x His	RV144_wk26	100 (16/16)	1973	100 (15/15)	2010	100 (17/17)	1993	100 (9/9)	2723
IgG	N/A	CD4i	HxB2 new 8b core 6x His	RV305_wk0	0.0 (0/17)		5.6 (1/18)	111	10.5 (2/19)	283	0.0 (0/12)	
IgG	N/A	CD4i	HxB2 new 8b core 6x His	RV305_wk2	100 (17/17)	20647	100 (18/18)	18979	31.6 (6/19)	238	0.0 (0/12)	
IgG	N/A	CD4i	HxB2 new 8b core 6x His	RV305_wk24	94.4 (17/18)	3166	94.4 (17/18)	3218	26.3 (5/19)	204	0.0 (0/12)	
IgG	N/A	CD4i	HxB2 new 8b core 6x His	RV305_wk26	94.4 (17/18)	8787	100 (18/18)	6962	36.8 (7/19)	232	0.0 (0/12)	
IgG	N/A	CD4i	HxB2 new 8b core 6x His	RV305_wk48	100 (17/17)	3755	94.4 (17/18)	3747	36.8 (7/19)	206	0.0 (0/12)	
IgG	N/A	CD4i	HxB2 new 8b core 6x His	RV305_wk72	94.4 (17/18)	2695	94.4 (17/18)	2462	33.3 (6/18)	186	0.0 (0/12)	
IgG	N/A	CD4i	YU2 gp120 WT	RV144_wk26	6.3 (1/16)	872	0.0 (0/15)		6.3 (1/16)	2007	0.0 (0/9)	
IgG	N/A	CD4i	YU2 gp120 WT	RV305_wk0	0.0 (0/17)		11.1 (2/18)	196	5.3 (1/19)	183	0.0 (0/12)	
IgG	N/A	CD4i	YU2 gp120 WT	RV305_wk2	0.0 (0/17)		5.6 (1/18)	21474	10.5 (2/19)	227	0.0 (0/11)	
IgG	N/A	CD4i	YU2 gp120 WT	RV305_wk24	58.8 (10/17)	4414	64.7 (11/17)	4654	15.8 (3/19)	236	0.0 (0/12)	
IgG	N/A	CD4i	YU2 gp120 WT	RV305_wk26	0.0 (0/18)		5.9 (1/17)	24263	26.3 (5/19)	262	0.0 (0/11)	
IgG	N/A	CD4i	YU2 gp120 WT	RV305_wk48	64.7 (11/17)	4869	55.6 (10/18)	7197	26.3 (5/19)	262	0.0 (0/12)	
IgG	N/A	CD4i	YU2 gp120 WT	RV305_wk72	66.7 (12/18)	3248	58.8 (10/17)	4651	22.2 (4/18)	197	0.0 (0/11)	

S1 Table continued

					Group 1: Combination		Group 2: AIDSVAX B/E		Group 3: ALVAC-HIV		RV305_Placebo Group	
Isotype	Clade	Env Region	Antigen	Study Week	Response Rate *(Responders/ Total)*	Median MFI *Responders*	Response Rate *(Responders/ Total)*	Median MFI *Responders*	Response Rate *(Responders/ Total)*	Median MFI *Responders*	Response Rate *(Responders/ Total)*	Median MFI *Responders*
IgG	N/A	CD4bs	RSC3	RV144_wk26	0.0 (0/16)		0.0 (0/15)		0.0 (0/17)		0.0 (0/9)	
IgG	N/A	CD4bs	RSC3	RV305_wk0	0.0 (0/17)		0.0 (0/18)		0.0 (0/19)		0.0 (0/12)	
IgG	N/A	CD4bs	RSC3	RV305_wk2	5.9 (1/17)	133	0.0 (0/18)		0.0 (0/19)		0.0 (0/11)	
IgG	N/A	CD4bs	RSC3	RV305_wk24	0.0 (0/18)		0.0 (0/18)		0.0 (0/19)		0.0 (0/12)	
IgG	N/A	CD4bs	RSC3	RV305_wk26	0.0 (0/18)		0.0 (0/18)		0.0 (0/19)		0.0 (0/11)	
IgG	N/A	CD4bs	RSC3	RV305_wk48	0.0 (0/17)		0.0 (0/18)		0.0 (0/19)		0.0 (0/12)	
IgG	N/A	CD4bs	RSC3	RV305_wk72	0.0 (0/18)		0.0 (0/18)		0.0 (0/18)		0.0 (0/11)	
IgG	N/A	CD4bs	RSC3_P363Npair	RV144_wk26	0.0 (0/16)		0.0 (0/15)		0.0 (0/17)		0.0 (0/9)	
IgG	N/A	CD4bs	RSC3_P363Npair	RV305_wk0	0.0 (0/17)		0.0 (0/18)		0.0 (0/19)		0.0 (0/12)	
IgG	N/A	CD4bs	RSC3_P363Npair	RV305_wk2	0.0 (0/17)		0.0 (0/18)		0.0 (0/19)		0.0 (0/11)	
IgG	N/A	CD4bs	RSC3_P363Npair	RV305_wk24	0.0 (0/18)		0.0 (0/18)		0.0 (0/19)		0.0 (0/12)	
IgG	N/A	CD4bs	RSC3_P363Npair	RV305_wk26	0.0 (0/18)		0.0 (0/18)		0.0 (0/19)		0.0 (0/11)	
IgG	N/A	CD4bs	RSC3_P363Npair	RV305_wk48	0.0 (0/17)		0.0 (0/18)		0.0 (0/19)		0.0 (0/12)	
IgG	N/A	CD4bs	RSC3_P363Npair	RV305_wk72	0.0 (0/18)		0.0 (0/18)		0.0 (0/18)		0.0 (0/11)	
IgG	N/A	Gag (non-Env)	p24	RV144_wk26	43.8 (7/16)	13446	53.3 (8/15)	25810	35.3 (6/17)	14857	55.6 (5/9)	14320
IgG	N/A	Gag (non-Env)	p24	RV305_wk0	0.0 (0/17)		0.0 (0/18)		0.0 (0/19)		8.3 (1/12)	2391
IgG	N/A	Gag (non-Env)	p24	RV305_wk2	23.5 (4/17)	15056	0.0 (0/18)		26.3 (5/19)	3742	8.3 (1/12)	1771
IgG	N/A	Gag (non-Env)	p24	RV305_wk24	16.7 (3/18)	10338	0.0 (0/18)		15.8 (3/19)	4067	0.0 (0/12)	
IgG	N/A	Gag (non-Env)	p24	RV305_wk26	55.6 (10/18)	5352	0.0 (0/18)		36.8 (7/19)	9333	8.3 (1/12)	2562
IgG	N/A	Gag (non-Env)	p24	RV305_wk48	29.4 (5/17)	3069	0.0 (0/18)		31.6 (6/19)	6707	8.3 (1/12)	2700
IgG	N/A	Gag (non-Env)	p24	RV305_wk72	22.2 (4/18)	8105	0.0 (0/18)		22.2 (4/18)	3300	8.3 (1/12)	2316
