# Supplementary material for: Viral vector delivered immunogen focuses HIV-1 antibody specificity and increases durability of the circulating antibody recall response
Source: PLoS Pathog. 2023 May 31;19(5):e1011359. doi: 10.1371/journal.ppat.1011359 (PMC10284421; doi:10.1371/journal.ppat.1011359)
Supplement: S2 Table — (PDF) [file ppat.1011359.s015.pdf]

S2 Table. Linear peptide epitope definition.

Epitope	Peptide Range	Amino Acid (aa) Range (HXB2 Numbering)
C1.1	#24-25	aa71-88
C1.2	#35-36	aa104-121
C1-V1	#40	aa119-133
V2.hs	#53-54	aa163-180
V2.2	#57	aa175-189
C2	#84	aa256-270
V3	#99-100	aa301-318
C5.1	#149-150	aa463-480
C5.2	#157-158	aa487-504
C5.3	#160	aa496-510
