# Supplementary material for: Viral vector delivered immunogen focuses HIV-1 antibody specificity and increases durability of the circulating antibody recall response
Source: PLoS Pathog. 2023 May 31;19(5):e1011359. doi: 10.1371/journal.ppat.1011359 (PMC10284421; doi:10.1371/journal.ppat.1011359)
Supplement: S3 Table — (PDF) [file ppat.1011359.s016.pdf]

S3 Table. Plasma IgG binding to linear V2 hotspot at RV305 weeks 2 and 26.

		Response Rate (%) to V2 Hotspot (V2.hs)	
**Strain**	**Vaccination Group**	**RV305 Week 2**	**RV305 Week 26**
AE.A244	G1 – Combination	100	100
	G2 – AIDSVAX	100	89
	G3 – ALVAC	21	37
	RV305 Placebo	0	0
B.MN	G1 – Combination	60	50
	G2 – AIDSVAX	50	44
	G3 – ALVAC	0	0
	RV305 Placebo	0	0
AE.TH023	G1 – Combination	100	100
	G2 – AIDSVAX	100	89
	G3 – ALVAC	21	37
	RV305 Placebo	0	0
