# Supplementary material for: Viral vector delivered immunogen focuses HIV-1 antibody specificity and increases durability of the circulating antibody recall response
Source: PLoS Pathog. 2023 May 31;19(5):e1011359. doi: 10.1371/journal.ppat.1011359 (PMC10284421; doi:10.1371/journal.ppat.1011359)
Supplement: S4 Table — (PDF) [file ppat.1011359.s017.pdf]

S4 Table. Median IgG CH58 µg/mL equivalent V1V2 concentrations among positive responders.

				G1-Combination		G2-AIDSVAX B/E		G3-ALVAC-HIV		RV305 Placebo	
Isotype	Clade	Antigen	Study Week	Number of Positive Responders	Median (µg/ml)	Number of Positive Responders	Median (µg/ml)	Number of Positive Responders	Median (µg/ml)	Number of Positive Responders	Median (µg/ml)
IgG	AE	AE.A244 V1V2 tags	RV144_wk26	14	11.47	13	10.32	15	14.71	8	15.97
IgG	AE	AE.A244 V1V2 tags	RV305_wk0	1	0.47	0				2	0.13
IgG	AE	AE.A244 V1V2 tags	RV305_wk2	6	17.265	12	20.765	15	0.25	2	0.1
IgG	AE	AE.A244 V1V2 tags	RV305_wk24	16	1.32	17	1.26	10	0.22	1	0.11
IgG	AE	AE.A244 V1V2 tags	RV305_wk26	17	13.42	16	10.51	16	0.33	1	0.1
IgG	AE	AE.A244 V1V2 tags	RV305_wk48	17	1.32	18	1.345	15	0.22	1	0.12
IgG	AE	AE.A244 V1V2 tags	RV305_wk72	16	0.79	18	0.82	8	0.14	1	0.11
IgG	B	gp70_B.CaseA2 V1/V2/169K	RV144_wk26	12	0.595	14	0.245	15	0.16	9	0.39
IgG	B	gp70_B.CaseA2 V1/V2/169K	RV305_wk0	1	0.11	0		0		0	
IgG	B	gp70_B.CaseA2 V1/V2/169K	RV305_wk2	15	1.43	16	1.35	1	0.02	0	
IgG	B	gp70_B.CaseA2 V1/V2/169K	RV305_wk24	7	0.1	7	0.15	0		0	
IgG	B	gp70_B.CaseA2 V1/V2/169K	RV305_wk26	16	0.75	17	0.4	0		0	
IgG	B	gp70_B.CaseA2 V1/V2/169K	RV305_wk48	8	0.105	7	0.16	0		0	
IgG	B	gp70_B.CaseA2 V1/V2/169K	RV305_wk72	6	0.17	5	0.1	0		0	
IgG	B	gp70_B.CaseA_V1_V2	RV144_wk26	12	8.365	13	4.7	15	3.35	9	4.38
IgG	B	gp70_B.CaseA_V1_V2	RV305_wk0	1	2.15	0		1	1.01	0	
IgG	B	gp70_B.CaseA_V1_V2	RV305_wk2	17	20	17	9.53	1	2.29	0	
IgG	B	gp70_B.CaseA_V1_V2	RV305_wk24	8	0.965	4	2.92	0		0	
IgG	B	gp70_B.CaseA_V1_V2	RV305_wk26	16	9.84	17	6.8	1	2.49	0	
IgG	B	gp70_B.CaseA_V1_V2	RV305_wk48	9	1.04	6	1.595	1	2.66	0	
IgG	B	gp70_B.CaseA_V1_V2	RV305_wk72	7	1.2	3	2.61	1	2.76	0	

No median concentration values listed if there were no positive responders at a given time point.
