# Supplementary material for: Viral vector delivered immunogen focuses HIV-1 antibody specificity and increases durability of the circulating antibody recall response
Source: PLoS Pathog. 2023 May 31;19(5):e1011359. doi: 10.1371/journal.ppat.1011359 (PMC10284421; doi:10.1371/journal.ppat.1011359)
Supplement: S5 Table — (PDF) [file ppat.1011359.s018.pdf]

S5 Table. Percent of positive responders in all three RV305 vaccine groups for IgG gp70_B.CaseA_V1_V2 CH58 μg/mL equivalent concentrations above 2.98 μg/mL concentration associated with decreased HIV-1 risk.

Study Week	Value	Negative Responders	Positive Responders	Total Number of Participants Analyzed
RV144_wk26	n	18	31	49
	%	37	**63**	
RV305_wk0	n	2	0	2
	%	100	0	
RV305_wk2	n	7	28	35
	%	20	**80**	
RV305_wk24	n	9	3	12
	%	75	**25**	
RV305_wk26	n	7	27	34
	%	21	**79**	
RV305_wk48	n	15	1	16
	%	94	**6**	
RV305_wk72	n	9	2	11
	%	82	**18**	
Total		67	92	159

Percent of positive responders for IgG gp70_B.CaseA_V1_V2, where concentration was reported and was >2.98 CH58 μg/mL value associated with decreased HIV-1 risk [1] are shown in **bold font**, at each week.

Reference

1. Corey L, Gilbert PB, Tomaras GD, Haynes BF, Pantaleo G, Fauci AS. Immune correlates of vaccine protection against HIV-1 acquisition. Sci Transl Med. 2015;7(310):310rv7. doi: 10.1126/scitranslmed.aac7732. PubMed PMID: 26491081; PubMed Central PMCID: PMCPMC4751141.
