# Supplementary material for: Viral vector delivered immunogen focuses HIV-1 antibody specificity and increases durability of the circulating antibody recall response
Source: PLoS Pathog. 2023 May 31;19(5):e1011359. doi: 10.1371/journal.ppat.1011359 (PMC10284421; doi:10.1371/journal.ppat.1011359)
Supplement: S6 Table — (PDF) [file ppat.1011359.s019.pdf]

S6 Table. Median IgG breadth scores per vaccination group.

Isotype	Panel	Group	Study Week	Number of Participant Analyzed for Breadth Score Calculation	Median Breadth Score
IgG	V1V2 breadth panel	G1_Combination	RV144_wk0	16	12.5
IgG	V1V2 breadth panel	G1_Combination	RV144_wk26	16	6047.2
IgG	V1V2 breadth panel	G1_Combination	RV305_wk0	17	10.5
IgG	V1V2 breadth panel	G1_Combination	RV305_wk2	17	16041.8
IgG	V1V2 breadth panel	G1_Combination	RV305_wk24	17	1180.8
IgG	V1V2 breadth panel	G1_Combination	RV305_wk26	18	9458
IgG	V1V2 breadth panel	G1_Combination	RV305_wk48	17	1337.8
IgG	V1V2 breadth panel	G1_Combination	RV305_wk72	17	853.4
IgG	V1V2 breadth panel	G2_AIDSVAX B/E	RV144_wk0	15	<10
IgG	V1V2 breadth panel	G2_AIDSVAX B/E	RV144_wk26	15	5922.2
IgG	V1V2 breadth panel	G2_AIDSVAX B/E	RV305_wk0	18	<10
IgG	V1V2 breadth panel	G2_AIDSVAX B/E	RV305_wk2	18	15053.1
IgG	V1V2 breadth panel	G2_AIDSVAX B/E	RV305_wk24	18	1229.8
IgG	V1V2 breadth panel	G2_AIDSVAX B/E	RV305_wk26	17	11083.5
IgG	V1V2 breadth panel	G2_AIDSVAX B/E	RV305_wk48	18	1100.2
IgG	V1V2 breadth panel	G2_AIDSVAX B/E	RV305_wk72	18	703.7
IgG	V1V2 breadth panel	G3_ALVAC-HIV	RV144_wk0	17	<10
IgG	V1V2 breadth panel	G3_ALVAC-HIV	RV144_wk26	16	6247.2
IgG	V1V2 breadth panel	G3_ALVAC-HIV	RV305_wk0	18	<10
IgG	V1V2 breadth panel	G3_ALVAC-HIV	RV305_wk2	18	190.7
IgG	V1V2 breadth panel	G3_ALVAC-HIV	RV305_wk24	19	62.9
IgG	V1V2 breadth panel	G3_ALVAC-HIV	RV305_wk26	18	329.9
IgG	V1V2 breadth panel	G3_ALVAC-HIV	RV305_wk48	19	175.1
IgG	V1V2 breadth panel	G3_ALVAC-HIV	RV305_wk72	18	100.9
IgG	V1V2 breadth panel	RV305_Placebo	RV144_wk0	11	<10
IgG	V1V2 breadth panel	RV305_Placebo	RV144_wk26	9	6360.2
IgG	V1V2 breadth panel	RV305_Placebo	RV305_wk0	12	12.6
IgG	V1V2 breadth panel	RV305_Placebo	RV305_wk2	12	<10
IgG	V1V2 breadth panel	RV305_Placebo	RV305_wk24	12	<10
IgG	V1V2 breadth panel	RV305_Placebo	RV305_wk26	12	<10
IgG	V1V2 breadth panel	RV305_Placebo	RV305_wk48	12	<10
IgG	V1V2 breadth panel	RV305_Placebo	RV305_wk72	12	<10

S6 Table continued

Isotype	Panel	Group	Study Week	Number of Participant Analyzed for Breadth Score Calculation	Median Breadth Score
IgG	gp120 breadth panel	G1_Combination	RV144_wk0	16	<10
IgG	gp120 breadth panel	G1_Combination	RV144_wk26	16	7597.4
IgG	gp120 breadth panel	G1_Combination	RV305_wk0	17	55.5
IgG	gp120 breadth panel	G1_Combination	RV305_wk2	17	21105.1
IgG	gp120 breadth panel	G1_Combination	RV305_wk24	18	4287.9
IgG	gp120 breadth panel	G1_Combination	RV305_wk26	18	13991
IgG	gp120 breadth panel	G1_Combination	RV305_wk48	17	4717.6
IgG	gp120 breadth panel	G1_Combination	RV305_wk72	18	4492.7
IgG	gp120 breadth panel	G2_AIDSVAX B/E	RV144_wk0	15	<10
IgG	gp120 breadth panel	G2_AIDSVAX B/E	RV144_wk26	15	8432.5
IgG	gp120 breadth panel	G2_AIDSVAX B/E	RV305_wk0	18	62.3
IgG	gp120 breadth panel	G2_AIDSVAX B/E	RV305_wk2	18	21730.3
IgG	gp120 breadth panel	G2_AIDSVAX B/E	RV305_wk24	18	4864.3
IgG	gp120 breadth panel	G2_AIDSVAX B/E	RV305_wk26	18	13577.9
IgG	gp120 breadth panel	G2_AIDSVAX B/E	RV305_wk48	18	5167.5
IgG	gp120 breadth panel	G2_AIDSVAX B/E	RV305_wk72	18	4166
IgG	gp120 breadth panel	G3_ALVAC-HIV	RV144_wk0	17	<10
IgG	gp120 breadth panel	G3_ALVAC-HIV	RV144_wk26	17	7695.1
IgG	gp120 breadth panel	G3_ALVAC-HIV	RV305_wk0	19	73.1
IgG	gp120 breadth panel	G3_ALVAC-HIV	RV305_wk2	19	167.4
IgG	gp120 breadth panel	G3_ALVAC-HIV	RV305_wk24	19	135
IgG	gp120 breadth panel	G3_ALVAC-HIV	RV305_wk26	19	254
IgG	gp120 breadth panel	G3_ALVAC-HIV	RV305_wk48	19	208.3
IgG	gp120 breadth panel	G3_ALVAC-HIV	RV305_wk72	18	138.7
IgG	gp120 breadth panel	RV305_Placebo	RV144_wk0	11	<10
IgG	gp120 breadth panel	RV305_Placebo	RV144_wk26	9	9193.8
IgG	gp120 breadth panel	RV305_Placebo	RV305_wk0	12	36.5
IgG	gp120 breadth panel	RV305_Placebo	RV305_wk2	12	32.5
IgG	gp120 breadth panel	RV305_Placebo	RV305_wk24	12	36.1
IgG	gp120 breadth panel	RV305_Placebo	RV305_wk26	12	30.9
IgG	gp120 breadth panel	RV305_Placebo	RV305_wk48	12	33.8
IgG	gp120 breadth panel	RV305_Placebo	RV305_wk72	12	32.3

S6 Table continued

Isotype	Panel	Group	Study Week	Number of Participant Analyzed for Breadth Score Calculation	Median Breadth Score
IgG	gp140 breadth panel	G1_Combination	RV144_wk0	16	<10
IgG	gp140 breadth panel	G1_Combination	RV144_wk26	16	5556.9
IgG	gp140 breadth panel	G1_Combination	RV305_wk0	17	24.8
IgG	gp140 breadth panel	G1_Combination	RV305_wk2	17	24152.3
IgG	gp140 breadth panel	G1_Combination	RV305_wk24	18	4240.6
IgG	gp140 breadth panel	G1_Combination	RV305_wk26	18	13394.4
IgG	gp140 breadth panel	G1_Combination	RV305_wk48	17	5098.2
IgG	gp140 breadth panel	G1_Combination	RV305_wk72	18	3429.8
IgG	gp140 breadth panel	G2_AIDSVAX B/E	RV144_wk0	15	<10
IgG	gp140 breadth panel	G2_AIDSVAX B/E	RV144_wk26	15	5424.7
IgG	gp140 breadth panel	G2_AIDSVAX B/E	RV305_wk0	18	30.5
IgG	gp140 breadth panel	G2_AIDSVAX B/E	RV305_wk2	18	24391.5
IgG	gp140 breadth panel	G2_AIDSVAX B/E	RV305_wk24	18	4440.3
IgG	gp140 breadth panel	G2_AIDSVAX B/E	RV305_wk26	18	14099.3
IgG	gp140 breadth panel	G2_AIDSVAX B/E	RV305_wk48	18	4806
IgG	gp140 breadth panel	G2_AIDSVAX B/E	RV305_wk72	18	3175.5
IgG	gp140 breadth panel	G3_ALVAC-HIV	RV144_wk0	17	<10
IgG	gp140 breadth panel	G3_ALVAC-HIV	RV144_wk26	17	6047.5
IgG	gp140 breadth panel	G3_ALVAC-HIV	RV305_wk0	19	36.5
IgG	gp140 breadth panel	G3_ALVAC-HIV	RV305_wk2	19	75.8
IgG	gp140 breadth panel	G3_ALVAC-HIV	RV305_wk24	19	84.1
IgG	gp140 breadth panel	G3_ALVAC-HIV	RV305_wk26	19	171.1
IgG	gp140 breadth panel	G3_ALVAC-HIV	RV305_wk48	19	94.4
IgG	gp140 breadth panel	G3_ALVAC-HIV	RV305_wk72	18	78.8
IgG	gp140 breadth panel	RV305_Placebo	RV144_wk0	11	<10
IgG	gp140 breadth panel	RV305_Placebo	RV144_wk26	9	6852.8
IgG	gp140 breadth panel	RV305_Placebo	RV305_wk0	12	<10
IgG	gp140 breadth panel	RV305_Placebo	RV305_wk2	12	<10
IgG	gp140 breadth panel	RV305_Placebo	RV305_wk24	12	<10
IgG	gp140 breadth panel	RV305_Placebo	RV305_wk26	12	<10
IgG	gp140 breadth panel	RV305_Placebo	RV305_wk48	12	<10
IgG	gp140 breadth panel	RV305_Placebo	RV305_wk72	12	<10
