# Supplementary material for: Viral vector delivered immunogen focuses HIV-1 antibody specificity and increases durability of the circulating antibody recall response
Source: PLoS Pathog. 2023 May 31;19(5):e1011359. doi: 10.1371/journal.ppat.1011359 (PMC10284421; doi:10.1371/journal.ppat.1011359)
Supplement: S7 Table — (PDF) [file ppat.1011359.s020.pdf]

S7 Table. BAMA plasma binding IgG1 response rates and group median binding magnitudes (MFI) to gp120, gp140, V1V2, V3, CD4 inducible, CD4 binding site, and Gag HIV-1 regions.

					Group 1: Combination		Group 2: AIDSVAX B/E		Group 3: ALVAC-HIV		RV305_Placebo Group	
Isotype	Clade	Env Region	Antigen	Study Week	Response Rate *(Responders/ Total)*	Median MFI *Responders*	Response Rate *(Responders/ Total)*	Median MFI *Responders*	Response Rate *(Responders/ Total)*	Median MFI *Responders*	Response Rate *(Responders/ Total)*	Median MFI *Responders*
IgG1	A	gp120	51802_D11gp120.avi/293F	RV144_wk26	0.0 (0/17)		6.7 (1/15)	112	0.0 (0/17)		0.0 (0/10)	
IgG1	A	gp120	51802_D11gp120.avi/293F	RV305_wk0	0.0 (0/20)		0.0 (0/18)		0.0 (0/19)		0.0 (0/13)	
IgG1	A	gp120	51802_D11gp120.avi/293F	RV305_wk2	80.0 (16/20)	318	88.9 (16/18)	440	0.0 (0/19)		0.0 (0/13)	
IgG1	A	gp120	51802_D11gp120.avi/293F	RV305_wk24	0.0 (0/20)		0.0 (0/18)		0.0 (0/19)		0.0 (0/13)	
IgG1	A	gp120	51802_D11gp120.avi/293F	RV305_wk26	30.0 (6/20)	194	38.9 (7/18)	311	0.0 (0/19)		0.0 (0/13)	
IgG1	A	gp120	51802_D11gp120.avi/293F	RV305_wk48	0.0 (0/20)		0.0 (0/18)		0.0 (0/19)		0.0 (0/13)	
IgG1	A	gp120	51802_D11gp120.avi/293F	RV305_wk72	0.0 (0/20)		0.0 (0/18)		0.0 (0/18)		0.0 (0/13)	
IgG1	B	gp120	B.6240_D11gp120/293F	RV144_wk26	23.5 (4/17)	267	60.0 (9/15)	118	35.3 (6/17)	231	40.0 (4/10)	150
IgG1	B	gp120	B.6240_D11gp120/293F	RV305_wk0	0.0 (0/20)		0.0 (0/18)		0.0 (0/19)		0.0 (0/13)	
IgG1	B	gp120	B.6240_D11gp120/293F	RV305_wk2	100 (20/20)	1044	94.4 (17/18)	1473	0.0 (0/19)		0.0 (0/13)	
IgG1	B	gp120	B.6240_D11gp120/293F	RV305_wk24	5.0 (1/20)	108	0.0 (0/18)		0.0 (0/19)		0.0 (0/13)	
IgG1	B	gp120	B.6240_D11gp120/293F	RV305_wk26	90.0 (18/20)	369	94.4 (17/18)	528	0.0 (0/19)		0.0 (0/13)	
IgG1	B	gp120	B.6240_D11gp120/293F	RV305_wk48	5.0 (1/20)	116	0.0 (0/18)		0.0 (0/19)		0.0 (0/13)	
IgG1	B	gp120	B.6240_D11gp120/293F	RV305_wk72	0.0 (0/20)		0.0 (0/18)		0.0 (0/18)		0.0 (0/13)	
IgG1	B	gp120	BORI_D11gp120.avi/293F	RV144_wk26	0.0 (0/17)		26.7 (4/15)	113	5.9 (1/17)	160	10.0 (1/10)	133
IgG1	B	gp120	BORI_D11gp120.avi/293F	RV305_wk0	0.0 (0/20)		0.0 (0/18)		0.0 (0/19)		0.0 (0/13)	
IgG1	B	gp120	BORI_D11gp120.avi/293F	RV305_wk2	100 (20/20)	648	100 (18/18)	795	0.0 (0/19)		0.0 (0/13)	
IgG1	B	gp120	BORI_D11gp120.avi/293F	RV305_wk24	5.0 (1/20)	108	0.0 (0/18)		0.0 (0/19)		0.0 (0/13)	
IgG1	B	gp120	BORI_D11gp120.avi/293F	RV305_wk26	50.0 (10/20)	318	88.9 (16/18)	202	0.0 (0/19)		0.0 (0/13)	
IgG1	B	gp120	BORI_D11gp120.avi/293F	RV305_wk48	0.0 (0/20)		0.0 (0/18)		0.0 (0/19)		0.0 (0/13)	
IgG1	B	gp120	BORI_D11gp120.avi/293F	RV305_wk72	0.0 (0/20)		0.0 (0/18)		0.0 (0/18)		0.0 (0/13)	
IgG1	B	gp120	MN gp120 gDneg/293F	RV144_wk26	92.3 (12/13)	274	85.7 (12/14)	1577	83.3 (10/12)	1298	90.0 (9/10)	1002
IgG1	B	gp120	MN gp120 gDneg/293F	RV305_wk0	0.0 (0/16)		0.0 (0/17)		0.0 (0/16)		0.0 (0/13)	
IgG1	B	gp120	MN gp120 gDneg/293F	RV305_wk2	100 (17/17)	7228	100 (15/15)	10738	0.0 (0/16)		0.0 (0/13)	
IgG1	B	gp120	MN gp120 gDneg/293F	RV305_wk24	45.0 (9/20)	331	55.6 (10/18)	235	0.0 (0/19)		0.0 (0/13)	
IgG1	B	gp120	MN gp120 gDneg/293F	RV305_wk26	100 (18/18)	2688	100 (16/16)	4210	0.0 (0/16)		0.0 (0/13)	
IgG1	B	gp120	MN gp120 gDneg/293F	RV305_wk48	50.0 (9/18)	349	76.5 (13/17)	245	0.0 (0/17)		0.0 (0/13)	
IgG1	B	gp120	MN gp120 gDneg/293F	RV305_wk72	38.9 (7/18)	260	33.3 (5/15)	149	0.0 (0/16)		0.0 (0/13)	

					Group 1: Combination		Group 2: AIDSVAX B/E		Group 3: ALVAC-HIV		RV305_Placebo Group	
Isotype	Clade	Env Region	Antigen	Study Week	Response Rate (Responders/ Total)	Median MFI Responders	Response Rate (Responders/ Total)	Median MFI Responders	Response Rate (Responders/ Total)	Median MFI Responders	Response Rate (Responders/ Total)	Median MFI Responders
IgG1	B	gp120	TT31P.2792_D11gp120.avi/293F	RV144_wk26	88.2 (15/17)	250	86.7 (13/15)	492	82.4 (14/17)	335	90.0 (9/10)	398
IgG1	B	gp120	TT31P.2792_D11gp120.avi/293F	RV305_wk0	0.0 (0/20)		0.0 (0/18)		0.0 (0/19)		0.0 (0/13)	
IgG1	B	gp120	TT31P.2792_D11gp120.avi/293F	RV305_wk2	100 (20/20)	3930	100 (18/18)	3950	0.0 (0/19)		0.0 (0/13)	
IgG1	B	gp120	TT31P.2792_D11gp120.avi/293F	RV305_wk24	65.0 (13/20)	247	66.7 (12/18)	166	0.0 (0/19)		0.0 (0/13)	
IgG1	B	gp120	TT31P.2792_D11gp120.avi/293F	RV305_wk26	100 (20/20)	881	100 (18/18)	1277	0.0 (0/19)		0.0 (0/13)	
IgG1	B	gp120	TT31P.2792_D11gp120.avi/293F	RV305_wk48	65.0 (13/20)	257	72.2 (13/18)	180	0.0 (0/19)		0.0 (0/13)	
IgG1	B	gp120	TT31P.2792_D11gp120.avi/293F	RV305_wk72	50.0 (10/20)	251	50.0 (9/18)	161	0.0 (0/18)		0.0 (0/13)	
IgG1	C	gp120	1086C_D7gp120.avi/293F	RV144_wk26	94.1 (16/17)	2024	93.3 (14/15)	2003	100 (17/17)	1822	100 (10/10)	1907
IgG1	C	gp120	1086C_D7gp120.avi/293F	RV305_wk0	0.0 (0/20)		0.0 (0/18)		0.0 (0/19)		0.0 (0/13)	
IgG1	C	gp120	1086C_D7gp120.avi/293F	RV305_wk2	100 (20/20)	13139	100 (18/18)	11749	0.0 (0/19)		0.0 (0/13)	
IgG1	C	gp120	1086C_D7gp120.avi/293F	RV305_wk24	80.0 (16/20)	826	94.4 (17/18)	747	0.0 (0/19)		0.0 (0/13)	
IgG1	C	gp120	1086C_D7gp120.avi/293F	RV305_wk26	100 (20/20)	3772	100 (18/18)	4085	0.0 (0/19)		0.0 (0/13)	
IgG1	C	gp120	1086C_D7gp120.avi/293F	RV305_wk48	90.0 (18/20)	936	94.4 (17/18)	826	0.0 (0/19)		0.0 (0/13)	
IgG1	C	gp120	1086C_D7gp120.avi/293F	RV305_wk72	80.0 (16/20)	764	83.3 (15/18)	469	0.0 (0/18)		0.0 (0/13)	
IgG1	C	gp120	96ZM651.D11gp120.avi	RV144_wk26	17.6 (3/17)	161	26.7 (4/15)	138	17.6 (3/17)	193	30.0 (3/10)	591
IgG1	C	gp120	96ZM651.D11gp120.avi	RV305_wk0	0.0 (0/20)		0.0 (0/18)		0.0 (0/19)		0.0 (0/13)	
IgG1	C	gp120	96ZM651.D11gp120.avi	RV305_wk2	95.0 (19/20)	800	100 (18/18)	590	0.0 (0/19)		0.0 (0/13)	
IgG1	C	gp120	96ZM651.D11gp120.avi	RV305_wk24	5.0 (1/20)	240	0.0 (0/18)		0.0 (0/19)		0.0 (0/13)	
IgG1	C	gp120	96ZM651.D11gp120.avi	RV305_wk26	60.0 (12/20)	534	83.3 (15/18)	210	0.0 (0/19)		0.0 (0/13)	
IgG1	C	gp120	96ZM651.D11gp120.avi	RV305_wk48	5.0 (1/20)	177	0.0 (0/18)		0.0 (0/19)		0.0 (0/13)	
IgG1	C	gp120	96ZM651.D11gp120.avi	RV305_wk72	0.0 (0/20)		0.0 (0/18)		0.0 (0/18)		0.0 (0/13)	
IgG1	C	gp120	TV1c8_D11gp120.avi/293F	RV144_wk26	41.2 (7/17)	374	66.7 (10/15)	432	52.9 (9/17)	406	70.0 (7/10)	265
IgG1	C	gp120	TV1c8_D11gp120.avi/293F	RV305_wk0	0.0 (0/20)		0.0 (0/18)		0.0 (0/19)		0.0 (0/13)	
IgG1	C	gp120	TV1c8_D11gp120.avi/293F	RV305_wk2	100 (20/20)	3040	100 (18/18)	3562	0.0 (0/19)		0.0 (0/13)	
IgG1	C	gp120	TV1c8_D11gp120.avi/293F	RV305_wk24	10.0 (2/20)	141	5.6 (1/18)	137	0.0 (0/19)		0.0 (0/13)	
IgG1	C	gp120	TV1c8_D11gp120.avi/293F	RV305_wk26	90.0 (18/20)	861	100 (18/18)	1035	0.0 (0/19)		0.0 (0/13)	
IgG1	C	gp120	TV1c8_D11gp120.avi/293F	RV305_wk48	10.0 (2/20)	106	5.6 (1/18)	188	0.0 (0/19)		0.0 (0/13)	
IgG1	C	gp120	TV1c8_D11gp120.avi/293F	RV305_wk72	0.0 (0/20)		0.0 (0/18)		0.0 (0/18)		0.0 (0/13)	
IgG1	CRF01_AE	gp120	254008_D11gp120.avi/293F	RV144_wk26	47.1 (8/17)	153	53.3 (8/15)	221	52.9 (9/17)	136	80.0 (8/10)	193
IgG1	CRF01_AE	gp120	254008_D11gp120.avi/293F	RV305_wk0	0.0 (0/20)		0.0 (0/18)		0.0 (0/19)		0.0 (0/13)	
IgG1	CRF01_AE	gp120	254008_D11gp120.avi/293F	RV305_wk2	100 (20/20)	1494	100 (18/18)	1479	0.0 (0/19)		0.0 (0/13)	
IgG1	CRF01_AE	gp120	254008_D11gp120.avi/293F	RV305_wk24	35.0 (7/20)	255	27.8 (5/18)	155	0.0 (0/19)		0.0 (0/13)	
IgG1	CRF01_AE	gp120	254008_D11gp120.avi/293F	RV305_wk26	90.0 (18/20)	498	94.4 (17/18)	378	0.0 (0/19)		0.0 (0/13)	
IgG1	CRF01_AE	gp120	254008_D11gp120.avi/293F	RV305_wk48	35.0 (7/20)	207	38.9 (7/18)	153	0.0 (0/19)		0.0 (0/13)	
IgG1	CRF01_AE	gp120	254008_D11gp120.avi/293F	RV305_wk72	25.0 (5/20)	207	22.2 (4/18)	130	0.0 (0/18)		0.0 (0/13)	

					Group 1: Combination		Group 2: AIDSVAX B/E		Group 3: ALVAC-HIV		RV305_Placebo Group	
Isotype	Clade	Env Region	Antigen	Study Week	Response Rate (Responders/ Total)	Median MFI Responders	Response Rate (Responders/ Total)	Median MFI Responders	Response Rate (Responders/ Total)	Median MFI Responders	Response Rate (Responders/ Total)	Median MFI Responders
IgG1	CRF01_AE	gp120	92TH023 gp120 gDneg 293F mon	RV144_wk26	29.4 (5/17)	225	26.7 (4/15)	333	35.3 (6/17)	205	40.0 (4/10)	286
IgG1	CRF01_AE	gp120	92TH023 gp120 gDneg 293F mon	RV305_wk0	0.0 (0/20)		0.0 (0/18)		0.0 (0/19)		0.0 (0/13)	
IgG1	CRF01_AE	gp120	92TH023 gp120 gDneg 293F mon	RV305_wk2	95.0 (19/20)	2539	94.4 (17/18)	1946	0.0 (0/19)		0.0 (0/13)	
IgG1	CRF01_AE	gp120	92TH023 gp120 gDneg 293F mon	RV305_wk24	0.0 (0/20)		5.6 (1/18)	326	0.0 (0/19)		0.0 (0/13)	
IgG1	CRF01_AE	gp120	92TH023 gp120 gDneg 293F mon	RV305_wk26	65.0 (13/20)	459	72.2 (13/18)	373	0.0 (0/19)		0.0 (0/13)	
IgG1	CRF01_AE	gp120	92TH023 gp120 gDneg 293F mon	RV305_wk48	0.0 (0/20)		5.6 (1/18)	622	0.0 (0/19)		0.0 (0/13)	
IgG1	CRF01_AE	gp120	92TH023 gp120 gDneg 293F mon	RV305_wk72	0.0 (0/20)		5.6 (1/18)	189	0.0 (0/18)		0.0 (0/13)	
IgG1	CRF01_AE	gp120	A244 D11gp120_avi	RV144_wk26	52.9 (9/17)	877	66.7 (10/15)	437	70.6 (12/17)	461	80.0 (8/10)	516
IgG1	CRF01_AE	gp120	A244 D11gp120_avi	RV305_wk0	0.0 (0/20)		0.0 (0/18)		0.0 (0/19)		0.0 (0/13)	
IgG1	CRF01_AE	gp120	A244 D11gp120_avi	RV305_wk2	100 (20/20)	5841	100 (18/18)	4181	0.0 (0/19)		0.0 (0/13)	
IgG1	CRF01_AE	gp120	A244 D11gp120_avi	RV305_wk24	25.0 (5/20)	120	27.8 (5/18)	240	0.0 (0/19)		0.0 (0/13)	
IgG1	CRF01_AE	gp120	A244 D11gp120_avi	RV305_wk26	94.7 (18/19)	985	100 (18/18)	737	0.0 (0/19)		0.0 (0/13)	
IgG1	CRF01_AE	gp120	A244 D11gp120_avi	RV305_wk48	30.0 (6/20)	217	27.8 (5/18)	208	0.0 (0/19)		0.0 (0/13)	
IgG1	CRF01_AE	gp120	A244 D11gp120_avi	RV305_wk72	10.0 (2/20)	145	27.8 (5/18)	152	0.0 (0/18)		0.0 (0/13)	
IgG1	CRF01_AE	gp120	CM235 gp120	RV144_wk26	0.0 (0/17)		0.0 (0/15)		0.0 (0/17)		0.0 (0/10)	
IgG1	CRF01_AE	gp120	CM235 gp120	RV305_wk0	0.0 (0/20)		0.0 (0/18)		0.0 (0/19)		0.0 (0/13)	
IgG1	CRF01_AE	gp120	CM235 gp120	RV305_wk2	30.0 (6/20)	2374	27.8 (5/18)	6813	0.0 (0/19)		0.0 (0/13)	
IgG1	CRF01_AE	gp120	CM235 gp120	RV305_wk24	0.0 (0/20)		0.0 (0/18)		0.0 (0/19)		0.0 (0/13)	
IgG1	CRF01_AE	gp120	CM235 gp120	RV305_wk26	5.0 (1/20)	2360	11.1 (2/18)	2660	0.0 (0/19)		0.0 (0/13)	
IgG1	CRF01_AE	gp120	CM235 gp120	RV305_wk48	5.0 (1/20)	6658	0.0 (0/18)		0.0 (0/19)		0.0 (0/13)	
IgG1	CRF01_AE	gp120	CM235 gp120	RV305_wk72	5.0 (1/20)	8544	0.0 (0/18)		0.0 (0/18)		0.0 (0/13)	
IgG1	CRF07_BC	gp120	BJOX002_D11gp120.avi/293F	RV144_wk26	0.0 (0/17)		0.0 (0/15)		0.0 (0/17)		20.0 (2/10)	137
IgG1	CRF07_BC	gp120	BJOX002_D11gp120.avi/293F	RV305_wk0	0.0 (0/20)		0.0 (0/18)		0.0 (0/19)		0.0 (0/13)	
IgG1	CRF07_BC	gp120	BJOX002_D11gp120.avi/293F	RV305_wk2	95.0 (19/20)	284	88.9 (16/18)	289	0.0 (0/19)		0.0 (0/13)	
IgG1	CRF07_BC	gp120	BJOX002_D11gp120.avi/293F	RV305_wk24	0.0 (0/20)		0.0 (0/18)		0.0 (0/19)		0.0 (0/13)	
IgG1	CRF07_BC	gp120	BJOX002_D11gp120.avi/293F	RV305_wk26	30.0 (6/20)	218	33.3 (6/18)	350	0.0 (0/19)		0.0 (0/13)	
IgG1	CRF07_BC	gp120	BJOX002_D11gp120.avi/293F	RV305_wk48	0.0 (0/20)		0.0 (0/18)		0.0 (0/19)		0.0 (0/13)	
IgG1	CRF07_BC	gp120	BJOX002_D11gp120.avi/293F	RV305_wk72	0.0 (0/20)		0.0 (0/18)		0.0 (0/18)		0.0 (0/13)	
IgG1	CRF07_BC	gp120	CNE20_D11gp120.avi/293F	RV144_wk26	29.4 (5/17)	123	53.3 (8/15)	195	29.4 (5/17)	217	50.0 (5/10)	254
IgG1	CRF07_BC	gp120	CNE20_D11gp120.avi/293F	RV305_wk0	0.0 (0/20)		0.0 (0/18)		0.0 (0/19)		0.0 (0/13)	
IgG1	CRF07_BC	gp120	CNE20_D11gp120.avi/293F	RV305_wk2	100 (20/20)	1343	100 (18/18)	1546	0.0 (0/19)		0.0 (0/13)	
IgG1	CRF07_BC	gp120	CNE20_D11gp120.avi/293F	RV305_wk24	20.0 (4/20)	178	0.0 (0/18)		0.0 (0/19)		0.0 (0/13)	
IgG1	CRF07_BC	gp120	CNE20_D11gp120.avi/293F	RV305_wk26	75.0 (15/20)	433	94.4 (17/18)	394	0.0 (0/19)		0.0 (0/13)	
IgG1	CRF07_BC	gp120	CNE20_D11gp120.avi/293F	RV305_wk48	15.0 (3/20)	159	11.1 (2/18)	103	0.0 (0/19)		0.0 (0/13)	
IgG1	CRF07_BC	gp120	CNE20_D11gp120.avi/293F	RV305_wk72	0.0 (0/20)		0.0 (0/18)		0.0 (0/18)		0.0 (0/13)	

					Group 1: Combination		Group 2: AIDSVAX B/E		Group 3: ALVAC-HIV		RV305_Placebo Group	
Isotype	Clade	Env Region	Antigen	Study Week	Response Rate (Responders/ Total)	Median MFI Responders	Response Rate (Responders/ Total)	Median MFI Responders	Response Rate (Responders/ Total)	Median MFI Responders	Response Rate (Responders/ Total)	Median MFI Responders
IgG1	Consensus	gp120	Con 6 gp120/B	RV144_wk26	17.6 (3/17)	189	46.7 (7/15)	145	29.4 (5/17)	187	30.0 (3/10)	200
IgG1	Consensus	gp120	Con 6 gp120/B	RV305_wk0	0.0 (0/20)		0.0 (0/18)		0.0 (0/19)		0.0 (0/13)	
IgG1	Consensus	gp120	Con 6 gp120/B	RV305_wk2	100 (20/20)	1785	100 (18/18)	2057	0.0 (0/19)		0.0 (0/13)	
IgG1	Consensus	gp120	Con 6 gp120/B	RV305_wk24	20.0 (4/20)	144	0.0 (0/18)		0.0 (0/19)		0.0 (0/13)	
IgG1	Consensus	gp120	Con 6 gp120/B	RV305_wk26	85.0 (17/20)	357	77.8 (14/18)	374	0.0 (0/19)		0.0 (0/13)	
IgG1	Consensus	gp120	Con 6 gp120/B	RV305_wk48	15.0 (3/20)	183	5.6 (1/18)	216	0.0 (0/19)		0.0 (0/13)	
IgG1	Consensus	gp120	Con 6 gp120/B	RV305_wk72	5.0 (1/20)	112	5.6 (1/18)	138	0.0 (0/18)		0.0 (0/13)	
IgG1	A	gp140	9004S.gp140C.avi	RV144_wk26	0.0 (0/17)		0.0 (0/15)		0.0 (0/17)		0.0 (0/10)	
IgG1	A	gp140	9004S.gp140C.avi	RV305_wk0	0.0 (0/20)		0.0 (0/18)		0.0 (0/19)		0.0 (0/13)	
IgG1	A	gp140	9004S.gp140C.avi	RV305_wk2	80.0 (16/20)	296	88.9 (16/18)	292	0.0 (0/19)		0.0 (0/13)	
IgG1	A	gp140	9004S.gp140C.avi	RV305_wk24	0.0 (0/20)		0.0 (0/18)		0.0 (0/19)		0.0 (0/13)	
IgG1	A	gp140	9004S.gp140C.avi	RV305_wk26	10.0 (2/20)	233	22.2 (4/18)	222	0.0 (0/19)		0.0 (0/13)	
IgG1	A	gp140	9004S.gp140C.avi	RV305_wk48	0.0 (0/20)		0.0 (0/18)		0.0 (0/19)		0.0 (0/13)	
IgG1	A	gp140	9004S.gp140C.avi	RV305_wk72	0.0 (0/20)		0.0 (0/18)		0.0 (0/18)		0.0 (0/13)	
IgG1	B	gp140	RHPA4259_C7.gp140C.avi	RV144_wk26	23.5 (4/17)	188	40.0 (6/15)	311	35.3 (6/17)	182	10.0 (1/10)	194
IgG1	B	gp140	RHPA4259_C7.gp140C.avi	RV305_wk0	0.0 (0/20)		0.0 (0/18)		0.0 (0/19)		0.0 (0/13)	
IgG1	B	gp140	RHPA4259_C7.gp140C.avi	RV305_wk2	100 (20/20)	1745	100 (18/18)	2228	0.0 (0/19)		0.0 (0/13)	
IgG1	B	gp140	RHPA4259_C7.gp140C.avi	RV305_wk24	20.0 (4/20)	199	0.0 (0/18)		0.0 (0/19)		0.0 (0/13)	
IgG1	B	gp140	RHPA4259_C7.gp140C.avi	RV305_wk26	85.0 (17/20)	427	94.4 (17/18)	473	0.0 (0/19)		0.0 (0/13)	
IgG1	B	gp140	RHPA4259_C7.gp140C.avi	RV305_wk48	15.0 (3/20)	180	0.0 (0/18)		0.0 (0/19)		0.0 (0/13)	
IgG1	B	gp140	RHPA4259_C7.gp140C.avi	RV305_wk72	0.0 (0/20)		0.0 (0/18)		0.0 (0/18)		0.0 (0/13)	
IgG1	B	gp140	SC42261_gp140.avi/293F	RV144_wk26	41.2 (7/17)	176	46.7 (7/15)	495	47.1 (8/17)	215	30.0 (3/10)	135
IgG1	B	gp140	SC42261_gp140.avi/293F	RV305_wk0	0.0 (0/20)		0.0 (0/18)		0.0 (0/19)		0.0 (0/13)	
IgG1	B	gp140	SC42261_gp140.avi/293F	RV305_wk2	100 (20/20)	3538	100 (18/18)	4535	0.0 (0/19)		0.0 (0/13)	
IgG1	B	gp140	SC42261_gp140.avi/293F	RV305_wk24	30.0 (6/20)	168	0.0 (0/18)		0.0 (0/19)		0.0 (0/13)	
IgG1	B	gp140	SC42261_gp140.avi/293F	RV305_wk26	90.0 (18/20)	655	100 (18/18)	759	0.0 (0/19)		0.0 (0/13)	
IgG1	B	gp140	SC42261_gp140.avi/293F	RV305_wk48	30.0 (6/20)	170	22.2 (4/18)	116	0.0 (0/19)		0.0 (0/13)	
IgG1	B	gp140	SC42261_gp140.avi/293F	RV305_wk72	10.0 (2/20)	202	0.0 (0/18)		0.0 (0/18)		0.0 (0/13)	
IgG1	B	gp140	WITO4160.gp140C.avi	RV144_wk26	11.8 (2/17)	164	33.3 (5/15)	196	23.5 (4/17)	125	10.0 (1/10)	170
IgG1	B	gp140	WITO4160.gp140C.avi	RV305_wk0	0.0 (0/20)		0.0 (0/18)		0.0 (0/19)		0.0 (0/13)	
IgG1	B	gp140	WITO4160.gp140C.avi	RV305_wk2	100 (20/20)	1820	100 (18/18)	2030	0.0 (0/19)		0.0 (0/13)	
IgG1	B	gp140	WITO4160.gp140C.avi	RV305_wk24	5.0 (1/20)	156	0.0 (0/18)		0.0 (0/19)		0.0 (0/13)	
IgG1	B	gp140	WITO4160.gp140C.avi	RV305_wk26	80.0 (16/20)	461	83.3 (15/18)	571	0.0 (0/19)		0.0 (0/13)	
IgG1	B	gp140	WITO4160.gp140C.avi	RV305_wk48	5.0 (1/20)	200	0.0 (0/18)		0.0 (0/19)		0.0 (0/13)	
IgG1	B	gp140	WITO4160.gp140C.avi	RV305_wk72	0.0 (0/20)		0.0 (0/18)		0.0 (0/18)		0.0 (0/13)	

S7 Table continued

					Group 1: Combination		Group 2: AIDSVAX B/E		Group 3: ALVAC-HIV		RV305_Placebo Group	
**Isotype**	**Clade**	**Env Region**	**Antigen**	**Study Week**	**Response Rate (Responders/ Total)**	**Median MFI Responders**	**Response Rate (Responders/ Total)**	**Median MFI Responders**	**Response Rate (Responders/ Total)**	**Median MFI Responders**	**Response Rate (Responders/ Total)**	**Median MFI Responders**
IgG1	C	gp140	1086C gp140C_avi	RV144_wk26	94.1 (16/17)	2577	100 (15/15)	2492	100 (17/17)	2943	100 (10/10)	2881
IgG1	C	gp140	1086C gp140C_avi	RV305_wk0	0.0 (0/20)		0.0 (0/18)		0.0 (0/19)		0.0 (0/13)	
IgG1	C	gp140	1086C gp140C_avi	RV305_wk2	100 (20/20)	23503	100 (18/18)	23112	0.0 (0/19)		0.0 (0/13)	
IgG1	C	gp140	1086C gp140C_avi	RV305_wk24	85.0 (17/20)	1404	94.4 (17/18)	860	0.0 (0/19)		0.0 (0/13)	
IgG1	C	gp140	1086C gp140C_avi	RV305_wk26	100 (20/20)	8863	100 (18/18)	8571	0.0 (0/19)		0.0 (0/13)	
IgG1	C	gp140	1086C gp140C_avi	RV305_wk48	85.0 (17/20)	1853	94.4 (17/18)	1044	0.0 (0/19)		0.0 (0/13)	
IgG1	C	gp140	1086C gp140C_avi	RV305_wk72	75.0 (15/20)	1017	83.3 (15/18)	652	0.0 (0/18)		0.0 (0/13)	
IgG1	C	gp140	BF1266_gp140C.avi/293F	RV144_wk26	35.3 (6/17)	270	40.0 (6/15)	464	52.9 (9/17)	206	50.0 (5/10)	174
IgG1	C	gp140	BF1266_gp140C.avi/293F	RV305_wk0	0.0 (0/20)		0.0 (0/18)		0.0 (0/19)		0.0 (0/13)	
IgG1	C	gp140	BF1266_gp140C.avi/293F	RV305_wk2	100 (20/20)	2979	100 (18/18)	3530	0.0 (0/19)		0.0 (0/13)	
IgG1	C	gp140	BF1266_gp140C.avi/293F	RV305_wk24	35.0 (7/20)	183	5.6 (1/18)	142	0.0 (0/19)		0.0 (0/13)	
IgG1	C	gp140	BF1266_gp140C.avi/293F	RV305_wk26	95.0 (19/20)	993	100 (18/18)	1043	0.0 (0/19)		0.0 (0/13)	
IgG1	C	gp140	BF1266_gp140C.avi/293F	RV305_wk48	40.0 (8/20)	133	27.8 (5/18)	173	0.0 (0/19)		0.0 (0/13)	
IgG1	C	gp140	BF1266_gp140C.avi/293F	RV305_wk72	5.0 (1/20)	222	5.6 (1/18)	111	0.0 (0/18)		0.0 (0/13)	
IgG1	C	gp140	C.CH505TF_gp140/293F	RV144_wk26	17.6 (3/17)	129	40.0 (6/15)	196	17.6 (3/17)	453	20.0 (2/10)	236
IgG1	C	gp140	C.CH505TF_gp140/293F	RV305_wk0	0.0 (0/20)		0.0 (0/18)		0.0 (0/19)		0.0 (0/13)	
IgG1	C	gp140	C.CH505TF_gp140/293F	RV305_wk2	100 (20/20)	1606	100 (18/18)	1700	0.0 (0/19)		0.0 (0/13)	
IgG1	C	gp140	C.CH505TF_gp140/293F	RV305_wk24	10.0 (2/20)	215	0.0 (0/18)		0.0 (0/19)		0.0 (0/13)	
IgG1	C	gp140	C.CH505TF_gp140/293F	RV305_wk26	80.0 (16/20)	569	94.4 (17/18)	405	0.0 (0/19)		0.0 (0/13)	
IgG1	C	gp140	C.CH505TF_gp140/293F	RV305_wk48	5.0 (1/20)	246	5.6 (1/18)	153	0.0 (0/19)		0.0 (0/13)	
IgG1	C	gp140	C.CH505TF_gp140/293F	RV305_wk72	5.0 (1/20)	206	0.0 (0/18)		0.0 (0/18)		0.0 (0/13)	
IgG1	Consensus	gp140	Con S gp140 CFI	RV144_wk26	52.9 (9/17)	249	53.8 (7/13)	680	70.6 (12/17)	309	80.0 (8/10)	170
IgG1	Consensus	gp140	Con S gp140 CFI	RV305_wk0	0.0 (0/20)		0.0 (0/18)		0.0 (0/19)		0.0 (0/13)	
IgG1	Consensus	gp140	Con S gp140 CFI	RV305_wk2	100 (20/20)	10108	100 (18/18)	8119	0.0 (0/19)		0.0 (0/13)	
IgG1	Consensus	gp140	Con S gp140 CFI	RV305_wk24	30.0 (6/20)	293	11.1 (2/18)	178	0.0 (0/19)		0.0 (0/13)	
IgG1	Consensus	gp140	Con S gp140 CFI	RV305_wk26	95.0 (19/20)	1227	100 (18/18)	1457	0.0 (0/19)		0.0 (0/13)	
IgG1	Consensus	gp140	Con S gp140 CFI	RV305_wk48	45.0 (9/20)	144	27.8 (5/18)	186	0.0 (0/19)		0.0 (0/13)	
IgG1	Consensus	gp140	Con S gp140 CFI	RV305_wk72	10.0 (2/20)	200	5.6 (1/18)	117	0.0 (0/18)		0.0 (0/13)	

					Group 1: Combination		Group 2: AIDSVAX B/E		Group 3: ALVAC-HIV		RV305_Placebo Group	
Isotype	Clade	Env Region	Antigen	Study Week	Response Rate (Responders/ Total)	Median MFI Responders	Response Rate (Responders/ Total)	Median MFI Responders	Response Rate (Responders/ Total)	Median MFI Responders	Response Rate (Responders/ Total)	Median MFI Responders
IgG1	Consensus CRF01_AE	gp140	AE.01.con_env03 gp140CF_avi	RV144_wk26	41.2 (7/17)	354	40.0 (6/15)	296	35.3 (6/17)	443	40.0 (4/10)	538
IgG1	Consensus CRF01_AE	gp140	AE.01.con_env03 gp140CF_avi	RV305_wk0	0.0 (0/20)		0.0 (0/18)		0.0 (0/19)		0.0 (0/13)	
IgG1	Consensus CRF01_AE	gp140	AE.01.con_env03 gp140CF_avi	RV305_wk2	100 (20/20)	4315	100 (18/18)	4047	0.0 (0/19)		0.0 (0/13)	
IgG1	Consensus CRF01_AE	gp140	AE.01.con_env03 gp140CF_avi	RV305_wk24	5.0 (1/20)	163	16.7 (3/18)	192	0.0 (0/19)		0.0 (0/13)	
IgG1	Consensus CRF01_AE	gp140	AE.01.con_env03 gp140CF_avi	RV305_wk26	90.0 (18/20)	811	94.4 (17/18)	618	0.0 (0/19)		0.0 (0/13)	
IgG1	Consensus CRF01_AE	gp140	AE.01.con_env03 gp140CF_avi	RV305_wk48	15.0 (3/20)	121	22.2 (4/18)	165	0.0 (0/19)		0.0 (0/13)	
IgG1	Consensus CRF01_AE	gp140	AE.01.con_env03 gp140CF_avi	RV305_wk72	0.0 (0/20)		5.6 (1/18)	305	0.0 (0/18)		0.0 (0/13)	
IgG1	A	V1V2	gp70-191084_B7 V1V2	RV144_wk26	76.5 (13/17)	3947	86.7 (13/15)	2194	100 (17/17)	1267	100 (10/10)	2847
IgG1	A	V1V2	gp70-191084_B7 V1V2	RV305_wk0	0.0 (0/20)		0.0 (0/18)		0.0 (0/19)		0.0 (0/13)	
IgG1	A	V1V2	gp70-191084_B7 V1V2	RV305_wk2	100 (20/20)	13093	100 (18/18)	15141	15.8 (3/19)	458	0.0 (0/13)	
IgG1	A	V1V2	gp70-191084_B7 V1V2	RV305_wk24	65.0 (13/20)	1207	61.1 (11/18)	911	10.5 (2/19)	215	0.0 (0/13)	
IgG1	A	V1V2	gp70-191084_B7 V1V2	RV305_wk26	100 (20/20)	6193	100 (18/18)	6138	15.8 (3/19)	866	0.0 (0/13)	
IgG1	A	V1V2	gp70-191084_B7 V1V2	RV305_wk48	65.0 (13/20)	760	55.6 (10/18)	1463	10.5 (2/19)	594	0.0 (0/13)	
IgG1	A	V1V2	gp70-191084_B7 V1V2	RV305_wk72	45.0 (9/20)	735	35.3 (6/17)	3776	5.6 (1/18)	280	0.0 (0/13)	
IgG1	B	V1V2	gp70-62357.14 V1V2	RV144_wk26	0.0 (0/17)		20.0 (3/15)	248	5.9 (1/17)	105	10.0 (1/10)	166
IgG1	B	V1V2	gp70-62357.14 V1V2	RV305_wk0	0.0 (0/20)		0.0 (0/18)		0.0 (0/19)		0.0 (0/13)	
IgG1	B	V1V2	gp70-62357.14 V1V2	RV305_wk2	5.0 (1/20)	330	27.8 (5/18)	1324	0.0 (0/19)		0.0 (0/13)	
IgG1	B	V1V2	gp70-62357.14 V1V2	RV305_wk24	0.0 (0/20)		0.0 (0/18)		0.0 (0/19)		0.0 (0/13)	
IgG1	B	V1V2	gp70-62357.14 V1V2	RV305_wk26	0.0 (0/20)		5.6 (1/18)	154	0.0 (0/19)		0.0 (0/13)	
IgG1	B	V1V2	gp70-62357.14 V1V2	RV305_wk48	0.0 (0/20)		0.0 (0/18)		0.0 (0/19)		0.0 (0/13)	
IgG1	B	V1V2	gp70-62357.14 V1V2	RV305_wk72	0.0 (0/20)		0.0 (0/18)		0.0 (0/18)		0.0 (0/13)	
IgG1	B	V1V2	gp70-700010058 V1V2	RV144_wk26	0.0 (0/17)		0.0 (0/15)		5.9 (1/17)	172	0.0 (0/10)	
IgG1	B	V1V2	gp70-700010058 V1V2	RV305_wk0	0.0 (0/20)		0.0 (0/18)		0.0 (0/19)		0.0 (0/13)	
IgG1	B	V1V2	gp70-700010058 V1V2	RV305_wk2	60.0 (12/20)	427	44.4 (8/18)	1081	0.0 (0/19)		0.0 (0/13)	
IgG1	B	V1V2	gp70-700010058 V1V2	RV305_wk24	0.0 (0/20)		0.0 (0/18)		0.0 (0/19)		0.0 (0/13)	
IgG1	B	V1V2	gp70-700010058 V1V2	RV305_wk26	40.0 (8/20)	995	50.0 (9/18)	387	0.0 (0/19)		0.0 (0/13)	
IgG1	B	V1V2	gp70-700010058 V1V2	RV305_wk48	0.0 (0/20)		0.0 (0/18)		0.0 (0/19)		0.0 (0/13)	
IgG1	B	V1V2	gp70-700010058 V1V2	RV305_wk72	0.0 (0/20)		0.0 (0/18)		0.0 (0/18)		0.0 (0/13)	
IgG1	A	V1V2	gp70-191084_B7 V1V2	RV144_wk26	76.5 (13/17)	3947	86.7 (13/15)	2194	100 (17/17)	1267	100 (10/10)	2847
IgG1	A	V1V2	gp70-191084_B7 V1V2	RV305_wk0	0.0 (0/20)		0.0 (0/18)		0.0 (0/19)		0.0 (0/13)	
IgG1	A	V1V2	gp70-191084_B7 V1V2	RV305_wk2	100 (20/20)	13093	100 (18/18)	15141	15.8 (3/19)	458	0.0 (0/13)	

					Group 1: Combination		Group 2: AIDSVAX B/E		Group 3: ALVAC-HIV		RV305_Placebo Group	
Isotype	Clade	Env Region	Antigen	Study Week	Response Rate (Responders/ Total)	Median MFI Responders	Response Rate (Responders/ Total)	Median MFI Responders	Response Rate (Responders/ Total)	Median MFI Responders	Response Rate (Responders/ Total)	Median MFI Responders
IgG1	B	V1V2	gp70-RHPA4259.7 V1V2	RV144_wk26	5.9 (1/17)	130	20.0 (3/15)	559	5.9 (1/17)	169	20.0 (2/10)	265
IgG1	B	V1V2	gp70-RHPA4259.7 V1V2	RV305_wk0	0.0 (0/20)		0.0 (0/18)		0.0 (0/19)		0.0 (0/13)	
IgG1	B	V1V2	gp70-RHPA4259.7 V1V2	RV305_wk2	45.0 (9/20)	222	33.3 (6/18)	2068	0.0 (0/19)		0.0 (0/13)	
IgG1	B	V1V2	gp70-RHPA4259.7 V1V2	RV305_wk24	0.0 (0/20)		0.0 (0/18)		0.0 (0/19)		0.0 (0/13)	
IgG1	B	V1V2	gp70-RHPA4259.7 V1V2	RV305_wk26	10.0 (2/20)	140	11.1 (2/18)	165	0.0 (0/19)		0.0 (0/13)	
IgG1	B	V1V2	gp70-RHPA4259.7 V1V2	RV305_wk48	0.0 (0/20)		0.0 (0/18)		0.0 (0/19)		0.0 (0/13)	
IgG1	B	V1V2	gp70-RHPA4259.7 V1V2	RV305_wk72	5.0 (1/20)	382	0.0 (0/18)		0.0 (0/18)		0.0 (0/13)	
IgG1	B	V1V2	gp70-TT31P.2F10.2792 V1V2	RV144_wk26	17.6 (3/17)	143	20.0 (3/15)	744	11.8 (2/17)	388	20.0 (2/10)	318
IgG1	B	V1V2	gp70-TT31P.2F10.2792 V1V2	RV305_wk0	0.0 (0/20)		0.0 (0/18)		0.0 (0/19)		0.0 (0/13)	
IgG1	B	V1V2	gp70-TT31P.2F10.2792 V1V2	RV305_wk2	45.0 (9/20)	430	38.9 (7/18)	897	0.0 (0/19)		0.0 (0/13)	
IgG1	B	V1V2	gp70-TT31P.2F10.2792 V1V2	RV305_wk24	0.0 (0/20)		0.0 (0/18)		0.0 (0/19)		0.0 (0/13)	
IgG1	B	V1V2	gp70-TT31P.2F10.2792 V1V2	RV305_wk26	10.0 (2/20)	124	22.2 (4/18)	222	0.0 (0/19)		0.0 (0/13)	
IgG1	B	V1V2	gp70-TT31P.2F10.2792 V1V2	RV305_wk48	0.0 (0/20)		0.0 (0/18)		0.0 (0/19)		0.0 (0/13)	
IgG1	B	V1V2	gp70-TT31P.2F10.2792 V1V2	RV305_wk72	0.0 (0/20)		0.0 (0/18)		0.0 (0/18)		0.0 (0/13)	
IgG1	B	V1V2	gp70_B.CaseA2 V1/V2/169K	RV144_wk26	35.3 (6/17)	173	20.0 (3/15)	1137	29.4 (5/17)	407	30.0 (3/10)	288
IgG1	B	V1V2	gp70_B.CaseA2 V1/V2/169K	RV305_wk0	0.0 (0/20)		0.0 (0/18)		0.0 (0/19)		0.0 (0/13)	
IgG1	B	V1V2	gp70_B.CaseA2 V1/V2/169K	RV305_wk2	70.0 (14/20)	1925	72.2 (13/18)	2383	0.0 (0/19)		0.0 (0/13)	
IgG1	B	V1V2	gp70_B.CaseA2 V1/V2/169K	RV305_wk24	10.0 (2/20)	288	5.6 (1/18)	365	0.0 (0/19)		0.0 (0/13)	
IgG1	B	V1V2	gp70_B.CaseA2 V1/V2/169K	RV305_wk26	45.0 (9/20)	259	38.9 (7/18)	259	0.0 (0/19)		0.0 (0/13)	
IgG1	B	V1V2	gp70_B.CaseA2 V1/V2/169K	RV305_wk48	5.0 (1/20)	625	5.6 (1/18)	211	0.0 (0/19)		0.0 (0/13)	
IgG1	B	V1V2	gp70_B.CaseA2 V1/V2/169K	RV305_wk72	5.0 (1/20)	170	5.6 (1/18)	173	0.0 (0/18)		0.0 (0/13)	
IgG1	B	V1V2	gp70_B.CaseA_V1_V2	RV144_wk26	23.5 (4/17)	317	20.0 (3/15)	556	17.6 (3/17)	335	20.0 (2/10)	523
IgG1	B	V1V2	gp70_B.CaseA_V1_V2	RV305_wk0	0.0 (0/20)		0.0 (0/18)		0.0 (0/19)		0.0 (0/13)	
IgG1	B	V1V2	gp70_B.CaseA_V1_V2	RV305_wk2	70.0 (14/20)	471	38.9 (7/18)	2921	0.0 (0/19)		0.0 (0/13)	
IgG1	B	V1V2	gp70_B.CaseA_V1_V2	RV305_wk24	0.0 (0/20)		0.0 (0/18)		0.0 (0/19)		0.0 (0/13)	
IgG1	B	V1V2	gp70_B.CaseA_V1_V2	RV305_wk26	30.0 (6/20)	225	22.2 (4/18)	820	0.0 (0/19)		0.0 (0/13)	
IgG1	B	V1V2	gp70_B.CaseA_V1_V2	RV305_wk48	0.0 (0/20)		0.0 (0/18)		0.0 (0/19)		0.0 (0/13)	
IgG1	B	V1V2	gp70_B.CaseA_V1_V2	RV305_wk72	5.0 (1/20)	240	0.0 (0/18)		0.0 (0/18)		0.0 (0/13)	
IgG1	C	V1V2	C.1086C_V1_V2 Tags	RV144_wk26	62.5 (10/16)	1734	66.7 (10/15)	599	70.6 (12/17)	562	90.0 (9/10)	657
IgG1	C	V1V2	C.1086C_V1_V2 Tags	RV305_wk0	0.0 (0/20)		0.0 (0/18)		0.0 (0/19)		0.0 (0/13)	
IgG1	C	V1V2	C.1086C_V1_V2 Tags	RV305_wk2	100 (20/20)	3909	94.4 (17/18)	2055	0.0 (0/19)		0.0 (0/13)	
IgG1	C	V1V2	C.1086C_V1_V2 Tags	RV305_wk24	15.0 (3/20)	514	22.2 (4/18)	595	0.0 (0/19)		0.0 (0/13)	
IgG1	C	V1V2	C.1086C_V1_V2 Tags	RV305_wk26	90.0 (18/20)	788	77.8 (14/18)	856	0.0 (0/19)		0.0 (0/13)	
IgG1	C	V1V2	C.1086C_V1_V2 Tags	RV305_wk48	15.0 (3/20)	350	22.2 (4/18)	429	0.0 (0/19)		0.0 (0/13)	
IgG1	C	V1V2	C.1086C_V1_V2 Tags	RV305_wk72	10.0 (2/20)	209	16.7 (3/18)	193	0.0 (0/18)		0.0 (0/13)	

S7 Table continued

					Group 1: Combination		Group 2: AIDSVAX B/E		Group 3: ALVAC-HIV		RV305_Placebo Group	
Isotype	Clade	Env Region	Antigen	Study Week	Response Rate (Responders/ Total)	Median MFI Responders	Response Rate (Responders/ Total)	Median MFI Responders	Response Rate (Responders/ Total)	Median MFI Responders	Response Rate (Responders/ Total)	Median MFI Responders
IgG1	C	V1V2	gp70-001428.2.42 V1V2	RV144_wk26	17.6 (3/17)	160	20.0 (3/15)	472	11.8 (2/17)	398	10.0 (1/10)	230
IgG1	C	V1V2	gp70-001428.2.42 V1V2	RV305_wk0	0.0 (0/20)		0.0 (0/18)		0.0 (0/19)		0.0 (0/13)	
IgG1	C	V1V2	gp70-001428.2.42 V1V2	RV305_wk2	80.0 (16/20)	573	83.3 (15/18)	1113	0.0 (0/19)		0.0 (0/13)	
IgG1	C	V1V2	gp70-001428.2.42 V1V2	RV305_wk24	0.0 (0/20)		5.6 (1/18)	211	0.0 (0/19)		0.0 (0/13)	
IgG1	C	V1V2	gp70-001428.2.42 V1V2	RV305_wk26	45.0 (9/20)	255	44.4 (8/18)	460	0.0 (0/19)		0.0 (0/13)	
IgG1	C	V1V2	gp70-001428.2.42 V1V2	RV305_wk48	5.0 (1/20)	137	5.6 (1/18)	146	0.0 (0/19)		0.0 (0/13)	
IgG1	C	V1V2	gp70-001428.2.42 V1V2	RV305_wk72	0.0 (0/20)		0.0 (0/18)		0.0 (0/18)		0.0 (0/13)	
IgG1	C	V1V2	gp70-7060101641 V1V2	RV144_wk26	23.5 (4/17)	606	20.0 (3/15)	1041	29.4 (5/17)	391	20.0 (2/10)	830
IgG1	C	V1V2	gp70-7060101641 V1V2	RV305_wk0	0.0 (0/20)		0.0 (0/18)		0.0 (0/19)		0.0 (0/13)	
IgG1	C	V1V2	gp70-7060101641 V1V2	RV305_wk2	95.0 (19/20)	1317	94.4 (17/18)	1610	0.0 (0/19)		0.0 (0/13)	
IgG1	C	V1V2	gp70-7060101641 V1V2	RV305_wk24	15.0 (3/20)	268	5.6 (1/18)	2496	0.0 (0/19)		0.0 (0/13)	
IgG1	C	V1V2	gp70-7060101641 V1V2	RV305_wk26	70.0 (14/20)	478	66.7 (12/18)	759	0.0 (0/19)		0.0 (0/13)	
IgG1	C	V1V2	gp70-7060101641 V1V2	RV305_wk48	15.0 (3/20)	250	5.6 (1/18)	2216	0.0 (0/19)		7.7 (1/13)	259
IgG1	C	V1V2	gp70-7060101641 V1V2	RV305_wk72	10.0 (2/20)	234	5.6 (1/18)	1675	0.0 (0/18)		0.0 (0/13)	
IgG1	C	V1V2	gp70-96ZM651.02 V1v2	RV144_wk26	41.2 (7/17)	462	53.3 (8/15)	531	41.2 (7/17)	1806	60.0 (6/10)	314
IgG1	C	V1V2	gp70-96ZM651.02 V1v2	RV305_wk0	0.0 (0/20)		0.0 (0/18)		0.0 (0/19)		0.0 (0/13)	
IgG1	C	V1V2	gp70-96ZM651.02 V1v2	RV305_wk2	95.0 (19/20)	6210	100 (18/18)	13565	0.0 (0/19)		0.0 (0/13)	
IgG1	C	V1V2	gp70-96ZM651.02 V1v2	RV305_wk24	20.0 (4/20)	1114	11.1 (2/18)	4604	0.0 (0/19)		0.0 (0/13)	
IgG1	C	V1V2	gp70-96ZM651.02 V1v2	RV305_wk26	70.0 (14/20)	722	88.9 (16/18)	891	10.5 (2/19)	109	0.0 (0/13)	
IgG1	C	V1V2	gp70-96ZM651.02 V1v2	RV305_wk48	15.0 (3/20)	1696	16.7 (3/18)	1114	5.3 (1/19)	314	0.0 (0/13)	
IgG1	C	V1V2	gp70-96ZM651.02 V1v2	RV305_wk72	15.0 (3/20)	446	11.1 (2/18)	2159	0.0 (0/18)		0.0 (0/13)	
IgG1	C	V1V2	gp70-BF1266_431a_V1V2	RV144_wk26	17.6 (3/17)	162	20.0 (3/15)	1674	11.8 (2/17)	1759	20.0 (2/10)	347
IgG1	C	V1V2	gp70-BF1266_431a_V1V2	RV305_wk0	0.0 (0/20)		0.0 (0/18)		5.3 (1/19)	202	0.0 (0/13)	
IgG1	C	V1V2	gp70-BF1266_431a_V1V2	RV305_wk2	50.0 (10/20)	783	55.6 (10/18)	3435	5.3 (1/19)	168	0.0 (0/13)	
IgG1	C	V1V2	gp70-BF1266_431a_V1V2	RV305_wk24	0.0 (0/20)		5.6 (1/18)	460	5.3 (1/19)	150	0.0 (0/13)	
IgG1	C	V1V2	gp70-BF1266_431a_V1V2	RV305_wk26	25.0 (5/20)	241	33.3 (6/18)	387	5.3 (1/19)	177	0.0 (0/13)	
IgG1	C	V1V2	gp70-BF1266_431a_V1V2	RV305_wk48	5.0 (1/20)	249	5.6 (1/18)	373	5.3 (1/19)	263	0.0 (0/13)	
IgG1	C	V1V2	gp70-BF1266_431a_V1V2	RV305_wk72	0.0 (0/20)		5.6 (1/18)	189	5.6 (1/18)	215	0.0 (0/13)	
IgG1	C	V1V2	gp70-CAP210.2.00.E8 V1V2	RV144_wk26	0.0 (0/17)		6.7 (1/15)	103	5.9 (1/17)	138	0.0 (0/10)	
IgG1	C	V1V2	gp70-CAP210.2.00.E8 V1V2	RV305_wk0	0.0 (0/20)		0.0 (0/18)		0.0 (0/19)		0.0 (0/13)	
IgG1	C	V1V2	gp70-CAP210.2.00.E8 V1V2	RV305_wk2	20.0 (4/20)	486	22.2 (4/18)	1185	0.0 (0/19)		0.0 (0/13)	
IgG1	C	V1V2	gp70-CAP210.2.00.E8 V1V2	RV305_wk24	0.0 (0/20)		0.0 (0/18)		0.0 (0/19)		0.0 (0/13)	
IgG1	C	V1V2	gp70-CAP210.2.00.E8 V1V2	RV305_wk26	0.0 (0/20)		0.0 (0/18)		0.0 (0/19)		0.0 (0/13)	
IgG1	C	V1V2	gp70-CAP210.2.00.E8 V1V2	RV305_wk48	0.0 (0/20)		0.0 (0/18)		0.0 (0/19)		0.0 (0/13)	
IgG1	C	V1V2	gp70-CAP210.2.00.E8 V1V2	RV305_wk72	0.0 (0/20)		0.0 (0/18)		0.0 (0/18)		0.0 (0/13)	

S7 Table continued

					Group 1: Combination		Group 2: AIDSVAX B/E		Group 3: ALVAC-HIV		RV305_Placebo Group	
Isotype	Clade	Env Region	Antigen	Study Week	Response Rate (Responders/ Total)	Median MFI Responders	Response Rate (Responders/ Total)	Median MFI Responders	Response Rate (Responders/ Total)	Median MFI Responders	Response Rate (Responders/ Total)	Median MFI Responders
IgG1	C	V1V2	gp70-Ce1086_B2 V1V2	RV144_wk26	88.2 (15/17)	1852	86.7 (13/15)	1689	94.1 (16/17)	1425	100 (10/10)	1971
IgG1	C	V1V2	gp70-Ce1086_B2 V1V2	RV305_wk0	0.0 (0/20)		0.0 (0/18)		0.0 (0/19)		0.0 (0/13)	
IgG1	C	V1V2	gp70-Ce1086_B2 V1V2	RV305_wk2	100 (20/20)	5656	100 (18/18)	6176	10.5 (2/19)	143	0.0 (0/13)	
IgG1	C	V1V2	gp70-Ce1086_B2 V1V2	RV305_wk24	40.0 (8/20)	770	44.4 (8/18)	1776	0.0 (0/19)		7.7 (1/13)	278
IgG1	C	V1V2	gp70-Ce1086_B2 V1V2	RV305_wk26	95.0 (19/20)	2826	100 (18/18)	2866	5.3 (1/19)	101	0.0 (0/13)	
IgG1	C	V1V2	gp70-Ce1086_B2 V1V2	RV305_wk48	45.0 (9/20)	387	44.4 (8/18)	1591	0.0 (0/19)		7.7 (1/13)	524
IgG1	C	V1V2	gp70-Ce1086_B2 V1V2	RV305_wk72	30.0 (6/20)	851	27.8 (5/18)	2853	0.0 (0/18)		0.0 (0/13)	
IgG1	C	V1V2	gp70-TV1.21 V1V2	RV144_wk26	35.3 (6/17)	302	20.0 (3/15)	2019	17.6 (3/17)	728	30.0 (3/10)	517
IgG1	C	V1V2	gp70-TV1.21 V1V2	RV305_wk0	0.0 (0/20)		0.0 (0/18)		0.0 (0/19)		0.0 (0/13)	
IgG1	C	V1V2	gp70-TV1.21 V1V2	RV305_wk2	60.0 (12/20)	532	50.0 (9/18)	1149	0.0 (0/19)		0.0 (0/13)	
IgG1	C	V1V2	gp70-TV1.21 V1V2	RV305_wk24	0.0 (0/20)		0.0 (0/18)		0.0 (0/19)		0.0 (0/13)	
IgG1	C	V1V2	gp70-TV1.21 V1V2	RV305_wk26	25.0 (5/20)	143	27.8 (5/18)	621	0.0 (0/19)		0.0 (0/13)	
IgG1	C	V1V2	gp70-TV1.21 V1V2	RV305_wk48	0.0 (0/20)		0.0 (0/18)		0.0 (0/19)		0.0 (0/13)	
IgG1	C	V1V2	gp70-TV1.21 V1V2	RV305_wk72	0.0 (0/20)		0.0 (0/18)		0.0 (0/18)		0.0 (0/13)	
IgG1	CRF01_AE	V1V2	AE.A244 V1V2 tags	RV144_wk26	94.1 (16/17)	5265	93.3 (14/15)	2478	94.1 (16/17)	1782	100 (10/10)	3107
IgG1	CRF01_AE	V1V2	AE.A244 V1V2 tags	RV305_wk0	0.0 (0/20)		0.0 (0/18)		0.0 (0/19)		0.0 (0/13)	
IgG1	CRF01_AE	V1V2	AE.A244 V1V2 tags	RV305_wk2	100 (20/20)	11499	100 (18/18)	7624	0.0 (0/19)		0.0 (0/13)	
IgG1	CRF01_AE	V1V2	AE.A244 V1V2 tags	RV305_wk24	25.0 (5/20)	565	23.5 (4/17)	2105	0.0 (0/19)		0.0 (0/13)	
IgG1	CRF01_AE	V1V2	AE.A244 V1V2 tags	RV305_wk26	95.0 (19/20)	2601	100 (18/18)	2432	0.0 (0/19)		0.0 (0/13)	
IgG1	CRF01_AE	V1V2	AE.A244 V1V2 tags	RV305_wk48	30.0 (6/20)	375	33.3 (6/18)	1137	0.0 (0/19)		0.0 (0/13)	
IgG1	CRF01_AE	V1V2	AE.A244 V1V2 tags	RV305_wk72	25.0 (5/20)	148	22.2 (4/18)	785	0.0 (0/18)		0.0 (0/13)	
IgG1	CRF01_AE	V1V2	gp70-C2101.c01_V1V2	RV144_wk26	56.3 (9/16)	2846	73.3 (11/15)	895	47.1 (8/17)	2248	100 (10/10)	635
IgG1	CRF01_AE	V1V2	gp70-C2101.c01_V1V2	RV305_wk0	0.0 (0/20)		0.0 (0/18)		0.0 (0/19)		0.0 (0/13)	
IgG1	CRF01_AE	V1V2	gp70-C2101.c01_V1V2	RV305_wk2	100 (20/20)	10407	100 (18/18)	9983	10.5 (2/19)	365	0.0 (0/13)	
IgG1	CRF01_AE	V1V2	gp70-C2101.c01_V1V2	RV305_wk24	50.0 (10/20)	1155	44.4 (8/18)	1187	0.0 (0/19)		0.0 (0/13)	
IgG1	CRF01_AE	V1V2	gp70-C2101.c01_V1V2	RV305_wk26	100 (20/20)	3509	100 (18/18)	4245	15.8 (3/19)	368	0.0 (0/13)	
IgG1	CRF01_AE	V1V2	gp70-C2101.c01_V1V2	RV305_wk48	50.0 (10/20)	541	44.4 (8/18)	1199	10.5 (2/19)	208	0.0 (0/13)	
IgG1	CRF01_AE	V1V2	gp70-C2101.c01_V1V2	RV305_wk72	35.0 (7/20)	565	38.9 (7/18)	669	5.6 (1/18)	150	0.0 (0/13)	
IgG1	CRF01_AE	V1V2	gp70-CM244.ec1 V1V2	RV144_wk26	93.8 (15/16)	4689	93.3 (14/15)	4228	100 (17/17)	3348	100 (10/10)	5424
IgG1	CRF01_AE	V1V2	gp70-CM244.ec1 V1V2	RV305_wk0	0.0 (0/20)		0.0 (0/18)		0.0 (0/19)		0.0 (0/13)	
IgG1	CRF01_AE	V1V2	gp70-CM244.ec1 V1V2	RV305_wk2	100 (20/20)	11838	100 (18/18)	12496	15.8 (3/19)	119	0.0 (0/13)	
IgG1	CRF01_AE	V1V2	gp70-CM244.ec1 V1V2	RV305_wk24	50.0 (10/20)	1442	50.0 (9/18)	734	0.0 (0/19)		0.0 (0/13)	
IgG1	CRF01_AE	V1V2	gp70-CM244.ec1 V1V2	RV305_wk26	95.0 (19/20)	7189	100 (18/18)	6642	10.5 (2/19)	182	0.0 (0/13)	
IgG1	CRF01_AE	V1V2	gp70-CM244.ec1 V1V2	RV305_wk48	55.0 (11/20)	721	50.0 (9/18)	1005	5.3 (1/19)	215	0.0 (0/13)	
IgG1	CRF01_AE	V1V2	gp70-CM244.ec1 V1V2	RV305_wk72	40.0 (8/20)	992	38.9 (7/18)	3287	0.0 (0/18)		0.0 (0/13)	

S7 Table continued

					Group 1: Combination		Group 2: AIDSVAX B/E		Group 3: ALVAC-HIV		RV305_Placebo Group	
Isotype	Clade	Env Region	Antigen	Study Week	Response Rate (Responders/ Total)	Median MFI Responders	Response Rate (Responders/ Total)	Median MFI Responders	Response Rate (Responders/ Total)	Median MFI Responders	Response Rate (Responders/ Total)	Median MFI Responders
IgG1	CRF07_BC	V1V2	gp70-BJOX002000.03.2 V1V2	RV144_wk26	47.1 (8/17)	478	46.7 (7/15)	352	47.1 (8/17)	843	60.0 (6/10)	445
IgG1	CRF07_BC	V1V2	gp70-BJOX002000.03.2 V1V2	RV305_wk0	0.0 (0/20)		0.0 (0/18)		0.0 (0/19)		0.0 (0/13)	
IgG1	CRF07_BC	V1V2	gp70-BJOX002000.03.2 V1V2	RV305_wk2	100 (20/20)	2968	94.4 (17/18)	5911	0.0 (0/19)		0.0 (0/13)	
IgG1	CRF07_BC	V1V2	gp70-BJOX002000.03.2 V1V2	RV305_wk24	20.0 (4/20)	719	22.2 (4/18)	332	0.0 (0/19)		0.0 (0/13)	
IgG1	CRF07_BC	V1V2	gp70-BJOX002000.03.2 V1V2	RV305_wk26	85.0 (17/20)	468	88.9 (16/18)	1056	0.0 (0/19)		0.0 (0/13)	
IgG1	CRF07_BC	V1V2	gp70-BJOX002000.03.2 V1V2	RV305_wk48	20.0 (4/20)	601	16.7 (3/18)	376	0.0 (0/19)		0.0 (0/13)	
IgG1	CRF07_BC	V1V2	gp70-BJOX002000.03.2 V1V2	RV305_wk72	15.0 (3/20)	627	11.1 (2/18)	554	0.0 (0/18)		0.0 (0/13)	
IgG1	CRF01_AE	V2	AE.A244 V2 tags/293F	RV144_wk26	29.4 (5/17)	393	26.7 (4/15)	668	47.1 (8/17)	212	70.0 (7/10)	110
IgG1	CRF01_AE	V2	AE.A244 V2 tags/293F	RV305_wk0	0.0 (0/20)		0.0 (0/18)		0.0 (0/19)		0.0 (0/13)	
IgG1	CRF01_AE	V2	AE.A244 V2 tags/293F	RV305_wk2	90.0 (18/20)	1237	88.9 (16/18)	1041	0.0 (0/19)		0.0 (0/13)	
IgG1	CRF01_AE	V2	AE.A244 V2 tags/293F	RV305_wk24	5.3 (1/19)	405	22.2 (4/18)	291	0.0 (0/19)		0.0 (0/13)	
IgG1	CRF01_AE	V2	AE.A244 V2 tags/293F	RV305_wk26	60.0 (12/20)	251	44.4 (8/18)	359	0.0 (0/19)		0.0 (0/13)	
IgG1	CRF01_AE	V2	AE.A244 V2 tags/293F	RV305_wk48	10.0 (2/20)	178	22.2 (4/18)	237	0.0 (0/19)		0.0 (0/13)	
IgG1	CRF01_AE	V2	AE.A244 V2 tags/293F	RV305_wk72	5.0 (1/20)	907	11.1 (2/18)	193	0.0 (0/18)		0.0 (0/13)	
IgG1	B	V3	B.MN V3 gp70	RV144_wk26	17.6 (3/17)	148	20.0 (3/15)	154	17.6 (3/17)	149	0.0 (0/10)	
IgG1	B	V3	B.MN V3 gp70	RV305_wk0	0.0 (0/20)		0.0 (0/18)		0.0 (0/19)		0.0 (0/13)	
IgG1	B	V3	B.MN V3 gp70	RV305_wk2	100 (20/20)	488	88.9 (16/18)	597	0.0 (0/19)		0.0 (0/13)	
IgG1	B	V3	B.MN V3 gp70	RV305_wk24	10.0 (2/20)	147	0.0 (0/18)		0.0 (0/19)		0.0 (0/13)	
IgG1	B	V3	B.MN V3 gp70	RV305_wk26	55.0 (11/20)	247	66.7 (12/18)	298	0.0 (0/19)		0.0 (0/13)	
IgG1	B	V3	B.MN V3 gp70	RV305_wk48	5.0 (1/20)	171	0.0 (0/18)		0.0 (0/19)		0.0 (0/13)	
IgG1	B	V3	B.MN V3 gp70	RV305_wk72	0.0 (0/20)		0.0 (0/18)		0.0 (0/18)		0.0 (0/13)	
IgG1	N/A	CD4i	HxB2 new 8b core 6x His	RV144_wk26	5.9 (1/17)	164	13.3 (2/15)	121	5.9 (1/17)	116	10.0 (1/10)	386
IgG1	N/A	CD4i	HxB2 new 8b core 6x His	RV305_wk0	0.0 (0/20)		0.0 (0/18)		0.0 (0/19)		0.0 (0/13)	
IgG1	N/A	CD4i	HxB2 new 8b core 6x His	RV305_wk2	100 (20/20)	323	100 (18/18)	296	0.0 (0/19)		0.0 (0/13)	
IgG1	N/A	CD4i	HxB2 new 8b core 6x His	RV305_wk24	15.0 (3/20)	129	0.0 (0/18)		0.0 (0/19)		0.0 (0/13)	
IgG1	N/A	CD4i	HxB2 new 8b core 6x His	RV305_wk26	55.0 (11/20)	252	38.9 (7/18)	197	0.0 (0/19)		0.0 (0/13)	
IgG1	N/A	CD4i	HxB2 new 8b core 6x His	RV305_wk48	5.0 (1/20)	196	5.6 (1/18)	114	0.0 (0/19)		0.0 (0/13)	
IgG1	N/A	CD4i	HxB2 new 8b core 6x His	RV305_wk72	5.0 (1/20)	147	0.0 (0/18)		0.0 (0/18)		0.0 (0/13)	
IgG1	N/A	CD4i	YU2 gp120 WT	RV144_wk26	0.0 (0/17)		6.7 (1/15)	167	6.3 (1/16)	102	12.5 (1/8)	191
IgG1	N/A	CD4i	YU2 gp120 WT	RV305_wk0	0.0 (0/20)		0.0 (0/18)		0.0 (0/19)		0.0 (0/13)	
IgG1	N/A	CD4i	YU2 gp120 WT	RV305_wk2	0.0 (0/19)		5.9 (1/17)	396	0.0 (0/19)		0.0 (0/13)	
IgG1	N/A	CD4i	YU2 gp120 WT	RV305_wk24	5.3 (1/19)	340	0.0 (0/18)		0.0 (0/19)		0.0 (0/13)	
IgG1	N/A	CD4i	YU2 gp120 WT	RV305_wk26	0.0 (0/19)		5.9 (1/17)	540	0.0 (0/19)		0.0 (0/13)	
IgG1	N/A	CD4i	YU2 gp120 WT	RV305_wk48	15.0 (3/20)	231	5.6 (1/18)	132	0.0 (0/19)		0.0 (0/13)	
IgG1	N/A	CD4i	YU2 gp120 WT	RV305_wk72	5.0 (1/20)	142	0.0 (0/18)		0.0 (0/18)		0.0 (0/13)	

S7 Table continued

					Group 1: Combination		Group 2: AIDSVAX B/E		Group 3: ALVAC-HIV		RV305_Placebo Group	
**Isotype**	**Clade**	**Env Region**	**Antigen**	**Study Week**	**Response Rate (Responders/ Total)**	**Median MFI Responders**	**Response Rate (Responders/ Total)**	**Median MFI Responders**	**Response Rate (Responders/ Total)**	**Median MFI Responders**	**Response Rate (Responders/ Total)**	**Median MFI Responders**
IgG1	N/A	CD4bs	RSC3	RV144_wk26	0.0 (0/17)		0.0 (0/15)		0.0 (0/17)		0.0 (0/10)	
IgG1	N/A	CD4bs	RSC3	RV305_wk0	0.0 (0/20)		0.0 (0/18)		0.0 (0/19)		0.0 (0/13)	
IgG1	N/A	CD4bs	RSC3	RV305_wk2	0.0 (0/20)		0.0 (0/18)		0.0 (0/19)		0.0 (0/13)	
IgG1	N/A	CD4bs	RSC3	RV305_wk24	0.0 (0/20)		0.0 (0/18)		0.0 (0/19)		0.0 (0/13)	
IgG1	N/A	CD4bs	RSC3	RV305_wk26	0.0 (0/20)		0.0 (0/18)		0.0 (0/19)		0.0 (0/13)	
IgG1	N/A	CD4bs	RSC3	RV305_wk48	0.0 (0/20)		0.0 (0/18)		0.0 (0/19)		0.0 (0/13)	
IgG1	N/A	CD4bs	RSC3	RV305_wk72	0.0 (0/20)		0.0 (0/18)		0.0 (0/18)		0.0 (0/13)	
IgG1	N/A	CD4bs	RSC3_P363Npair	RV144_wk26	0.0 (0/17)		0.0 (0/15)		0.0 (0/17)		0.0 (0/10)	
IgG1	N/A	CD4bs	RSC3_P363Npair	RV305_wk0	0.0 (0/20)		0.0 (0/18)		0.0 (0/19)		0.0 (0/13)	
IgG1	N/A	CD4bs	RSC3_P363Npair	RV305_wk2	0.0 (0/20)		0.0 (0/18)		0.0 (0/19)		0.0 (0/13)	
IgG1	N/A	CD4bs	RSC3_P363Npair	RV305_wk24	0.0 (0/20)		0.0 (0/18)		0.0 (0/19)		0.0 (0/13)	
IgG1	N/A	CD4bs	RSC3_P363Npair	RV305_wk26	0.0 (0/20)		0.0 (0/18)		0.0 (0/19)		0.0 (0/13)	
IgG1	N/A	CD4bs	RSC3_P363Npair	RV305_wk48	0.0 (0/20)		0.0 (0/18)		0.0 (0/19)		0.0 (0/13)	
IgG1	N/A	CD4bs	RSC3_P363Npair	RV305_wk72	0.0 (0/20)		0.0 (0/18)		0.0 (0/18)		0.0 (0/13)	
IgG1	N/A	Gag (non-Env)	p24	RV144_wk26	41.2 (7/17)	1556	53.3 (8/15)	3010	35.3 (6/17)	1601	40.0 (4/10)	1265
IgG1	N/A	Gag (non-Env)	p24	RV305_wk0	10.0 (2/20)	423	0.0 (0/18)		10.5 (2/19)	228	15.4 (2/13)	146
IgG1	N/A	Gag (non-Env)	p24	RV305_wk2	25.0 (5/20)	5097	0.0 (0/18)		31.6 (6/19)	406	7.7 (1/13)	136
IgG1	N/A	Gag (non-Env)	p24	RV305_wk24	30.0 (6/20)	434	0.0 (0/18)		21.1 (4/19)	437	7.7 (1/13)	119
IgG1	N/A	Gag (non-Env)	p24	RV305_wk26	50.0 (10/20)	834	0.0 (0/18)		33.3 (6/18)	1958	7.7 (1/13)	142
IgG1	N/A	Gag (non-Env)	p24	RV305_wk48	30.0 (6/20)	937	0.0 (0/18)		31.6 (6/19)	587	7.7 (1/13)	135
IgG1	N/A	Gag (non-Env)	p24	RV305_wk72	25.0 (5/20)	914	0.0 (0/18)		11.1 (2/18)	133	7.7 (1/13)	128
IgG1	N/A	CD4bs	RSC3	RV144_wk26	0.0 (0/17)		0.0 (0/15)		0.0 (0/17)		0.0 (0/10)	
IgG1	N/A	CD4bs	RSC3	RV305_wk0	0.0 (0/20)		0.0 (0/18)		0.0 (0/19)		0.0 (0/13)	
IgG1	N/A	CD4bs	RSC3	RV305_wk2	0.0 (0/20)		0.0 (0/18)		0.0 (0/19)		0.0 (0/13)	
IgG1	N/A	CD4bs	RSC3	RV305_wk24	0.0 (0/20)		0.0 (0/18)		0.0 (0/19)		0.0 (0/13)	
IgG1	N/A	CD4bs	RSC3	RV305_wk26	0.0 (0/20)		0.0 (0/18)		0.0 (0/19)		0.0 (0/13)	
IgG1	N/A	CD4bs	RSC3	RV305_wk48	0.0 (0/20)		0.0 (0/18)		0.0 (0/19)		0.0 (0/13)	
IgG1	N/A	CD4bs	RSC3	RV305_wk72	0.0 (0/20)		0.0 (0/18)		0.0 (0/18)		0.0 (0/13)	
IgG1	N/A	CD4bs	RSC3_P363Npair	RV144_wk26	0.0 (0/17)		0.0 (0/15)		0.0 (0/17)		0.0 (0/10)	
IgG1	N/A	CD4bs	RSC3_P363Npair	RV305_wk0	0.0 (0/20)		0.0 (0/18)		0.0 (0/19)		0.0 (0/13)	
IgG1	N/A	CD4bs	RSC3_P363Npair	RV305_wk2	0.0 (0/20)		0.0 (0/18)		0.0 (0/19)		0.0 (0/13)	
IgG1	N/A	CD4bs	RSC3_P363Npair	RV305_wk24	0.0 (0/20)		0.0 (0/18)		0.0 (0/19)		0.0 (0/13)	
