# Supplementary material for: Viral vector delivered immunogen focuses HIV-1 antibody specificity and increases durability of the circulating antibody recall response
Source: PLoS Pathog. 2023 May 31;19(5):e1011359. doi: 10.1371/journal.ppat.1011359 (PMC10284421; doi:10.1371/journal.ppat.1011359)
Supplement: S8 Table — (PDF) [file ppat.1011359.s021.pdf]

S8 Table. BAMA plasma binding IgG3 response rates and group median binding magnitudes (MFI) to gp120, gp140, V1V2, V3, CD4 inducible, CD4 binding site, and Gag HIV-1 regions.

					Group 1: Combination		Group 2: AIDSVAX B/E		Group 3: ALVAC-HIV		RV305_Placebo Group	
Isotype	Clade	Env Region	Antigen	Study Week	Response Rate *(Responders/ Total)*	Median MFI *Responders*	Response Rate *(Responders/ Total)*	Median MFI *Responders*	Response Rate *(Responders/ Total)*	Median MFI *Responders*	Response Rate *(Responders/ Total)*	Median MFI *Responders*
IgG3	A	gp120	51802_D11gp120.avi/293F	RV144_wk26	5.9 (1/17)	282	6.7 (1/15)	210	5.9 (1/17)	101	0.0 (0/10)	
IgG3	A	gp120	51802_D11gp120.avi/293F	RV305_wk0	0.0 (0/20)		5.6 (1/18)	174	0.0 (0/19)		0.0 (0/13)	
IgG3	A	gp120	51802_D11gp120.avi/293F	RV305_wk2	10.0 (2/20)	381	22.2 (4/18)	244	0.0 (0/19)		0.0 (0/13)	
IgG3	A	gp120	51802_D11gp120.avi/293F	RV305_wk24	0.0 (0/20)		5.6 (1/18)	159	0.0 (0/19)		0.0 (0/13)	
IgG3	A	gp120	51802_D11gp120.avi/293F	RV305_wk26	0.0 (0/20)		5.6 (1/18)	123	0.0 (0/19)		0.0 (0/13)	
IgG3	A	gp120	51802_D11gp120.avi/293F	RV305_wk48	0.0 (0/20)		5.6 (1/18)	162	0.0 (0/19)		0.0 (0/13)	
IgG3	A	gp120	51802_D11gp120.avi/293F	RV305_wk72	0.0 (0/20)		5.6 (1/18)	146	0.0 (0/18)		0.0 (0/13)	
IgG3	B	gp120	B.6240_D11gp120/293F	RV144_wk26	29.4 (5/17)	240	46.7 (7/15)	163	23.5 (4/17)	203	0.0 (0/10)	
IgG3	B	gp120	B.6240_D11gp120/293F	RV305_wk0	0.0 (0/20)		0.0 (0/18)		0.0 (0/19)		0.0 (0/13)	
IgG3	B	gp120	B.6240_D11gp120/293F	RV305_wk2	25.0 (5/20)	387	16.7 (3/18)	561	0.0 (0/19)		0.0 (0/13)	
IgG3	B	gp120	B.6240_D11gp120/293F	RV305_wk24	0.0 (0/20)		0.0 (0/18)		0.0 (0/19)		0.0 (0/13)	
IgG3	B	gp120	B.6240_D11gp120/293F	RV305_wk26	20.0 (4/20)	234	5.6 (1/18)	190	5.3 (1/19)	418	0.0 (0/13)	
IgG3	B	gp120	B.6240_D11gp120/293F	RV305_wk48	0.0 (0/20)		0.0 (0/18)		0.0 (0/19)		0.0 (0/13)	
IgG3	B	gp120	B.6240_D11gp120/293F	RV305_wk72	0.0 (0/20)		0.0 (0/18)		0.0 (0/18)		0.0 (0/13)	
IgG3	B	gp120	BORI_D11gp120.avi/293F	RV144_wk26	17.6 (3/17)	160	13.3 (2/15)	299	11.8 (2/17)	202	20.0 (2/10)	255
IgG3	B	gp120	BORI_D11gp120.avi/293F	RV305_wk0	0.0 (0/20)		0.0 (0/18)		0.0 (0/19)		0.0 (0/13)	
IgG3	B	gp120	BORI_D11gp120.avi/293F	RV305_wk2	10.0 (2/20)	496	22.2 (4/18)	305	0.0 (0/19)		0.0 (0/13)	
IgG3	B	gp120	BORI_D11gp120.avi/293F	RV305_wk24	0.0 (0/20)		0.0 (0/18)		0.0 (0/19)		0.0 (0/13)	
IgG3	B	gp120	BORI_D11gp120.avi/293F	RV305_wk26	5.0 (1/20)	122	5.6 (1/18)	114	0.0 (0/19)		0.0 (0/13)	
IgG3	B	gp120	BORI_D11gp120.avi/293F	RV305_wk48	0.0 (0/20)		0.0 (0/18)		0.0 (0/19)		0.0 (0/13)	
IgG3	B	gp120	BORI_D11gp120.avi/293F	RV305_wk72	0.0 (0/20)		0.0 (0/18)		0.0 (0/18)		0.0 (0/13)	
IgG3	B	gp120	MN gp120 gDneg/293F	RV144_wk26	64.7 (11/17)	733	86.7 (13/15)	690	70.6 (12/17)	343	60.0 (6/10)	405
IgG3	B	gp120	MN gp120 gDneg/293F	RV305_wk0	5.0 (1/20)	297	5.6 (1/18)	231	0.0 (0/19)		0.0 (0/13)	
IgG3	B	gp120	MN gp120 gDneg/293F	RV305_wk2	45.0 (9/20)	1643	61.1 (11/18)	384	0.0 (0/19)		0.0 (0/13)	
IgG3	B	gp120	MN gp120 gDneg/293F	RV305_wk24	10.0 (2/20)	410	5.6 (1/18)	223	0.0 (0/19)		0.0 (0/13)	
IgG3	B	gp120	MN gp120 gDneg/293F	RV305_wk26	35.0 (7/20)	873	22.2 (4/18)	800	0.0 (0/19)		0.0 (0/13)	
IgG3	B	gp120	MN gp120 gDneg/293F	RV305_wk48	10.0 (2/20)	451	5.6 (1/18)	347	0.0 (0/19)		0.0 (0/13)	
IgG3	B	gp120	MN gp120 gDneg/293F	RV305_wk72	10.0 (2/20)	314	5.6 (1/18)	263	0.0 (0/18)		0.0 (0/13)	

S8 Table continued

					Group 1: Combination		Group 2: AIDSVAX B/E		Group 3: ALVAC-HIV		RV305_Placebo Group	
Isotype	Clade	Env Region	Antigen	Study Week	Response Rate (Responders/ Total)	Median MFI Responders	Response Rate (Responders/ Total)	Median MFI Responders	Response Rate (Responders/ Total)	Median MFI Responders	Response Rate (Responders/ Total)	Median MFI Responders
IgG3	B	gp120	TT31P.2792_D11gp120.avi/293F	RV144_wk26	41.2 (7/17)	404	66.7 (10/15)	221	47.1 (8/17)	215	40.0 (4/10)	262
IgG3	B	gp120	TT31P.2792_D11gp120.avi/293F	RV305_wk0	0.0 (0/20)		0.0 (0/18)		0.0 (0/19)		0.0 (0/13)	
IgG3	B	gp120	TT31P.2792_D11gp120.avi/293F	RV305_wk2	20.0 (4/20)	515	27.8 (5/18)	331	0.0 (0/19)		0.0 (0/13)	
IgG3	B	gp120	TT31P.2792_D11gp120.avi/293F	RV305_wk24	5.0 (1/20)	105	0.0 (0/18)		0.0 (0/19)		0.0 (0/13)	
IgG3	B	gp120	TT31P.2792_D11gp120.avi/293F	RV305_wk26	10.0 (2/20)	171	11.1 (2/18)	160	0.0 (0/19)		0.0 (0/13)	
IgG3	B	gp120	TT31P.2792_D11gp120.avi/293F	RV305_wk48	0.0 (0/20)		0.0 (0/18)		0.0 (0/19)		0.0 (0/13)	
IgG3	B	gp120	TT31P.2792_D11gp120.avi/293F	RV305_wk72	0.0 (0/20)		0.0 (0/18)		0.0 (0/18)		0.0 (0/13)	
IgG3	C	gp120	1086C_D7gp120.avi/293F	RV144_wk26	70.6 (12/17)	372	80.0 (12/15)	383	76.5 (13/17)	365	80.0 (8/10)	371
IgG3	C	gp120	1086C_D7gp120.avi/293F	RV305_wk0	0.0 (0/20)		0.0 (0/18)		0.0 (0/19)		7.7 (1/13)	110
IgG3	C	gp120	1086C_D7gp120.avi/293F	RV305_wk2	20.0 (4/20)	246	33.3 (6/18)	268	0.0 (0/19)		7.7 (1/13)	104
IgG3	C	gp120	1086C_D7gp120.avi/293F	RV305_wk24	0.0 (0/20)		0.0 (0/18)		0.0 (0/19)		7.7 (1/13)	125
IgG3	C	gp120	1086C_D7gp120.avi/293F	RV305_wk26	5.0 (1/20)	214	11.1 (2/18)	180	0.0 (0/19)		0.0 (0/13)	
IgG3	C	gp120	1086C_D7gp120.avi/293F	RV305_wk48	5.0 (1/20)	121	0.0 (0/18)		0.0 (0/19)		0.0 (0/13)	
IgG3	C	gp120	1086C_D7gp120.avi/293F	RV305_wk72	0.0 (0/20)		0.0 (0/18)		0.0 (0/18)		0.0 (0/13)	
IgG3	C	gp120	96ZM651.D11gp120.avi	RV144_wk26	5.9 (1/17)	110	6.7 (1/15)	262	11.8 (2/17)	201	0.0 (0/10)	
IgG3	C	gp120	96ZM651.D11gp120.avi	RV305_wk0	0.0 (0/20)		0.0 (0/18)		0.0 (0/19)		0.0 (0/13)	
IgG3	C	gp120	96ZM651.D11gp120.avi	RV305_wk2	0.0 (0/20)		11.1 (2/18)	165	0.0 (0/19)		0.0 (0/13)	
IgG3	C	gp120	96ZM651.D11gp120.avi	RV305_wk24	0.0 (0/20)		0.0 (0/18)		0.0 (0/19)		0.0 (0/13)	
IgG3	C	gp120	96ZM651.D11gp120.avi	RV305_wk26	0.0 (0/20)		0.0 (0/18)		0.0 (0/19)		0.0 (0/13)	
IgG3	C	gp120	96ZM651.D11gp120.avi	RV305_wk48	0.0 (0/20)		0.0 (0/18)		0.0 (0/19)		0.0 (0/13)	
IgG3	C	gp120	96ZM651.D11gp120.avi	RV305_wk72	0.0 (0/20)		0.0 (0/18)		0.0 (0/18)		0.0 (0/13)	
IgG3	C	gp120	TV1c8_D11gp120.avi/293F	RV144_wk26	41.2 (7/17)	253	53.3 (8/15)	578	29.4 (5/17)	135	40.0 (4/10)	274
IgG3	C	gp120	TV1c8_D11gp120.avi/293F	RV305_wk0	0.0 (0/20)		5.6 (1/18)	283	0.0 (0/19)		0.0 (0/13)	
IgG3	C	gp120	TV1c8_D11gp120.avi/293F	RV305_wk2	30.0 (6/20)	543	27.8 (5/18)	915	0.0 (0/19)		0.0 (0/13)	
IgG3	C	gp120	TV1c8_D11gp120.avi/293F	RV305_wk24	5.0 (1/20)	115	5.6 (1/18)	292	0.0 (0/19)		0.0 (0/13)	
IgG3	C	gp120	TV1c8_D11gp120.avi/293F	RV305_wk26	25.0 (5/20)	289	11.1 (2/18)	426	0.0 (0/19)		0.0 (0/13)	
IgG3	C	gp120	TV1c8_D11gp120.avi/293F	RV305_wk48	5.0 (1/20)	111	5.6 (1/18)	375	0.0 (0/19)		0.0 (0/13)	
IgG3	C	gp120	TV1c8_D11gp120.avi/293F	RV305_wk72	0.0 (0/20)		5.6 (1/18)	296	0.0 (0/18)		0.0 (0/13)	
IgG3	CRF01_AE	gp120	254008_D11gp120.avi/293F	RV144_wk26	29.4 (5/17)	200	33.3 (5/15)	190	41.2 (7/17)	148	40.0 (4/10)	236
IgG3	CRF01_AE	gp120	254008_D11gp120.avi/293F	RV305_wk0	0.0 (0/20)		0.0 (0/18)		0.0 (0/19)		0.0 (0/13)	
IgG3	CRF01_AE	gp120	254008_D11gp120.avi/293F	RV305_wk2	0.0 (0/20)		5.6 (1/18)	407	0.0 (0/19)		0.0 (0/13)	
IgG3	CRF01_AE	gp120	254008_D11gp120.avi/293F	RV305_wk24	0.0 (0/20)		0.0 (0/18)		0.0 (0/19)		0.0 (0/13)	
IgG3	CRF01_AE	gp120	254008_D11gp120.avi/293F	RV305_wk26	0.0 (0/20)		0.0 (0/18)		0.0 (0/19)		0.0 (0/13)	
IgG3	CRF01_AE	gp120	254008_D11gp120.avi/293F	RV305_wk48	0.0 (0/20)		0.0 (0/18)		0.0 (0/19)		0.0 (0/13)	
IgG3	CRF01_AE	gp120	254008_D11gp120.avi/293F	RV305_wk72	0.0 (0/20)		0.0 (0/18)		0.0 (0/18)		0.0 (0/13)	

S8 Table continued

					Group 1: Combination		Group 2: AIDSVAX B/E		Group 3: ALVAC-HIV		RV305_Placebo Group	
Isotype	Clade	Env Region	Antigen	Study Week	Response Rate (Responders/ Total)	Median MFI Responders	Response Rate (Responders/ Total)	Median MFI Responders	Response Rate (Responders/ Total)	Median MFI Responders	Response Rate (Responders/ Total)	Median MFI Responders
IgG3	CRF01_AE	gp120	92TH023 gp120 gDneg 293F mon	RV144_wk26	11.8 (2/17)	143	26.7 (4/15)	269	23.5 (4/17)	359	0.0 (0/10)	
IgG3	CRF01_AE	gp120	92TH023 gp120 gDneg 293F mon	RV305_wk0	0.0 (0/20)		0.0 (0/18)		0.0 (0/19)		0.0 (0/13)	
IgG3	CRF01_AE	gp120	92TH023 gp120 gDneg 293F mon	RV305_wk2	10.0 (2/20)	186	11.1 (2/18)	580	0.0 (0/19)		0.0 (0/13)	
IgG3	CRF01_AE	gp120	92TH023 gp120 gDneg 293F mon	RV305_wk24	0.0 (0/20)		0.0 (0/18)		0.0 (0/19)		0.0 (0/13)	
IgG3	CRF01_AE	gp120	92TH023 gp120 gDneg 293F mon	RV305_wk26	0.0 (0/20)		5.6 (1/18)	128	0.0 (0/19)		0.0 (0/13)	
IgG3	CRF01_AE	gp120	92TH023 gp120 gDneg 293F mon	RV305_wk48	0.0 (0/20)		0.0 (0/18)		0.0 (0/19)		0.0 (0/13)	
IgG3	CRF01_AE	gp120	92TH023 gp120 gDneg 293F mon	RV305_wk72	0.0 (0/20)		0.0 (0/18)		0.0 (0/18)		0.0 (0/13)	
IgG3	CRF01_AE	gp120	A244 D11gp120_avi	RV144_wk26	41.2 (7/17)	275	53.3 (8/15)	259	58.8 (10/17)	212	50.0 (5/10)	207
IgG3	CRF01_AE	gp120	A244 D11gp120_avi	RV305_wk0	0.0 (0/20)		0.0 (0/18)		0.0 (0/19)		0.0 (0/13)	
IgG3	CRF01_AE	gp120	A244 D11gp120_avi	RV305_wk2	20.0 (4/20)	205	22.2 (4/18)	330	0.0 (0/19)		0.0 (0/13)	
IgG3	CRF01_AE	gp120	A244 D11gp120_avi	RV305_wk24	0.0 (0/20)		0.0 (0/18)		0.0 (0/19)		0.0 (0/13)	
IgG3	CRF01_AE	gp120	A244 D11gp120_avi	RV305_wk26	0.0 (0/20)		5.6 (1/18)	226	0.0 (0/19)		0.0 (0/13)	
IgG3	CRF01_AE	gp120	A244 D11gp120_avi	RV305_wk48	0.0 (0/20)		5.6 (1/18)	115	0.0 (0/19)		0.0 (0/13)	
IgG3	CRF01_AE	gp120	A244 D11gp120_avi	RV305_wk72	0.0 (0/20)		0.0 (0/18)		0.0 (0/18)		0.0 (0/13)	
IgG3	CRF01_AE	gp120	CM235 gp120	RV144_wk26	5.9 (1/17)	564	20.0 (3/15)	715	11.8 (2/17)	525	0.0 (0/10)	
IgG3	CRF01_AE	gp120	CM235 gp120	RV305_wk0	5.0 (1/20)	1927	16.7 (3/18)	972	0.0 (0/19)		15.4 (2/13)	520
IgG3	CRF01_AE	gp120	CM235 gp120	RV305_wk2	5.0 (1/20)	1983	16.7 (3/18)	787	0.0 (0/19)		15.4 (2/13)	624
IgG3	CRF01_AE	gp120	CM235 gp120	RV305_wk24	5.0 (1/20)	1239	0.0 (0/18)		0.0 (0/19)		15.4 (2/13)	517
IgG3	CRF01_AE	gp120	CM235 gp120	RV305_wk26	5.0 (1/20)	1392	0.0 (0/18)		0.0 (0/19)		15.4 (2/13)	498
IgG3	CRF01_AE	gp120	CM235 gp120	RV305_wk48	10.0 (2/20)	1202	0.0 (0/18)		0.0 (0/19)		15.4 (2/13)	417
IgG3	CRF01_AE	gp120	CM235 gp120	RV305_wk72	10.0 (2/20)	694	0.0 (0/18)		0.0 (0/18)		15.4 (2/13)	1450
IgG3	CRF07_BC	gp120	BJOX002_D11gp120.avi/293F	RV144_wk26	5.9 (1/17)	508	6.7 (1/15)	123	5.9 (1/17)	154	20.0 (2/10)	112
IgG3	CRF07_BC	gp120	BJOX002_D11gp120.avi/293F	RV305_wk0	5.0 (1/20)	201	0.0 (0/18)		0.0 (0/19)		0.0 (0/13)	
IgG3	CRF07_BC	gp120	BJOX002_D11gp120.avi/293F	RV305_wk2	5.0 (1/20)	237	5.6 (1/18)	191	0.0 (0/19)		0.0 (0/13)	
IgG3	CRF07_BC	gp120	BJOX002_D11gp120.avi/293F	RV305_wk24	5.0 (1/20)	422	0.0 (0/18)		0.0 (0/19)		0.0 (0/13)	
IgG3	CRF07_BC	gp120	BJOX002_D11gp120.avi/293F	RV305_wk26	5.0 (1/20)	355	0.0 (0/18)		0.0 (0/19)		0.0 (0/13)	
IgG3	CRF07_BC	gp120	BJOX002_D11gp120.avi/293F	RV305_wk48	5.0 (1/20)	248	0.0 (0/18)		0.0 (0/19)		0.0 (0/13)	
IgG3	CRF07_BC	gp120	BJOX002_D11gp120.avi/293F	RV305_wk72	5.0 (1/20)	146	0.0 (0/18)		0.0 (0/18)		0.0 (0/13)	
IgG3	CRF07_BC	gp120	CNE20_D11gp120.avi/293F	RV144_wk26	23.5 (4/17)	152	46.7 (7/15)	144	17.6 (3/17)	224	30.0 (3/10)	452
IgG3	CRF07_BC	gp120	CNE20_D11gp120.avi/293F	RV305_wk0	0.0 (0/20)		0.0 (0/18)		0.0 (0/19)		0.0 (0/13)	
IgG3	CRF07_BC	gp120	CNE20_D11gp120.avi/293F	RV305_wk2	5.0 (1/20)	386	16.7 (3/18)	228	0.0 (0/19)		0.0 (0/13)	
IgG3	CRF07_BC	gp120	CNE20_D11gp120.avi/293F	RV305_wk24	0.0 (0/20)		0.0 (0/18)		0.0 (0/19)		0.0 (0/13)	
IgG3	CRF07_BC	gp120	CNE20_D11gp120.avi/293F	RV305_wk26	0.0 (0/20)		0.0 (0/18)		0.0 (0/19)		0.0 (0/13)	
IgG3	CRF07_BC	gp120	CNE20_D11gp120.avi/293F	RV305_wk48	0.0 (0/20)		0.0 (0/18)		0.0 (0/19)		0.0 (0/13)	
IgG3	CRF07_BC	gp120	CNE20_D11gp120.avi/293F	RV305_wk72	0.0 (0/20)		0.0 (0/18)		0.0 (0/18)		0.0 (0/13)	

S8 Table continued

					Group 1: Combination		Group 2: AIDSVAX B/E		Group 3: ALVAC-HIV		RV305_Placebo Group	
Isotype	Clade	Env Region	Antigen	Study Week	Response Rate (Responders/ Total)	Median MFI Responders	Response Rate (Responders/ Total)	Median MFI Responders	Response Rate (Responders/ Total)	Median MFI Responders	Response Rate (Responders/ Total)	Median MFI Responders
IgG3	Consensus	gp120	Con 6 gp120/B	RV144_wk26	17.6 (3/17)	203	20.0 (3/15)	136	11.8 (2/17)	176	20.0 (2/10)	164
IgG3	Consensus	gp120	Con 6 gp120/B	RV305_wk0	0.0 (0/20)		0.0 (0/18)		0.0 (0/19)		0.0 (0/13)	
IgG3	Consensus	gp120	Con 6 gp120/B	RV305_wk2	10.0 (2/20)	509	16.7 (3/18)	573	0.0 (0/19)		0.0 (0/13)	
IgG3	Consensus	gp120	Con 6 gp120/B	RV305_wk24	0.0 (0/20)		0.0 (0/18)		0.0 (0/19)		0.0 (0/13)	
IgG3	Consensus	gp120	Con 6 gp120/B	RV305_wk26	5.0 (1/20)	112	0.0 (0/18)		0.0 (0/19)		0.0 (0/13)	
IgG3	Consensus	gp120	Con 6 gp120/B	RV305_wk48	0.0 (0/20)		0.0 (0/18)		0.0 (0/19)		0.0 (0/13)	
IgG3	Consensus	gp120	Con 6 gp120/B	RV305_wk72	0.0 (0/20)		0.0 (0/18)		0.0 (0/18)		0.0 (0/13)	
IgG3	A	gp140	9004S.gp140C.avi	RV144_wk26	5.9 (1/17)	127	0.0 (0/15)		0.0 (0/17)		0.0 (0/10)	
IgG3	A	gp140	9004S.gp140C.avi	RV305_wk0	0.0 (0/20)		0.0 (0/18)		0.0 (0/19)		0.0 (0/13)	
IgG3	A	gp140	9004S.gp140C.avi	RV305_wk2	5.0 (1/20)	282	11.1 (2/18)	146	0.0 (0/19)		0.0 (0/13)	
IgG3	A	gp140	9004S.gp140C.avi	RV305_wk24	0.0 (0/20)		0.0 (0/18)		0.0 (0/19)		0.0 (0/13)	
IgG3	A	gp140	9004S.gp140C.avi	RV305_wk26	0.0 (0/20)		0.0 (0/18)		0.0 (0/19)		0.0 (0/13)	
IgG3	A	gp140	9004S.gp140C.avi	RV305_wk48	0.0 (0/20)		0.0 (0/18)		0.0 (0/19)		0.0 (0/13)	
IgG3	A	gp140	9004S.gp140C.avi	RV305_wk72	0.0 (0/20)		0.0 (0/18)		0.0 (0/18)		0.0 (0/13)	
IgG3	B	gp140	RHPA4259_C7.gp140C.avi	RV144_wk26	29.4 (5/17)	121	46.7 (7/15)	165	17.6 (3/17)	149	40.0 (4/10)	258
IgG3	B	gp140	RHPA4259_C7.gp140C.avi	RV305_wk0	0.0 (0/20)		0.0 (0/18)		0.0 (0/19)		0.0 (0/13)	
IgG3	B	gp140	RHPA4259_C7.gp140C.avi	RV305_wk2	10.0 (2/20)	1058	27.8 (5/18)	174	0.0 (0/19)		0.0 (0/13)	
IgG3	B	gp140	RHPA4259_C7.gp140C.avi	RV305_wk24	0.0 (0/20)		0.0 (0/18)		0.0 (0/19)		0.0 (0/13)	
IgG3	B	gp140	RHPA4259_C7.gp140C.avi	RV305_wk26	10.0 (2/20)	169	5.6 (1/18)	114	0.0 (0/19)		0.0 (0/13)	
IgG3	B	gp140	RHPA4259_C7.gp140C.avi	RV305_wk48	0.0 (0/20)		0.0 (0/18)		0.0 (0/19)		0.0 (0/13)	
IgG3	B	gp140	RHPA4259_C7.gp140C.avi	RV305_wk72	0.0 (0/20)		0.0 (0/18)		0.0 (0/18)		0.0 (0/13)	
IgG3	B	gp140	SC42261_gp140.avi/293F	RV144_wk26	41.2 (7/17)	147	53.3 (8/15)	147	23.5 (4/17)	178	40.0 (4/10)	309
IgG3	B	gp140	SC42261_gp140.avi/293F	RV305_wk0	0.0 (0/20)		0.0 (0/18)		0.0 (0/19)		0.0 (0/13)	
IgG3	B	gp140	SC42261_gp140.avi/293F	RV305_wk2	15.0 (3/20)	258	27.8 (5/18)	208	0.0 (0/19)		0.0 (0/13)	
IgG3	B	gp140	SC42261_gp140.avi/293F	RV305_wk24	0.0 (0/20)		0.0 (0/18)		0.0 (0/19)		0.0 (0/13)	
IgG3	B	gp140	SC42261_gp140.avi/293F	RV305_wk26	5.0 (1/20)	282	5.6 (1/18)	144	0.0 (0/19)		0.0 (0/13)	
IgG3	B	gp140	SC42261_gp140.avi/293F	RV305_wk48	0.0 (0/20)		0.0 (0/18)		0.0 (0/19)		0.0 (0/13)	
IgG3	B	gp140	SC42261_gp140.avi/293F	RV305_wk72	0.0 (0/20)		0.0 (0/18)		0.0 (0/18)		0.0 (0/13)	
IgG3	B	gp140	WITO4160.gp140C.avi	RV144_wk26	5.9 (1/17)	117	6.7 (1/15)	133	5.9 (1/17)	151	0.0 (0/10)	
IgG3	B	gp140	WITO4160.gp140C.avi	RV305_wk0	0.0 (0/20)		0.0 (0/18)		0.0 (0/19)		0.0 (0/13)	
IgG3	B	gp140	WITO4160.gp140C.avi	RV305_wk2	10.0 (2/20)	138	16.7 (3/18)	113	0.0 (0/19)		0.0 (0/13)	
IgG3	B	gp140	WITO4160.gp140C.avi	RV305_wk24	0.0 (0/20)		0.0 (0/18)		0.0 (0/19)		0.0 (0/13)	
IgG3	B	gp140	WITO4160.gp140C.avi	RV305_wk26	0.0 (0/20)		0.0 (0/18)		0.0 (0/19)		0.0 (0/13)	
IgG3	B	gp140	WITO4160.gp140C.avi	RV305_wk48	0.0 (0/20)		0.0 (0/18)		0.0 (0/19)		0.0 (0/13)	
IgG3	B	gp140	WITO4160.gp140C.avi	RV305_wk72	0.0 (0/20)		0.0 (0/18)		0.0 (0/18)		0.0 (0/13)	

S8 Table continued

					Group 1: Combination		Group 2: AIDSVAX B/E		Group 3: ALVAC-HIV		RV305_Placebo Group	
Isotype	Clade	Env Region	Antigen	Study Week	Response Rate (Responders/ Total)	Median MFI Responders	Response Rate (Responders/ Total)	Median MFI Responders	Response Rate (Responders/ Total)	Median MFI Responders	Response Rate (Responders/ Total)	Median MFI Responders
IgG3	C	gp140	1086C gp140C_avi	RV144_wk26	82.4 (14/17)	286	80.0 (12/15)	716	94.1 (16/17)	277	90.0 (9/10)	310
IgG3	C	gp140	1086C gp140C_avi	RV305_wk0	0.0 (0/20)		0.0 (0/18)		0.0 (0/19)		0.0 (0/13)	
IgG3	C	gp140	1086C gp140C_avi	RV305_wk2	45.0 (9/20)	186	50.0 (9/18)	317	0.0 (0/19)		0.0 (0/13)	
IgG3	C	gp140	1086C gp140C_avi	RV305_wk24	0.0 (0/20)		0.0 (0/18)		0.0 (0/19)		0.0 (0/13)	
IgG3	C	gp140	1086C gp140C_avi	RV305_wk26	15.0 (3/20)	145	16.7 (3/18)	173	0.0 (0/19)		0.0 (0/13)	
IgG3	C	gp140	1086C gp140C_avi	RV305_wk48	0.0 (0/20)		0.0 (0/18)		0.0 (0/19)		0.0 (0/13)	
IgG3	C	gp140	1086C gp140C_avi	RV305_wk72	0.0 (0/20)		0.0 (0/18)		0.0 (0/18)		0.0 (0/13)	
IgG3	C	gp140	BF1266_gp140C.avi/293F	RV144_wk26	35.3 (6/17)	130	26.7 (4/15)	143	17.6 (3/17)	261	10.0 (1/10)	683
IgG3	C	gp140	BF1266_gp140C.avi/293F	RV305_wk0	0.0 (0/20)		0.0 (0/18)		0.0 (0/19)		0.0 (0/13)	
IgG3	C	gp140	BF1266_gp140C.avi/293F	RV305_wk2	15.0 (3/20)	267	11.1 (2/18)	597	0.0 (0/19)		0.0 (0/13)	
IgG3	C	gp140	BF1266_gp140C.avi/293F	RV305_wk24	0.0 (0/20)		0.0 (0/18)		0.0 (0/19)		0.0 (0/13)	
IgG3	C	gp140	BF1266_gp140C.avi/293F	RV305_wk26	0.0 (0/20)		0.0 (0/18)		0.0 (0/19)		0.0 (0/13)	
IgG3	C	gp140	BF1266_gp140C.avi/293F	RV305_wk48	0.0 (0/20)		0.0 (0/18)		0.0 (0/19)		0.0 (0/13)	
IgG3	C	gp140	BF1266_gp140C.avi/293F	RV305_wk72	0.0 (0/20)		0.0 (0/18)		0.0 (0/18)		0.0 (0/13)	
IgG3	C	gp140	C.CH505TF_gp140/293F	RV144_wk26	11.8 (2/17)	110	6.7 (1/15)	102	11.8 (2/17)	133	10.0 (1/10)	105
IgG3	C	gp140	C.CH505TF_gp140/293F	RV305_wk0	0.0 (0/20)		0.0 (0/18)		0.0 (0/19)		0.0 (0/13)	
IgG3	C	gp140	C.CH505TF_gp140/293F	RV305_wk2	0.0 (0/20)		5.6 (1/18)	178	0.0 (0/19)		0.0 (0/13)	
IgG3	C	gp140	C.CH505TF_gp140/293F	RV305_wk24	0.0 (0/20)		0.0 (0/18)		0.0 (0/19)		0.0 (0/13)	
IgG3	C	gp140	C.CH505TF_gp140/293F	RV305_wk26	0.0 (0/20)		0.0 (0/18)		0.0 (0/19)		0.0 (0/13)	
IgG3	C	gp140	C.CH505TF_gp140/293F	RV305_wk48	0.0 (0/20)		0.0 (0/18)		0.0 (0/19)		0.0 (0/13)	
IgG3	C	gp140	C.CH505TF_gp140/293F	RV305_wk72	0.0 (0/20)		0.0 (0/18)		0.0 (0/18)		0.0 (0/13)	
IgG3	Consensus	gp140	Con S gp140 CFI	RV144_wk26	35.3 (6/17)	215	66.7 (10/15)	179	35.3 (6/17)	165	50.0 (5/10)	209
IgG3	Consensus	gp140	Con S gp140 CFI	RV305_wk0	0.0 (0/20)		0.0 (0/18)		0.0 (0/19)		0.0 (0/13)	
IgG3	Consensus	gp140	Con S gp140 CFI	RV305_wk2	15.0 (3/20)	366	27.8 (5/18)	211	0.0 (0/19)		0.0 (0/13)	
IgG3	Consensus	gp140	Con S gp140 CFI	RV305_wk24	0.0 (0/20)		0.0 (0/18)		0.0 (0/19)		0.0 (0/13)	
IgG3	Consensus	gp140	Con S gp140 CFI	RV305_wk26	0.0 (0/20)		5.6 (1/18)	210	0.0 (0/19)		0.0 (0/13)	
IgG3	Consensus	gp140	Con S gp140 CFI	RV305_wk48	0.0 (0/20)		0.0 (0/18)		0.0 (0/19)		0.0 (0/13)	
IgG3	Consensus	gp140	Con S gp140 CFI	RV305_wk72	0.0 (0/20)		0.0 (0/18)		0.0 (0/18)		0.0 (0/13)	

S8 Table continued

					Group 1: Combination		Group 2: AIDSVAX B/E		Group 3: ALVAC-HIV		RV305_Placebo Group	
Isotype	Clade	Env Region	Antigen	Study Week	Response Rate (Responders/ Total)	Median MFI Responders	Response Rate (Responders/ Total)	Median MFI Responders	Response Rate (Responders/ Total)	Median MFI Responders	Response Rate (Responders/ Total)	Median MFI Responders
IgG3	Consensus CRF01_AE	gp140	AE.01.con_env03 gp140CF_avi	RV144_wk26	17.6 (3/17)	205	33.3 (5/15)	241	29.4 (5/17)	245	30.0 (3/10)	117
IgG3	Consensus CRF01_AE	gp140	AE.01.con_env03 gp140CF_avi	RV305_wk0	0.0 (0/20)		0.0 (0/18)		0.0 (0/19)		0.0 (0/13)	
IgG3	Consensus CRF01_AE	gp140	AE.01.con_env03 gp140CF_avi	RV305_wk2	10.0 (2/20)	311	16.7 (3/18)	272	0.0 (0/19)		0.0 (0/13)	
IgG3	Consensus CRF01_AE	gp140	AE.01.con_env03 gp140CF_avi	RV305_wk24	0.0 (0/20)		0.0 (0/18)		0.0 (0/19)		0.0 (0/13)	
IgG3	Consensus CRF01_AE	gp140	AE.01.con_env03 gp140CF_avi	RV305_wk26	0.0 (0/20)		0.0 (0/18)		0.0 (0/19)		0.0 (0/13)	
IgG3	Consensus CRF01_AE	gp140	AE.01.con_env03 gp140CF_avi	RV305_wk48	0.0 (0/20)		0.0 (0/18)		0.0 (0/19)		0.0 (0/13)	
IgG3	Consensus CRF01_AE	gp140	AE.01.con_env03 gp140CF_avi	RV305_wk72	0.0 (0/20)		0.0 (0/18)		0.0 (0/18)		0.0 (0/13)	
IgG3	A	V1V2	gp70-191084_B7 V1V2	RV144_wk26	41.2 (7/17)	466	46.7 (7/15)	566	35.3 (6/17)	1456	50.0 (5/10)	334
IgG3	A	V1V2	gp70-191084_B7 V1V2	RV305_wk0	0.0 (0/20)		0.0 (0/18)		0.0 (0/19)		0.0 (0/13)	
IgG3	A	V1V2	gp70-191084_B7 V1V2	RV305_wk2	20.0 (4/20)	334	16.7 (3/18)	147	0.0 (0/19)		0.0 (0/13)	
IgG3	A	V1V2	gp70-191084_B7 V1V2	RV305_wk24	0.0 (0/20)		5.6 (1/18)	261	0.0 (0/19)		0.0 (0/13)	
IgG3	A	V1V2	gp70-191084_B7 V1V2	RV305_wk26	15.0 (3/20)	531	5.6 (1/18)	257	0.0 (0/19)		0.0 (0/13)	
IgG3	A	V1V2	gp70-191084_B7 V1V2	RV305_wk48	5.0 (1/20)	113	0.0 (0/18)		0.0 (0/19)		0.0 (0/13)	
IgG3	A	V1V2	gp70-191084_B7 V1V2	RV305_wk72	5.0 (1/20)	128	0.0 (0/18)		0.0 (0/18)		0.0 (0/13)	
IgG3	B	V1V2	gp70-62357.14 V1V2	RV144_wk26	11.8 (2/17)	471	6.7 (1/15)	216	0.0 (0/17)		10.0 (1/10)	221
IgG3	B	V1V2	gp70-62357.14 V1V2	RV305_wk0	0.0 (0/20)		0.0 (0/18)		0.0 (0/19)		0.0 (0/13)	
IgG3	B	V1V2	gp70-62357.14 V1V2	RV305_wk2	5.0 (1/20)	645	0.0 (0/18)		0.0 (0/19)		0.0 (0/13)	
IgG3	B	V1V2	gp70-62357.14 V1V2	RV305_wk24	0.0 (0/20)		0.0 (0/18)		0.0 (0/19)		0.0 (0/13)	
IgG3	B	V1V2	gp70-62357.14 V1V2	RV305_wk26	5.0 (1/20)	644	0.0 (0/18)		0.0 (0/19)		0.0 (0/13)	
IgG3	B	V1V2	gp70-62357.14 V1V2	RV305_wk48	0.0 (0/20)		0.0 (0/18)		0.0 (0/19)		0.0 (0/13)	
IgG3	B	V1V2	gp70-62357.14 V1V2	RV305_wk72	0.0 (0/20)		0.0 (0/18)		0.0 (0/18)		0.0 (0/13)	
IgG3	B	V1V2	gp70-700010058 V1V2	RV144_wk26	5.9 (1/17)	111	0.0 (0/15)		5.9 (1/17)	1637	0.0 (0/10)	
IgG3	B	V1V2	gp70-700010058 V1V2	RV305_wk0	0.0 (0/20)		0.0 (0/18)		0.0 (0/19)		0.0 (0/13)	
IgG3	B	V1V2	gp70-700010058 V1V2	RV305_wk2	5.0 (1/20)	664	0.0 (0/18)		0.0 (0/19)		0.0 (0/13)	
IgG3	B	V1V2	gp70-700010058 V1V2	RV305_wk24	0.0 (0/20)		0.0 (0/18)		0.0 (0/19)		0.0 (0/13)	
IgG3	B	V1V2	gp70-700010058 V1V2	RV305_wk26	5.0 (1/20)	493	0.0 (0/18)		0.0 (0/19)		0.0 (0/13)	

S8 Table continued

					Group 1: Combination		Group 2: AIDSVAX B/E		Group 3: ALVAC-HIV		RV305_Placebo Group	
Isotype	Clade	Env Region	Antigen	Study Week	Response Rate (Responders/ Total)	Median MFI Responders	Response Rate (Responders/ Total)	Median MFI Responders	Response Rate (Responders/ Total)	Median MFI Responders	Response Rate (Responders/ Total)	Median MFI Responders
IgG3	C	gp140	1086C gp140C_avi	RV144_wk26	82.4 (14/17)	286	80.0 (12/15)	716	94.1 (16/17)	277	90.0 (9/10)	310
IgG3	C	gp140	1086C gp140C_avi	RV305_wk0	0.0 (0/20)		0.0 (0/18)		0.0 (0/19)		0.0 (0/13)	
IgG3	C	gp140	1086C gp140C_avi	RV305_wk2	45.0 (9/20)	186	50.0 (9/18)	317	0.0 (0/19)		0.0 (0/13)	
IgG3	C	gp140	1086C gp140C_avi	RV305_wk24	0.0 (0/20)		0.0 (0/18)		0.0 (0/19)		0.0 (0/13)	
IgG3	C	gp140	1086C gp140C_avi	RV305_wk26	15.0 (3/20)	145	16.7 (3/18)	173	0.0 (0/19)		0.0 (0/13)	
IgG3	C	gp140	1086C gp140C_avi	RV305_wk48	0.0 (0/20)		0.0 (0/18)		0.0 (0/19)		0.0 (0/13)	
IgG3	C	gp140	1086C gp140C_avi	RV305_wk72	0.0 (0/20)		0.0 (0/18)		0.0 (0/18)		0.0 (0/13)	
IgG3	C	gp140	BF1266_gp140C.avi/293F	RV144_wk26	35.3 (6/17)	130	26.7 (4/15)	143	17.6 (3/17)	261	10.0 (1/10)	683
IgG3	C	gp140	BF1266_gp140C.avi/293F	RV305_wk0	0.0 (0/20)		0.0 (0/18)		0.0 (0/19)		0.0 (0/13)	
IgG3	C	gp140	BF1266_gp140C.avi/293F	RV305_wk2	15.0 (3/20)	267	11.1 (2/18)	597	0.0 (0/19)		0.0 (0/13)	
IgG3	C	gp140	BF1266_gp140C.avi/293F	RV305_wk24	0.0 (0/20)		0.0 (0/18)		0.0 (0/19)		0.0 (0/13)	
IgG3	C	gp140	BF1266_gp140C.avi/293F	RV305_wk26	0.0 (0/20)		0.0 (0/18)		0.0 (0/19)		0.0 (0/13)	
IgG3	C	gp140	BF1266_gp140C.avi/293F	RV305_wk48	0.0 (0/20)		0.0 (0/18)		0.0 (0/19)		0.0 (0/13)	
IgG3	C	gp140	BF1266_gp140C.avi/293F	RV305_wk72	0.0 (0/20)		0.0 (0/18)		0.0 (0/18)		0.0 (0/13)	
IgG3	C	gp140	C.CH505TF_gp140/293F	RV144_wk26	11.8 (2/17)	110	6.7 (1/15)	102	11.8 (2/17)	133	10.0 (1/10)	105
IgG3	C	gp140	C.CH505TF_gp140/293F	RV305_wk0	0.0 (0/20)		0.0 (0/18)		0.0 (0/19)		0.0 (0/13)	
IgG3	C	gp140	C.CH505TF_gp140/293F	RV305_wk2	0.0 (0/20)		5.6 (1/18)	178	0.0 (0/19)		0.0 (0/13)	
IgG3	C	gp140	C.CH505TF_gp140/293F	RV305_wk24	0.0 (0/20)		0.0 (0/18)		0.0 (0/19)		0.0 (0/13)	
IgG3	C	gp140	C.CH505TF_gp140/293F	RV305_wk26	0.0 (0/20)		0.0 (0/18)		0.0 (0/19)		0.0 (0/13)	
IgG3	C	gp140	C.CH505TF_gp140/293F	RV305_wk48	0.0 (0/20)		0.0 (0/18)		0.0 (0/19)		0.0 (0/13)	
IgG3	C	gp140	C.CH505TF_gp140/293F	RV305_wk72	0.0 (0/20)		0.0 (0/18)		0.0 (0/18)		0.0 (0/13)	
IgG3	Consensus	gp140	Con S gp140 CFI	RV144_wk26	35.3 (6/17)	215	66.7 (10/15)	179	35.3 (6/17)	165	50.0 (5/10)	209
IgG3	Consensus	gp140	Con S gp140 CFI	RV305_wk0	0.0 (0/20)		0.0 (0/18)		0.0 (0/19)		0.0 (0/13)	
IgG3	Consensus	gp140	Con S gp140 CFI	RV305_wk2	15.0 (3/20)	366	27.8 (5/18)	211	0.0 (0/19)		0.0 (0/13)	
IgG3	Consensus	gp140	Con S gp140 CFI	RV305_wk24	0.0 (0/20)		0.0 (0/18)		0.0 (0/19)		0.0 (0/13)	
IgG3	Consensus	gp140	Con S gp140 CFI	RV305_wk26	0.0 (0/20)		5.6 (1/18)	210	0.0 (0/19)		0.0 (0/13)	
IgG3	Consensus	gp140	Con S gp140 CFI	RV305_wk48	0.0 (0/20)		0.0 (0/18)		0.0 (0/19)		0.0 (0/13)	
IgG3	Consensus	gp140	Con S gp140 CFI	RV305_wk72	0.0 (0/20)		0.0 (0/18)		0.0 (0/18)		0.0 (0/13)	

S8 Table continued

					Group 1: Combination		Group 2: AIDSVAX B/E		Group 3: ALVAC-HIV		RV305_Placebo Group	
Isotype	Clade	Env Region	Antigen	Study Week	Response Rate (Responders/ Total)	Median MFI Responders	Response Rate (Responders/ Total)	Median MFI Responders	Response Rate (Responders/ Total)	Median MFI Responders	Response Rate (Responders/ Total)	Median MFI Responders
IgG3	B	V1V2	gp70-RHPA4259.7 V1V2	RV144_wk26	23.5 (4/17)	326	20.0 (3/15)	147	17.6 (3/17)	280	10.0 (1/10)	185
IgG3	B	V1V2	gp70-RHPA4259.7 V1V2	RV305_wk0	5.0 (1/20)	140	0.0 (0/18)		0.0 (0/19)		0.0 (0/13)	
IgG3	B	V1V2	gp70-RHPA4259.7 V1V2	RV305_wk2	20.0 (4/20)	285	5.6 (1/18)	111	0.0 (0/19)		0.0 (0/13)	
IgG3	B	V1V2	gp70-RHPA4259.7 V1V2	RV305_wk24	5.0 (1/20)	175	0.0 (0/18)		0.0 (0/19)		0.0 (0/13)	
IgG3	B	V1V2	gp70-RHPA4259.7 V1V2	RV305_wk26	15.0 (3/20)	273	5.6 (1/18)	181	0.0 (0/19)		0.0 (0/13)	
IgG3	B	V1V2	gp70-RHPA4259.7 V1V2	RV305_wk48	5.0 (1/20)	192	0.0 (0/18)		0.0 (0/19)		0.0 (0/13)	
IgG3	B	V1V2	gp70-RHPA4259.7 V1V2	RV305_wk72	5.0 (1/20)	153	0.0 (0/18)		0.0 (0/18)		0.0 (0/13)	
IgG3	B	V1V2	gp70-TT31P.2F10.2792 V1V2	RV144_wk26	11.8 (2/17)	568	6.7 (1/15)	207	11.8 (2/17)	994	10.0 (1/10)	216
IgG3	B	V1V2	gp70-TT31P.2F10.2792 V1V2	RV305_wk0	0.0 (0/20)		0.0 (0/18)		0.0 (0/19)		0.0 (0/13)	
IgG3	B	V1V2	gp70-TT31P.2F10.2792 V1V2	RV305_wk2	5.0 (1/20)	838	0.0 (0/18)		0.0 (0/19)		0.0 (0/13)	
IgG3	B	V1V2	gp70-TT31P.2F10.2792 V1V2	RV305_wk24	0.0 (0/20)		0.0 (0/18)		0.0 (0/19)		0.0 (0/13)	
IgG3	B	V1V2	gp70-TT31P.2F10.2792 V1V2	RV305_wk26	5.0 (1/20)	820	0.0 (0/18)		0.0 (0/19)		0.0 (0/13)	
IgG3	B	V1V2	gp70-TT31P.2F10.2792 V1V2	RV305_wk48	0.0 (0/20)		0.0 (0/18)		0.0 (0/19)		0.0 (0/13)	
IgG3	B	V1V2	gp70-TT31P.2F10.2792 V1V2	RV305_wk72	0.0 (0/20)		0.0 (0/18)		0.0 (0/18)		0.0 (0/13)	
IgG3	B	V1V2	gp70_B.CaseA2 V1/V2/169K	RV144_wk26	29.4 (5/17)	296	13.3 (2/15)	239	11.8 (2/17)	1931	20.0 (2/10)	251
IgG3	B	V1V2	gp70_B.CaseA2 V1/V2/169K	RV305_wk0	5.0 (1/20)	2024	0.0 (0/18)		0.0 (0/19)		0.0 (0/13)	
IgG3	B	V1V2	gp70_B.CaseA2 V1/V2/169K	RV305_wk2	15.0 (3/20)	490	5.6 (1/18)	132	0.0 (0/19)		0.0 (0/13)	
IgG3	B	V1V2	gp70_B.CaseA2 V1/V2/169K	RV305_wk24	5.0 (1/20)	911	0.0 (0/18)		0.0 (0/19)		0.0 (0/13)	
IgG3	B	V1V2	gp70_B.CaseA2 V1/V2/169K	RV305_wk26	10.0 (2/20)	622	0.0 (0/18)		0.0 (0/19)		0.0 (0/13)	
IgG3	B	V1V2	gp70_B.CaseA2 V1/V2/169K	RV305_wk48	5.0 (1/20)	1934	0.0 (0/18)		0.0 (0/19)		0.0 (0/13)	
IgG3	B	V1V2	gp70_B.CaseA2 V1/V2/169K	RV305_wk72	5.0 (1/20)	492	0.0 (0/18)		0.0 (0/18)		0.0 (0/13)	
IgG3	B	V1V2	gp70_B.CaseA_V1_V2	RV144_wk26	11.8 (2/17)	576	6.7 (1/15)	277	11.8 (2/17)	1582	10.0 (1/10)	301
IgG3	B	V1V2	gp70_B.CaseA_V1_V2	RV305_wk0	5.0 (1/20)	3282	0.0 (0/18)		0.0 (0/19)		0.0 (0/13)	
IgG3	B	V1V2	gp70_B.CaseA_V1_V2	RV305_wk2	15.0 (3/20)	628	5.6 (1/18)	125	0.0 (0/19)		0.0 (0/13)	
IgG3	B	V1V2	gp70_B.CaseA_V1_V2	RV305_wk24	5.0 (1/20)	1451	0.0 (0/18)		0.0 (0/19)		0.0 (0/13)	
IgG3	B	V1V2	gp70_B.CaseA_V1_V2	RV305_wk26	15.0 (3/20)	629	0.0 (0/18)		0.0 (0/19)		0.0 (0/13)	
IgG3	B	V1V2	gp70_B.CaseA_V1_V2	RV305_wk48	5.0 (1/20)	1182	0.0 (0/18)		0.0 (0/19)		0.0 (0/13)	
IgG3	B	V1V2	gp70_B.CaseA_V1_V2	RV305_wk72	5.0 (1/20)	851	0.0 (0/18)		0.0 (0/18)		0.0 (0/13)	
IgG3	C	V1V2	C.1086C_V1_V2 Tags	RV144_wk26	47.1 (8/17)	332	73.3 (11/15)	404	41.2 (7/17)	491	60.0 (6/10)	224
IgG3	C	V1V2	C.1086C_V1_V2 Tags	RV305_wk0	0.0 (0/20)		0.0 (0/18)		0.0 (0/19)		0.0 (0/13)	
IgG3	C	V1V2	C.1086C_V1_V2 Tags	RV305_wk2	20.0 (4/20)	559	33.3 (6/18)	185	0.0 (0/19)		7.7 (1/13)	116
IgG3	C	V1V2	C.1086C_V1_V2 Tags	RV305_wk24	0.0 (0/20)		0.0 (0/18)		5.3 (1/19)	161	0.0 (0/13)	
IgG3	C	V1V2	C.1086C_V1_V2 Tags	RV305_wk26	10.0 (2/20)	968	5.6 (1/18)	125	5.3 (1/19)	149	0.0 (0/13)	
IgG3	C	V1V2	C.1086C_V1_V2 Tags	RV305_wk48	5.0 (1/20)	116	0.0 (0/18)		0.0 (0/19)		0.0 (0/13)	
IgG3	C	V1V2	C.1086C_V1_V2 Tags	RV305_wk72	0.0 (0/20)		0.0 (0/18)		0.0 (0/18)		0.0 (0/13)	

S8 Table continued

					Group 1: Combination		Group 2: AIDSVAX B/E		Group 3: ALVAC-HIV		RV305_Placebo Group	
Isotype	Clade	Env Region	Antigen	Study Week	Response Rate (Responders/ Total)	Median MFI Responders	Response Rate (Responders/ Total)	Median MFI Responders	Response Rate (Responders/ Total)	Median MFI Responders	Response Rate (Responders/ Total)	Median MFI Responders
IgG3	C	V1V2	gp70-001428.2.42 V1V2	RV144_wk26	11.8 (2/17)	429	13.3 (2/15)	209	23.5 (4/17)	161	20.0 (2/10)	188
IgG3	C	V1V2	gp70-001428.2.42 V1V2	RV305_wk0	0.0 (0/20)		0.0 (0/18)		0.0 (0/19)		0.0 (0/13)	
IgG3	C	V1V2	gp70-001428.2.42 V1V2	RV305_wk2	10.0 (2/20)	415	5.6 (1/18)	109	0.0 (0/19)		0.0 (0/13)	
IgG3	C	V1V2	gp70-001428.2.42 V1V2	RV305_wk24	0.0 (0/20)		0.0 (0/18)		0.0 (0/19)		0.0 (0/13)	
IgG3	C	V1V2	gp70-001428.2.42 V1V2	RV305_wk26	10.0 (2/20)	428	5.6 (1/18)	127	0.0 (0/19)		0.0 (0/13)	
IgG3	C	V1V2	gp70-001428.2.42 V1V2	RV305_wk48	0.0 (0/20)		0.0 (0/18)		0.0 (0/19)		0.0 (0/13)	
IgG3	C	V1V2	gp70-001428.2.42 V1V2	RV305_wk72	0.0 (0/20)		0.0 (0/18)		0.0 (0/18)		0.0 (0/13)	
IgG3	C	V1V2	gp70-7060101641 V1V2	RV144_wk26	17.6 (3/17)	500	20.0 (3/15)	283	17.6 (3/17)	691	20.0 (2/10)	697
IgG3	C	V1V2	gp70-7060101641 V1V2	RV305_wk0	0.0 (0/20)		0.0 (0/18)		0.0 (0/19)		0.0 (0/13)	
IgG3	C	V1V2	gp70-7060101641 V1V2	RV305_wk2	5.0 (1/20)	387	5.6 (1/18)	574	0.0 (0/19)		0.0 (0/13)	
IgG3	C	V1V2	gp70-7060101641 V1V2	RV305_wk24	0.0 (0/20)		0.0 (0/18)		0.0 (0/19)		0.0 (0/13)	
IgG3	C	V1V2	gp70-7060101641 V1V2	RV305_wk26	5.0 (1/20)	386	0.0 (0/18)		0.0 (0/19)		0.0 (0/13)	
IgG3	C	V1V2	gp70-7060101641 V1V2	RV305_wk48	0.0 (0/20)		0.0 (0/18)		0.0 (0/19)		0.0 (0/13)	
IgG3	C	V1V2	gp70-7060101641 V1V2	RV305_wk72	0.0 (0/20)		0.0 (0/18)		0.0 (0/18)		0.0 (0/13)	
IgG3	C	V1V2	gp70-96ZM651.02 V1v2	RV144_wk26	0.0 (0/17)		0.0 (0/15)		0.0 (0/17)		0.0 (0/10)	
IgG3	C	V1V2	gp70-96ZM651.02 V1v2	RV305_wk0	0.0 (0/20)		0.0 (0/18)		0.0 (0/19)		0.0 (0/13)	
IgG3	C	V1V2	gp70-96ZM651.02 V1v2	RV305_wk2	0.0 (0/20)		0.0 (0/18)		0.0 (0/19)		0.0 (0/13)	
IgG3	C	V1V2	gp70-96ZM651.02 V1v2	RV305_wk24	0.0 (0/20)		0.0 (0/18)		0.0 (0/19)		0.0 (0/13)	
IgG3	C	V1V2	gp70-96ZM651.02 V1v2	RV305_wk26	0.0 (0/20)		0.0 (0/18)		0.0 (0/19)		0.0 (0/13)	
IgG3	C	V1V2	gp70-96ZM651.02 V1v2	RV305_wk48	0.0 (0/20)		0.0 (0/18)		0.0 (0/19)		0.0 (0/13)	
IgG3	C	V1V2	gp70-96ZM651.02 V1v2	RV305_wk72	0.0 (0/20)		0.0 (0/18)		0.0 (0/18)		0.0 (0/13)	
IgG3	C	V1V2	gp70-BF1266_431a_V1V2	RV144_wk26	11.8 (2/17)	1067	6.7 (1/15)	264	11.8 (2/17)	3881	10.0 (1/10)	182
IgG3	C	V1V2	gp70-BF1266_431a_V1V2	RV305_wk0	0.0 (0/20)		0.0 (0/18)		0.0 (0/19)		0.0 (0/13)	
IgG3	C	V1V2	gp70-BF1266_431a_V1V2	RV305_wk2	5.0 (1/20)	642	0.0 (0/18)		0.0 (0/19)		0.0 (0/13)	
IgG3	C	V1V2	gp70-BF1266_431a_V1V2	RV305_wk24	0.0 (0/20)		0.0 (0/18)		0.0 (0/19)		0.0 (0/13)	
IgG3	C	V1V2	gp70-BF1266_431a_V1V2	RV305_wk26	5.0 (1/20)	576	0.0 (0/18)		0.0 (0/19)		0.0 (0/13)	
IgG3	C	V1V2	gp70-BF1266_431a_V1V2	RV305_wk48	0.0 (0/20)		0.0 (0/18)		0.0 (0/19)		0.0 (0/13)	
IgG3	C	V1V2	gp70-BF1266_431a_V1V2	RV305_wk72	0.0 (0/20)		0.0 (0/18)		0.0 (0/18)		0.0 (0/13)	
IgG3	C	V1V2	gp70-CAP210.2.00.E8 V1V2	RV144_wk26	5.9 (1/17)	268	0.0 (0/15)		5.9 (1/17)	1716	0.0 (0/10)	
IgG3	C	V1V2	gp70-CAP210.2.00.E8 V1V2	RV305_wk0	0.0 (0/20)		0.0 (0/18)		0.0 (0/19)		0.0 (0/13)	
IgG3	C	V1V2	gp70-CAP210.2.00.E8 V1V2	RV305_wk2	5.0 (1/20)	260	0.0 (0/18)		0.0 (0/19)		0.0 (0/13)	
IgG3	C	V1V2	gp70-CAP210.2.00.E8 V1V2	RV305_wk24	0.0 (0/20)		0.0 (0/18)		0.0 (0/19)		0.0 (0/13)	
IgG3	C	V1V2	gp70-CAP210.2.00.E8 V1V2	RV305_wk26	5.0 (1/20)	234	0.0 (0/18)		0.0 (0/19)		0.0 (0/13)	
IgG3	C	V1V2	gp70-CAP210.2.00.E8 V1V2	RV305_wk48	0.0 (0/20)		0.0 (0/18)		0.0 (0/19)		0.0 (0/13)	
IgG3	C	V1V2	gp70-CAP210.2.00.E8 V1V2	RV305_wk72	0.0 (0/20)		0.0 (0/18)		0.0 (0/18)		0.0 (0/13)	

S8 Table continued

					Group 1: Combination		Group 2: AIDSVAX B/E		Group 3: ALVAC-HIV		RV305_Placebo Group	
Isotype	Clade	Env Region	Antigen	Study Week	Response Rate (Responders/ Total)	Median MFI Responders	Response Rate (Responders/ Total)	Median MFI Responders	Response Rate (Responders/ Total)	Median MFI Responders	Response Rate (Responders/ Total)	Median MFI Responders
IgG3	C	V1V2	gp70-Ce1086_B2 V1V2	RV144_wk26	58.8 (10/17)	540	86.7 (13/15)	480	64.7 (11/17)	1624	60.0 (6/10)	1071
IgG3	C	V1V2	gp70-Ce1086_B2 V1V2	RV305_wk0	0.0 (0/20)		0.0 (0/18)		0.0 (0/19)		0.0 (0/13)	
IgG3	C	V1V2	gp70-Ce1086_B2 V1V2	RV305_wk2	20.0 (4/20)	308	33.3 (6/18)	317	10.5 (2/19)	185	0.0 (0/13)	
IgG3	C	V1V2	gp70-Ce1086_B2 V1V2	RV305_wk24	0.0 (0/20)		5.6 (1/18)	400	0.0 (0/19)		0.0 (0/13)	
IgG3	C	V1V2	gp70-Ce1086_B2 V1V2	RV305_wk26	15.0 (3/20)	476	16.7 (3/18)	117	5.3 (1/19)	281	0.0 (0/13)	
IgG3	C	V1V2	gp70-Ce1086_B2 V1V2	RV305_wk48	5.0 (1/20)	111	0.0 (0/18)		5.3 (1/19)	103	0.0 (0/13)	
IgG3	C	V1V2	gp70-Ce1086_B2 V1V2	RV305_wk72	5.0 (1/20)	111	0.0 (0/18)		0.0 (0/18)		0.0 (0/13)	
IgG3	C	V1V2	gp70-TV1.21 V1V2	RV144_wk26	5.9 (1/17)	1277	6.7 (1/15)	366	5.9 (1/17)	3248	10.0 (1/10)	361
IgG3	C	V1V2	gp70-TV1.21 V1V2	RV305_wk0	0.0 (0/20)		0.0 (0/18)		0.0 (0/19)		0.0 (0/13)	
IgG3	C	V1V2	gp70-TV1.21 V1V2	RV305_wk2	5.0 (1/20)	1114	0.0 (0/18)		0.0 (0/19)		0.0 (0/13)	
IgG3	C	V1V2	gp70-TV1.21 V1V2	RV305_wk24	0.0 (0/20)		0.0 (0/18)		0.0 (0/19)		0.0 (0/13)	
IgG3	C	V1V2	gp70-TV1.21 V1V2	RV305_wk26	5.0 (1/20)	1045	0.0 (0/18)		0.0 (0/19)		0.0 (0/13)	
IgG3	C	V1V2	gp70-TV1.21 V1V2	RV305_wk48	0.0 (0/20)		0.0 (0/18)		0.0 (0/19)		0.0 (0/13)	
IgG3	C	V1V2	gp70-TV1.21 V1V2	RV305_wk72	0.0 (0/20)		0.0 (0/18)		0.0 (0/18)		0.0 (0/13)	
IgG3	CRF01_AE	V1V2	AE.A244 V1V2 tags	RV144_wk26	82.4 (14/17)	674	86.7 (13/15)	778	76.5 (13/17)	992	80.0 (8/10)	882
IgG3	CRF01_AE	V1V2	AE.A244 V1V2 tags	RV305_wk0	0.0 (0/20)		0.0 (0/18)		5.3 (1/19)	124	0.0 (0/13)	
IgG3	CRF01_AE	V1V2	AE.A244 V1V2 tags	RV305_wk2	45.0 (9/20)	244	66.7 (12/18)	375	5.3 (1/19)	130	0.0 (0/13)	
IgG3	CRF01_AE	V1V2	AE.A244 V1V2 tags	RV305_wk24	5.0 (1/20)	121	5.6 (1/18)	152	5.3 (1/19)	121	0.0 (0/13)	
IgG3	CRF01_AE	V1V2	AE.A244 V1V2 tags	RV305_wk26	35.0 (7/20)	177	27.8 (5/18)	263	5.3 (1/19)	109	0.0 (0/13)	
IgG3	CRF01_AE	V1V2	AE.A244 V1V2 tags	RV305_wk48	5.0 (1/20)	388	5.6 (1/18)	216	5.3 (1/19)	104	0.0 (0/13)	
IgG3	CRF01_AE	V1V2	AE.A244 V1V2 tags	RV305_wk72	5.0 (1/20)	456	5.6 (1/18)	132	0.0 (0/18)		0.0 (0/13)	
IgG3	CRF01_AE	V1V2	gp70-C2101.c01_V1V2	RV144_wk26	35.3 (6/17)	1211	40.0 (6/15)	451	17.6 (3/17)	2348	20.0 (2/10)	2303
IgG3	CRF01_AE	V1V2	gp70-C2101.c01_V1V2	RV305_wk0	0.0 (0/20)		0.0 (0/18)		0.0 (0/19)		0.0 (0/13)	
IgG3	CRF01_AE	V1V2	gp70-C2101.c01_V1V2	RV305_wk2	10.0 (2/20)	136	16.7 (3/18)	233	0.0 (0/19)		0.0 (0/13)	
IgG3	CRF01_AE	V1V2	gp70-C2101.c01_V1V2	RV305_wk24	0.0 (0/20)		5.6 (1/18)	187	0.0 (0/19)		0.0 (0/13)	
IgG3	CRF01_AE	V1V2	gp70-C2101.c01_V1V2	RV305_wk26	0.0 (0/20)		5.6 (1/18)	215	0.0 (0/19)		0.0 (0/13)	
IgG3	CRF01_AE	V1V2	gp70-C2101.c01_V1V2	RV305_wk48	0.0 (0/20)		0.0 (0/18)		0.0 (0/19)		0.0 (0/13)	
IgG3	CRF01_AE	V1V2	gp70-C2101.c01_V1V2	RV305_wk72	0.0 (0/20)		0.0 (0/18)		0.0 (0/18)		0.0 (0/13)	
IgG3	CRF01_AE	V1V2	gp70-CM244.ec1 V1V2	RV144_wk26	70.6 (12/17)	703	86.7 (13/15)	863	64.7 (11/17)	2394	80.0 (8/10)	1218
IgG3	CRF01_AE	V1V2	gp70-CM244.ec1 V1V2	RV305_wk0	0.0 (0/20)		0.0 (0/18)		0.0 (0/19)		0.0 (0/13)	
IgG3	CRF01_AE	V1V2	gp70-CM244.ec1 V1V2	RV305_wk2	35.0 (7/20)	157	38.9 (7/18)	310	10.5 (2/19)	250	7.7 (1/13)	107
IgG3	CRF01_AE	V1V2	gp70-CM244.ec1 V1V2	RV305_wk24	0.0 (0/20)		5.6 (1/18)	544	0.0 (0/19)		0.0 (0/13)	
IgG3	CRF01_AE	V1V2	gp70-CM244.ec1 V1V2	RV305_wk26	20.0 (4/20)	406	22.2 (4/18)	163	5.3 (1/19)	372	7.7 (1/13)	105
IgG3	CRF01_AE	V1V2	gp70-CM244.ec1 V1V2	RV305_wk48	5.0 (1/20)	142	0.0 (0/18)		5.3 (1/19)	129	0.0 (0/13)	
IgG3	CRF01_AE	V1V2	gp70-CM244.ec1 V1V2	RV305_wk72	5.0 (1/20)	134	0.0 (0/18)		0.0 (0/18)		7.7 (1/13)	105

S8 Table continued

					Group 1: Combination		Group 2: AIDSVAX B/E		Group 3: ALVAC-HIV		RV305_Placebo Group	
Isotype	Clade	Env Region	Antigen	Study Week	Response Rate (Responders/ Total)	Median MFI Responders	Response Rate (Responders/ Total)	Median MFI Responders	Response Rate (Responders/ Total)	Median MFI Responders	Response Rate (Responders/ Total)	Median MFI Responders
IgG3	CRF07_BC	V1V2	gp70-BJOX002000.03.2 V1V2	RV144_wk26	0.0 (0/17)		0.0 (0/15)		5.9 (1/17)	9592	0.0 (0/10)	
IgG3	CRF07_BC	V1V2	gp70-BJOX002000.03.2 V1V2	RV305_wk0	5.0 (1/20)	7411	0.0 (0/18)		0.0 (0/19)		0.0 (0/13)	
IgG3	CRF07_BC	V1V2	gp70-BJOX002000.03.2 V1V2	RV305_wk2	0.0 (0/20)		0.0 (0/18)		0.0 (0/19)		0.0 (0/13)	
IgG3	CRF07_BC	V1V2	gp70-BJOX002000.03.2 V1V2	RV305_wk24	0.0 (0/20)		0.0 (0/18)		0.0 (0/19)		0.0 (0/13)	
IgG3	CRF07_BC	V1V2	gp70-BJOX002000.03.2 V1V2	RV305_wk26	0.0 (0/20)		0.0 (0/18)		0.0 (0/19)		0.0 (0/13)	
IgG3	CRF07_BC	V1V2	gp70-BJOX002000.03.2 V1V2	RV305_wk48	0.0 (0/20)		0.0 (0/18)		0.0 (0/19)		0.0 (0/13)	
IgG3	CRF07_BC	V1V2	gp70-BJOX002000.03.2 V1V2	RV305_wk72	0.0 (0/20)		0.0 (0/18)		0.0 (0/18)		0.0 (0/13)	
IgG3	CRF01_AE	V2	AE.A244 V2 tags/293F	RV144_wk26	35.3 (6/17)	189	53.3 (8/15)	340	52.9 (9/17)	327	50.0 (5/10)	265
IgG3	CRF01_AE	V2	AE.A244 V2 tags/293F	RV305_wk0	0.0 (0/20)		0.0 (0/18)		0.0 (0/19)		0.0 (0/13)	
IgG3	CRF01_AE	V2	AE.A244 V2 tags/293F	RV305_wk2	5.0 (1/20)	865	16.7 (3/18)	324	0.0 (0/19)		0.0 (0/13)	
IgG3	CRF01_AE	V2	AE.A244 V2 tags/293F	RV305_wk24	0.0 (0/20)		0.0 (0/18)		0.0 (0/19)		0.0 (0/13)	
IgG3	CRF01_AE	V2	AE.A244 V2 tags/293F	RV305_wk26	5.0 (1/20)	185	0.0 (0/18)		0.0 (0/19)		0.0 (0/13)	
IgG3	CRF01_AE	V2	AE.A244 V2 tags/293F	RV305_wk48	0.0 (0/20)		0.0 (0/18)		0.0 (0/19)		0.0 (0/13)	
IgG3	CRF01_AE	V2	AE.A244 V2 tags/293F	RV305_wk72	0.0 (0/20)		0.0 (0/18)		0.0 (0/18)		0.0 (0/13)	
IgG3	B	V3	B.MN V3 gp70	RV144_wk26	47.1 (8/17)	158	60.0 (9/15)	153	23.5 (4/17)	182	30.0 (3/10)	158
IgG3	B	V3	B.MN V3 gp70	RV305_wk0	0.0 (0/20)		0.0 (0/18)		0.0 (0/19)		0.0 (0/13)	
IgG3	B	V3	B.MN V3 gp70	RV305_wk2	30.0 (6/20)	179	33.3 (6/18)	499	0.0 (0/19)		0.0 (0/13)	
IgG3	B	V3	B.MN V3 gp70	RV305_wk24	0.0 (0/20)		5.6 (1/18)	108	0.0 (0/19)		0.0 (0/13)	
IgG3	B	V3	B.MN V3 gp70	RV305_wk26	10.0 (2/20)	211	16.7 (3/18)	168	0.0 (0/19)		0.0 (0/13)	
IgG3	B	V3	B.MN V3 gp70	RV305_wk48	0.0 (0/20)		0.0 (0/18)		0.0 (0/19)		0.0 (0/13)	
IgG3	B	V3	B.MN V3 gp70	RV305_wk72	0.0 (0/20)		0.0 (0/18)		0.0 (0/18)		0.0 (0/13)	
IgG3	N/A	CD4i	HxB2 new 8b core 6x His	RV144_wk26	0.0 (0/17)		6.7 (1/15)	102	0.0 (0/17)		20.0 (2/10)	145
IgG3	N/A	CD4i	HxB2 new 8b core 6x His	RV305_wk0	0.0 (0/20)		0.0 (0/18)		0.0 (0/19)		0.0 (0/13)	
IgG3	N/A	CD4i	HxB2 new 8b core 6x His	RV305_wk2	0.0 (0/20)		5.6 (1/18)	495	0.0 (0/19)		0.0 (0/13)	
IgG3	N/A	CD4i	HxB2 new 8b core 6x His	RV305_wk24	0.0 (0/20)		0.0 (0/18)		0.0 (0/19)		0.0 (0/13)	
IgG3	N/A	CD4i	HxB2 new 8b core 6x His	RV305_wk26	0.0 (0/20)		0.0 (0/18)		0.0 (0/19)		0.0 (0/13)	
IgG3	N/A	CD4i	HxB2 new 8b core 6x His	RV305_wk48	0.0 (0/20)		0.0 (0/18)		0.0 (0/19)		0.0 (0/13)	
IgG3	N/A	CD4i	HxB2 new 8b core 6x His	RV305_wk72	0.0 (0/20)		0.0 (0/18)		0.0 (0/18)		0.0 (0/13)	
IgG3	N/A	CD4i	YU2 gp120 WT	RV144_wk26	5.9 (1/17)	8502	0.0 (0/15)		0.0 (0/17)		0.0 (0/10)	
IgG3	N/A	CD4i	YU2 gp120 WT	RV305_wk0	0.0 (0/20)		0.0 (0/18)		0.0 (0/19)		0.0 (0/13)	
IgG3	N/A	CD4i	YU2 gp120 WT	RV305_wk2	0.0 (0/20)		0.0 (0/18)		0.0 (0/19)		0.0 (0/13)	
IgG3	N/A	CD4i	YU2 gp120 WT	RV305_wk24	0.0 (0/20)		0.0 (0/18)		0.0 (0/19)		0.0 (0/13)	
IgG3	N/A	CD4i	YU2 gp120 WT	RV305_wk26	0.0 (0/20)		0.0 (0/18)		0.0 (0/19)		0.0 (0/13)	
IgG3	N/A	CD4i	YU2 gp120 WT	RV305_wk48	0.0 (0/20)		0.0 (0/18)		0.0 (0/19)		0.0 (0/13)	
IgG3	N/A	CD4i	YU2 gp120 WT	RV305_wk72	0.0 (0/20)		0.0 (0/18)		0.0 (0/18)		0.0 (0/13)	

S8 Table continued

					Group 1: Combination		Group 2: AIDSVAX B/E		Group 3: ALVAC-HIV		RV305_Placebo Group	
**Isotype**	**Clade**	**Env Region**	**Antigen**	**Study Week**	**Response Rate (Responders/ Total)**	**Median MFI Responders**	**Response Rate (Responders/ Total)**	**Median MFI Responders**	**Response Rate (Responders/ Total)**	**Median MFI Responders**	**Response Rate (Responders/ Total)**	**Median MFI Responders**
IgG3	N/A	CD4bs	RSC3	RV144_wk26	0.0 (0/17)		0.0 (0/15)		0.0 (0/17)		0.0 (0/10)	
IgG3	N/A	CD4bs	RSC3	RV305_wk0	0.0 (0/20)		0.0 (0/18)		0.0 (0/19)		0.0 (0/13)	
IgG3	N/A	CD4bs	RSC3	RV305_wk2	0.0 (0/20)		0.0 (0/18)		0.0 (0/19)		0.0 (0/13)	
IgG3	N/A	CD4bs	RSC3	RV305_wk24	0.0 (0/20)		0.0 (0/18)		0.0 (0/19)		0.0 (0/13)	
IgG3	N/A	CD4bs	RSC3	RV305_wk26	0.0 (0/20)		0.0 (0/18)		0.0 (0/19)		0.0 (0/13)	
IgG3	N/A	CD4bs	RSC3	RV305_wk48	0.0 (0/20)		0.0 (0/18)		0.0 (0/19)		0.0 (0/13)	
IgG3	N/A	CD4bs	RSC3	RV305_wk72	0.0 (0/20)		0.0 (0/18)		0.0 (0/18)		0.0 (0/13)	
IgG3	N/A	CD4bs	RSC3_P363Npair	RV144_wk26	0.0 (0/17)		0.0 (0/15)		0.0 (0/17)		0.0 (0/10)	
IgG3	N/A	CD4bs	RSC3_P363Npair	RV305_wk0	0.0 (0/20)		0.0 (0/18)		0.0 (0/19)		0.0 (0/13)	
IgG3	N/A	CD4bs	RSC3_P363Npair	RV305_wk2	0.0 (0/20)		0.0 (0/18)		0.0 (0/19)		0.0 (0/13)	
IgG3	N/A	CD4bs	RSC3_P363Npair	RV305_wk24	0.0 (0/20)		0.0 (0/18)		0.0 (0/19)		0.0 (0/13)	
IgG3	N/A	CD4bs	RSC3_P363Npair	RV305_wk26	0.0 (0/20)		0.0 (0/18)		0.0 (0/19)		0.0 (0/13)	
IgG3	N/A	CD4bs	RSC3_P363Npair	RV305_wk48	0.0 (0/20)		0.0 (0/18)		0.0 (0/19)		0.0 (0/13)	
IgG3	N/A	CD4bs	RSC3_P363Npair	RV305_wk72	0.0 (0/20)		0.0 (0/18)		0.0 (0/18)		0.0 (0/13)	
IgG3	N/A	Gag (non-Env)	p24	RV144_wk26	35.3 (6/17)	1201	26.7 (4/15)	642	17.6 (3/17)	875	30.0 (3/10)	2298
IgG3	N/A	Gag (non-Env)	p24	RV305_wk0	0.0 (0/20)		0.0 (0/18)		0.0 (0/19)		0.0 (0/13)	
IgG3	N/A	Gag (non-Env)	p24	RV305_wk2	10.0 (2/20)	6276	5.6 (1/18)	262	0.0 (0/19)		0.0 (0/13)	
IgG3	N/A	Gag (non-Env)	p24	RV305_wk24	5.0 (1/20)	1457	0.0 (0/18)		0.0 (0/19)		0.0 (0/13)	
IgG3	N/A	Gag (non-Env)	p24	RV305_wk26	15.0 (3/20)	2080	0.0 (0/18)		5.3 (1/19)	3339	0.0 (0/13)	
IgG3	N/A	Gag (non-Env)	p24	RV305_wk48	5.0 (1/20)	1581	0.0 (0/18)		5.3 (1/19)	268	0.0 (0/13)	
IgG3	N/A	Gag (non-Env)	p24	RV305_wk72	5.0 (1/20)	872	0.0 (0/18)		0.0 (0/18)		0.0 (0/13)	
