# Supplementary material for: Viral vector delivered immunogen focuses HIV-1 antibody specificity and increases durability of the circulating antibody recall response
Source: PLoS Pathog. 2023 May 31;19(5):e1011359. doi: 10.1371/journal.ppat.1011359 (PMC10284421; doi:10.1371/journal.ppat.1011359)
Supplement: S9 Table — (PDF) [file ppat.1011359.s022.pdf]

S9 Table. Median IgG3 breadth scores by group and study week.

Isotype	Panel	Group	Study Week	Number of Participant Analyzed for Breadth Score Calculation	Median Breadth Score
IgG3	V1V2 breadth panel	G1_Combination	RV144_wk0	17	<10
IgG3	V1V2 breadth panel	G1_Combination	RV144_wk26	17	120.3
IgG3	V1V2 breadth panel	G1_Combination	RV305_wk0	20	<10
IgG3	V1V2 breadth panel	G1_Combination	RV305_wk2	20	16
IgG3	V1V2 breadth panel	G1_Combination	RV305_wk24	20	<10
IgG3	V1V2 breadth panel	G1_Combination	RV305_wk26	20	20.8
IgG3	V1V2 breadth panel	G1_Combination	RV305_wk48	20	<10
IgG3	V1V2 breadth panel	G1_Combination	RV305_wk72	20	<10
IgG3	V1V2 breadth panel	G2_AIDSVAX B/E	RV144_wk0	15	<10
IgG3	V1V2 breadth panel	G2_AIDSVAX B/E	RV144_wk26	15	97.2
IgG3	V1V2 breadth panel	G2_AIDSVAX B/E	RV305_wk0	18	<10
IgG3	V1V2 breadth panel	G2_AIDSVAX B/E	RV305_wk2	18	32.2
IgG3	V1V2 breadth panel	G2_AIDSVAX B/E	RV305_wk24	18	<10
IgG3	V1V2 breadth panel	G2_AIDSVAX B/E	RV305_wk26	18	11.7
IgG3	V1V2 breadth panel	G2_AIDSVAX B/E	RV305_wk48	18	<10
IgG3	V1V2 breadth panel	G2_AIDSVAX B/E	RV305_wk72	18	<10
IgG3	V1V2 breadth panel	G3_ALVAC-HIV	RV144_wk0	17	<10
IgG3	V1V2 breadth panel	G3_ALVAC-HIV	RV144_wk26	17	170.1
IgG3	V1V2 breadth panel	G3_ALVAC-HIV	RV305_wk0	19	<10
IgG3	V1V2 breadth panel	G3_ALVAC-HIV	RV305_wk2	19	<10
IgG3	V1V2 breadth panel	G3_ALVAC-HIV	RV305_wk24	19	<10
IgG3	V1V2 breadth panel	G3_ALVAC-HIV	RV305_wk26	19	<10
IgG3	V1V2 breadth panel	G3_ALVAC-HIV	RV305_wk48	19	<10
IgG3	V1V2 breadth panel	G3_ALVAC-HIV	RV305_wk72	18	<10
IgG3	V1V2 breadth panel	RV305_Placebo	RV144_wk0	12	<10
IgG3	V1V2 breadth panel	RV305_Placebo	RV144_wk26	10	97.3
IgG3	V1V2 breadth panel	RV305_Placebo	RV305_wk0	13	<10
IgG3	V1V2 breadth panel	RV305_Placebo	RV305_wk2	13	<10
IgG3	V1V2 breadth panel	RV305_Placebo	RV305_wk24	13	<10
IgG3	V1V2 breadth panel	RV305_Placebo	RV305_wk26	13	<10
IgG3	V1V2 breadth panel	RV305_Placebo	RV305_wk48	13	<10
IgG3	V1V2 breadth panel	RV305_Placebo	RV305_wk72	13	<10

Isotype	Panel	Group	Study Week	Number of Participant Analyzed for Breadth Score Calculation	Median Breadth Score
IgG3	gp120 breadth panel	G1_Combination	RV144_wk0	17	<10
IgG3	gp120 breadth panel	G1_Combination	RV144_wk26	17	54.5
IgG3	gp120 breadth panel	G1_Combination	RV305_wk0	20	<10
IgG3	gp120 breadth panel	G1_Combination	RV305_wk2	20	25.7
IgG3	gp120 breadth panel	G1_Combination	RV305_wk24	20	<10
IgG3	gp120 breadth panel	G1_Combination	RV305_wk26	20	17.2
IgG3	gp120 breadth panel	G1_Combination	RV305_wk48	20	<10
IgG3	gp120 breadth panel	G1_Combination	RV305_wk72	20	<10
IgG3	gp120 breadth panel	G2_AIDSVAX B/E	RV144_wk0	15	<10
IgG3	gp120 breadth panel	G2_AIDSVAX B/E	RV144_wk26	15	87.3
IgG3	gp120 breadth panel	G2_AIDSVAX B/E	RV305_wk0	18	<10
IgG3	gp120 breadth panel	G2_AIDSVAX B/E	RV305_wk2	18	28.5
IgG3	gp120 breadth panel	G2_AIDSVAX B/E	RV305_wk24	18	<10
IgG3	gp120 breadth panel	G2_AIDSVAX B/E	RV305_wk26	18	13.3
IgG3	gp120 breadth panel	G2_AIDSVAX B/E	RV305_wk48	18	<10
IgG3	gp120 breadth panel	G2_AIDSVAX B/E	RV305_wk72	18	<10
IgG3	gp120 breadth panel	G3_ALVAC-HIV	RV144_wk0	17	<10
IgG3	gp120 breadth panel	G3_ALVAC-HIV	RV144_wk26	17	64.2
IgG3	gp120 breadth panel	G3_ALVAC-HIV	RV305_wk0	19	<10
IgG3	gp120 breadth panel	G3_ALVAC-HIV	RV305_wk2	19	<10
IgG3	gp120 breadth panel	G3_ALVAC-HIV	RV305_wk24	19	<10
IgG3	gp120 breadth panel	G3_ALVAC-HIV	RV305_wk26	19	<10
IgG3	gp120 breadth panel	G3_ALVAC-HIV	RV305_wk48	19	<10
IgG3	gp120 breadth panel	G3_ALVAC-HIV	RV305_wk72	18	<10
IgG3	gp120 breadth panel	RV305_Placebo	RV144_wk0	12	<10
IgG3	gp120 breadth panel	RV305_Placebo	RV144_wk26	10	48.7
IgG3	gp120 breadth panel	RV305_Placebo	RV305_wk0	13	<10
IgG3	gp120 breadth panel	RV305_Placebo	RV305_wk2	13	<10
IgG3	gp120 breadth panel	RV305_Placebo	RV305_wk24	13	<10
IgG3	gp120 breadth panel	RV305_Placebo	RV305_wk26	13	<10
IgG3	gp120 breadth panel	RV305_Placebo	RV305_wk48	13	<10
IgG3	gp120 breadth panel	RV305_Placebo	RV305_wk72	13	<10

S9 Table continued

Isotype	Panel	Group	Study Week	Number of Participant Analyzed for Breadth Score Calculation	Median Breadth Score
IgG3	gp140 breadth panel	G1_Combination	RV144_wk0	17	<10
IgG3	gp140 breadth panel	G1_Combination	RV144_wk26	17	53.5
IgG3	gp140 breadth panel	G1_Combination	RV305_wk0	20	<10
IgG3	gp140 breadth panel	G1_Combination	RV305_wk2	20	19
IgG3	gp140 breadth panel	G1_Combination	RV305_wk24	20	<10
IgG3	gp140 breadth panel	G1_Combination	RV305_wk26	20	<10
IgG3	gp140 breadth panel	G1_Combination	RV305_wk48	20	<10
IgG3	gp140 breadth panel	G1_Combination	RV305_wk72	20	<10
IgG3	gp140 breadth panel	G2_AIDSVAX B/E	RV144_wk0	15	<10
IgG3	gp140 breadth panel	G2_AIDSVAX B/E	RV144_wk26	15	135.6
IgG3	gp140 breadth panel	G2_AIDSVAX B/E	RV305_wk0	18	<10
IgG3	gp140 breadth panel	G2_AIDSVAX B/E	RV305_wk2	18	30.5
IgG3	gp140 breadth panel	G2_AIDSVAX B/E	RV305_wk24	18	<10
IgG3	gp140 breadth panel	G2_AIDSVAX B/E	RV305_wk26	18	11.6
IgG3	gp140 breadth panel	G2_AIDSVAX B/E	RV305_wk48	18	<10
IgG3	gp140 breadth panel	G2_AIDSVAX B/E	RV305_wk72	18	<10
IgG3	gp140 breadth panel	G3_ALVAC-HIV	RV144_wk0	17	<10
IgG3	gp140 breadth panel	G3_ALVAC-HIV	RV144_wk26	17	68.7
IgG3	gp140 breadth panel	G3_ALVAC-HIV	RV305_wk0	19	<10
IgG3	gp140 breadth panel	G3_ALVAC-HIV	RV305_wk2	19	<10
IgG3	gp140 breadth panel	G3_ALVAC-HIV	RV305_wk24	19	<10
IgG3	gp140 breadth panel	G3_ALVAC-HIV	RV305_wk26	19	<10
IgG3	gp140 breadth panel	G3_ALVAC-HIV	RV305_wk48	19	<10
IgG3	gp140 breadth panel	G3_ALVAC-HIV	RV305_wk72	18	<10
IgG3	gp140 breadth panel	RV305_Placebo	RV144_wk0	12	<10
IgG3	gp140 breadth panel	RV305_Placebo	RV144_wk26	10	72.9
IgG3	gp140 breadth panel	RV305_Placebo	RV305_wk0	13	<10
IgG3	gp140 breadth panel	RV305_Placebo	RV305_wk2	13	<10
IgG3	gp140 breadth panel	RV305_Placebo	RV305_wk24	13	<10
IgG3	gp140 breadth panel	RV305_Placebo	RV305_wk26	13	<10
IgG3	gp140 breadth panel	RV305_Placebo	RV305_wk48	13	<10
IgG3	gp140 breadth panel	RV305_Placebo	RV305_wk72	13	<10
