# Supplementary material for: Viral vector delivered immunogen focuses HIV-1 antibody specificity and increases durability of the circulating antibody recall response
Source: PLoS Pathog. 2023 May 31;19(5):e1011359. doi: 10.1371/journal.ppat.1011359 (PMC10284421; doi:10.1371/journal.ppat.1011359)
Supplement: S10 Table — (PDF) [file ppat.1011359.s023.pdf]

S10 Table. Median IgG3 CH58 µg/mL equivalent V1V2 concentrations.

				G1-Combination		G2-AIDSVAX B/E		G3-ALVAC-HIV		RV305 Placebo	
Isotype	Clade	Antigen	Study Week	Number of Positive Responders	Median (µg/ml)	Number of Positive Responders	Median (µg/ml)	Number of Positive Responders	Median (µg/ml)	Number of Positive Responders	Median (µg/ml)
IgG3	AE	AE.A244 V1V2 tags	RV144_wk26	14	0.28	13	0.38	13	0.37	8	0.425
IgG3	AE	AE.A244 V1V2 tags	RV305_wk0	0		0		1	0.07	0	
IgG3	AE	AE.A244 V1V2 tags	RV305_wk2	9	0.14	12	0.17	1	0.07	0	
IgG3	AE	AE.A244 V1V2 tags	RV305_wk24	1	0.07	1	0.08	1	0.06	0	
IgG3	AE	AE.A244 V1V2 tags	RV305_wk26	7	0.11	5	0.13	1	0.06	0	
IgG3	AE	AE.A244 V1V2 tags	RV305_wk48	1	0.21	1	0.09	1	0.06	0	
IgG3	AE	AE.A244 V1V2 tags	RV305_wk72	1	0.21	1	0.06			0	
IgG3	B	gp70_B.CaseA2 V1/V2/169K	RV144_wk26	5	0.06	2	0.04	2	0.205	2	0.05
IgG3	B	gp70_B.CaseA2 V1/V2/169K	RV305_wk0	1	0.23	0				0	
IgG3	B	gp70_B.CaseA2 V1/V2/169K	RV305_wk2	3	0.09	1	0.02			0	
IgG3	B	gp70_B.CaseA2 V1/V2/169K	RV305_wk24	1	0.12	0				0	
IgG3	B	gp70_B.CaseA2 V1/V2/169K	RV305_wk26	2	0.095	0				0	
IgG3	B	gp70_B.CaseA2 V1/V2/169K	RV305_wk48	1	0.26	0				0	
IgG3	B	gp70_B.CaseA2 V1/V2/169K	RV305_wk72	1	0.07	0				0	
IgG3	B	gp70_B.CaseA_V1_V2	RV144_wk26	2	0.32	1	0.13	2	0.625	1	0.19
IgG3	B	gp70_B.CaseA_V1_V2	RV305_wk0	1	1.27	0				0	
IgG3	B	gp70_B.CaseA_V1_V2	RV305_wk2	3	0.35	1	0.06			0	
IgG3	B	gp70_B.CaseA_V1_V2	RV305_wk24	1	0.6	0				0	
IgG3	B	gp70_B.CaseA_V1_V2	RV305_wk26	3	0.36	0				0	
IgG3	B	gp70_B.CaseA_V1_V2	RV305_wk48	1	0.5	0				0	
IgG3	B	gp70_B.CaseA_V1_V2	RV305_wk72	1	0.37	0				0	

No median concentration values listed if there were no positive responders for a given time point.
