# Supplementary material for: Viral vector delivered immunogen focuses HIV-1 antibody specificity and increases durability of the circulating antibody recall response
Source: PLoS Pathog. 2023 May 31;19(5):e1011359. doi: 10.1371/journal.ppat.1011359 (PMC10284421; doi:10.1371/journal.ppat.1011359)
Supplement: S11 Table — (PDF) [file ppat.1011359.s024.pdf]

S11 Table. BAMA plasma binding IgG2 response rates and group median binding magnitudes (MFI) to gp120, gp140, V1V2, V3, CD4 inducible, CD4 binding site, and Gag HIV-1 regions.

					Group 1: Combination		Group 2: AIDSVAX B/E		Group 3: ALVAC-HIV		RV305_Placebo Group	
Isotype	Clade	Env Region	Antigen	Study Week	Response Rate *(Responders/ Total)*	Median MFI *Responders*	Response Rate *(Responders/ Total)*	Median MFI *Responders*	Response Rate *(Responders/ Total)*	Median MFI *Responders*	Response Rate *(Responders/ Total)*	Median MFI *Responders*
IgG2	A	gp120	51802_D11gp120.avi/293F	RV144_wk26	0.0 (0/4)		0.0 (0/1)		0.0 (0/2)			
IgG2	A	gp120	51802_D11gp120.avi/293F	RV305_wk0	0.0 (0/4)				0.0 (0/2)			
IgG2	A	gp120	51802_D11gp120.avi/293F	RV305_wk2	0.0 (0/4)		0.0 (0/2)		0.0 (0/3)			
IgG2	A	gp120	51802_D11gp120.avi/293F	RV305_wk26	0.0 (0/3)				0.0 (0/3)			
IgG2	A	gp120	51802_D11gp120.avi/293F	RV305_wk48	0.0 (0/4)		0.0 (0/3)		0.0 (0/3)			
IgG2	A	gp120	51802_D11gp120.avi/293F	RV305_wk72	0.0 (0/3)		0.0 (0/3)		0.0 (0/3)			
IgG2	B	gp120	B.6240_D11gp120/293F	RV144_wk26	0.0 (0/4)		0.0 (0/1)		0.0 (0/2)			
IgG2	B	gp120	B.6240_D11gp120/293F	RV305_wk0	0.0 (0/4)				0.0 (0/2)			
IgG2	B	gp120	B.6240_D11gp120/293F	RV305_wk2	0.0 (0/4)		0.0 (0/2)		0.0 (0/3)			
IgG2	B	gp120	B.6240_D11gp120/293F	RV305_wk26	0.0 (0/3)				0.0 (0/3)			
IgG2	B	gp120	B.6240_D11gp120/293F	RV305_wk48	0.0 (0/4)		0.0 (0/3)		0.0 (0/3)			
IgG2	B	gp120	B.6240_D11gp120/293F	RV305_wk72	0.0 (0/3)		0.0 (0/3)		0.0 (0/3)			
IgG2	B	gp120	BORI_D11gp120.avi/293F	RV144_wk26	0.0 (0/4)		0.0 (0/1)		0.0 (0/2)			
IgG2	B	gp120	BORI_D11gp120.avi/293F	RV305_wk0	0.0 (0/4)				0.0 (0/2)			
IgG2	B	gp120	BORI_D11gp120.avi/293F	RV305_wk2	0.0 (0/4)		0.0 (0/2)		0.0 (0/3)			
IgG2	B	gp120	BORI_D11gp120.avi/293F	RV305_wk26	0.0 (0/3)				0.0 (0/3)			
IgG2	B	gp120	BORI_D11gp120.avi/293F	RV305_wk48	0.0 (0/4)		0.0 (0/3)		0.0 (0/3)			
IgG2	B	gp120	BORI_D11gp120.avi/293F	RV305_wk72	0.0 (0/3)		0.0 (0/3)		0.0 (0/3)			
IgG2	B	gp120	MN gp120 gDneg/293F	RV144_wk26	0.0 (0/16)		0.0 (0/15)		0.0 (0/17)		0.0 (0/9)	
IgG2	B	gp120	MN gp120 gDneg/293F	RV305_wk0	0.0 (0/20)		0.0 (0/18)		0.0 (0/19)		0.0 (0/12)	
IgG2	B	gp120	MN gp120 gDneg/293F	RV305_wk2	5.0 (1/20)	253	5.6 (1/18)	171	0.0 (0/19)		0.0 (0/12)	
IgG2	B	gp120	MN gp120 gDneg/293F	RV305_wk24	0.0 (0/20)		0.0 (0/18)		0.0 (0/19)		0.0 (0/12)	
IgG2	B	gp120	MN gp120 gDneg/293F	RV305_wk26	0.0 (0/20)		0.0 (0/18)		0.0 (0/19)		0.0 (0/13)	
IgG2	B	gp120	MN gp120 gDneg/293F	RV305_wk48	0.0 (0/20)		0.0 (0/18)		0.0 (0/19)		0.0 (0/12)	
IgG2	B	gp120	MN gp120 gDneg/293F	RV305_wk72	0.0 (0/20)		0.0 (0/18)		0.0 (0/18)		0.0 (0/12)	
IgG2	B	gp120	TT31P.2792_D11gp120.avi/293F	RV144_wk26	0.0 (0/4)		0.0 (0/1)		0.0 (0/2)			
IgG2	B	gp120	TT31P.2792_D11gp120.avi/293F	RV305_wk0	0.0 (0/4)				0.0 (0/2)			
IgG2	B	gp120	TT31P.2792_D11gp120.avi/293F	RV305_wk2	0.0 (0/4)		50.0 (1/2)	106	0.0 (0/3)			
IgG2	B	gp120	TT31P.2792_D11gp120.avi/293F	RV305_wk26	0.0 (0/3)				0.0 (0/3)			
IgG2	B	gp120	TT31P.2792_D11gp120.avi/293F	RV305_wk48	0.0 (0/4)		0.0 (0/3)		0.0 (0/3)			
IgG2	B	gp120	TT31P.2792_D11gp120.avi/293F	RV305_wk72	0.0 (0/3)		0.0 (0/3)		0.0 (0/3)			

S11 Table continued

					Group 1: Combination		Group 2: AIDSVAX B/E		Group 3: ALVAC-HIV		RV305_Placebo Group	
Isotype	Clade	Env Region	Antigen	Study Week	Response Rate (Responders/ Total)	Median MFI Responders	Response Rate (Responders/ Total)	Median MFI Responders	Response Rate (Responders/ Total)	Median MFI Responders	Response Rate (Responders/ Total)	Median MFI Responders
IgG2	C	gp120	1086C_D7gp120.avi/293F	RV144_wk26	0.0 (0/16)		0.0 (0/15)		0.0 (0/17)		11.1 (1/9)	982
IgG2	C	gp120	1086C_D7gp120.avi/293F	RV305_wk0	0.0 (0/20)		0.0 (0/18)		0.0 (0/19)		0.0 (0/12)	
IgG2	C	gp120	1086C_D7gp120.avi/293F	RV305_wk2	10.0 (2/20)	123	0.0 (0/18)		0.0 (0/19)		0.0 (0/12)	
IgG2	C	gp120	1086C_D7gp120.avi/293F	RV305_wk24	0.0 (0/20)		0.0 (0/18)		0.0 (0/19)		0.0 (0/12)	
IgG2	C	gp120	1086C_D7gp120.avi/293F	RV305_wk26	0.0 (0/20)		0.0 (0/18)		0.0 (0/19)		0.0 (0/13)	
IgG2	C	gp120	1086C_D7gp120.avi/293F	RV305_wk48	0.0 (0/20)		0.0 (0/18)		0.0 (0/19)		0.0 (0/12)	
IgG2	C	gp120	1086C_D7gp120.avi/293F	RV305_wk72	0.0 (0/20)		0.0 (0/18)		0.0 (0/18)		0.0 (0/12)	
IgG2	C	gp120	96ZM651.D11gp120.avi	RV144_wk26	0.0 (0/4)		0.0 (0/1)		0.0 (0/2)			
IgG2	C	gp120	96ZM651.D11gp120.avi	RV305_wk0	0.0 (0/4)				0.0 (0/2)			
IgG2	C	gp120	96ZM651.D11gp120.avi	RV305_wk2	0.0 (0/4)		0.0 (0/2)		0.0 (0/3)			
IgG2	C	gp120	96ZM651.D11gp120.avi	RV305_wk26	0.0 (0/3)				0.0 (0/3)			
IgG2	C	gp120	96ZM651.D11gp120.avi	RV305_wk48	0.0 (0/4)		0.0 (0/3)		0.0 (0/3)			
IgG2	C	gp120	96ZM651.D11gp120.avi	RV305_wk72	0.0 (0/3)		0.0 (0/3)		0.0 (0/3)			
IgG2	C	gp120	TV1c8_D11gp120.avi/293F	RV144_wk26	0.0 (0/4)		0.0 (0/1)		0.0 (0/2)			
IgG2	C	gp120	TV1c8_D11gp120.avi/293F	RV305_wk0	0.0 (0/4)				0.0 (0/2)			
IgG2	C	gp120	TV1c8_D11gp120.avi/293F	RV305_wk2	0.0 (0/4)		0.0 (0/2)		0.0 (0/3)			
IgG2	C	gp120	TV1c8_D11gp120.avi/293F	RV305_wk26	0.0 (0/3)				0.0 (0/3)			
IgG2	C	gp120	TV1c8_D11gp120.avi/293F	RV305_wk48	0.0 (0/4)		0.0 (0/3)		0.0 (0/3)			
IgG2	C	gp120	TV1c8_D11gp120.avi/293F	RV305_wk72	0.0 (0/3)		0.0 (0/3)		0.0 (0/3)			
IgG2	CRF01_AE	gp120	254008_D11gp120.avi/293F	RV144_wk26	0.0 (0/4)		0.0 (0/1)		0.0 (0/2)			
IgG2	CRF01_AE	gp120	254008_D11gp120.avi/293F	RV305_wk0	0.0 (0/4)				0.0 (0/2)			
IgG2	CRF01_AE	gp120	254008_D11gp120.avi/293F	RV305_wk2	0.0 (0/4)		0.0 (0/2)		0.0 (0/3)			
IgG2	CRF01_AE	gp120	254008_D11gp120.avi/293F	RV305_wk26	0.0 (0/3)				0.0 (0/3)			
IgG2	CRF01_AE	gp120	254008_D11gp120.avi/293F	RV305_wk48	0.0 (0/4)		0.0 (0/3)		0.0 (0/3)			
IgG2	CRF01_AE	gp120	254008_D11gp120.avi/293F	RV305_wk72	0.0 (0/3)		0.0 (0/3)		0.0 (0/3)			
IgG2	CRF01_AE	gp120	92TH023 gp120 gDneg 293F mon	RV144_wk26	0.0 (0/16)		0.0 (0/15)		0.0 (0/17)		0.0 (0/9)	
IgG2	CRF01_AE	gp120	92TH023 gp120 gDneg 293F mon	RV305_wk0	0.0 (0/20)		0.0 (0/18)		0.0 (0/19)		0.0 (0/12)	
IgG2	CRF01_AE	gp120	92TH023 gp120 gDneg 293F mon	RV305_wk2	5.0 (1/20)	174	0.0 (0/18)		0.0 (0/19)		0.0 (0/12)	
IgG2	CRF01_AE	gp120	92TH023 gp120 gDneg 293F mon	RV305_wk24	0.0 (0/20)		0.0 (0/18)		0.0 (0/19)		0.0 (0/12)	
IgG2	CRF01_AE	gp120	92TH023 gp120 gDneg 293F mon	RV305_wk26	5.0 (1/20)	242	0.0 (0/18)		0.0 (0/19)		0.0 (0/13)	
IgG2	CRF01_AE	gp120	92TH023 gp120 gDneg 293F mon	RV305_wk48	0.0 (0/20)		0.0 (0/18)		0.0 (0/19)		0.0 (0/12)	
IgG2	CRF01_AE	gp120	92TH023 gp120 gDneg 293F mon	RV305_wk72	0.0 (0/20)		0.0 (0/18)		0.0 (0/18)		0.0 (0/12)	

S11 Table continued

					Group 1: Combination		Group 2: AIDSVAX B/E		Group 3: ALVAC-HIV		RV305_Placebo Group	
Isotype	Clade	Env Region	Antigen	Study Week	Response Rate (Responders/ Total)	Median MFI Responders	Response Rate (Responders/ Total)	Median MFI Responders	Response Rate (Responders/ Total)	Median MFI Responders	Response Rate (Responders/ Total)	Median MFI Responders
IgG2	CRF01_AE	gp120	A244 D11gp120_avi	RV144_wk26	0.0 (0/16)		0.0 (0/15)		0.0 (0/17)		0.0 (0/9)	
IgG2	CRF01_AE	gp120	A244 D11gp120_avi	RV305_wk0	0.0 (0/20)		0.0 (0/18)		0.0 (0/19)		0.0 (0/12)	
IgG2	CRF01_AE	gp120	A244 D11gp120_avi	RV305_wk2	10.0 (2/20)	170	0.0 (0/18)		0.0 (0/19)		0.0 (0/12)	
IgG2	CRF01_AE	gp120	A244 D11gp120_avi	RV305_wk24	0.0 (0/20)		0.0 (0/18)		0.0 (0/19)		0.0 (0/12)	
IgG2	CRF01_AE	gp120	A244 D11gp120_avi	RV305_wk26	5.0 (1/20)	260	0.0 (0/18)		0.0 (0/19)		0.0 (0/13)	
IgG2	CRF01_AE	gp120	A244 D11gp120_avi	RV305_wk48	0.0 (0/20)		0.0 (0/18)		0.0 (0/19)		0.0 (0/12)	
IgG2	CRF01_AE	gp120	A244 D11gp120_avi	RV305_wk72	0.0 (0/20)		0.0 (0/18)		0.0 (0/18)		0.0 (0/12)	
IgG2	CRF01_AE	gp120	CM235 gp120	RV144_wk26	0.0 (0/16)		0.0 (0/15)		0.0 (0/17)		0.0 (0/9)	
IgG2	CRF01_AE	gp120	CM235 gp120	RV305_wk0	0.0 (0/20)		0.0 (0/18)		0.0 (0/19)		0.0 (0/12)	
IgG2	CRF01_AE	gp120	CM235 gp120	RV305_wk2	0.0 (0/20)		0.0 (0/18)		0.0 (0/19)		0.0 (0/12)	
IgG2	CRF01_AE	gp120	CM235 gp120	RV305_wk24	0.0 (0/20)		0.0 (0/18)		5.3 (1/19)	1293	0.0 (0/12)	
IgG2	CRF01_AE	gp120	CM235 gp120	RV305_wk26	0.0 (0/20)		0.0 (0/18)		0.0 (0/19)		0.0 (0/13)	
IgG2	CRF01_AE	gp120	CM235 gp120	RV305_wk48	0.0 (0/20)		0.0 (0/18)		0.0 (0/19)		0.0 (0/12)	
IgG2	CRF01_AE	gp120	CM235 gp120	RV305_wk72	5.0 (1/20)	1976	0.0 (0/18)		0.0 (0/18)		0.0 (0/12)	
IgG2	CRF07_BC	gp120	BJOX002_D11gp120.avi/293F	RV144_wk26	0.0 (0/4)		0.0 (0/1)		0.0 (0/2)			
IgG2	CRF07_BC	gp120	BJOX002_D11gp120.avi/293F	RV305_wk0	0.0 (0/4)				0.0 (0/2)			
IgG2	CRF07_BC	gp120	BJOX002_D11gp120.avi/293F	RV305_wk2	0.0 (0/4)		0.0 (0/2)		0.0 (0/3)			
IgG2	CRF07_BC	gp120	BJOX002_D11gp120.avi/293F	RV305_wk26	0.0 (0/3)				0.0 (0/3)			
IgG2	CRF07_BC	gp120	BJOX002_D11gp120.avi/293F	RV305_wk48	0.0 (0/4)		0.0 (0/3)		0.0 (0/3)			
IgG2	CRF07_BC	gp120	BJOX002_D11gp120.avi/293F	RV305_wk72	0.0 (0/3)		0.0 (0/3)		0.0 (0/3)			
IgG2	CRF07_BC	gp120	CNE20_D11gp120.avi/293F	RV144_wk26	0.0 (0/4)		0.0 (0/1)		0.0 (0/2)			
IgG2	CRF07_BC	gp120	CNE20_D11gp120.avi/293F	RV305_wk0	0.0 (0/4)				0.0 (0/2)			
IgG2	CRF07_BC	gp120	CNE20_D11gp120.avi/293F	RV305_wk2	0.0 (0/3)		0.0 (0/2)		0.0 (0/3)			
IgG2	CRF07_BC	gp120	CNE20_D11gp120.avi/293F	RV305_wk26	0.0 (0/3)				0.0 (0/3)			
IgG2	CRF07_BC	gp120	CNE20_D11gp120.avi/293F	RV305_wk48	0.0 (0/4)		0.0 (0/3)		0.0 (0/3)			
IgG2	CRF07_BC	gp120	CNE20_D11gp120.avi/293F	RV305_wk72	0.0 (0/3)		0.0 (0/3)		0.0 (0/3)			
IgG2	Consensus	gp120	Con 6 gp120/B	RV144_wk26	0.0 (0/16)		0.0 (0/15)		0.0 (0/17)		0.0 (0/9)	
IgG2	Consensus	gp120	Con 6 gp120/B	RV305_wk0	0.0 (0/20)		0.0 (0/18)		0.0 (0/19)		0.0 (0/12)	
IgG2	Consensus	gp120	Con 6 gp120/B	RV305_wk2	5.0 (1/20)	385	0.0 (0/18)		0.0 (0/19)		0.0 (0/12)	
IgG2	Consensus	gp120	Con 6 gp120/B	RV305_wk24	0.0 (0/20)		0.0 (0/18)		0.0 (0/19)		0.0 (0/12)	
IgG2	Consensus	gp120	Con 6 gp120/B	RV305_wk26	0.0 (0/20)		0.0 (0/18)		0.0 (0/19)		0.0 (0/13)	
IgG2	Consensus	gp120	Con 6 gp120/B	RV305_wk48	0.0 (0/20)		0.0 (0/18)		0.0 (0/19)		0.0 (0/12)	
IgG2	Consensus	gp120	Con 6 gp120/B	RV305_wk72	0.0 (0/20)		0.0 (0/18)		0.0 (0/18)		0.0 (0/12)	

S11 Table continued

					Group 1: Combination		Group 2: AIDSVAX B/E		Group 3: ALVAC-HIV		RV305_Placebo Group	
Isotype	Clade	Env Region	Antigen	Study Week	Response Rate (Responders/ Total)	Median MFI Responders	Response Rate (Responders/ Total)	Median MFI Responders	Response Rate (Responders/ Total)	Median MFI Responders	Response Rate (Responders/ Total)	Median MFI Responders
IgG2	A	gp140	9004S.gp140C.avi	RV144_wk26	0.0 (0/4)		0.0 (0/1)		0.0 (0/2)			
IgG2	A	gp140	9004S.gp140C.avi	RV305_wk0	0.0 (0/4)				0.0 (0/2)			
IgG2	A	gp140	9004S.gp140C.avi	RV305_wk2	0.0 (0/4)		0.0 (0/2)		0.0 (0/3)			
IgG2	A	gp140	9004S.gp140C.avi	RV305_wk26	0.0 (0/3)				0.0 (0/3)			
IgG2	A	gp140	9004S.gp140C.avi	RV305_wk48	0.0 (0/4)		0.0 (0/3)		0.0 (0/3)			
IgG2	A	gp140	9004S.gp140C.avi	RV305_wk72	0.0 (0/3)		0.0 (0/3)		0.0 (0/3)			
IgG2	B	gp140	RHPA4259_C7.gp140C.avi	RV144_wk26	0.0 (0/4)		0.0 (0/1)		0.0 (0/2)			
IgG2	B	gp140	RHPA4259_C7.gp140C.avi	RV305_wk0	0.0 (0/4)				0.0 (0/2)			
IgG2	B	gp140	RHPA4259_C7.gp140C.avi	RV305_wk2	0.0 (0/4)		0.0 (0/2)		0.0 (0/3)			
IgG2	B	gp140	RHPA4259_C7.gp140C.avi	RV305_wk26	0.0 (0/3)				0.0 (0/3)			
IgG2	B	gp140	RHPA4259_C7.gp140C.avi	RV305_wk48	0.0 (0/4)		0.0 (0/3)		0.0 (0/3)			
IgG2	B	gp140	RHPA4259_C7.gp140C.avi	RV305_wk72	0.0 (0/3)		0.0 (0/3)		0.0 (0/3)			
IgG2	B	gp140	SC42261_gp140.avi/293F	RV144_wk26	0.0 (0/4)		0.0 (0/1)		0.0 (0/2)			
IgG2	B	gp140	SC42261_gp140.avi/293F	RV305_wk0	0.0 (0/4)				0.0 (0/2)			
IgG2	B	gp140	SC42261_gp140.avi/293F	RV305_wk2	0.0 (0/4)		0.0 (0/2)		0.0 (0/3)			
IgG2	B	gp140	SC42261_gp140.avi/293F	RV305_wk26	0.0 (0/3)				0.0 (0/3)			
IgG2	B	gp140	SC42261_gp140.avi/293F	RV305_wk48	0.0 (0/4)		0.0 (0/3)		0.0 (0/3)			
IgG2	B	gp140	SC42261_gp140.avi/293F	RV305_wk72	0.0 (0/3)		0.0 (0/3)		0.0 (0/3)			
IgG2	B	gp140	WITO4160.gp140C.avi	RV144_wk26	0.0 (0/4)		0.0 (0/1)		0.0 (0/2)			
IgG2	B	gp140	WITO4160.gp140C.avi	RV305_wk0	0.0 (0/4)				0.0 (0/2)			
IgG2	B	gp140	WITO4160.gp140C.avi	RV305_wk2	0.0 (0/4)		0.0 (0/2)		0.0 (0/3)			
IgG2	B	gp140	WITO4160.gp140C.avi	RV305_wk26	0.0 (0/3)				0.0 (0/3)			
IgG2	B	gp140	WITO4160.gp140C.avi	RV305_wk48	0.0 (0/4)		0.0 (0/3)		0.0 (0/3)			
IgG2	B	gp140	WITO4160.gp140C.avi	RV305_wk72	0.0 (0/3)		0.0 (0/3)		0.0 (0/3)			
IgG2	C	gp140	1086C gp140C_avi	RV144_wk26	0.0 (0/16)		0.0 (0/15)		0.0 (0/17)		0.0 (0/9)	
IgG2	C	gp140	1086C gp140C_avi	RV305_wk0	0.0 (0/20)		0.0 (0/18)		0.0 (0/19)		0.0 (0/12)	
IgG2	C	gp140	1086C gp140C_avi	RV305_wk2	10.0 (2/20)	223	0.0 (0/18)		0.0 (0/19)		0.0 (0/12)	
IgG2	C	gp140	1086C gp140C_avi	RV305_wk24	0.0 (0/20)		0.0 (0/18)		0.0 (0/19)		0.0 (0/12)	
IgG2	C	gp140	1086C gp140C_avi	RV305_wk26	5.0 (1/20)	154	0.0 (0/18)		0.0 (0/19)		0.0 (0/13)	
IgG2	C	gp140	1086C gp140C_avi	RV305_wk48	0.0 (0/20)		0.0 (0/18)		0.0 (0/19)		0.0 (0/12)	
IgG2	C	gp140	1086C gp140C_avi	RV305_wk72	0.0 (0/20)		0.0 (0/18)		0.0 (0/18)		0.0 (0/12)	

S11 Table continued

					Group 1: Combination		Group 2: AIDSVAX B/E		Group 3: ALVAC-HIV		RV305_Placebo Group	
Isotype	Clade	Env Region	Antigen	Study Week	Response Rate (Responders/ Total)	Median MFI Responders	Response Rate (Responders/ Total)	Median MFI Responders	Response Rate (Responders/ Total)	Median MFI Responders	Response Rate (Responders/ Total)	Median MFI Responders
IgG2	C	gp140	BF1266_gp140C.avi/293F	RV144_wk26	0.0 (0/4)		0.0 (0/1)		0.0 (0/2)			
IgG2	C	gp140	BF1266_gp140C.avi/293F	RV305_wk0	0.0 (0/4)				0.0 (0/2)			
IgG2	C	gp140	BF1266_gp140C.avi/293F	RV305_wk2	0.0 (0/4)		0.0 (0/2)		0.0 (0/3)			
IgG2	C	gp140	BF1266_gp140C.avi/293F	RV305_wk26	0.0 (0/3)				0.0 (0/3)			
IgG2	C	gp140	BF1266_gp140C.avi/293F	RV305_wk48	0.0 (0/4)		0.0 (0/3)		0.0 (0/3)			
IgG2	C	gp140	BF1266_gp140C.avi/293F	RV305_wk72	0.0 (0/3)		0.0 (0/3)		0.0 (0/3)			
IgG2	C	gp140	C.CH505TF_gp140/293F	RV144_wk26	0.0 (0/4)		0.0 (0/1)		0.0 (0/2)			
IgG2	C	gp140	C.CH505TF_gp140/293F	RV305_wk0	0.0 (0/4)				0.0 (0/2)			
IgG2	C	gp140	C.CH505TF_gp140/293F	RV305_wk2	0.0 (0/4)		0.0 (0/2)		0.0 (0/3)			
IgG2	C	gp140	C.CH505TF_gp140/293F	RV305_wk26	0.0 (0/3)				0.0 (0/3)			
IgG2	C	gp140	C.CH505TF_gp140/293F	RV305_wk48	0.0 (0/4)		0.0 (0/3)		0.0 (0/3)			
IgG2	C	gp140	C.CH505TF_gp140/293F	RV305_wk72	0.0 (0/3)		0.0 (0/3)		0.0 (0/3)			
IgG2	Consensus	gp140	Con S gp140 CFI	RV144_wk26	0.0 (0/16)		0.0 (0/15)		0.0 (0/17)		0.0 (0/9)	
IgG2	Consensus	gp140	Con S gp140 CFI	RV305_wk0	0.0 (0/20)		0.0 (0/18)		0.0 (0/19)		0.0 (0/12)	
IgG2	Consensus	gp140	Con S gp140 CFI	RV305_wk2	5.0 (1/20)	123	0.0 (0/18)		0.0 (0/19)		0.0 (0/12)	
IgG2	Consensus	gp140	Con S gp140 CFI	RV305_wk24	0.0 (0/20)		0.0 (0/18)		0.0 (0/19)		0.0 (0/12)	
IgG2	Consensus	gp140	Con S gp140 CFI	RV305_wk26	0.0 (0/20)		0.0 (0/18)		0.0 (0/19)		0.0 (0/13)	
IgG2	Consensus	gp140	Con S gp140 CFI	RV305_wk48	0.0 (0/20)		0.0 (0/18)		0.0 (0/19)		0.0 (0/12)	
IgG2	Consensus	gp140	Con S gp140 CFI	RV305_wk72	0.0 (0/20)		0.0 (0/18)		0.0 (0/18)		0.0 (0/12)	
IgG2	Consensus CRF01_AE	gp140	AE.01.con_env03 gp140CF_avi	RV144_wk26	0.0 (0/16)		0.0 (0/15)		0.0 (0/17)		0.0 (0/9)	
IgG2	Consensus CRF01_AE	gp140	AE.01.con_env03 gp140CF_avi	RV305_wk0	0.0 (0/20)		0.0 (0/18)		0.0 (0/19)		0.0 (0/12)	
IgG2	Consensus CRF01_AE	gp140	AE.01.con_env03 gp140CF_avi	RV305_wk2	5.0 (1/20)	193	0.0 (0/18)		0.0 (0/19)		0.0 (0/12)	
IgG2	Consensus CRF01_AE	gp140	AE.01.con_env03 gp140CF_avi	RV305_wk24	0.0 (0/20)		0.0 (0/18)		0.0 (0/19)		0.0 (0/12)	
IgG2	Consensus CRF01_AE	gp140	AE.01.con_env03 gp140CF_avi	RV305_wk26	5.0 (1/20)	236	0.0 (0/18)		0.0 (0/19)		0.0 (0/13)	
IgG2	Consensus CRF01_AE	gp140	AE.01.con_env03 gp140CF_avi	RV305_wk48	0.0 (0/20)		0.0 (0/18)		0.0 (0/19)		0.0 (0/12)	
IgG2	Consensus CRF01_AE	gp140	AE.01.con_env03 gp140CF_avi	RV305_wk72	0.0 (0/20)		0.0 (0/18)		0.0 (0/18)		0.0 (0/12)	

S11 Table continued

					Group 1: Combination		Group 2: AIDSVAX B/E		Group 3: ALVAC-HIV		RV305_Placebo Group	
Isotype	Clade	Env Region	Antigen	Study Week	Response Rate (Responders/ Total)	Median MFI Responders	Response Rate (Responders/ Total)	Median MFI Responders	Response Rate (Responders/ Total)	Median MFI Responders	Response Rate (Responders/ Total)	Median MFI Responders
IgG2	A	V1V2	gp70-191084_B7 V1V2	RV144_wk26	0.0 (0/4)		0.0 (0/2)		33.3 (1/3)	115		
IgG2	A	V1V2	gp70-191084_B7 V1V2	RV305_wk0	0.0 (0/4)		0.0 (0/3)		0.0 (0/3)			
IgG2	A	V1V2	gp70-191084_B7 V1V2	RV305_wk2	50.0 (2/4)	121	0.0 (0/3)		0.0 (0/3)			
IgG2	A	V1V2	gp70-191084_B7 V1V2	RV305_wk26	0.0 (0/4)		0.0 (0/3)		0.0 (0/2)			
IgG2	A	V1V2	gp70-191084_B7 V1V2	RV305_wk48	0.0 (0/4)		0.0 (0/3)		0.0 (0/3)			
IgG2	A	V1V2	gp70-191084_B7 V1V2	RV305_wk72	0.0 (0/4)		0.0 (0/3)		0.0 (0/3)			
IgG2	B	V1V2	gp70-62357.14 V1V2	RV144_wk26	0.0 (0/4)		0.0 (0/2)		0.0 (0/3)			
IgG2	B	V1V2	gp70-62357.14 V1V2	RV305_wk0	0.0 (0/4)		0.0 (0/3)		0.0 (0/3)			
IgG2	B	V1V2	gp70-62357.14 V1V2	RV305_wk2	0.0 (0/4)		0.0 (0/3)		0.0 (0/3)			
IgG2	B	V1V2	gp70-62357.14 V1V2	RV305_wk26	0.0 (0/4)		0.0 (0/3)		0.0 (0/2)			
IgG2	B	V1V2	gp70-62357.14 V1V2	RV305_wk48	0.0 (0/4)		0.0 (0/3)		0.0 (0/3)			
IgG2	B	V1V2	gp70-62357.14 V1V2	RV305_wk72	0.0 (0/4)		0.0 (0/3)		0.0 (0/3)			
IgG2	B	V1V2	gp70-700010058 V1V2	RV144_wk26	0.0 (0/4)		0.0 (0/2)		0.0 (0/3)			
IgG2	B	V1V2	gp70-700010058 V1V2	RV305_wk0	0.0 (0/4)		0.0 (0/3)		0.0 (0/3)			
IgG2	B	V1V2	gp70-700010058 V1V2	RV305_wk2	0.0 (0/4)		0.0 (0/3)		0.0 (0/3)			
IgG2	B	V1V2	gp70-700010058 V1V2	RV305_wk26	0.0 (0/4)		0.0 (0/3)		0.0 (0/3)			
IgG2	B	V1V2	gp70-700010058 V1V2	RV305_wk48	0.0 (0/4)		0.0 (0/3)		0.0 (0/3)			
IgG2	B	V1V2	gp70-700010058 V1V2	RV305_wk72	0.0 (0/4)		0.0 (0/3)		0.0 (0/3)			
IgG2	B	V1V2	gp70-RHPA4259.7 V1V2	RV144_wk26	0.0 (0/4)		0.0 (0/2)		0.0 (0/3)			
IgG2	B	V1V2	gp70-RHPA4259.7 V1V2	RV305_wk0	0.0 (0/4)		0.0 (0/3)		0.0 (0/3)			
IgG2	B	V1V2	gp70-RHPA4259.7 V1V2	RV305_wk2	0.0 (0/4)		0.0 (0/3)		0.0 (0/3)			
IgG2	B	V1V2	gp70-RHPA4259.7 V1V2	RV305_wk26	0.0 (0/4)		0.0 (0/3)		0.0 (0/3)			
IgG2	B	V1V2	gp70-RHPA4259.7 V1V2	RV305_wk48	0.0 (0/4)		0.0 (0/3)		0.0 (0/3)			
IgG2	B	V1V2	gp70-RHPA4259.7 V1V2	RV305_wk72	0.0 (0/4)		0.0 (0/3)		0.0 (0/3)			
IgG2	B	V1V2	gp70-TT31P.2F10.2792 V1V2	RV144_wk26	0.0 (0/4)		0.0 (0/2)		0.0 (0/3)			
IgG2	B	V1V2	gp70-TT31P.2F10.2792 V1V2	RV305_wk0	0.0 (0/4)		0.0 (0/3)		0.0 (0/3)			
IgG2	B	V1V2	gp70-TT31P.2F10.2792 V1V2	RV305_wk2	0.0 (0/4)		0.0 (0/3)		0.0 (0/3)			
IgG2	B	V1V2	gp70-TT31P.2F10.2792 V1V2	RV305_wk26	0.0 (0/4)		0.0 (0/3)		0.0 (0/3)			
IgG2	B	V1V2	gp70-TT31P.2F10.2792 V1V2	RV305_wk48	0.0 (0/4)		0.0 (0/3)		0.0 (0/3)			
IgG2	B	V1V2	gp70-TT31P.2F10.2792 V1V2	RV305_wk72	0.0 (0/4)		0.0 (0/3)		0.0 (0/3)			

					Group 1: Combination		Group 2: AIDSVAX B/E		Group 3: ALVAC-HIV		RV305_Placebo Group	
Isotype	Clade	Env Region	Antigen	Study Week	Response Rate (Responders/ Total)	Median MFI Responders	Response Rate (Responders/ Total)	Median MFI Responders	Response Rate (Responders/ Total)	Median MFI Responders	Response Rate (Responders/ Total)	Median MFI Responders
IgG2	B	V1V2	gp70_B.CaseA2 V1/V2/169K	RV144_wk26	0.0 (0/16)		0.0 (0/15)		0.0 (0/17)		0.0 (0/9)	
IgG2	B	V1V2	gp70_B.CaseA2 V1/V2/169K	RV305_wk0	0.0 (0/20)		0.0 (0/18)		0.0 (0/19)		0.0 (0/12)	
IgG2	B	V1V2	gp70_B.CaseA2 V1/V2/169K	RV305_wk2	0.0 (0/20)		0.0 (0/18)		0.0 (0/19)		0.0 (0/12)	
IgG2	B	V1V2	gp70_B.CaseA2 V1/V2/169K	RV305_wk24	0.0 (0/20)		0.0 (0/18)		0.0 (0/19)		0.0 (0/12)	
IgG2	B	V1V2	gp70_B.CaseA2 V1/V2/169K	RV305_wk26	0.0 (0/20)		0.0 (0/18)		0.0 (0/19)		0.0 (0/13)	
IgG2	B	V1V2	gp70_B.CaseA2 V1/V2/169K	RV305_wk48	0.0 (0/20)		0.0 (0/18)		0.0 (0/19)		0.0 (0/12)	
IgG2	B	V1V2	gp70_B.CaseA2 V1/V2/169K	RV305_wk72	0.0 (0/20)		0.0 (0/18)		0.0 (0/18)		0.0 (0/12)	
IgG2	B	V1V2	gp70_B.CaseA_V1_V2	RV144_wk26	0.0 (0/16)		0.0 (0/15)		0.0 (0/17)		0.0 (0/9)	
IgG2	B	V1V2	gp70_B.CaseA_V1_V2	RV305_wk0	0.0 (0/20)		0.0 (0/18)		0.0 (0/19)		0.0 (0/12)	
IgG2	B	V1V2	gp70_B.CaseA_V1_V2	RV305_wk2	0.0 (0/20)		0.0 (0/18)		0.0 (0/19)		0.0 (0/12)	
IgG2	B	V1V2	gp70_B.CaseA_V1_V2	RV305_wk24	0.0 (0/20)		0.0 (0/18)		0.0 (0/19)		0.0 (0/12)	
IgG2	B	V1V2	gp70_B.CaseA_V1_V2	RV305_wk26	0.0 (0/20)		0.0 (0/18)		0.0 (0/19)		0.0 (0/13)	
IgG2	B	V1V2	gp70_B.CaseA_V1_V2	RV305_wk48	0.0 (0/20)		0.0 (0/18)		0.0 (0/19)		0.0 (0/12)	
IgG2	B	V1V2	gp70_B.CaseA_V1_V2	RV305_wk72	0.0 (0/20)		0.0 (0/18)		0.0 (0/18)		0.0 (0/12)	
IgG2	C	V1V2	C.1086C_V1_V2 Tags	RV144_wk26	0.0 (0/16)		0.0 (0/15)		0.0 (0/17)		0.0 (0/9)	
IgG2	C	V1V2	C.1086C_V1_V2 Tags	RV305_wk0	0.0 (0/20)		0.0 (0/18)		0.0 (0/19)		0.0 (0/12)	
IgG2	C	V1V2	C.1086C_V1_V2 Tags	RV305_wk2	5.0 (1/20)	282	5.6 (1/18)	119	0.0 (0/19)		0.0 (0/12)	
IgG2	C	V1V2	C.1086C_V1_V2 Tags	RV305_wk24	0.0 (0/20)		0.0 (0/18)		0.0 (0/19)		0.0 (0/12)	
IgG2	C	V1V2	C.1086C_V1_V2 Tags	RV305_wk26	0.0 (0/20)		0.0 (0/18)		0.0 (0/19)		0.0 (0/13)	
IgG2	C	V1V2	C.1086C_V1_V2 Tags	RV305_wk48	0.0 (0/20)		0.0 (0/18)		0.0 (0/19)		0.0 (0/12)	
IgG2	C	V1V2	C.1086C_V1_V2 Tags	RV305_wk72	0.0 (0/20)		0.0 (0/18)		0.0 (0/18)		0.0 (0/12)	
IgG2	C	V1V2	gp70-001428.2.42 V1V2	RV144_wk26	0.0 (0/4)		0.0 (0/2)		0.0 (0/3)			
IgG2	C	V1V2	gp70-001428.2.42 V1V2	RV305_wk0	0.0 (0/4)		0.0 (0/3)		0.0 (0/3)			
IgG2	C	V1V2	gp70-001428.2.42 V1V2	RV305_wk2	0.0 (0/4)		0.0 (0/3)		0.0 (0/3)			
IgG2	C	V1V2	gp70-001428.2.42 V1V2	RV305_wk26	0.0 (0/4)		0.0 (0/3)		0.0 (0/3)			
IgG2	C	V1V2	gp70-001428.2.42 V1V2	RV305_wk48	0.0 (0/4)		0.0 (0/3)		0.0 (0/3)			
IgG2	C	V1V2	gp70-001428.2.42 V1V2	RV305_wk72	0.0 (0/4)		0.0 (0/3)		0.0 (0/3)			
IgG2	C	V1V2	gp70-7060101641 V1V2	RV144_wk26	0.0 (0/4)		0.0 (0/2)		0.0 (0/3)			
IgG2	C	V1V2	gp70-7060101641 V1V2	RV305_wk0	0.0 (0/4)		0.0 (0/3)		0.0 (0/3)			
IgG2	C	V1V2	gp70-7060101641 V1V2	RV305_wk2	0.0 (0/4)		0.0 (0/3)		0.0 (0/3)			
IgG2	C	V1V2	gp70-7060101641 V1V2	RV305_wk26	0.0 (0/4)		0.0 (0/3)		0.0 (0/3)			
IgG2	C	V1V2	gp70-7060101641 V1V2	RV305_wk48	0.0 (0/4)		0.0 (0/3)		0.0 (0/3)			
IgG2	C	V1V2	gp70-7060101641 V1V2	RV305_wk72	0.0 (0/4)		0.0 (0/3)		0.0 (0/3)			

S11 Table continued

					Group 1: Combination		Group 2: AIDSVAX B/E		Group 3: ALVAC-HIV		RV305_Placebo Group	
Isotype	Clade	Env Region	Antigen	Study Week	Response Rate (Responders/ Total)	Median MFI Responders	Response Rate (Responders/ Total)	Median MFI Responders	Response Rate (Responders/ Total)	Median MFI Responders	Response Rate (Responders/ Total)	Median MFI Responders
IgG2	C	V1V2	gp70-96ZM651.02 V1v2	RV144_wk26	0.0 (0/16)		0.0 (0/15)		5.9 (1/17)	134	0.0 (0/9)	
IgG2	C	V1V2	gp70-96ZM651.02 V1v2	RV305_wk0	0.0 (0/20)		0.0 (0/18)		0.0 (0/19)		0.0 (0/12)	
IgG2	C	V1V2	gp70-96ZM651.02 V1v2	RV305_wk2	0.0 (0/20)		0.0 (0/18)		0.0 (0/19)		0.0 (0/12)	
IgG2	C	V1V2	gp70-96ZM651.02 V1v2	RV305_wk24	0.0 (0/20)		0.0 (0/18)		0.0 (0/19)		0.0 (0/12)	
IgG2	C	V1V2	gp70-96ZM651.02 V1v2	RV305_wk26	0.0 (0/20)		0.0 (0/18)		0.0 (0/19)		0.0 (0/13)	
IgG2	C	V1V2	gp70-96ZM651.02 V1v2	RV305_wk48	0.0 (0/20)		0.0 (0/18)		0.0 (0/19)		0.0 (0/12)	
IgG2	C	V1V2	gp70-96ZM651.02 V1v2	RV305_wk72	0.0 (0/20)		0.0 (0/18)		0.0 (0/18)		0.0 (0/12)	
IgG2	C	V1V2	gp70-BF1266_431a_V1V2	RV144_wk26	0.0 (0/4)		0.0 (0/2)		0.0 (0/3)			
IgG2	C	V1V2	gp70-BF1266_431a_V1V2	RV305_wk0	0.0 (0/4)		0.0 (0/3)		0.0 (0/3)			
IgG2	C	V1V2	gp70-BF1266_431a_V1V2	RV305_wk2	0.0 (0/4)		0.0 (0/3)		0.0 (0/3)			
IgG2	C	V1V2	gp70-BF1266_431a_V1V2	RV305_wk26	0.0 (0/4)		0.0 (0/3)		0.0 (0/3)			
IgG2	C	V1V2	gp70-BF1266_431a_V1V2	RV305_wk48	0.0 (0/4)		0.0 (0/3)		0.0 (0/3)			
IgG2	C	V1V2	gp70-BF1266_431a_V1V2	RV305_wk72	0.0 (0/4)		0.0 (0/3)		0.0 (0/3)			
IgG2	C	V1V2	gp70-CAP210.2.00.E8 V1V2	RV144_wk26	0.0 (0/4)		0.0 (0/2)		0.0 (0/3)			
IgG2	C	V1V2	gp70-CAP210.2.00.E8 V1V2	RV305_wk0	0.0 (0/4)		0.0 (0/3)		0.0 (0/3)			
IgG2	C	V1V2	gp70-CAP210.2.00.E8 V1V2	RV305_wk2	0.0 (0/4)		0.0 (0/3)		0.0 (0/3)			
IgG2	C	V1V2	gp70-CAP210.2.00.E8 V1V2	RV305_wk26	0.0 (0/4)		0.0 (0/3)		0.0 (0/3)			
IgG2	C	V1V2	gp70-CAP210.2.00.E8 V1V2	RV305_wk48	0.0 (0/4)		0.0 (0/3)		0.0 (0/3)			
IgG2	C	V1V2	gp70-CAP210.2.00.E8 V1V2	RV305_wk72	0.0 (0/4)		0.0 (0/3)		0.0 (0/3)			
IgG2	C	V1V2	gp70-Ce1086_B2 V1V2	RV144_wk26	6.3 (1/16)	194	6.7 (1/15)	107	5.9 (1/17)	138	0.0 (0/9)	
IgG2	C	V1V2	gp70-Ce1086_B2 V1V2	RV305_wk0	0.0 (0/20)		0.0 (0/18)		0.0 (0/19)		0.0 (0/12)	
IgG2	C	V1V2	gp70-Ce1086_B2 V1V2	RV305_wk2	0.0 (0/20)		5.6 (1/18)	202	0.0 (0/19)		0.0 (0/12)	
IgG2	C	V1V2	gp70-Ce1086_B2 V1V2	RV305_wk24	0.0 (0/20)		0.0 (0/18)		0.0 (0/19)		0.0 (0/12)	
IgG2	C	V1V2	gp70-Ce1086_B2 V1V2	RV305_wk26	5.0 (1/20)	105	0.0 (0/18)		0.0 (0/19)		0.0 (0/13)	
IgG2	C	V1V2	gp70-Ce1086_B2 V1V2	RV305_wk48	0.0 (0/20)		0.0 (0/18)		0.0 (0/19)		0.0 (0/12)	
IgG2	C	V1V2	gp70-Ce1086_B2 V1V2	RV305_wk72	0.0 (0/20)		0.0 (0/18)		0.0 (0/18)		0.0 (0/12)	
IgG2	C	V1V2	gp70-TV1.21 V1V2	RV144_wk26	0.0 (0/4)		0.0 (0/2)		0.0 (0/3)			
IgG2	C	V1V2	gp70-TV1.21 V1V2	RV305_wk0	0.0 (0/4)		0.0 (0/3)		0.0 (0/3)			
IgG2	C	V1V2	gp70-TV1.21 V1V2	RV305_wk2	0.0 (0/4)		33.3 (1/3)	107	0.0 (0/3)			
IgG2	C	V1V2	gp70-TV1.21 V1V2	RV305_wk26	0.0 (0/4)		0.0 (0/3)		0.0 (0/3)			
IgG2	C	V1V2	gp70-TV1.21 V1V2	RV305_wk48	0.0 (0/4)		0.0 (0/3)		0.0 (0/3)			
IgG2	C	V1V2	gp70-TV1.21 V1V2	RV305_wk72	0.0 (0/4)		0.0 (0/3)		0.0 (0/3)			

S11 Table continued

					Group 1: Combination		Group 2: AIDSVAX B/E		Group 3: ALVAC-HIV		RV305_Placebo Group	
Isotype	Clade	Env Region	Antigen	Study Week	Response Rate (Responders/ Total)	Median MFI Responders	Response Rate (Responders/ Total)	Median MFI Responders	Response Rate (Responders/ Total)	Median MFI Responders	Response Rate (Responders/ Total)	Median MFI Responders
IgG2	CRF01_AE	V1V2	AE.A244 V1V2 tags	RV144_wk26	12.5 (2/16)	133	6.7 (1/15)	129	0.0 (0/17)		0.0 (0/9)	
IgG2	CRF01_AE	V1V2	AE.A244 V1V2 tags	RV305_wk0	0.0 (0/20)		0.0 (0/18)		0.0 (0/19)		0.0 (0/12)	
IgG2	CRF01_AE	V1V2	AE.A244 V1V2 tags	RV305_wk2	5.0 (1/20)	532	16.7 (3/18)	111	0.0 (0/19)		0.0 (0/12)	
IgG2	CRF01_AE	V1V2	AE.A244 V1V2 tags	RV305_wk24	0.0 (0/20)		0.0 (0/18)		0.0 (0/19)		0.0 (0/12)	
IgG2	CRF01_AE	V1V2	AE.A244 V1V2 tags	RV305_wk26	10.0 (2/20)	148	0.0 (0/18)		0.0 (0/19)		0.0 (0/13)	
IgG2	CRF01_AE	V1V2	AE.A244 V1V2 tags	RV305_wk48	0.0 (0/20)		0.0 (0/18)		0.0 (0/19)		0.0 (0/12)	
IgG2	CRF01_AE	V1V2	AE.A244 V1V2 tags	RV305_wk72	0.0 (0/20)		0.0 (0/18)		0.0 (0/18)		0.0 (0/12)	
IgG2	CRF01_AE	V1V2	gp70-C2101.c01_V1V2	RV144_wk26	6.3 (1/16)	321	0.0 (0/15)		5.9 (1/17)	104	0.0 (0/9)	
IgG2	CRF01_AE	V1V2	gp70-C2101.c01_V1V2	RV305_wk0	0.0 (0/20)		0.0 (0/18)		0.0 (0/19)		0.0 (0/12)	
IgG2	CRF01_AE	V1V2	gp70-C2101.c01_V1V2	RV305_wk2	5.0 (1/20)	561	5.6 (1/18)	1217	0.0 (0/19)		0.0 (0/12)	
IgG2	CRF01_AE	V1V2	gp70-C2101.c01_V1V2	RV305_wk24	0.0 (0/20)		0.0 (0/18)		0.0 (0/19)		0.0 (0/12)	
IgG2	CRF01_AE	V1V2	gp70-C2101.c01_V1V2	RV305_wk26	5.0 (1/20)	291	0.0 (0/18)		0.0 (0/19)		0.0 (0/13)	
IgG2	CRF01_AE	V1V2	gp70-C2101.c01_V1V2	RV305_wk48	0.0 (0/20)		0.0 (0/18)		0.0 (0/19)		0.0 (0/12)	
IgG2	CRF01_AE	V1V2	gp70-C2101.c01_V1V2	RV305_wk72	0.0 (0/20)		0.0 (0/18)		0.0 (0/18)		0.0 (0/12)	
IgG2	CRF01_AE	V1V2	gp70-CM244.ec1 V1V2	RV144_wk26	6.3 (1/16)	213	13.3 (2/15)	132	5.9 (1/17)	213	0.0 (0/9)	
IgG2	CRF01_AE	V1V2	gp70-CM244.ec1 V1V2	RV305_wk0	0.0 (0/20)		0.0 (0/18)		0.0 (0/19)		0.0 (0/12)	
IgG2	CRF01_AE	V1V2	gp70-CM244.ec1 V1V2	RV305_wk2	5.0 (1/20)	108	5.6 (1/18)	348	0.0 (0/19)		0.0 (0/12)	
IgG2	CRF01_AE	V1V2	gp70-CM244.ec1 V1V2	RV305_wk24	5.0 (1/20)	108	0.0 (0/18)		0.0 (0/19)		0.0 (0/12)	
IgG2	CRF01_AE	V1V2	gp70-CM244.ec1 V1V2	RV305_wk26	5.0 (1/20)	203	0.0 (0/18)		0.0 (0/19)		0.0 (0/13)	
IgG2	CRF01_AE	V1V2	gp70-CM244.ec1 V1V2	RV305_wk48	5.0 (1/20)	171	0.0 (0/18)		0.0 (0/19)		0.0 (0/12)	
IgG2	CRF01_AE	V1V2	gp70-CM244.ec1 V1V2	RV305_wk72	0.0 (0/20)		0.0 (0/18)		0.0 (0/18)		0.0 (0/12)	
IgG2	CRF07_BC	V1V2	gp70-BJOX002000.03.2 V1V2	RV144_wk26	0.0 (0/4)		0.0 (0/2)		0.0 (0/3)			
IgG2	CRF07_BC	V1V2	gp70-BJOX002000.03.2 V1V2	RV305_wk0	0.0 (0/4)		0.0 (0/3)		0.0 (0/3)			
IgG2	CRF07_BC	V1V2	gp70-BJOX002000.03.2 V1V2	RV305_wk2	0.0 (0/4)		0.0 (0/3)		0.0 (0/3)			
IgG2	CRF07_BC	V1V2	gp70-BJOX002000.03.2 V1V2	RV305_wk26	0.0 (0/4)		0.0 (0/3)		0.0 (0/2)			
IgG2	CRF07_BC	V1V2	gp70-BJOX002000.03.2 V1V2	RV305_wk48	0.0 (0/4)		0.0 (0/3)		0.0 (0/3)			
IgG2	CRF07_BC	V1V2	gp70-BJOX002000.03.2 V1V2	RV305_wk72	0.0 (0/4)		0.0 (0/3)		0.0 (0/3)			
IgG2	CRF01_AE	V2	AE.A244 V2 tags/293F	RV144_wk26	0.0 (0/16)		0.0 (0/15)		0.0 (0/17)		0.0 (0/9)	
IgG2	CRF01_AE	V2	AE.A244 V2 tags/293F	RV305_wk0	0.0 (0/20)		0.0 (0/18)		0.0 (0/19)		0.0 (0/12)	
IgG2	CRF01_AE	V2	AE.A244 V2 tags/293F	RV305_wk2	0.0 (0/20)		0.0 (0/18)		0.0 (0/19)		0.0 (0/12)	
IgG2	CRF01_AE	V2	AE.A244 V2 tags/293F	RV305_wk24	0.0 (0/20)		0.0 (0/18)		0.0 (0/19)		0.0 (0/12)	
IgG2	CRF01_AE	V2	AE.A244 V2 tags/293F	RV305_wk26	0.0 (0/20)		0.0 (0/18)		0.0 (0/19)		0.0 (0/13)	
IgG2	CRF01_AE	V2	AE.A244 V2 tags/293F	RV305_wk48	0.0 (0/20)		0.0 (0/18)		0.0 (0/19)		0.0 (0/12)	
IgG2	CRF01_AE	V2	AE.A244 V2 tags/293F	RV305_wk72	0.0 (0/20)		0.0 (0/18)		0.0 (0/18)		0.0 (0/12)	

S11 Table continued

					Group 1: Combination		Group 2: AIDSVAX B/E		Group 3: ALVAC-HIV		RV305_Placebo Group	
Isotype	Clade	Env Region	Antigen	Study Week	Response Rate (Responders/ Total)	Median MFI Responders	Response Rate (Responders/ Total)	Median MFI Responders	Response Rate (Responders/ Total)	Median MFI Responders	Response Rate (Responders/ Total)	Median MFI Responders
IgG2	B	V3	B.MN V3 gp70	RV144_wk26	0.0 (0/16)		0.0 (0/15)		0.0 (0/17)		0.0 (0/9)	
IgG2	B	V3	B.MN V3 gp70	RV305_wk0	0.0 (0/20)		0.0 (0/18)		0.0 (0/19)		0.0 (0/12)	
IgG2	B	V3	B.MN V3 gp70	RV305_wk2	0.0 (0/20)		5.6 (1/18)	167	0.0 (0/19)		0.0 (0/12)	
IgG2	B	V3	B.MN V3 gp70	RV305_wk24	0.0 (0/20)		0.0 (0/18)		0.0 (0/19)		0.0 (0/12)	
IgG2	B	V3	B.MN V3 gp70	RV305_wk26	0.0 (0/20)		0.0 (0/18)		0.0 (0/19)		0.0 (0/13)	
IgG2	B	V3	B.MN V3 gp70	RV305_wk48	0.0 (0/20)		0.0 (0/18)		0.0 (0/19)		0.0 (0/12)	
IgG2	B	V3	B.MN V3 gp70	RV305_wk72	0.0 (0/20)		0.0 (0/18)		0.0 (0/18)		0.0 (0/12)	
IgG2	N/A	CD4bs	RSC3_P363Npair	RV144_wk26	0.0 (0/1)		0.0 (0/3)		0.0 (0/5)			
IgG2	N/A	CD4bs	RSC3_P363Npair	RV305_wk0	0.0 (0/3)		0.0 (0/4)		0.0 (0/5)			
IgG2	N/A	CD4bs	RSC3_P363Npair	RV305_wk2	0.0 (0/3)		0.0 (0/4)		0.0 (0/5)			
IgG2	N/A	CD4bs	RSC3_P363Npair	RV305_wk26	0.0 (0/4)		0.0 (0/4)		0.0 (0/6)			
IgG2	N/A	CD4bs	RSC3_P363Npair	RV305_wk48	0.0 (0/3)		0.0 (0/4)		0.0 (0/5)			
IgG2	N/A	CD4bs	RSC3_P363Npair	RV305_wk72	0.0 (0/4)		0.0 (0/4)		0.0 (0/5)			
