# Supplementary material for: Viral vector delivered immunogen focuses HIV-1 antibody specificity and increases durability of the circulating antibody recall response
Source: PLoS Pathog. 2023 May 31;19(5):e1011359. doi: 10.1371/journal.ppat.1011359 (PMC10284421; doi:10.1371/journal.ppat.1011359)
Supplement: S12 Table — (PDF) [file ppat.1011359.s025.pdf]

**S12 Table. BAMA plasma binding IgG4 response rates and group median binding magnitudes (MFI) to gp120, gp140, V1V2, V3, CD4 inducible, CD4 binding site, and Gag HIV-1 regions.**

| | | | | | Group 1: Combination | | Group 2: AIDSVAX B/E | | Group 3: ALVAC-HIV | | RV305_Placebo Group | |
|---|---|---|---|---|---|---|---|---|---|---|---|---|
| Isotype | Clade | Env Region | Antigen | Study Week | Response Rate *(Responders/ Total)* | Median MFI *Responders* | Response Rate *(Responders/ Total)* | Median MFI *Responders* | Response Rate *(Responders/ Total)* | Median MFI *Responders* | Response Rate *(Responders/ Total)* | Median MFI *Responders* |
| IgG4 | A | gp120 | 51802_D11gp120.avi/293F | RV144_wk26 | 23.5 (4/17) | 783 | 13.3 (2/15) | 417 | 5.9 (1/17) | 827 | 0.0 (0/10) | |
| IgG4 | A | gp120 | 51802_D11gp120.avi/293F | RV305_wk0 | 20.0 (4/20) | 524 | 11.1 (2/18) | 475 | 5.3 (1/19) | 149 | 0.0 (0/13) | |
| IgG4 | A | gp120 | 51802_D11gp120.avi/293F | RV305_wk2 | 80.0 (16/20) | 1728 | 77.8 (14/18) | 1827 | 0.0 (0/19) | | 0.0 (0/13) | |
| IgG4 | A | gp120 | 51802_D11gp120.avi/293F | RV305_wk24 | 95.0 (19/20) | 1811 | 77.8 (14/18) | 1439 | 5.3 (1/19) | 1380 | 0.0 (0/13) | |
| IgG4 | A | gp120 | 51802_D11gp120.avi/293F | RV305_wk26 | 90.0 (18/20) | 2181 | 83.3 (15/18) | 3151 | 5.3 (1/19) | 136 | 0.0 (0/13) | |
| IgG4 | A | gp120 | 51802_D11gp120.avi/293F | RV305_wk48 | 85.0 (17/20) | 1936 | 83.3 (15/18) | 2546 | 15.8 (3/19) | 150 | 0.0 (0/13) | |
| IgG4 | A | gp120 | 51802_D11gp120.avi/293F | RV305_wk72 | 90.0 (18/20) | 1379 | 83.3 (15/18) | 1731 | 16.7 (3/18) | 150 | 0.0 (0/13) | |
| IgG4 | B | gp120 | B.6240_D11gp120/293F | RV144_wk26 | 17.6 (3/17) | 2269 | 13.3 (2/15) | 573 | 5.9 (1/17) | 1718 | 0.0 (0/10) | |
| IgG4 | B | gp120 | B.6240_D11gp120/293F | RV305_wk0 | 15.0 (3/20) | 1888 | 16.7 (3/18) | 505 | 0.0 (0/19) | | 0.0 (0/13) | |
| IgG4 | B | gp120 | B.6240_D11gp120/293F | RV305_wk2 | 80.0 (16/20) | 2452 | 83.3 (15/18) | 3240 | 0.0 (0/19) | | 0.0 (0/13) | |
| IgG4 | B | gp120 | B.6240_D11gp120/293F | RV305_wk24 | 95.0 (19/20) | 2891 | 77.8 (14/18) | 2604 | 5.3 (1/19) | 2745 | 0.0 (0/13) | |
| IgG4 | B | gp120 | B.6240_D11gp120/293F | RV305_wk26 | 90.0 (18/20) | 3023 | 83.3 (15/18) | 4531 | 0.0 (0/19) | | 0.0 (0/13) | |
| IgG4 | B | gp120 | B.6240_D11gp120/293F | RV305_wk48 | 85.0 (17/20) | 2929 | 83.3 (15/18) | 4272 | 5.3 (1/19) | 336 | 0.0 (0/13) | |
| IgG4 | B | gp120 | B.6240_D11gp120/293F | RV305_wk72 | 85.0 (17/20) | 2384 | 77.8 (14/18) | 3254 | 11.1 (2/18) | 1309 | 0.0 (0/13) | |
| IgG4 | B | gp120 | BORI_D11gp120.avi/293F | RV144_wk26 | 23.5 (4/17) | 1039 | 13.3 (2/15) | 212 | 5.9 (1/17) | 437 | 0.0 (0/10) | |
| IgG4 | B | gp120 | BORI_D11gp120.avi/293F | RV305_wk0 | 10.0 (2/20) | 570 | 11.1 (2/18) | 241 | 0.0 (0/19) | | 0.0 (0/13) | |
| IgG4 | B | gp120 | BORI_D11gp120.avi/293F | RV305_wk2 | 85.0 (17/20) | 1278 | 77.8 (14/18) | 1984 | 0.0 (0/19) | | 0.0 (0/13) | |
| IgG4 | B | gp120 | BORI_D11gp120.avi/293F | RV305_wk24 | 100 (20/20) | 1143 | 77.8 (14/18) | 910 | 5.3 (1/19) | 907 | 0.0 (0/13) | |
| IgG4 | B | gp120 | BORI_D11gp120.avi/293F | RV305_wk26 | 90.0 (18/20) | 1273 | 83.3 (15/18) | 2227 | 0.0 (0/19) | | 0.0 (0/13) | |
| IgG4 | B | gp120 | BORI_D11gp120.avi/293F | RV305_wk48 | 85.0 (17/20) | 1113 | 83.3 (15/18) | 1552 | 5.3 (1/19) | 123 | 0.0 (0/13) | |
| IgG4 | B | gp120 | BORI_D11gp120.avi/293F | RV305_wk72 | 95.0 (19/20) | 777 | 77.8 (14/18) | 1051 | 5.6 (1/18) | 652 | 0.0 (0/13) | |
| IgG4 | B | gp120 | MN gp120 gDneg/293F | RV144_wk26 | 29.4 (5/17) | 4069 | 13.3 (2/15) | 1307 | 5.9 (1/17) | 3184 | 0.0 (0/10) | |
| IgG4 | B | gp120 | MN gp120 gDneg/293F | RV305_wk0 | 15.0 (3/20) | 3062 | 11.1 (2/18) | 1264 | 10.5 (2/19) | 362 | 0.0 (0/13) | |
| IgG4 | B | gp120 | MN gp120 gDneg/293F | RV305_wk2 | 85.0 (17/20) | 5631 | 88.9 (16/18) | 6572 | 10.5 (2/19) | 381 | 0.0 (0/13) | |
| IgG4 | B | gp120 | MN gp120 gDneg/293F | RV305_wk24 | 95.0 (19/20) | 5063 | 77.8 (14/18) | 4542 | 5.3 (1/19) | 7932 | 0.0 (0/13) | |
| IgG4 | B | gp120 | MN gp120 gDneg/293F | RV305_wk26 | 90.0 (18/20) | 7575 | 88.9 (16/18) | 8640 | 21.1 (4/19) | 384 | 0.0 (0/13) | |
| IgG4 | B | gp120 | MN gp120 gDneg/293F | RV305_wk48 | 85.0 (17/20) | 5158 | 83.3 (15/18) | 6983 | 15.8 (3/19) | 390 | 0.0 (0/13) | |
| IgG4 | B | gp120 | MN gp120 gDneg/293F | RV305_wk72 | 95.0 (19/20) | 3723 | 83.3 (15/18) | 4704 | 16.7 (3/18) | 362 | 0.0 (0/13) | |

S12 Table continued

| | | | | | Group 1: Combination | | Group 2: AIDSVAX B/E | | Group 3: ALVAC-HIV | | RV305_Placebo Group | |
|---|---|---|---|---|---|---|---|---|---|---|---|---|
| Isotype | Clade | Env Region | Antigen | Study Week | Response Rate (Responders/ Total) | Median MFI Responders | Response Rate (Responders/ Total) | Median MFI Responders | Response Rate (Responders/ Total) | Median MFI Responders | Response Rate (Responders/ Total) | Median MFI Responders |
| IgG4 | B | gp120 | TT31P.2792_D11gp120.avi/293F | RV144_wk26 | 23.5 (4/17) | 5589 | 13.3 (2/15) | 2947 | 5.9 (1/17) | 8235 | 0.0 (0/10) | |
| IgG4 | B | gp120 | TT31P.2792_D11gp120.avi/293F | RV305_wk0 | 20.0 (4/20) | 4075 | 11.1 (2/18) | 4431 | 5.3 (1/19) | 1517 | 0.0 (0/13) | |
| IgG4 | B | gp120 | TT31P.2792_D11gp120.avi/293F | RV305_wk2 | 80.0 (16/20) | 10428 | 83.3 (15/18) | 15579 | 0.0 (0/19) | | 0.0 (0/13) | |
| IgG4 | B | gp120 | TT31P.2792_D11gp120.avi/293F | RV305_wk24 | 95.0 (19/20) | 12267 | 77.8 (14/18) | 10033 | 5.3 (1/19) | 14173 | 0.0 (0/13) | |
| IgG4 | B | gp120 | TT31P.2792_D11gp120.avi/293F | RV305_wk26 | 90.0 (18/20) | 14626 | 83.3 (15/18) | 18984 | 0.0 (0/19) | | 0.0 (0/13) | |
| IgG4 | B | gp120 | TT31P.2792_D11gp120.avi/293F | RV305_wk48 | 85.0 (17/20) | 12962 | 83.3 (15/18) | 17079 | 5.3 (1/19) | 1591 | 0.0 (0/13) | |
| IgG4 | B | gp120 | TT31P.2792_D11gp120.avi/293F | RV305_wk72 | 90.0 (18/20) | 9629 | 77.8 (14/18) | 12814 | 11.1 (2/18) | 6327 | 0.0 (0/13) | |
| IgG4 | C | gp120 | 1086C_D7gp120.avi/293F | RV144_wk26 | 23.5 (4/17) | 6601 | 13.3 (2/15) | 2421 | 5.9 (1/17) | 8513 | 0.0 (0/10) | |
| IgG4 | C | gp120 | 1086C_D7gp120.avi/293F | RV305_wk0 | 20.0 (4/20) | 4656 | 16.7 (3/18) | 2024 | 0.0 (0/19) | | 0.0 (0/13) | |
| IgG4 | C | gp120 | 1086C_D7gp120.avi/293F | RV305_wk2 | 80.0 (16/20) | 10897 | 83.3 (15/18) | 15804 | 0.0 (0/19) | | 0.0 (0/13) | |
| IgG4 | C | gp120 | 1086C_D7gp120.avi/293F | RV305_wk24 | 90.0 (18/20) | 12319 | 77.8 (14/18) | 10802 | 5.3 (1/19) | 14760 | 0.0 (0/13) | |
| IgG4 | C | gp120 | 1086C_D7gp120.avi/293F | RV305_wk26 | 90.0 (18/20) | 14866 | 88.9 (16/18) | 18450 | 10.5 (2/19) | 1511 | 0.0 (0/13) | |
| IgG4 | C | gp120 | 1086C_D7gp120.avi/293F | RV305_wk48 | 85.0 (17/20) | 11369 | 83.3 (15/18) | 19121 | 15.8 (3/19) | 1086 | 0.0 (0/13) | |
| IgG4 | C | gp120 | 1086C_D7gp120.avi/293F | RV305_wk72 | 90.0 (18/20) | 9146 | 83.3 (15/18) | 13712 | 16.7 (3/18) | 1398 | 0.0 (0/13) | |
| IgG4 | C | gp120 | 96ZM651.D11gp120.avi | RV144_wk26 | 23.5 (4/17) | 1747 | 13.3 (2/15) | 665 | 5.9 (1/17) | 1729 | 0.0 (0/10) | |
| IgG4 | C | gp120 | 96ZM651.D11gp120.avi | RV305_wk0 | 15.0 (3/20) | 1507 | 11.1 (2/18) | 565 | 0.0 (0/19) | | 0.0 (0/13) | |
| IgG4 | C | gp120 | 96ZM651.D11gp120.avi | RV305_wk2 | 80.0 (16/20) | 2842 | 83.3 (15/18) | 3157 | 0.0 (0/19) | | 0.0 (0/13) | |
| IgG4 | C | gp120 | 96ZM651.D11gp120.avi | RV305_wk24 | 90.0 (18/20) | 3332 | 77.8 (14/18) | 2496 | 5.3 (1/19) | 2592 | 0.0 (0/13) | |
| IgG4 | C | gp120 | 96ZM651.D11gp120.avi | RV305_wk26 | 90.0 (18/20) | 3371 | 83.3 (15/18) | 5700 | 5.3 (1/19) | 1709 | 0.0 (0/13) | |
| IgG4 | C | gp120 | 96ZM651.D11gp120.avi | RV305_wk48 | 85.0 (17/20) | 2648 | 83.3 (15/18) | 4824 | 10.5 (2/19) | 393 | 0.0 (0/13) | |
| IgG4 | C | gp120 | 96ZM651.D11gp120.avi | RV305_wk72 | 85.0 (17/20) | 2298 | 77.8 (14/18) | 3386 | 5.6 (1/18) | 2050 | 0.0 (0/13) | |
| IgG4 | C | gp120 | TV1c8_D11gp120.avi/293F | RV144_wk26 | 29.4 (5/17) | 1376 | 13.3 (2/15) | 712 | 5.9 (1/17) | 1580 | 0.0 (0/10) | |
| IgG4 | C | gp120 | TV1c8_D11gp120.avi/293F | RV305_wk0 | 20.0 (4/20) | 1049 | 16.7 (3/18) | 605 | 5.3 (1/19) | 258 | 0.0 (0/13) | |
| IgG4 | C | gp120 | TV1c8_D11gp120.avi/293F | RV305_wk2 | 85.0 (17/20) | 2893 | 83.3 (15/18) | 3878 | 5.3 (1/19) | 276 | 0.0 (0/13) | |
| IgG4 | C | gp120 | TV1c8_D11gp120.avi/293F | RV305_wk24 | 95.0 (19/20) | 3164 | 83.3 (15/18) | 2516 | 10.5 (2/19) | 1455 | 0.0 (0/13) | |
| IgG4 | C | gp120 | TV1c8_D11gp120.avi/293F | RV305_wk26 | 90.0 (18/20) | 4041 | 88.9 (16/18) | 5565 | 15.8 (3/19) | 225 | 0.0 (0/13) | |
| IgG4 | C | gp120 | TV1c8_D11gp120.avi/293F | RV305_wk48 | 85.0 (17/20) | 3242 | 83.3 (15/18) | 4767 | 15.8 (3/19) | 258 | 0.0 (0/13) | |
| IgG4 | C | gp120 | TV1c8_D11gp120.avi/293F | RV305_wk72 | 90.0 (18/20) | 2367 | 83.3 (15/18) | 3348 | 16.7 (3/18) | 279 | 0.0 (0/13) | |
| IgG4 | CRF01_AE | gp120 | 254008_D11gp120.avi/293F | RV144_wk26 | 17.6 (3/17) | 4133 | 13.3 (2/15) | 1716 | 5.9 (1/17) | 3766 | 0.0 (0/10) | |
| IgG4 | CRF01_AE | gp120 | 254008_D11gp120.avi/293F | RV305_wk0 | 20.0 (4/20) | 2201 | 11.1 (2/18) | 2711 | 5.3 (1/19) | 832 | 0.0 (0/13) | |
| IgG4 | CRF01_AE | gp120 | 254008_D11gp120.avi/293F | RV305_wk2 | 80.0 (16/20) | 5404 | 83.3 (15/18) | 8021 | 0.0 (0/19) | | 0.0 (0/13) | |
| IgG4 | CRF01_AE | gp120 | 254008_D11gp120.avi/293F | RV305_wk24 | 95.0 (19/20) | 6849 | 77.8 (14/18) | 6188 | 10.5 (2/19) | 3616 | 0.0 (0/13) | |
| IgG4 | CRF01_AE | gp120 | 254008_D11gp120.avi/293F | RV305_wk26 | 90.0 (18/20) | 8230 | 83.3 (15/18) | 11289 | 10.5 (2/19) | 811 | 0.0 (0/13) | |
| IgG4 | CRF01_AE | gp120 | 254008_D11gp120.avi/293F | RV305_wk48 | 85.0 (17/20) | 7452 | 83.3 (15/18) | 9344 | 10.5 (2/19) | 837 | 0.0 (0/13) | |
| IgG4 | CRF01_AE | gp120 | 254008_D11gp120.avi/293F | RV305_wk72 | 85.0 (17/20) | 6131 | 77.8 (14/18) | 7892 | 5.6 (1/18) | 5156 | 0.0 (0/13) | |

| | | | | | Group 1: Combination | | Group 2: AIDSVAX B/E | | Group 3: ALVAC-HIV | | RV305_Placebo Group | |
|---|---|---|---|---|---|---|---|---|---|---|---|---|
| Isotype | Clade | Env Region | Antigen | Study Week | Response Rate (Responders/ Total) | Median MFI Responders | Response Rate (Responders/ Total) | Median MFI Responders | Response Rate (Responders/ Total) | Median MFI Responders | Response Rate (Responders/ Total) | Median MFI Responders |
| IgG4 | CRF01_AE | gp120 | 92TH023 gp120 gDneg 293F mon | RV144_wk26 | 29.4 (5/17) | 1226 | 20.0 (3/15) | 176 | 5.9 (1/17) | 778 | 0.0 (0/10) | |
| IgG4 | CRF01_AE | gp120 | 92TH023 gp120 gDneg 293F mon | RV305_wk0 | 20.0 (4/20) | 507 | 11.1 (2/18) | 369 | 5.3 (1/19) | 102 | 0.0 (0/13) | |
| IgG4 | CRF01_AE | gp120 | 92TH023 gp120 gDneg 293F mon | RV305_wk2 | 85.0 (17/20) | 2454 | 88.9 (16/18) | 3058 | 5.3 (1/19) | 367 | 0.0 (0/13) | |
| IgG4 | CRF01_AE | gp120 | 92TH023 gp120 gDneg 293F mon | RV305_wk24 | 100 (20/20) | 1682 | 83.3 (15/18) | 1210 | 5.3 (1/19) | 1543 | 0.0 (0/13) | |
| IgG4 | CRF01_AE | gp120 | 92TH023 gp120 gDneg 293F mon | RV305_wk26 | 90.0 (18/20) | 2172 | 88.9 (16/18) | 3309 | 21.1 (4/19) | 245 | 0.0 (0/13) | |
| IgG4 | CRF01_AE | gp120 | 92TH023 gp120 gDneg 293F mon | RV305_wk48 | 85.0 (17/20) | 1589 | 83.3 (15/18) | 1899 | 15.8 (3/19) | 235 | 0.0 (0/13) | |
| IgG4 | CRF01_AE | gp120 | 92TH023 gp120 gDneg 293F mon | RV305_wk72 | 95.0 (19/20) | 1050 | 83.3 (15/18) | 1189 | 11.1 (2/18) | 638 | 0.0 (0/13) | |
| IgG4 | CRF01_AE | gp120 | A244 D11gp120_avi | RV144_wk26 | 23.5 (4/17) | 2515 | 13.3 (2/15) | 929 | 5.9 (1/17) | 2815 | 0.0 (0/10) | |
| IgG4 | CRF01_AE | gp120 | A244 D11gp120_avi | RV305_wk0 | 20.0 (4/20) | 1564 | 11.1 (2/18) | 1334 | 5.3 (1/19) | 531 | 0.0 (0/13) | |
| IgG4 | CRF01_AE | gp120 | A244 D11gp120_avi | RV305_wk2 | 85.0 (17/20) | 3319 | 88.9 (16/18) | 5111 | 5.3 (1/19) | 413 | 0.0 (0/13) | |
| IgG4 | CRF01_AE | gp120 | A244 D11gp120_avi | RV305_wk24 | 95.0 (19/20) | 3842 | 77.8 (14/18) | 2933 | 10.5 (2/19) | 2335 | 0.0 (0/13) | |
| IgG4 | CRF01_AE | gp120 | A244 D11gp120_avi | RV305_wk26 | 90.0 (18/20) | 3827 | 83.3 (15/18) | 6850 | 10.5 (2/19) | 1478 | 0.0 (0/13) | |
| IgG4 | CRF01_AE | gp120 | A244 D11gp120_avi | RV305_wk48 | 85.0 (17/20) | 3495 | 83.3 (15/18) | 4956 | 15.8 (3/19) | 445 | 0.0 (0/13) | |
| IgG4 | CRF01_AE | gp120 | A244 D11gp120_avi | RV305_wk72 | 90.0 (18/20) | 2952 | 83.3 (15/18) | 3114 | 11.1 (2/18) | 2065 | 0.0 (0/13) | |
| IgG4 | CRF01_AE | gp120 | CM235 gp120 | RV144_wk26 | 23.5 (4/17) | 1962 | 13.3 (2/15) | 643 | 5.9 (1/17) | 1560 | 0.0 (0/10) | |
| IgG4 | CRF01_AE | gp120 | CM235 gp120 | RV305_wk0 | 20.0 (4/20) | 930 | 11.1 (2/18) | 806 | 10.5 (2/19) | 427 | 7.7 (1/13) | 212 |
| IgG4 | CRF01_AE | gp120 | CM235 gp120 | RV305_wk2 | 85.0 (17/20) | 3758 | 83.3 (15/18) | 4116 | 5.3 (1/19) | 664 | 0.0 (0/13) | |
| IgG4 | CRF01_AE | gp120 | CM235 gp120 | RV305_wk24 | 95.0 (19/20) | 3528 | 77.8 (14/18) | 2617 | 21.1 (4/19) | 426 | 7.7 (1/13) | 241 |
| IgG4 | CRF01_AE | gp120 | CM235 gp120 | RV305_wk26 | 90.0 (18/20) | 4382 | 83.3 (15/18) | 5043 | 26.3 (5/19) | 320 | 7.7 (1/13) | 240 |
| IgG4 | CRF01_AE | gp120 | CM235 gp120 | RV305_wk48 | 85.0 (17/20) | 3145 | 83.3 (15/18) | 2965 | 31.6 (6/19) | 324 | 7.7 (1/13) | 267 |
| IgG4 | CRF01_AE | gp120 | CM235 gp120 | RV305_wk72 | 90.0 (18/20) | 2482 | 83.3 (15/18) | 2390 | 27.8 (5/18) | 455 | 7.7 (1/13) | 346 |
| IgG4 | CRF07_BC | gp120 | BJOX002_D11gp120.avi/293F | RV144_wk26 | 23.5 (4/17) | 735 | 13.3 (2/15) | 258 | 5.9 (1/17) | 436 | 0.0 (0/10) | |
| IgG4 | CRF07_BC | gp120 | BJOX002_D11gp120.avi/293F | RV305_wk0 | 15.0 (3/20) | 463 | 11.1 (2/18) | 216 | 0.0 (0/19) | | 0.0 (0/13) | |
| IgG4 | CRF07_BC | gp120 | BJOX002_D11gp120.avi/293F | RV305_wk2 | 85.0 (17/20) | 1358 | 83.3 (15/18) | 1895 | 10.5 (2/19) | 135 | 0.0 (0/13) | |
| IgG4 | CRF07_BC | gp120 | BJOX002_D11gp120.avi/293F | RV305_wk24 | 95.0 (19/20) | 1421 | 77.8 (14/18) | 997 | 5.3 (1/19) | 927 | 0.0 (0/13) | |
| IgG4 | CRF07_BC | gp120 | BJOX002_D11gp120.avi/293F | RV305_wk26 | 90.0 (18/20) | 1542 | 83.3 (15/18) | 2813 | 21.1 (4/19) | 106 | 0.0 (0/13) | |
| IgG4 | CRF07_BC | gp120 | BJOX002_D11gp120.avi/293F | RV305_wk48 | 85.0 (17/20) | 1212 | 83.3 (15/18) | 1691 | 21.1 (4/19) | 130 | 0.0 (0/13) | |
| IgG4 | CRF07_BC | gp120 | BJOX002_D11gp120.avi/293F | RV305_wk72 | 90.0 (18/20) | 872 | 77.8 (14/18) | 1116 | 16.7 (3/18) | 170 | 0.0 (0/13) | |
| IgG4 | CRF07_BC | gp120 | CNE20_D11gp120.avi/293F | RV144_wk26 | 23.5 (4/17) | 2326 | 13.3 (2/15) | 847 | 5.9 (1/17) | 2403 | 0.0 (0/10) | |
| IgG4 | CRF07_BC | gp120 | CNE20_D11gp120.avi/293F | RV305_wk0 | 15.0 (3/20) | 2537 | 11.1 (2/18) | 783 | 10.5 (2/19) | 453 | 0.0 (0/13) | |
| IgG4 | CRF07_BC | gp120 | CNE20_D11gp120.avi/293F | RV305_wk2 | 80.0 (16/20) | 5057 | 83.3 (15/18) | 6037 | 5.3 (1/19) | 463 | 0.0 (0/13) | |
| IgG4 | CRF07_BC | gp120 | CNE20_D11gp120.avi/293F | RV305_wk24 | 95.0 (19/20) | 4850 | 77.8 (14/18) | 3850 | 10.5 (2/19) | 2309 | 0.0 (0/13) | |
| IgG4 | CRF07_BC | gp120 | CNE20_D11gp120.avi/293F | RV305_wk26 | 90.0 (18/20) | 6197 | 83.3 (15/18) | 8937 | 15.8 (3/19) | 404 | 0.0 (0/13) | |
| IgG4 | CRF07_BC | gp120 | CNE20_D11gp120.avi/293F | RV305_wk48 | 85.0 (17/20) | 4836 | 83.3 (15/18) | 6635 | 21.1 (4/19) | 480 | 0.0 (0/13) | |
| IgG4 | CRF07_BC | gp120 | CNE20_D11gp120.avi/293F | RV305_wk72 | 90.0 (18/20) | 3725 | 77.8 (14/18) | 4356 | 16.7 (3/18) | 599 | 0.0 (0/13) | |

| | | | | | Group 1: Combination | | Group 2: AIDSVAX B/E | | Group 3: ALVAC-HIV | | RV305_Placebo Group | |
|---|---|---|---|---|---|---|---|---|---|---|---|---|
| Isotype | Clade | Env Region | Antigen | Study Week | Response Rate (Responders/ Total) | Median MFI Responders | Response Rate (Responders/ Total) | Median MFI Responders | Response Rate (Responders/ Total) | Median MFI Responders | Response Rate (Responders/ Total) | Median MFI Responders |
| IgG4 | Consensus | gp120 | Con 6 gp120/B | RV144_wk26 | 35.3 (6/17) | 1549 | 13.3 (2/15) | 553 | 11.8 (2/17) | 1018 | 0.0 (0/10) | |
| IgG4 | Consensus | gp120 | Con 6 gp120/B | RV305_wk0 | 20.0 (4/20) | 967 | 16.7 (3/18) | 711 | 10.5 (2/19) | 166 | 0.0 (0/13) | |
| IgG4 | Consensus | gp120 | Con 6 gp120/B | RV305_wk2 | 85.0 (17/20) | 5548 | 88.9 (16/18) | 6699 | 15.8 (3/19) | 168 | 0.0 (0/13) | |
| IgG4 | Consensus | gp120 | Con 6 gp120/B | RV305_wk24 | 100 (20/20) | 4239 | 83.3 (15/18) | 2829 | 26.3 (5/19) | 161 | 0.0 (0/13) | |
| IgG4 | Consensus | gp120 | Con 6 gp120/B | RV305_wk26 | 90.0 (18/20) | 6005 | 88.9 (16/18) | 7846 | 36.8 (7/19) | 218 | 0.0 (0/13) | |
| IgG4 | Consensus | gp120 | Con 6 gp120/B | RV305_wk48 | 85.0 (17/20) | 4529 | 83.3 (15/18) | 5767 | 26.3 (5/19) | 210 | 0.0 (0/13) | |
| IgG4 | Consensus | gp120 | Con 6 gp120/B | RV305_wk72 | 95.0 (19/20) | 2815 | 83.3 (15/18) | 3466 | 27.8 (5/18) | 205 | 0.0 (0/13) | |
| IgG4 | N/A | gp120 | YU2 gp120 old core | RV144_wk26 | 0.0 (0/17) | | 0.0 (0/15) | | 0.0 (0/17) | | 0.0 (0/10) | |
| IgG4 | N/A | gp120 | YU2 gp120 old core | RV305_wk0 | 0.0 (0/20) | | 0.0 (0/18) | | 0.0 (0/19) | | 0.0 (0/13) | |
| IgG4 | N/A | gp120 | YU2 gp120 old core | RV305_wk2 | 0.0 (0/20) | | 0.0 (0/18) | | 0.0 (0/19) | | 0.0 (0/13) | |
| IgG4 | N/A | gp120 | YU2 gp120 old core | RV305_wk24 | 0.0 (0/20) | | 0.0 (0/18) | | 0.0 (0/19) | | 0.0 (0/13) | |
| IgG4 | N/A | gp120 | YU2 gp120 old core | RV305_wk26 | 0.0 (0/20) | | 0.0 (0/18) | | 0.0 (0/19) | | 0.0 (0/13) | |
| IgG4 | N/A | gp120 | YU2 gp120 old core | RV305_wk48 | 0.0 (0/20) | | 0.0 (0/18) | | 0.0 (0/19) | | 0.0 (0/13) | |
| IgG4 | N/A | gp120 | YU2 gp120 old core | RV305_wk72 | 0.0 (0/20) | | 0.0 (0/18) | | 0.0 (0/18) | | 0.0 (0/13) | |
| IgG4 | A | gp140 | 9004S.gp140C.avi | RV144_wk26 | 23.5 (4/17) | 418 | 6.7 (1/15) | 131 | 5.9 (1/17) | 320 | 0.0 (0/10) | |
| IgG4 | A | gp140 | 9004S.gp140C.avi | RV305_wk0 | 10.0 (2/20) | 361 | 11.1 (2/18) | 118 | 0.0 (0/19) | | 0.0 (0/13) | |
| IgG4 | A | gp140 | 9004S.gp140C.avi | RV305_wk2 | 80.0 (16/20) | 915 | 77.8 (14/18) | 1068 | 0.0 (0/19) | | 0.0 (0/13) | |
| IgG4 | A | gp140 | 9004S.gp140C.avi | RV305_wk24 | 90.0 (18/20) | 826 | 77.8 (14/18) | 541 | 5.3 (1/19) | 745 | 0.0 (0/13) | |
| IgG4 | A | gp140 | 9004S.gp140C.avi | RV305_wk26 | 90.0 (18/20) | 770 | 77.8 (14/18) | 1271 | 0.0 (0/19) | | 0.0 (0/13) | |
| IgG4 | A | gp140 | 9004S.gp140C.avi | RV305_wk48 | 85.0 (17/20) | 567 | 83.3 (15/18) | 779 | 0.0 (0/19) | | 0.0 (0/13) | |
| IgG4 | A | gp140 | 9004S.gp140C.avi | RV305_wk72 | 80.0 (16/20) | 510 | 66.7 (12/18) | 720 | 5.6 (1/18) | 461 | 0.0 (0/13) | |
| IgG4 | B | gp140 | RHPA4259_C7.gp140C.avi | RV144_wk26 | 35.3 (6/17) | 1278 | 13.3 (2/15) | 378 | 5.9 (1/17) | 1223 | 0.0 (0/10) | |
| IgG4 | B | gp140 | RHPA4259_C7.gp140C.avi | RV305_wk0 | 20.0 (4/20) | 585 | 11.1 (2/18) | 470 | 5.3 (1/19) | 105 | 0.0 (0/13) | |
| IgG4 | B | gp140 | RHPA4259_C7.gp140C.avi | RV305_wk2 | 85.0 (17/20) | 2182 | 83.3 (15/18) | 3851 | 5.3 (1/19) | 109 | 0.0 (0/13) | |
| IgG4 | B | gp140 | RHPA4259_C7.gp140C.avi | RV305_wk24 | 100 (20/20) | 1906 | 77.8 (14/18) | 1408 | 10.5 (2/19) | 1436 | 0.0 (0/13) | |
| IgG4 | B | gp140 | RHPA4259_C7.gp140C.avi | RV305_wk26 | 90.0 (18/20) | 2323 | 83.3 (15/18) | 3928 | 10.5 (2/19) | 108 | 0.0 (0/13) | |
| IgG4 | B | gp140 | RHPA4259_C7.gp140C.avi | RV305_wk48 | 85.0 (17/20) | 1999 | 83.3 (15/18) | 2886 | 21.1 (4/19) | 119 | 0.0 (0/13) | |
| IgG4 | B | gp140 | RHPA4259_C7.gp140C.avi | RV305_wk72 | 95.0 (19/20) | 1290 | 83.3 (15/18) | 1718 | 11.1 (2/18) | 1002 | 0.0 (0/13) | |
| IgG4 | B | gp140 | SC42261_gp140.avi/293F | RV144_wk26 | 29.4 (5/17) | 4585 | 13.3 (2/15) | 496 | 5.9 (1/17) | 1332 | 0.0 (0/10) | |
| IgG4 | B | gp140 | SC42261_gp140.avi/293F | RV305_wk0 | 15.0 (3/20) | 1488 | 11.1 (2/18) | 607 | 0.0 (0/19) | | 0.0 (0/13) | |
| IgG4 | B | gp140 | SC42261_gp140.avi/293F | RV305_wk2 | 85.0 (17/20) | 5082 | 77.8 (14/18) | 7619 | 0.0 (0/19) | | 0.0 (0/13) | |
| IgG4 | B | gp140 | SC42261_gp140.avi/293F | RV305_wk24 | 100 (20/20) | 4052 | 77.8 (14/18) | 2899 | 5.3 (1/19) | 4823 | 0.0 (0/13) | |
| IgG4 | B | gp140 | SC42261_gp140.avi/293F | RV305_wk26 | 90.0 (18/20) | 4998 | 83.3 (15/18) | 7917 | 5.3 (1/19) | 189 | 0.0 (0/13) | |
| IgG4 | B | gp140 | SC42261_gp140.avi/293F | RV305_wk48 | 90.0 (18/20) | 3237 | 83.3 (15/18) | 4659 | 10.5 (2/19) | 206 | 0.0 (0/13) | |
| IgG4 | B | gp140 | SC42261_gp140.avi/293F | RV305_wk72 | 95.0 (19/20) | 2280 | 83.3 (15/18) | 2811 | 5.6 (1/18) | 2094 | 0.0 (0/13) | |

| | | | | | Group 1: Combination | | Group 2: AIDSVAX B/E | | Group 3: ALVAC-HIV | | RV305_Placebo Group | |
|---|---|---|---|---|---|---|---|---|---|---|---|---|
| Isotype | Clade | Env Region | Antigen | Study Week | Response Rate (Responders/ Total) | Median MFI Responders | Response Rate (Responders/ Total) | Median MFI Responders | Response Rate (Responders/ Total) | Median MFI Responders | Response Rate (Responders/ Total) | Median MFI Responders |
| IgG4 | B | gp140 | WITO4160.gp140C.avi | RV144_wk26 | 29.4 (5/17) | 1524 | 13.3 (2/15) | 149 | 5.9 (1/17) | 657 | 0.0 (0/10) | |
| IgG4 | B | gp140 | WITO4160.gp140C.avi | RV305_wk0 | 15.0 (3/20) | 673 | 11.1 (2/18) | 272 | 0.0 (0/19) | | 0.0 (0/13) | |
| IgG4 | B | gp140 | WITO4160.gp140C.avi | RV305_wk2 | 85.0 (17/20) | 2037 | 77.8 (14/18) | 3307 | 0.0 (0/19) | | 0.0 (0/13) | |
| IgG4 | B | gp140 | WITO4160.gp140C.avi | RV305_wk24 | 95.0 (19/20) | 1470 | 77.8 (14/18) | 1093 | 5.3 (1/19) | 2766 | 0.0 (0/13) | |
| IgG4 | B | gp140 | WITO4160.gp140C.avi | RV305_wk26 | 90.0 (18/20) | 1608 | 83.3 (15/18) | 2759 | 0.0 (0/19) | | 0.0 (0/13) | |
| IgG4 | B | gp140 | WITO4160.gp140C.avi | RV305_wk48 | 85.0 (17/20) | 1140 | 83.3 (15/18) | 1771 | 0.0 (0/19) | | 0.0 (0/13) | |
| IgG4 | B | gp140 | WITO4160.gp140C.avi | RV305_wk72 | 90.0 (18/20) | 813 | 72.2 (13/18) | 1031 | 5.6 (1/18) | 1135 | 0.0 (0/13) | |
| IgG4 | C | gp140 | 1086C gp140C_avi | RV144_wk26 | 29.4 (5/17) | 7532 | 13.3 (2/15) | 1304 | 5.9 (1/17) | 5640 | 0.0 (0/10) | |
| IgG4 | C | gp140 | 1086C gp140C_avi | RV305_wk0 | 15.0 (3/20) | 5034 | 16.7 (3/18) | 1023 | 5.3 (1/19) | 518 | 0.0 (0/13) | |
| IgG4 | C | gp140 | 1086C gp140C_avi | RV305_wk2 | 85.0 (17/20) | 9497 | 83.3 (15/18) | 15643 | 5.3 (1/19) | 560 | 0.0 (0/13) | |
| IgG4 | C | gp140 | 1086C gp140C_avi | RV305_wk24 | 95.0 (19/20) | 9349 | 83.3 (15/18) | 7451 | 10.5 (2/19) | 6288 | 0.0 (0/13) | |
| IgG4 | C | gp140 | 1086C gp140C_avi | RV305_wk26 | 90.0 (18/20) | 10584 | 88.9 (16/18) | 17401 | 21.1 (4/19) | 518 | 0.0 (0/13) | |
| IgG4 | C | gp140 | 1086C gp140C_avi | RV305_wk48 | 85.0 (17/20) | 9480 | 88.9 (16/18) | 12813 | 26.3 (5/19) | 648 | 0.0 (0/13) | |
| IgG4 | C | gp140 | 1086C gp140C_avi | RV305_wk72 | 90.0 (18/20) | 6506 | 83.3 (15/18) | 8167 | 16.7 (3/18) | 669 | 0.0 (0/13) | |
| IgG4 | C | gp140 | BF1266_gp140C.avi/293F | RV144_wk26 | 29.4 (5/17) | 1127 | 13.3 (2/15) | 166 | 5.9 (1/17) | 615 | 0.0 (0/10) | |
| IgG4 | C | gp140 | BF1266_gp140C.avi/293F | RV305_wk0 | 15.0 (3/20) | 660 | 16.7 (3/18) | 124 | 0.0 (0/19) | | 0.0 (0/13) | |
| IgG4 | C | gp140 | BF1266_gp140C.avi/293F | RV305_wk2 | 85.0 (17/20) | 1922 | 83.3 (15/18) | 3542 | 0.0 (0/19) | | 0.0 (0/13) | |
| IgG4 | C | gp140 | BF1266_gp140C.avi/293F | RV305_wk24 | 100 (20/20) | 1552 | 83.3 (15/18) | 1093 | 5.3 (1/19) | 2155 | 0.0 (0/13) | |
| IgG4 | C | gp140 | BF1266_gp140C.avi/293F | RV305_wk26 | 90.0 (18/20) | 1735 | 83.3 (15/18) | 4021 | 10.5 (2/19) | 837 | 0.0 (0/13) | |
| IgG4 | C | gp140 | BF1266_gp140C.avi/293F | RV305_wk48 | 85.0 (17/20) | 1405 | 83.3 (15/18) | 2058 | 21.1 (4/19) | 109 | 0.0 (0/13) | |
| IgG4 | C | gp140 | BF1266_gp140C.avi/293F | RV305_wk72 | 95.0 (19/20) | 1011 | 83.3 (15/18) | 1228 | 5.6 (1/18) | 1043 | 0.0 (0/13) | |
| IgG4 | C | gp140 | C.CH505TF_gp140/293F | RV144_wk26 | 29.4 (5/17) | 1645 | 13.3 (2/15) | 224 | 5.9 (1/17) | 812 | 0.0 (0/10) | |
| IgG4 | C | gp140 | C.CH505TF_gp140/293F | RV305_wk0 | 10.0 (2/20) | 890 | 11.1 (2/18) | 291 | 0.0 (0/19) | | 0.0 (0/13) | |
| IgG4 | C | gp140 | C.CH505TF_gp140/293F | RV305_wk2 | 85.0 (17/20) | 2455 | 83.3 (15/18) | 3656 | 0.0 (0/19) | | 0.0 (0/13) | |
| IgG4 | C | gp140 | C.CH505TF_gp140/293F | RV305_wk24 | 90.0 (18/20) | 2269 | 83.3 (15/18) | 1272 | 5.3 (1/19) | 3191 | 0.0 (0/13) | |
| IgG4 | C | gp140 | C.CH505TF_gp140/293F | RV305_wk26 | 90.0 (18/20) | 2352 | 83.3 (15/18) | 4012 | 5.3 (1/19) | 372 | 0.0 (0/13) | |
| IgG4 | C | gp140 | C.CH505TF_gp140/293F | RV305_wk48 | 85.0 (17/20) | 1605 | 83.3 (15/18) | 2383 | 5.3 (1/19) | 116 | 0.0 (0/13) | |
| IgG4 | C | gp140 | C.CH505TF_gp140/293F | RV305_wk72 | 90.0 (18/20) | 998 | 83.3 (15/18) | 1452 | 5.6 (1/18) | 1261 | 0.0 (0/13) | |
| IgG4 | Consensus | gp140 | Con S gp140 CFI | RV144_wk26 | 35.3 (6/17) | 3136 | 13.3 (2/15) | 321 | 5.9 (1/17) | 1531 | 0.0 (0/10) | |
| IgG4 | Consensus | gp140 | Con S gp140 CFI | RV305_wk0 | 15.0 (3/20) | 1596 | 11.1 (2/18) | 627 | 0.0 (0/19) | | 0.0 (0/13) | |
| IgG4 | Consensus | gp140 | Con S gp140 CFI | RV305_wk2 | 85.0 (17/20) | 5440 | 83.3 (15/18) | 7849 | 0.0 (0/19) | | 0.0 (0/13) | |
| IgG4 | Consensus | gp140 | Con S gp140 CFI | RV305_wk24 | 100 (20/20) | 3460 | 83.3 (15/18) | 1890 | 10.5 (2/19) | 2200 | 0.0 (0/13) | |
| IgG4 | Consensus | gp140 | Con S gp140 CFI | RV305_wk26 | 90.0 (18/20) | 4507 | 83.3 (15/18) | 7713 | 15.8 (3/19) | 198 | 0.0 (0/13) | |
| IgG4 | Consensus | gp140 | Con S gp140 CFI | RV305_wk48 | 85.0 (17/20) | 3073 | 83.3 (15/18) | 5039 | 26.3 (5/19) | 159 | 0.0 (0/13) | |
| IgG4 | Consensus | gp140 | Con S gp140 CFI | RV305_wk72 | 95.0 (19/20) | 1974 | 83.3 (15/18) | 2879 | 16.7 (3/18) | 154 | 0.0 (0/13) | |

S12 Table continued

| | | | | | Group 1: Combination | | Group 2: AIDSVAX B/E | | Group 3: ALVAC-HIV | | RV305_Placebo Group | |
|---|---|---|---|---|---|---|---|---|---|---|---|---|
| Isotype | Clade | Env Region | Antigen | Study Week | Response Rate (Responders/ Total) | Median MFI Responders | Response Rate (Responders/ Total) | Median MFI Responders | Response Rate (Responders/ Total) | Median MFI Responders | Response Rate (Responders/ Total) | Median MFI Responders |
| IgG4 | Consensus CRF01_AE | gp140 | AE.01.con_env03 gp140CF_avi | RV144_wk26 | 29.4 (5/17) | 1952 | 20.0 (3/15) | 168 | 5.9 (1/17) | 889 | 0.0 (0/10) | |
| IgG4 | Consensus CRF01_AE | gp140 | AE.01.con_env03 gp140CF_avi | RV305_wk0 | 10.0 (2/20) | 1075 | 11.1 (2/18) | 427 | 0.0 (0/19) | | 0.0 (0/13) | |
| IgG4 | Consensus CRF01_AE | gp140 | AE.01.con_env03 gp140CF_avi | RV305_wk2 | 85.0 (17/20) | 2394 | 83.3 (15/18) | 4347 | 5.3 (1/19) | 200 | 0.0 (0/13) | |
| IgG4 | Consensus CRF01_AE | gp140 | AE.01.con_env03 gp140CF_avi | RV305_wk24 | 100 (20/20) | 1605 | 83.3 (15/18) | 1679 | 5.3 (1/19) | 1974 | 0.0 (0/13) | |
| IgG4 | Consensus CRF01_AE | gp140 | AE.01.con_env03 gp140CF_avi | RV305_wk26 | 95.0 (19/20) | 2153 | 88.9 (16/18) | 4081 | 21.1 (4/19) | 149 | 0.0 (0/13) | |
| IgG4 | Consensus CRF01_AE | gp140 | AE.01.con_env03 gp140CF_avi | RV305_wk48 | 85.0 (17/20) | 1443 | 83.3 (15/18) | 2298 | 15.8 (3/19) | 118 | 0.0 (0/13) | |
| IgG4 | Consensus CRF01_AE | gp140 | AE.01.con_env03 gp140CF_avi | RV305_wk72 | 95.0 (19/20) | 1008 | 83.3 (15/18) | 1480 | 5.6 (1/18) | 1460 | 0.0 (0/13) | |
| IgG4 | A | V1V2 | gp70-191084_B7 V1V2 | RV144_wk26 | 11.8 (2/17) | 425 | 0.0 (0/15) | | 0.0 (0/17) | | 10.0 (1/10) | 239 |
| IgG4 | A | V1V2 | gp70-191084_B7 V1V2 | RV305_wk0 | 5.0 (1/20) | 1042 | 0.0 (0/18) | | 0.0 (0/19) | | 0.0 (0/13) | |
| IgG4 | A | V1V2 | gp70-191084_B7 V1V2 | RV305_wk2 | 45.0 (9/20) | 507 | 38.9 (7/18) | 305 | 0.0 (0/19) | | 0.0 (0/13) | |
| IgG4 | A | V1V2 | gp70-191084_B7 V1V2 | RV305_wk24 | 25.0 (5/20) | 654 | 22.2 (4/18) | 256 | 0.0 (0/19) | | 0.0 (0/13) | |
| IgG4 | A | V1V2 | gp70-191084_B7 V1V2 | RV305_wk26 | 45.0 (9/20) | 681 | 38.9 (7/18) | 311 | 0.0 (0/19) | | 0.0 (0/13) | |
| IgG4 | A | V1V2 | gp70-191084_B7 V1V2 | RV305_wk48 | 25.0 (5/20) | 566 | 22.2 (4/18) | 358 | 0.0 (0/19) | | 0.0 (0/13) | |
| IgG4 | A | V1V2 | gp70-191084_B7 V1V2 | RV305_wk72 | 25.0 (5/20) | 665 | 16.7 (3/18) | 329 | 0.0 (0/18) | | 0.0 (0/13) | |
| IgG4 | B | V1V2 | gp70-62357.14 V1V2 | RV144_wk26 | 5.9 (1/17) | 378 | 0.0 (0/15) | | 0.0 (0/17) | | 0.0 (0/10) | |
| IgG4 | B | V1V2 | gp70-62357.14 V1V2 | RV305_wk0 | 5.0 (1/20) | 142 | 0.0 (0/18) | | 0.0 (0/19) | | 0.0 (0/13) | |
| IgG4 | B | V1V2 | gp70-62357.14 V1V2 | RV305_wk2 | 10.0 (2/20) | 1484 | 16.7 (3/18) | 686 | 0.0 (0/19) | | 0.0 (0/13) | |
| IgG4 | B | V1V2 | gp70-62357.14 V1V2 | RV305_wk24 | 10.0 (2/20) | 652 | 5.6 (1/18) | 1631 | 0.0 (0/19) | | 0.0 (0/13) | |
| IgG4 | B | V1V2 | gp70-62357.14 V1V2 | RV305_wk26 | 10.0 (2/20) | 1414 | 11.1 (2/18) | 3869 | 0.0 (0/19) | | 0.0 (0/13) | |
| IgG4 | B | V1V2 | gp70-62357.14 V1V2 | RV305_wk48 | 5.0 (1/20) | 581 | 11.1 (2/18) | 1326 | 0.0 (0/19) | | 0.0 (0/13) | |
| IgG4 | B | V1V2 | gp70-62357.14 V1V2 | RV305_wk72 | 10.0 (2/20) | 412 | 5.6 (1/18) | 1326 | 0.0 (0/18) | | 0.0 (0/13) | |
| IgG4 | B | V1V2 | gp70-700010058 V1V2 | RV144_wk26 | 17.6 (3/17) | 2318 | 0.0 (0/15) | | 0.0 (0/17) | | 0.0 (0/10) | |
| IgG4 | B | V1V2 | gp70-700010058 V1V2 | RV305_wk0 | 5.0 (1/20) | 3424 | 0.0 (0/18) | | 0.0 (0/19) | | 0.0 (0/13) | |
| IgG4 | B | V1V2 | gp70-700010058 V1V2 | RV305_wk2 | 55.0 (11/20) | 759 | 50.0 (9/18) | 354 | 0.0 (0/19) | | 0.0 (0/13) | |
| IgG4 | B | V1V2 | gp70-700010058 V1V2 | RV305_wk24 | 20.0 (4/20) | 1738 | 16.7 (3/18) | 148 | 0.0 (0/19) | | 0.0 (0/13) | |
| IgG4 | B | V1V2 | gp70-700010058 V1V2 | RV305_wk26 | 50.0 (10/20) | 1364 | 50.0 (9/18) | 680 | 0.0 (0/19) | | 0.0 (0/13) | |
| IgG4 | B | V1V2 | gp70-700010058 V1V2 | RV305_wk48 | 25.0 (5/20) | 270 | 22.2 (4/18) | 436 | 0.0 (0/19) | | 0.0 (0/13) | |
| IgG4 | B | V1V2 | gp70-700010058 V1V2 | RV305_wk72 | 20.0 (4/20) | 1014 | 11.1 (2/18) | 919 | 0.0 (0/18) | | 0.0 (0/13) | |

S12 Table continued

| | | | | | Group 1: Combination | | Group 2: AIDSVAX B/E | | Group 3: ALVAC-HIV | | RV305_Placebo Group | |
|---|---|---|---|---|---|---|---|---|---|---|---|---|
| Isotype | Clade | Env Region | Antigen | Study Week | Response Rate (Responders/ Total) | Median MFI Responders | Response Rate (Responders/ Total) | Median MFI Responders | Response Rate (Responders/ Total) | Median MFI Responders | Response Rate (Responders/ Total) | Median MFI Responders |
| IgG4 | B | V1V2 | gp70-RHPA4259.7 V1V2 | RV144_wk26 | 11.8 (2/17) | 245 | 0.0 (0/15) | | 0.0 (0/17) | | 0.0 (0/10) | |
| IgG4 | B | V1V2 | gp70-RHPA4259.7 V1V2 | RV305_wk0 | 0.0 (0/20) | | 0.0 (0/18) | | 0.0 (0/19) | | 0.0 (0/13) | |
| IgG4 | B | V1V2 | gp70-RHPA4259.7 V1V2 | RV305_wk2 | 20.0 (4/20) | 2447 | 22.2 (4/18) | 1087 | 0.0 (0/19) | | 0.0 (0/13) | |
| IgG4 | B | V1V2 | gp70-RHPA4259.7 V1V2 | RV305_wk24 | 15.0 (3/20) | 333 | 5.6 (1/18) | 2354 | 0.0 (0/19) | | 0.0 (0/13) | |
| IgG4 | B | V1V2 | gp70-RHPA4259.7 V1V2 | RV305_wk26 | 30.0 (6/20) | 189 | 16.7 (3/18) | 1311 | 0.0 (0/19) | | 0.0 (0/13) | |
| IgG4 | B | V1V2 | gp70-RHPA4259.7 V1V2 | RV305_wk48 | 10.0 (2/20) | 356 | 11.1 (2/18) | 2280 | 0.0 (0/19) | | 0.0 (0/13) | |
| IgG4 | B | V1V2 | gp70-RHPA4259.7 V1V2 | RV305_wk72 | 10.0 (2/20) | 237 | 5.6 (1/18) | 2023 | 0.0 (0/18) | | 0.0 (0/13) | |
| IgG4 | B | V1V2 | gp70-TT31P.2F10.2792 V1V2 | RV144_wk26 | 11.8 (2/17) | 386 | 0.0 (0/15) | | 0.0 (0/17) | | 0.0 (0/10) | |
| IgG4 | B | V1V2 | gp70-TT31P.2F10.2792 V1V2 | RV305_wk0 | 5.0 (1/20) | 174 | 0.0 (0/18) | | 0.0 (0/19) | | 0.0 (0/13) | |
| IgG4 | B | V1V2 | gp70-TT31P.2F10.2792 V1V2 | RV305_wk2 | 10.0 (2/20) | 6321 | 27.8 (5/18) | 205 | 0.0 (0/19) | | 0.0 (0/13) | |
| IgG4 | B | V1V2 | gp70-TT31P.2F10.2792 V1V2 | RV305_wk24 | 10.0 (2/20) | 2442 | 5.6 (1/18) | 1576 | 0.0 (0/19) | | 0.0 (0/13) | |
| IgG4 | B | V1V2 | gp70-TT31P.2F10.2792 V1V2 | RV305_wk26 | 10.0 (2/20) | 3350 | 27.8 (5/18) | 194 | 0.0 (0/19) | | 0.0 (0/13) | |
| IgG4 | B | V1V2 | gp70-TT31P.2F10.2792 V1V2 | RV305_wk48 | 5.0 (1/20) | 1052 | 5.6 (1/18) | 2493 | 0.0 (0/19) | | 0.0 (0/13) | |
| IgG4 | B | V1V2 | gp70-TT31P.2F10.2792 V1V2 | RV305_wk72 | 10.0 (2/20) | 643 | 5.6 (1/18) | 1154 | 0.0 (0/18) | | 0.0 (0/13) | |
| IgG4 | B | V1V2 | gp70_B.CaseA2 V1/V2/169K | RV144_wk26 | 17.6 (3/17) | 560 | 0.0 (0/15) | | 0.0 (0/17) | | 10.0 (1/10) | 200 |
| IgG4 | B | V1V2 | gp70_B.CaseA2 V1/V2/169K | RV305_wk0 | 5.0 (1/20) | 656 | 0.0 (0/18) | | 0.0 (0/19) | | 0.0 (0/13) | |
| IgG4 | B | V1V2 | gp70_B.CaseA2 V1/V2/169K | RV305_wk2 | 40.0 (8/20) | 694 | 55.6 (10/18) | 465 | 0.0 (0/19) | | 0.0 (0/13) | |
| IgG4 | B | V1V2 | gp70_B.CaseA2 V1/V2/169K | RV305_wk24 | 15.0 (3/20) | 11576 | 5.6 (1/18) | 4666 | 5.3 (1/19) | 108 | 0.0 (0/13) | |
| IgG4 | B | V1V2 | gp70_B.CaseA2 V1/V2/169K | RV305_wk26 | 35.0 (7/20) | 247 | 38.9 (7/18) | 753 | 0.0 (0/19) | | 0.0 (0/13) | |
| IgG4 | B | V1V2 | gp70_B.CaseA2 V1/V2/169K | RV305_wk48 | 15.0 (3/20) | 798 | 22.2 (4/18) | 312 | 0.0 (0/19) | | 0.0 (0/13) | |
| IgG4 | B | V1V2 | gp70_B.CaseA2 V1/V2/169K | RV305_wk72 | 15.0 (3/20) | 2080 | 11.1 (2/18) | 2644 | 0.0 (0/18) | | 0.0 (0/13) | |
| IgG4 | B | V1V2 | gp70_B.CaseA_V1_V2 | RV144_wk26 | 17.6 (3/17) | 664 | 0.0 (0/15) | | 0.0 (0/17) | | 0.0 (0/10) | |
| IgG4 | B | V1V2 | gp70_B.CaseA_V1_V2 | RV305_wk0 | 5.0 (1/20) | 666 | 0.0 (0/18) | | 0.0 (0/19) | | 0.0 (0/13) | |
| IgG4 | B | V1V2 | gp70_B.CaseA_V1_V2 | RV305_wk2 | 35.0 (7/20) | 409 | 44.4 (8/18) | 449 | 0.0 (0/19) | | 0.0 (0/13) | |
| IgG4 | B | V1V2 | gp70_B.CaseA_V1_V2 | RV305_wk24 | 20.0 (4/20) | 3010 | 5.6 (1/18) | 5391 | 5.3 (1/19) | 101 | 0.0 (0/13) | |
| IgG4 | B | V1V2 | gp70_B.CaseA_V1_V2 | RV305_wk26 | 40.0 (8/20) | 348 | 38.9 (7/18) | 853 | 0.0 (0/19) | | 0.0 (0/13) | |
| IgG4 | B | V1V2 | gp70_B.CaseA_V1_V2 | RV305_wk48 | 25.0 (5/20) | 159 | 27.8 (5/18) | 174 | 0.0 (0/19) | | 0.0 (0/13) | |
| IgG4 | B | V1V2 | gp70_B.CaseA_V1_V2 | RV305_wk72 | 20.0 (4/20) | 1162 | 5.6 (1/18) | 6519 | 0.0 (0/18) | | 0.0 (0/13) | |
| IgG4 | C | V1V2 | C.1086C_V1_V2 Tags | RV144_wk26 | 29.4 (5/17) | 260 | 6.7 (1/15) | 207 | 0.0 (0/17) | | 0.0 (0/10) | |
| IgG4 | C | V1V2 | C.1086C_V1_V2 Tags | RV305_wk0 | 5.0 (1/20) | 2051 | 0.0 (0/18) | | 0.0 (0/19) | | 0.0 (0/13) | |
| IgG4 | C | V1V2 | C.1086C_V1_V2 Tags | RV305_wk2 | 65.0 (13/20) | 833 | 77.8 (14/18) | 459 | 0.0 (0/19) | | 0.0 (0/13) | |
| IgG4 | C | V1V2 | C.1086C_V1_V2 Tags | RV305_wk24 | 45.0 (9/20) | 387 | 27.8 (5/18) | 395 | 5.3 (1/19) | 172 | 0.0 (0/13) | |
| IgG4 | C | V1V2 | C.1086C_V1_V2 Tags | RV305_wk26 | 50.0 (10/20) | 996 | 61.1 (11/18) | 392 | 0.0 (0/19) | | 0.0 (0/13) | |
| IgG4 | C | V1V2 | C.1086C_V1_V2 Tags | RV305_wk48 | 35.0 (7/20) | 445 | 44.4 (8/18) | 315 | 0.0 (0/19) | | 0.0 (0/13) | |
| IgG4 | C | V1V2 | C.1086C_V1_V2 Tags | RV305_wk72 | 30.0 (6/20) | 509 | 33.3 (6/18) | 203 | 0.0 (0/18) | | 0.0 (0/13) | |

S12 Table continued

| | | | | | Group 1: Combination | | Group 2: AIDSVAX B/E | | Group 3: ALVAC-HIV | | RV305_Placebo Group | |
|---|---|---|---|---|---|---|---|---|---|---|---|---|
| Isotype | Clade | Env Region | Antigen | Study Week | Response Rate (Responders/ Total) | Median MFI Responders | Response Rate (Responders/ Total) | Median MFI Responders | Response Rate (Responders/ Total) | Median MFI Responders | Response Rate (Responders/ Total) | Median MFI Responders |
| IgG4 | C | V1V2 | gp70-001428.2.42 V1V2 | RV144_wk26 | 11.8 (2/17) | 1703 | 0.0 (0/15) | | 0.0 (0/17) | | 0.0 (0/10) | |
| IgG4 | C | V1V2 | gp70-001428.2.42 V1V2 | RV305_wk0 | 5.0 (1/20) | 800 | 0.0 (0/18) | | 0.0 (0/19) | | 0.0 (0/13) | |
| IgG4 | C | V1V2 | gp70-001428.2.42 V1V2 | RV305_wk2 | 35.0 (7/20) | 290 | 38.9 (7/18) | 662 | 0.0 (0/19) | | 0.0 (0/13) | |
| IgG4 | C | V1V2 | gp70-001428.2.42 V1V2 | RV305_wk24 | 20.0 (4/20) | 1358 | 5.6 (1/18) | 895 | 0.0 (0/19) | | 0.0 (0/13) | |
| IgG4 | C | V1V2 | gp70-001428.2.42 V1V2 | RV305_wk26 | 35.0 (7/20) | 499 | 38.9 (7/18) | 771 | 0.0 (0/19) | | 0.0 (0/13) | |
| IgG4 | C | V1V2 | gp70-001428.2.42 V1V2 | RV305_wk48 | 10.0 (2/20) | 1089 | 22.2 (4/18) | 147 | 0.0 (0/19) | | 0.0 (0/13) | |
| IgG4 | C | V1V2 | gp70-001428.2.42 V1V2 | RV305_wk72 | 15.0 (3/20) | 870 | 11.1 (2/18) | 162 | 0.0 (0/18) | | 0.0 (0/13) | |
| IgG4 | C | V1V2 | gp70-7060101641 V1V2 | RV144_wk26 | 17.6 (3/17) | 457 | 0.0 (0/15) | | 0.0 (0/17) | | 10.0 (1/10) | 148 |
| IgG4 | C | V1V2 | gp70-7060101641 V1V2 | RV305_wk0 | 5.0 (1/20) | 871 | 0.0 (0/18) | | 0.0 (0/19) | | 0.0 (0/13) | |
| IgG4 | C | V1V2 | gp70-7060101641 V1V2 | RV305_wk2 | 40.0 (8/20) | 435 | 55.6 (10/18) | 362 | 0.0 (0/19) | | 0.0 (0/13) | |
| IgG4 | C | V1V2 | gp70-7060101641 V1V2 | RV305_wk24 | 20.0 (4/20) | 4902 | 11.1 (2/18) | 660 | 5.3 (1/19) | 266 | 0.0 (0/13) | |
| IgG4 | C | V1V2 | gp70-7060101641 V1V2 | RV305_wk26 | 40.0 (8/20) | 998 | 38.9 (7/18) | 351 | 0.0 (0/19) | | 0.0 (0/13) | |
| IgG4 | C | V1V2 | gp70-7060101641 V1V2 | RV305_wk48 | 20.0 (4/20) | 133 | 16.7 (3/18) | 1301 | 0.0 (0/19) | | 0.0 (0/13) | |
| IgG4 | C | V1V2 | gp70-7060101641 V1V2 | RV305_wk72 | 15.0 (3/20) | 2627 | 11.1 (2/18) | 650 | 0.0 (0/18) | | 0.0 (0/13) | |
| IgG4 | C | V1V2 | gp70-96ZM651.02 V1v2 | RV144_wk26 | 17.6 (3/17) | 2530 | 0.0 (0/15) | | 0.0 (0/17) | | 20.0 (2/10) | 222 |
| IgG4 | C | V1V2 | gp70-96ZM651.02 V1v2 | RV305_wk0 | 5.0 (1/20) | 5157 | 0.0 (0/18) | | 0.0 (0/19) | | 0.0 (0/13) | |
| IgG4 | C | V1V2 | gp70-96ZM651.02 V1v2 | RV305_wk2 | 45.0 (9/20) | 1054 | 50.0 (9/18) | 1473 | 5.3 (1/19) | 128 | 0.0 (0/13) | |
| IgG4 | C | V1V2 | gp70-96ZM651.02 V1v2 | RV305_wk24 | 25.0 (5/20) | 238 | 16.7 (3/18) | 445 | 5.3 (1/19) | 141 | 0.0 (0/13) | |
| IgG4 | C | V1V2 | gp70-96ZM651.02 V1v2 | RV305_wk26 | 45.0 (9/20) | 834 | 38.9 (7/18) | 369 | 5.3 (1/19) | 101 | 0.0 (0/13) | |
| IgG4 | C | V1V2 | gp70-96ZM651.02 V1v2 | RV305_wk48 | 15.0 (3/20) | 3364 | 22.2 (4/18) | 797 | 0.0 (0/19) | | 0.0 (0/13) | |
| IgG4 | C | V1V2 | gp70-96ZM651.02 V1v2 | RV305_wk72 | 20.0 (4/20) | 6875 | 11.1 (2/18) | 836 | 0.0 (0/18) | | 0.0 (0/13) | |
| IgG4 | C | V1V2 | gp70-BF1266_431a_V1V2 | RV144_wk26 | 5.9 (1/17) | 140 | 0.0 (0/15) | | 0.0 (0/17) | | 0.0 (0/10) | |
| IgG4 | C | V1V2 | gp70-BF1266_431a_V1V2 | RV305_wk0 | 0.0 (0/20) | | 0.0 (0/18) | | 0.0 (0/19) | | 0.0 (0/13) | |
| IgG4 | C | V1V2 | gp70-BF1266_431a_V1V2 | RV305_wk2 | 15.0 (3/20) | 478 | 27.8 (5/18) | 866 | 0.0 (0/19) | | 0.0 (0/13) | |
| IgG4 | C | V1V2 | gp70-BF1266_431a_V1V2 | RV305_wk24 | 5.0 (1/20) | 448 | 11.1 (2/18) | 1666 | 0.0 (0/19) | | 0.0 (0/13) | |
| IgG4 | C | V1V2 | gp70-BF1266_431a_V1V2 | RV305_wk26 | 10.0 (2/20) | 5055 | 22.2 (4/18) | 1381 | 0.0 (0/19) | | 0.0 (0/13) | |
| IgG4 | C | V1V2 | gp70-BF1266_431a_V1V2 | RV305_wk48 | 5.0 (1/20) | 795 | 16.7 (3/18) | 1587 | 0.0 (0/19) | | 0.0 (0/13) | |
| IgG4 | C | V1V2 | gp70-BF1266_431a_V1V2 | RV305_wk72 | 5.0 (1/20) | 404 | 11.1 (2/18) | 2199 | 0.0 (0/18) | | 0.0 (0/13) | |
| IgG4 | C | V1V2 | gp70-CAP210.2.00.E8 V1V2 | RV144_wk26 | 11.8 (2/17) | 468 | 0.0 (0/15) | | 0.0 (0/17) | | 0.0 (0/10) | |
| IgG4 | C | V1V2 | gp70-CAP210.2.00.E8 V1V2 | RV305_wk0 | 5.0 (1/20) | 139 | 0.0 (0/18) | | 0.0 (0/19) | | 0.0 (0/13) | |
| IgG4 | C | V1V2 | gp70-CAP210.2.00.E8 V1V2 | RV305_wk2 | 20.0 (4/20) | 431 | 22.2 (4/18) | 466 | 0.0 (0/19) | | 0.0 (0/13) | |
| IgG4 | C | V1V2 | gp70-CAP210.2.00.E8 V1V2 | RV305_wk24 | 10.0 (2/20) | 1062 | 5.6 (1/18) | 979 | 0.0 (0/19) | | 0.0 (0/13) | |
| IgG4 | C | V1V2 | gp70-CAP210.2.00.E8 V1V2 | RV305_wk26 | 15.0 (3/20) | 232 | 22.2 (4/18) | 527 | 0.0 (0/19) | | 0.0 (0/13) | |
| IgG4 | C | V1V2 | gp70-CAP210.2.00.E8 V1V2 | RV305_wk48 | 5.0 (1/20) | 380 | 5.6 (1/18) | 2341 | 0.0 (0/19) | | 0.0 (0/13) | |
| IgG4 | C | V1V2 | gp70-CAP210.2.00.E8 V1V2 | RV305_wk72 | 10.0 (2/20) | 381 | 5.6 (1/18) | 1007 | 0.0 (0/18) | | 0.0 (0/13) | |

| | | | | | Group 1: Combination | | Group 2: AIDSVAX B/E | | Group 3: ALVAC-HIV | | RV305_Placebo Group | |
|---|---|---|---|---|---|---|---|---|---|---|---|---|
| Isotype | Clade | Env Region | Antigen | Study Week | Response Rate (Responders/ Total) | Median MFI Responders | Response Rate (Responders/ Total) | Median MFI Responders | Response Rate (Responders/ Total) | Median MFI Responders | Response Rate (Responders/ Total) | Median MFI Responders |
| IgG4 | C | V1V2 | gp70-Ce1086_B2 V1V2 | RV144_wk26 | 58.8 (10/17) | 258 | 40.0 (6/15) | 156 | 23.5 (4/17) | 213 | 20.0 (2/10) | 218 |
| IgG4 | C | V1V2 | gp70-Ce1086_B2 V1V2 | RV305_wk0 | 5.0 (1/20) | 9844 | 0.0 (0/18) | | 0.0 (0/19) | | 0.0 (0/13) | |
| IgG4 | C | V1V2 | gp70-Ce1086_B2 V1V2 | RV305_wk2 | 80.0 (16/20) | 1150 | 83.3 (15/18) | 1069 | 0.0 (0/19) | | 0.0 (0/13) | |
| IgG4 | C | V1V2 | gp70-Ce1086_B2 V1V2 | RV305_wk24 | 50.0 (10/20) | 1312 | 38.9 (7/18) | 1325 | 0.0 (0/19) | | 0.0 (0/13) | |
| IgG4 | C | V1V2 | gp70-Ce1086_B2 V1V2 | RV305_wk26 | 80.0 (16/20) | 1686 | 72.2 (13/18) | 789 | 5.3 (1/19) | 131 | 0.0 (0/13) | |
| IgG4 | C | V1V2 | gp70-Ce1086_B2 V1V2 | RV305_wk48 | 60.0 (12/20) | 965 | 55.6 (10/18) | 753 | 0.0 (0/19) | | 0.0 (0/13) | |
| IgG4 | C | V1V2 | gp70-Ce1086_B2 V1V2 | RV305_wk72 | 55.0 (11/20) | 1243 | 44.4 (8/18) | 728 | 0.0 (0/18) | | 0.0 (0/13) | |
| IgG4 | C | V1V2 | gp70-TV1.21 V1V2 | RV144_wk26 | 11.8 (2/17) | 389 | 0.0 (0/15) | | 0.0 (0/17) | | 0.0 (0/10) | |
| IgG4 | C | V1V2 | gp70-TV1.21 V1V2 | RV305_wk0 | 0.0 (0/20) | | 0.0 (0/18) | | 0.0 (0/19) | | 0.0 (0/13) | |
| IgG4 | C | V1V2 | gp70-TV1.21 V1V2 | RV305_wk2 | 15.0 (3/20) | 1120 | 44.4 (8/18) | 213 | 0.0 (0/19) | | 0.0 (0/13) | |
| IgG4 | C | V1V2 | gp70-TV1.21 V1V2 | RV305_wk24 | 10.0 (2/20) | 1357 | 5.6 (1/18) | 2932 | 0.0 (0/19) | | 0.0 (0/13) | |
| IgG4 | C | V1V2 | gp70-TV1.21 V1V2 | RV305_wk26 | 10.0 (2/20) | 5899 | 22.2 (4/18) | 1077 | 0.0 (0/19) | | 0.0 (0/13) | |
| IgG4 | C | V1V2 | gp70-TV1.21 V1V2 | RV305_wk48 | 5.0 (1/20) | 1367 | 16.7 (3/18) | 253 | 0.0 (0/19) | | 0.0 (0/13) | |
| IgG4 | C | V1V2 | gp70-TV1.21 V1V2 | RV305_wk72 | 10.0 (2/20) | 532 | 11.1 (2/18) | 1138 | 0.0 (0/18) | | 0.0 (0/13) | |
| IgG4 | CRF01_AE | V1V2 | AE.A244 V1V2 tags | RV144_wk26 | 76.5 (13/17) | 257 | 40.0 (6/15) | 130 | 35.3 (6/17) | 244 | 50.0 (5/10) | 228 |
| IgG4 | CRF01_AE | V1V2 | AE.A244 V1V2 tags | RV305_wk0 | 10.0 (2/20) | 1689 | 0.0 (0/18) | | 0.0 (0/19) | | 0.0 (0/13) | |
| IgG4 | CRF01_AE | V1V2 | AE.A244 V1V2 tags | RV305_wk2 | 75.0 (15/20) | 1654 | 83.3 (15/18) | 959 | 5.3 (1/19) | 276 | 0.0 (0/13) | |
| IgG4 | CRF01_AE | V1V2 | AE.A244 V1V2 tags | RV305_wk24 | 50.0 (10/20) | 712 | 44.4 (8/18) | 769 | 5.3 (1/19) | 1727 | 0.0 (0/13) | |
| IgG4 | CRF01_AE | V1V2 | AE.A244 V1V2 tags | RV305_wk26 | 85.0 (17/20) | 1504 | 83.3 (15/18) | 612 | 5.3 (1/19) | 320 | 0.0 (0/13) | |
| IgG4 | CRF01_AE | V1V2 | AE.A244 V1V2 tags | RV305_wk48 | 55.0 (11/20) | 903 | 55.6 (10/18) | 1364 | 0.0 (0/19) | | 0.0 (0/13) | |
| IgG4 | CRF01_AE | V1V2 | AE.A244 V1V2 tags | RV305_wk72 | 50.0 (10/20) | 727 | 44.4 (8/18) | 785 | 5.6 (1/18) | 351 | 0.0 (0/13) | |
| IgG4 | CRF01_AE | V1V2 | gp70-C2101.c01_V1V2 | RV144_wk26 | 17.6 (3/17) | 886 | 0.0 (0/15) | | 0.0 (0/17) | | 10.0 (1/10) | 319 |
| IgG4 | CRF01_AE | V1V2 | gp70-C2101.c01_V1V2 | RV305_wk0 | 5.0 (1/20) | 3595 | 0.0 (0/18) | | 0.0 (0/19) | | 0.0 (0/13) | |
| IgG4 | CRF01_AE | V1V2 | gp70-C2101.c01_V1V2 | RV305_wk2 | 45.0 (9/20) | 582 | 55.6 (10/18) | 295 | 0.0 (0/19) | | 0.0 (0/13) | |
| IgG4 | CRF01_AE | V1V2 | gp70-C2101.c01_V1V2 | RV305_wk24 | 25.0 (5/20) | 932 | 27.8 (5/18) | 139 | 5.3 (1/19) | 343 | 0.0 (0/13) | |
| IgG4 | CRF01_AE | V1V2 | gp70-C2101.c01_V1V2 | RV305_wk26 | 45.0 (9/20) | 1550 | 50.0 (9/18) | 600 | 0.0 (0/19) | | 0.0 (0/13) | |
| IgG4 | CRF01_AE | V1V2 | gp70-C2101.c01_V1V2 | RV305_wk48 | 25.0 (5/20) | 290 | 33.3 (6/18) | 281 | 0.0 (0/19) | | 0.0 (0/13) | |
| IgG4 | CRF01_AE | V1V2 | gp70-C2101.c01_V1V2 | RV305_wk72 | 25.0 (5/20) | 846 | 16.7 (3/18) | 685 | 0.0 (0/18) | | 0.0 (0/13) | |
| IgG4 | CRF01_AE | V1V2 | gp70-CM244.ec1 V1V2 | RV144_wk26 | 58.8 (10/17) | 492 | 40.0 (6/15) | 311 | 41.2 (7/17) | 330 | 70.0 (7/10) | 164 |
| IgG4 | CRF01_AE | V1V2 | gp70-CM244.ec1 V1V2 | RV305_wk0 | 5.0 (1/20) | 13014 | 5.6 (1/18) | 132 | 0.0 (0/19) | | 0.0 (0/13) | |
| IgG4 | CRF01_AE | V1V2 | gp70-CM244.ec1 V1V2 | RV305_wk2 | 90.0 (18/20) | 1109 | 83.3 (15/18) | 1575 | 21.1 (4/19) | 148 | 0.0 (0/13) | |
| IgG4 | CRF01_AE | V1V2 | gp70-CM244.ec1 V1V2 | RV305_wk24 | 75.0 (15/20) | 1741 | 50.0 (9/18) | 1402 | 5.3 (1/19) | 1708 | 0.0 (0/13) | |
| IgG4 | CRF01_AE | V1V2 | gp70-CM244.ec1 V1V2 | RV305_wk26 | 85.0 (17/20) | 2287 | 83.3 (15/18) | 1012 | 21.1 (4/19) | 142 | 0.0 (0/13) | |
| IgG4 | CRF01_AE | V1V2 | gp70-CM244.ec1 V1V2 | RV305_wk48 | 70.0 (14/20) | 932 | 72.2 (13/18) | 1282 | 0.0 (0/19) | | 0.0 (0/13) | |
| IgG4 | CRF01_AE | V1V2 | gp70-CM244.ec1 V1V2 | RV305_wk72 | 65.0 (13/20) | 1988 | 66.7 (12/18) | 668 | 11.1 (2/18) | 151 | 0.0 (0/13) | |

| | | | | | Group 1: Combination | | Group 2: AIDSVAX B/E | | Group 3: ALVAC-HIV | | RV305_Placebo Group | |
|---|---|---|---|---|---|---|---|---|---|---|---|---|
| Isotype | Clade | Env Region | Antigen | Study Week | Response Rate (Responders/ Total) | Median MFI Responders | Response Rate (Responders/ Total) | Median MFI Responders | Response Rate (Responders/ Total) | Median MFI Responders | Response Rate (Responders/ Total) | Median MFI Responders |
| IgG4 | CRF07_BC | V1V2 | gp70-BJOX002000.03.2 V1V2 | RV144_wk26 | 17.6 (3/17) | 2303 | 0.0 (0/15) | | 0.0 (0/17) | | 10.0 (1/10) | 148 |
| IgG4 | CRF07_BC | V1V2 | gp70-BJOX002000.03.2 V1V2 | RV305_wk0 | 5.0 (1/20) | 4092 | 0.0 (0/18) | | 0.0 (0/19) | | 0.0 (0/13) | |
| IgG4 | CRF07_BC | V1V2 | gp70-BJOX002000.03.2 V1V2 | RV305_wk2 | 45.0 (9/20) | 468 | 50.0 (9/18) | 551 | 0.0 (0/19) | | 0.0 (0/13) | |
| IgG4 | CRF07_BC | V1V2 | gp70-BJOX002000.03.2 V1V2 | RV305_wk24 | 25.0 (5/20) | 281 | 16.7 (3/18) | 203 | 0.0 (0/19) | | 0.0 (0/13) | |
| IgG4 | CRF07_BC | V1V2 | gp70-BJOX002000.03.2 V1V2 | RV305_wk26 | 50.0 (10/20) | 485 | 55.6 (10/18) | 443 | 0.0 (0/19) | | 0.0 (0/13) | |
| IgG4 | CRF07_BC | V1V2 | gp70-BJOX002000.03.2 V1V2 | RV305_wk48 | 25.0 (5/20) | 260 | 16.7 (3/18) | 1211 | 0.0 (0/19) | | 0.0 (0/13) | |
| IgG4 | CRF07_BC | V1V2 | gp70-BJOX002000.03.2 V1V2 | RV305_wk72 | 25.0 (5/20) | 186 | 16.7 (3/18) | 309 | 0.0 (0/18) | | 0.0 (0/13) | |
| IgG4 | CRF01_AE | V2 | AE.A244 V2 tags/293F | RV144_wk26 | 23.5 (4/17) | 357 | 6.7 (1/15) | 203 | 0.0 (0/17) | | 0.0 (0/10) | |
| IgG4 | CRF01_AE | V2 | AE.A244 V2 tags/293F | RV305_wk0 | 5.0 (1/20) | 1195 | 0.0 (0/18) | | 0.0 (0/19) | | 0.0 (0/13) | |
| IgG4 | CRF01_AE | V2 | AE.A244 V2 tags/293F | RV305_wk2 | 55.0 (11/20) | 603 | 61.1 (11/18) | 429 | 0.0 (0/19) | | 0.0 (0/13) | |
| IgG4 | CRF01_AE | V2 | AE.A244 V2 tags/293F | RV305_wk24 | 45.0 (9/20) | 271 | 27.8 (5/18) | 551 | 5.3 (1/19) | 171 | 0.0 (0/13) | |
| IgG4 | CRF01_AE | V2 | AE.A244 V2 tags/293F | RV305_wk26 | 45.0 (9/20) | 883 | 44.4 (8/18) | 671 | 0.0 (0/19) | | 0.0 (0/13) | |
| IgG4 | CRF01_AE | V2 | AE.A244 V2 tags/293F | RV305_wk48 | 35.0 (7/20) | 413 | 33.3 (6/18) | 727 | 0.0 (0/19) | | 0.0 (0/13) | |
| IgG4 | CRF01_AE | V2 | AE.A244 V2 tags/293F | RV305_wk72 | 35.0 (7/20) | 314 | 27.8 (5/18) | 555 | 0.0 (0/18) | | 0.0 (0/13) | |
| IgG4 | B | V3 | B.MN V3 gp70 | RV144_wk26 | 11.8 (2/17) | 194 | 0.0 (0/15) | | 0.0 (0/17) | | 0.0 (0/10) | |
| IgG4 | B | V3 | B.MN V3 gp70 | RV305_wk0 | 0.0 (0/20) | | 0.0 (0/18) | | 0.0 (0/19) | | 0.0 (0/13) | |
| IgG4 | B | V3 | B.MN V3 gp70 | RV305_wk2 | 35.0 (7/20) | 552 | 38.9 (7/18) | 532 | 5.3 (1/19) | 323 | 0.0 (0/13) | |
| IgG4 | B | V3 | B.MN V3 gp70 | RV305_wk24 | 20.0 (4/20) | 140 | 16.7 (3/18) | 265 | 0.0 (0/19) | | 0.0 (0/13) | |
| IgG4 | B | V3 | B.MN V3 gp70 | RV305_wk26 | 45.0 (9/20) | 358 | 33.3 (6/18) | 267 | 0.0 (0/19) | | 0.0 (0/13) | |
| IgG4 | B | V3 | B.MN V3 gp70 | RV305_wk48 | 15.0 (3/20) | 140 | 22.2 (4/18) | 415 | 5.3 (1/19) | 130 | 0.0 (0/13) | |
| IgG4 | B | V3 | B.MN V3 gp70 | RV305_wk72 | 5.0 (1/20) | 377 | 16.7 (3/18) | 501 | 0.0 (0/18) | | 0.0 (0/13) | |
| IgG4 | N/A | CD4i | HxB2 new 8b core 6x His | RV144_wk26 | 17.6 (3/17) | 1314 | 6.7 (1/15) | 127 | 5.9 (1/17) | 104 | 0.0 (0/10) | |
| IgG4 | N/A | CD4i | HxB2 new 8b core 6x His | RV305_wk0 | 5.0 (1/20) | 951 | 5.6 (1/18) | 214 | 0.0 (0/19) | | 0.0 (0/13) | |
| IgG4 | N/A | CD4i | HxB2 new 8b core 6x His | RV305_wk2 | 35.0 (7/20) | 1167 | 44.4 (8/18) | 1393 | 0.0 (0/19) | | 0.0 (0/13) | |
| IgG4 | N/A | CD4i | HxB2 new 8b core 6x His | RV305_wk24 | 70.0 (14/20) | 935 | 50.0 (9/18) | 974 | 0.0 (0/19) | | 0.0 (0/13) | |
| IgG4 | N/A | CD4i | HxB2 new 8b core 6x His | RV305_wk26 | 60.0 (12/20) | 1402 | 55.6 (10/18) | 1865 | 5.3 (1/19) | 211 | 0.0 (0/13) | |
| IgG4 | N/A | CD4i | HxB2 new 8b core 6x His | RV305_wk48 | 55.0 (11/20) | 757 | 61.1 (11/18) | 1156 | 5.3 (1/19) | 167 | 0.0 (0/13) | |
| IgG4 | N/A | CD4i | HxB2 new 8b core 6x His | RV305_wk72 | 65.0 (13/20) | 564 | 61.1 (11/18) | 637 | 5.6 (1/18) | 114 | 0.0 (0/13) | |
| IgG4 | N/A | CD4i | YU2 gp120 WT | RV144_wk26 | 23.5 (4/17) | 3871 | 13.3 (2/15) | 625 | 5.9 (1/17) | 1488 | 0.0 (0/10) | |
| IgG4 | N/A | CD4i | YU2 gp120 WT | RV305_wk0 | 15.0 (3/20) | 1675 | 11.1 (2/18) | 742 | 0.0 (0/19) | | 0.0 (0/13) | |
| IgG4 | N/A | CD4i | YU2 gp120 WT | RV305_wk2 | 65.0 (13/20) | 4394 | 33.3 (6/18) | 3375 | 0.0 (0/19) | | 0.0 (0/13) | |
| IgG4 | N/A | CD4i | YU2 gp120 WT | RV305_wk24 | 100 (20/20) | 4188 | 77.8 (14/18) | 2868 | 5.3 (1/19) | 2982 | 0.0 (0/13) | |
| IgG4 | N/A | CD4i | YU2 gp120 WT | RV305_wk26 | 70.0 (14/20) | 3677 | 44.4 (8/18) | 5810 | 10.5 (2/19) | 215 | 0.0 (0/13) | |
| IgG4 | N/A | CD4i | YU2 gp120 WT | RV305_wk48 | 90.0 (18/20) | 3638 | 66.7 (12/18) | 4240 | 15.8 (3/19) | 268 | 0.0 (0/13) | |
| IgG4 | N/A | CD4i | YU2 gp120 WT | RV305_wk72 | 95.0 (19/20) | 2543 | 77.8 (14/18) | 2495 | 16.7 (3/18) | 224 | 0.0 (0/13) | |

S12 Table continued

| | | | | | Group 1: Combination | | Group 2: AIDSVAX B/E | | Group 3: ALVAC-HIV | | RV305_Placebo Group | |
|---|---|---|---|---|---|---|---|---|---|---|---|---|
| **Isotype** | **Clade** | **Env Region** | **Antigen** | **Study Week** | **Response Rate (Responders/ Total)** | **Median MFI Responders** | **Response Rate (Responders/ Total)** | **Median MFI Responders** | **Response Rate (Responders/ Total)** | **Median MFI Responders** | **Response Rate (Responders/ Total)** | **Median MFI Responders** |
| IgG4 | N/A | CD4bs | RSC3 | RV144_wk26 | 0.0 (0/17) | | 0.0 (0/15) | | 0.0 (0/17) | | 0.0 (0/10) | |
| IgG4 | N/A | CD4bs | RSC3 | RV305_wk0 | 0.0 (0/20) | | 0.0 (0/18) | | 0.0 (0/19) | | 0.0 (0/13) | |
| IgG4 | N/A | CD4bs | RSC3 | RV305_wk2 | 0.0 (0/20) | | 0.0 (0/18) | | 0.0 (0/19) | | 0.0 (0/13) | |
| IgG4 | N/A | CD4bs | RSC3 | RV305_wk24 | 0.0 (0/20) | | 0.0 (0/18) | | 0.0 (0/19) | | 0.0 (0/13) | |
| IgG4 | N/A | CD4bs | RSC3 | RV305_wk26 | 0.0 (0/20) | | 0.0 (0/18) | | 0.0 (0/19) | | 0.0 (0/13) | |
| IgG4 | N/A | CD4bs | RSC3 | RV305_wk48 | 0.0 (0/20) | | 0.0 (0/18) | | 0.0 (0/19) | | 0.0 (0/13) | |
| IgG4 | N/A | CD4bs | RSC3 | RV305_wk72 | 0.0 (0/20) | | 0.0 (0/18) | | 0.0 (0/18) | | 0.0 (0/13) | |
| IgG4 | N/A | CD4bs | RSC3_P363Npair | RV144_wk26 | 0.0 (0/17) | | 0.0 (0/15) | | 0.0 (0/17) | | 0.0 (0/10) | |
| IgG4 | N/A | CD4bs | RSC3_P363Npair | RV305_wk0 | 0.0 (0/20) | | 0.0 (0/18) | | 0.0 (0/19) | | 0.0 (0/13) | |
| IgG4 | N/A | CD4bs | RSC3_P363Npair | RV305_wk2 | 0.0 (0/20) | | 0.0 (0/18) | | 0.0 (0/19) | | 0.0 (0/13) | |
| IgG4 | N/A | CD4bs | RSC3_P363Npair | RV305_wk24 | 0.0 (0/20) | | 0.0 (0/18) | | 0.0 (0/19) | | 0.0 (0/13) | |
| IgG4 | N/A | CD4bs | RSC3_P363Npair | RV305_wk26 | 0.0 (0/20) | | 0.0 (0/18) | | 0.0 (0/19) | | 0.0 (0/13) | |
| IgG4 | N/A | CD4bs | RSC3_P363Npair | RV305_wk48 | 0.0 (0/20) | | 0.0 (0/18) | | 0.0 (0/19) | | 0.0 (0/13) | |
| IgG4 | N/A | CD4bs | RSC3_P363Npair | RV305_wk72 | 0.0 (0/20) | | 0.0 (0/18) | | 0.0 (0/18) | | 0.0 (0/13) | |
| IgG4 | N/A | Gag (non-Env) | p24 | RV144_wk26 | 0.0 (0/17) | | 0.0 (0/15) | | 0.0 (0/17) | | 0.0 (0/10) | |
| IgG4 | N/A | Gag (non-Env) | p24 | RV305_wk0 | 0.0 (0/20) | | 0.0 (0/18) | | 5.3 (1/19) | 470 | 0.0 (0/13) | |
| IgG4 | N/A | Gag (non-Env) | p24 | RV305_wk2 | 5.0 (1/20) | 157 | 5.6 (1/18) | 107 | 0.0 (0/19) | | 0.0 (0/13) | |
| IgG4 | N/A | Gag (non-Env) | p24 | RV305_wk24 | 0.0 (0/20) | | 0.0 (0/18) | | 5.3 (1/19) | 134 | 0.0 (0/13) | |
| IgG4 | N/A | Gag (non-Env) | p24 | RV305_wk26 | 10.0 (2/20) | 372 | 0.0 (0/18) | | 5.3 (1/19) | 123 | 0.0 (0/13) | |
| IgG4 | N/A | Gag (non-Env) | p24 | RV305_wk48 | 10.0 (2/20) | 133 | 0.0 (0/18) | | 0.0 (0/19) | | 0.0 (0/13) | |
| IgG4 | N/A | Gag (non-Env) | p24 | RV305_wk72 | 5.0 (1/20) | 125 | 0.0 (0/18) | | 0.0 (0/18) | | 0.0 (0/13) | |
