# Supplementary material for: Viral vector delivered immunogen focuses HIV-1 antibody specificity and increases durability of the circulating antibody recall response
Source: PLoS Pathog. 2023 May 31;19(5):e1011359. doi: 10.1371/journal.ppat.1011359 (PMC10284421; doi:10.1371/journal.ppat.1011359)
Supplement: S13 Table — (PDF) [file ppat.1011359.s026.pdf]

S13 Table. IgA1 percent responders and group median binding magnitudes (MFI) by antigen, study week, and group.

					Group 1: Combination		Group 2: AIDSVAX B/E		Group 3: ALVAC-HIV	
**Isotype**	**Clade**	**Env Region**	**Antigen**	**Study Week**	**Response Rate *(Responders/ Total)***	**Median MFI *Responders***	**Response Rate *(Responders/ Total)***	**Median MFI *Responders***	**Response Rate *(Responders/ Total)***	**Median MFI *Responders***
IgA1	B	gp120	MN gp120 gDneg/293F/mon	RV144_wk26	41.7 (5/12)	279	25.0 (2/8)	516	16.7 (2/12)	365
IgA1	B	gp120	MN gp120 gDneg/293F/mon	RV305_wk0	0.0 (0/12)		0.0 (0/8)		0.0 (0/12)	
IgA1	B	gp120	MN gp120 gDneg/293F/mon	RV305_wk2	83.3 (10/12)	380	75.0 (6/8)	300	0.0 (0/12)	
IgA1	B	gp120	MN gp120 gDneg/293F/mon	RV305_wk26	50.0 (6/12)	421	62.5 (5/8)	174	0.0 (0/12)	
IgA1	B	gp120	MN gp120 gDneg/293F/mon	RV305_wk48	16.7 (2/12)	220	12.5 (1/8)	720	8.3 (1/12)	133
IgA1	B	gp120	MN gp120 gDneg/293F/mon	RV305_wk72	16.7 (2/12)	152	12.5 (1/8)	401	8.3 (1/12)	181
IgA1	CRF01_AE	gp120	92TH023 gp120 gDneg 293F mon	RV144_wk26	33.3 (4/12)	312	25.0 (2/8)	418	16.7 (2/12)	398
IgA1	CRF01_AE	gp120	92TH023 gp120 gDneg 293F mon	RV305_wk0	0.0 (0/12)		0.0 (0/8)		0.0 (0/12)	
IgA1	CRF01_AE	gp120	92TH023 gp120 gDneg 293F mon	RV305_wk2	75.0 (9/12)	606	87.5 (7/8)	347	0.0 (0/12)	
IgA1	CRF01_AE	gp120	92TH023 gp120 gDneg 293F mon	RV305_wk26	50.0 (6/12)	606	37.5 (3/8)	125	0.0 (0/12)	
IgA1	CRF01_AE	gp120	92TH023 gp120 gDneg 293F mon	RV305_wk48	25.0 (3/12)	260	12.5 (1/8)	223	0.0 (0/12)	
IgA1	CRF01_AE	gp120	92TH023 gp120 gDneg 293F mon	RV305_wk72	25.0 (3/12)	159	12.5 (1/8)	122	0.0 (0/12)	
IgA1	CRF01_AE	gp120	A244 D11gp120_avi	RV144_wk26	33.3 (4/12)	1961	50.0 (4/8)	454	25.0 (3/12)	521
IgA1	CRF01_AE	gp120	A244 D11gp120_avi	RV305_wk0	0.0 (0/12)		0.0 (0/8)		8.3 (1/12)	398
IgA1	CRF01_AE	gp120	A244 D11gp120_avi	RV305_wk2	83.3 (10/12)	970	100 (8/8)	653	8.3 (1/12)	398
IgA1	CRF01_AE	gp120	A244 D11gp120_avi	RV305_wk26	50.0 (6/12)	1039	50.0 (4/8)	283	8.3 (1/12)	481
IgA1	CRF01_AE	gp120	A244 D11gp120_avi	RV305_wk48	33.3 (4/12)	937	12.5 (1/8)	668	8.3 (1/12)	687
IgA1	CRF01_AE	gp120	A244 D11gp120_avi	RV305_wk72	25.0 (3/12)	958	0.0 (0/8)		8.3 (1/12)	931
IgA1	Consensus	gp120	Con 6 gp120/B	RV144_wk26	58.3 (7/12)	724	50.0 (4/8)	458	25.0 (3/12)	320
IgA1	Consensus	gp120	Con 6 gp120/B	RV305_wk0	0.0 (0/12)		0.0 (0/8)		0.0 (0/12)	
IgA1	Consensus	gp120	Con 6 gp120/B	RV305_wk2	91.7 (11/12)	822	100 (8/8)	334	0.0 (0/12)	
IgA1	Consensus	gp120	Con 6 gp120/B	RV305_wk26	58.3 (7/12)	289	50.0 (4/8)	271	0.0 (0/12)	
IgA1	Consensus	gp120	Con 6 gp120/B	RV305_wk48	25.0 (3/12)	757	12.5 (1/8)	550	0.0 (0/12)	
IgA1	Consensus	gp120	Con 6 gp120/B	RV305_wk72	25.0 (3/12)	547	25.0 (2/8)	245	0.0 (0/12)	
IgA1	A	gp140	00MSA 4076 gp140	RV144_wk26	8.3 (1/12)	134	0.0 (0/8)		8.3 (1/12)	186
IgA1	A	gp140	00MSA 4076 gp140	RV305_wk0	8.3 (1/12)	232	0.0 (0/8)		0.0 (0/12)	
IgA1	A	gp140	00MSA 4076 gp140	RV305_wk2	66.7 (8/12)	502	37.5 (3/8)	473	0.0 (0/12)	
IgA1	A	gp140	00MSA 4076 gp140	RV305_wk26	25.0 (3/12)	206	25.0 (2/8)	1266	0.0 (0/12)	
IgA1	A	gp140	00MSA 4076 gp140	RV305_wk48	0.0 (0/12)		0.0 (0/8)		0.0 (0/12)	
IgA1	A	gp140	00MSA 4076 gp140	RV305_wk72	8.3 (1/12)	130	0.0 (0/8)		0.0 (0/12)	

S13 Table continued

					Group 1: Combination		Group 2: AIDSVAX B/E		Group 3: ALVAC-HIV	
**Isotype**	**Clade**	**Env Region**	**Antigen**	**Study Week**	**Response Rate *(Responders/ Total)***	**Median MFI *Responders***	**Response Rate *(Responders/ Total)***	**Median MFI *Responders***	**Response Rate *(Responders/ Total)***	**Median MFI *Responders***
IgA1	A	gp140	A1.con.env03 140 CF	RV144_wk26	0.0 (0/12)		0.0 (0/8)		0.0 (0/12)	
IgA1	A	gp140	A1.con.env03 140 CF	RV305_wk0	0.0 (0/12)		0.0 (0/8)		0.0 (0/12)	
IgA1	A	gp140	A1.con.env03 140 CF	RV305_wk2	16.7 (2/12)	503	12.5 (1/8)	6300	0.0 (0/12)	
IgA1	A	gp140	A1.con.env03 140 CF	RV305_wk26	16.7 (2/12)	149	12.5 (1/8)	3089	0.0 (0/12)	
IgA1	A	gp140	A1.con.env03 140 CF	RV305_wk48	0.0 (0/12)		12.5 (1/8)	134	0.0 (0/12)	
IgA1	A	gp140	A1.con.env03 140 CF	RV305_wk72	0.0 (0/12)		0.0 (0/8)		0.0 (0/12)	
IgA1	Consensus	gp140	Con S gp140 CFI	RV144_wk26	58.3 (7/12)	531	50.0 (4/8)	352	16.7 (2/12)	730
IgA1	Consensus	gp140	Con S gp140 CFI	RV305_wk0	0.0 (0/12)		0.0 (0/8)		0.0 (0/12)	
IgA1	Consensus	gp140	Con S gp140 CFI	RV305_wk2	83.3 (10/12)	382	100 (8/8)	261	0.0 (0/12)	
IgA1	Consensus	gp140	Con S gp140 CFI	RV305_wk26	58.3 (7/12)	175	37.5 (3/8)	165	0.0 (0/12)	
IgA1	Consensus	gp140	Con S gp140 CFI	RV305_wk48	16.7 (2/12)	406	12.5 (1/8)	1416	0.0 (0/12)	
IgA1	Consensus	gp140	Con S gp140 CFI	RV305_wk72	16.7 (2/12)	277	12.5 (1/8)	825	0.0 (0/12)	
IgA1	Consensus CRF01_AE	gp140	HV 13700 AE.con.env03 140 CF	RV144_wk26	25.0 (3/12)	208	12.5 (1/8)	369	8.3 (1/12)	313
IgA1	Consensus CRF01_AE	gp140	HV 13700 AE.con.env03 140 CF	RV305_wk0	0.0 (0/12)		0.0 (0/8)		0.0 (0/12)	
IgA1	Consensus CRF01_AE	gp140	HV 13700 AE.con.env03 140 CF	RV305_wk2	66.7 (8/12)	788	75.0 (6/8)	295	0.0 (0/12)	
IgA1	Consensus CRF01_AE	gp140	HV 13700 AE.con.env03 140 CF	RV305_wk26	33.3 (4/12)	540	12.5 (1/8)	2658	0.0 (0/12)	
IgA1	Consensus CRF01_AE	gp140	HV 13700 AE.con.env03 140 CF	RV305_wk48	25.0 (3/12)	157	12.5 (1/8)	212	0.0 (0/12)	
IgA1	Consensus CRF01_AE	gp140	HV 13700 AE.con.env03 140 CF	RV305_wk72	16.7 (2/12)	128	12.5 (1/8)	112	0.0 (0/12)	
IgA1	B	V1V2	gp70_B.CaseA2 V1/V2/169K	RV144_wk26	0.0 (0/12)		0.0 (0/8)		0.0 (0/12)	
IgA1	B	V1V2	gp70_B.CaseA2 V1/V2/169K	RV305_wk0	0.0 (0/12)		0.0 (0/8)		0.0 (0/12)	
IgA1	B	V1V2	gp70_B.CaseA2 V1/V2/169K	RV305_wk2	25.0 (3/12)	542	12.5 (1/8)	376	0.0 (0/12)	
IgA1	B	V1V2	gp70_B.CaseA2 V1/V2/169K	RV305_wk26	0.0 (0/12)		0.0 (0/8)		0.0 (0/12)	
IgA1	B	V1V2	gp70_B.CaseA2 V1/V2/169K	RV305_wk48	0.0 (0/12)		0.0 (0/8)		0.0 (0/12)	
IgA1	B	V1V2	gp70_B.CaseA2 V1/V2/169K	RV305_wk72	0.0 (0/12)		0.0 (0/8)		0.0 (0/12)	
IgA1	B	V1V2	gp70_B.CaseA_V1_V2	RV144_wk26	0.0 (0/12)		0.0 (0/8)		0.0 (0/12)	
IgA1	B	V1V2	gp70_B.CaseA_V1_V2	RV305_wk0	0.0 (0/12)		0.0 (0/8)		0.0 (0/12)	
IgA1	B	V1V2	gp70_B.CaseA_V1_V2	RV305_wk2	25.0 (3/12)	196	12.5 (1/8)	161	0.0 (0/12)	
IgA1	B	V1V2	gp70_B.CaseA_V1_V2	RV305_wk26	8.3 (1/12)	201	0.0 (0/8)		0.0 (0/12)	
IgA1	B	V1V2	gp70_B.CaseA_V1_V2	RV305_wk48	0.0 (0/12)		0.0 (0/8)		0.0 (0/12)	

					Group 1: Combination		Group 2: AIDSVAX B/E		Group 3: ALVAC-HIV	
**Isotype**	**Clade**	**Env Region**	**Antigen**	**Study Week**	**Response Rate *(Responders / Total)***	**Median MFI *Responders***	**Response Rate *(Responders / Total)***	**Median MFI *Responders***	**Response Rate *(Responders / Total)***	**Median MFI *Responders***
IgA1	B	V3	B.MN V3 gp70	RV144_wk26	0.0 (0/12)		12.5 (1/8)	496	8.3 (1/12)	504
IgA1	B	V3	B.MN V3 gp70	RV305_wk0	0.0 (0/12)		0.0 (0/8)		0.0 (0/12)	
IgA1	B	V3	B.MN V3 gp70	RV305_wk2	41.7 (5/12)	1168	37.5 (3/8)	1081	0.0 (0/12)	
IgA1	B	V3	B.MN V3 gp70	RV305_wk26	25.0 (3/12)	1117	25.0 (2/8)	11742	0.0 (0/12)	
IgA1	B	V3	B.MN V3 gp70	RV305_wk48	0.0 (0/12)		12.5 (1/8)	2629	0.0 (0/12)	
IgA1	B	V3	B.MN V3 gp70	RV305_wk72	0.0 (0/12)		12.5 (1/8)	1238	0.0 (0/12)	
