# Supplementary material for: Viral vector delivered immunogen focuses HIV-1 antibody specificity and increases durability of the circulating antibody recall response
Source: PLoS Pathog. 2023 May 31;19(5):e1011359. doi: 10.1371/journal.ppat.1011359 (PMC10284421; doi:10.1371/journal.ppat.1011359)
Supplement: S14 Table — (PDF) [file ppat.1011359.s027.pdf]

S14 Table. IgA1 binding magnitude comparison at RV144 week 26 and RV305 week 2.

			IgA1 Median (Minimum-Maximum) MFI Magnitude	
**Category**	**Antigen**	**FDR P**	**RV144 Week 26**	**RV305 Week 2**
gp140: Immune correlates of risk	00MSA 4076 gp140	<0.0001	8 (1-134)	181 (1-6923)
	A1.con.env03 140 CF	<0.0001	1 (1-86)	20 (1-6300)
gp120: Vaccine strain antigens	A244 D11gp120_avi	<0.0001	159 (9-3390)	698 (65-14348)
	92TH023 gp120 gDneg 293F mon	<0.0001	32 (1-767)	317 (4-12023)
	MN gp120 gDneg/293F/mon	<0.0001	37 (1-1246)	274 (1-18632)
