# Supplementary material for: Viral vector delivered immunogen focuses HIV-1 antibody specificity and increases durability of the circulating antibody recall response
Source: PLoS Pathog. 2023 May 31;19(5):e1011359. doi: 10.1371/journal.ppat.1011359 (PMC10284421; doi:10.1371/journal.ppat.1011359)
Supplement: S15 Table — (PDF) [file ppat.1011359.s028.pdf]

S15 Table. IgA2 percent responders by antigen, study week, and group.

Isotype	Clade	HIV-1 Region	Antigen	Study Week	G1_ Combination Response Rate *(Responders/Total)*	G2_ AIDSVAX B/E Response Rate *(Responders/Total)*	G3_ ALVAC-HIV Response Rate *(Responders/Total)*
IgA2	B	gp120	MN gp120 gDneg/293F/mon	RV144_wk26	0.0 (0/12)	0.0 (0/8)	0.0 (0/12)
IgA2	B	gp120	MN gp120 gDneg/293F/mon	RV305_wk0	0.0 (0/12)	0.0 (0/8)	0.0 (0/12)
IgA2	B	gp120	MN gp120 gDneg/293F/mon	RV305_wk2	0.0 (0/12)	0.0 (0/8)	0.0 (0/12)
IgA2	B	gp120	MN gp120 gDneg/293F/mon	RV305_wk26	0.0 (0/12)	0.0 (0/8)	0.0 (0/12)
IgA2	B	gp120	MN gp120 gDneg/293F/mon	RV305_wk48	0.0 (0/12)	0.0 (0/8)	0.0 (0/12)
IgA2	B	gp120	MN gp120 gDneg/293F/mon	RV305_wk72	0.0 (0/12)	0.0 (0/8)	0.0 (0/12)
IgA2	Consensus	gp120	Con 6 gp120/B	RV144_wk26	0.0 (0/12)	0.0 (0/8)	0.0 (0/12)
IgA2	Consensus	gp120	Con 6 gp120/B	RV305_wk0	0.0 (0/12)	0.0 (0/8)	0.0 (0/12)
IgA2	Consensus	gp120	Con 6 gp120/B	RV305_wk2	0.0 (0/12)	0.0 (0/8)	0.0 (0/12)
IgA2	Consensus	gp120	Con 6 gp120/B	RV305_wk26	0.0 (0/12)	0.0 (0/8)	0.0 (0/12)
IgA2	Consensus	gp120	Con 6 gp120/B	RV305_wk48	0.0 (0/12)	0.0 (0/8)	0.0 (0/12)
IgA2	Consensus	gp120	Con 6 gp120/B	RV305_wk72	0.0 (0/12)	0.0 (0/8)	0.0 (0/12)
IgA2	CRF01_AE	gp120	92TH023 gp120 gDneg 293F mon	RV144_wk26	0.0 (0/12)	0.0 (0/8)	0.0 (0/12)
IgA2	CRF01_AE	gp120	92TH023 gp120 gDneg 293F mon	RV305_wk0	0.0 (0/12)	0.0 (0/8)	0.0 (0/12)
IgA2	CRF01_AE	gp120	92TH023 gp120 gDneg 293F mon	RV305_wk2	0.0 (0/12)	0.0 (0/8)	0.0 (0/12)
IgA2	CRF01_AE	gp120	92TH023 gp120 gDneg 293F mon	RV305_wk26	0.0 (0/12)	0.0 (0/8)	0.0 (0/12)
IgA2	CRF01_AE	gp120	92TH023 gp120 gDneg 293F mon	RV305_wk48	0.0 (0/12)	0.0 (0/8)	0.0 (0/12)
IgA2	CRF01_AE	gp120	92TH023 gp120 gDneg 293F mon	RV305_wk72	0.0 (0/12)	0.0 (0/8)	0.0 (0/12)
IgA2	CRF01_AE	gp120	A244 D11gp120_avi	RV144_wk26	0.0 (0/12)	0.0 (0/8)	0.0 (0/12)
IgA2	CRF01_AE	gp120	A244 D11gp120_avi	RV305_wk0	0.0 (0/12)	0.0 (0/8)	0.0 (0/12)
IgA2	CRF01_AE	gp120	A244 D11gp120_avi	RV305_wk2	0.0 (0/12)	0.0 (0/8)	0.0 (0/12)
IgA2	CRF01_AE	gp120	A244 D11gp120_avi	RV305_wk26	0.0 (0/12)	0.0 (0/8)	0.0 (0/12)
IgA2	CRF01_AE	gp120	A244 D11gp120_avi	RV305_wk48	0.0 (0/12)	0.0 (0/8)	0.0 (0/12)
IgA2	CRF01_AE	gp120	A244 D11gp120_avi	RV305_wk72	0.0 (0/12)	0.0 (0/8)	0.0 (0/12)
IgA2	A	gp140	00MSA 4076 gp140	RV144_wk26	0.0 (0/12)	0.0 (0/8)	0.0 (0/12)
IgA2	A	gp140	00MSA 4076 gp140	RV305_wk0	0.0 (0/12)	0.0 (0/8)	0.0 (0/12)
IgA2	A	gp140	00MSA 4076 gp140	RV305_wk2	0.0 (0/12)	0.0 (0/8)	0.0 (0/12)
IgA2	A	gp140	00MSA 4076 gp140	RV305_wk26	0.0 (0/12)	0.0 (0/8)	0.0 (0/12)
IgA2	A	gp140	00MSA 4076 gp140	RV305_wk48	0.0 (0/12)	0.0 (0/8)	0.0 (0/12)
IgA2	A	gp140	00MSA 4076 gp140	RV305_wk72	0.0 (0/12)	0.0 (0/8)	0.0 (0/12)

S15 Table continued

Isotype	Clade	HIV-1 Region	Antigen	Study Week	G1_ Combination Response Rate *(Responders/Total)*	G2_ AIDSVAX B/E Response Rate *(Responders/Total)*	G3_ ALVAC-HIV Response Rate *(Responders/Total)*
IgA2	A	gp140	A1.con.env03 140 CF	RV144_wk26	0.0 (0/12)	0.0 (0/8)	0.0 (0/12)
IgA2	A	gp140	A1.con.env03 140 CF	RV305_wk0	0.0 (0/12)	0.0 (0/8)	0.0 (0/12)
IgA2	A	gp140	A1.con.env03 140 CF	RV305_wk2	0.0 (0/12)	0.0 (0/8)	0.0 (0/12)
IgA2	A	gp140	A1.con.env03 140 CF	RV305_wk26	0.0 (0/12)	0.0 (0/8)	0.0 (0/12)
IgA2	A	gp140	A1.con.env03 140 CF	RV305_wk48	0.0 (0/12)	0.0 (0/8)	0.0 (0/12)
IgA2	A	gp140	A1.con.env03 140 CF	RV305_wk72	0.0 (0/12)	0.0 (0/8)	0.0 (0/12)
IgA2	Consensus	gp140	Con S gp140 CFI	RV144_wk26	0.0 (0/12)	0.0 (0/8)	0.0 (0/12)
IgA2	Consensus	gp140	Con S gp140 CFI	RV305_wk0	0.0 (0/12)	0.0 (0/8)	0.0 (0/12)
IgA2	Consensus	gp140	Con S gp140 CFI	RV305_wk2	0.0 (0/12)	0.0 (0/8)	0.0 (0/12)
IgA2	Consensus	gp140	Con S gp140 CFI	RV305_wk26	0.0 (0/12)	0.0 (0/8)	0.0 (0/12)
IgA2	Consensus	gp140	Con S gp140 CFI	RV305_wk48	0.0 (0/12)	0.0 (0/8)	0.0 (0/12)
IgA2	Consensus	gp140	Con S gp140 CFI	RV305_wk72	0.0 (0/12)	0.0 (0/8)	0.0 (0/12)
IgA2	Consensus CRF01_AE	gp140	HV 13700 AE.con.env03 140 CF	RV144_wk26	0.0 (0/12)	0.0 (0/8)	0.0 (0/12)
IgA2	Consensus CRF01_AE	gp140	HV 13700 AE.con.env03 140 CF	RV305_wk0	0.0 (0/12)	0.0 (0/8)	0.0 (0/12)
IgA2	Consensus CRF01_AE	gp140	HV 13700 AE.con.env03 140 CF	RV305_wk2	0.0 (0/12)	0.0 (0/8)	0.0 (0/12)
IgA2	Consensus CRF01_AE	gp140	HV 13700 AE.con.env03 140 CF	RV305_wk26	0.0 (0/12)	0.0 (0/8)	0.0 (0/12)
IgA2	Consensus CRF01_AE	gp140	HV 13700 AE.con.env03 140 CF	RV305_wk48	0.0 (0/12)	0.0 (0/8)	0.0 (0/12)
IgA2	Consensus CRF01_AE	gp140	HV 13700 AE.con.env03 140 CF	RV305_wk72	0.0 (0/12)	0.0 (0/8)	0.0 (0/12)
IgA2	B	V1V2	gp70_B.CaseA2 V1/V2/169K	RV144_wk26	0.0 (0/12)	0.0 (0/8)	0.0 (0/12)
IgA2	B	V1V2	gp70_B.CaseA2 V1/V2/169K	RV305_wk0	0.0 (0/12)	0.0 (0/8)	0.0 (0/12)
IgA2	B	V1V2	gp70_B.CaseA2 V1/V2/169K	RV305_wk2	0.0 (0/12)	0.0 (0/8)	0.0 (0/12)
IgA2	B	V1V2	gp70_B.CaseA2 V1/V2/169K	RV305_wk26	0.0 (0/12)	0.0 (0/8)	0.0 (0/12)
IgA2	B	V1V2	gp70_B.CaseA2 V1/V2/169K	RV305_wk48	0.0 (0/12)	0.0 (0/8)	0.0 (0/12)
IgA2	B	V1V2	gp70_B.CaseA2 V1/V2/169K	RV305_wk72	0.0 (0/12)	0.0 (0/8)	0.0 (0/12)
IgA2	B	V1V2	gp70_B.CaseA_V1_V2	RV144_wk26	0.0 (0/12)	0.0 (0/8)	0.0 (0/12)
IgA2	B	V1V2	gp70_B.CaseA_V1_V2	RV305_wk0	0.0 (0/12)	0.0 (0/8)	0.0 (0/12)
IgA2	B	V1V2	gp70_B.CaseA_V1_V2	RV305_wk2	0.0 (0/12)	0.0 (0/8)	0.0 (0/12)
IgA2	B	V1V2	gp70_B.CaseA_V1_V2	RV305_wk26	0.0 (0/12)	0.0 (0/8)	0.0 (0/12)
IgA2	B	V1V2	gp70_B.CaseA_V1_V2	RV305_wk48	0.0 (0/12)	0.0 (0/8)	0.0 (0/12)
IgA2	B	V1V2	gp70_B.CaseA_V1_V2	RV305_wk72	0.0 (0/12)	0.0 (0/8)	0.0 (0/12)

Isotype	Clade	HIV-1 Region	Antigen	Study Week	G1_ Combination Response Rate *(Responders/Total)*	G2_ AIDSVAX B/E Response Rate *(Responders/Total)*	G3_ ALVAC-HIV Response Rate *(Responders/Total)*
IgA2	B	V3	B.MN V3 gp70	RV144_wk26	0.0 (0/12)	0.0 (0/8)	0.0 (0/12)
IgA2	B	V3	B.MN V3 gp70	RV305_wk0	0.0 (0/12)	0.0 (0/8)	0.0 (0/12)
IgA2	B	V3	B.MN V3 gp70	RV305_wk2	0.0 (0/12)	0.0 (0/8)	0.0 (0/12)
IgA2	B	V3	B.MN V3 gp70	RV305_wk26	0.0 (0/12)	0.0 (0/8)	0.0 (0/12)
IgA2	B	V3	B.MN V3 gp70	RV305_wk48	0.0 (0/12)	0.0 (0/8)	0.0 (0/12)
IgA2	B	V3	B.MN V3 gp70	RV305_wk72	0.0 (0/12)	0.0 (0/8)	0.0 (0/12)
